# Silylium-Catalyzed Regio- and Stereoselective Carbosilylation of Ynamides with Allylic Trimethylsilanes

**DOI:** 10.1021/acs.orglett.3c00221

**Published:** 2023-02-07

**Authors:** Paz Yepes, Ángel L. Suárez-Sobrino, Miguel A. Rodríguez, Alfredo Ballesteros

**Affiliations:** † Departamento de Química Orgánica e Inorgánica, Instituto Universitario de Química Organometálica “Enrique Moles”, Universidad de Oviedo, Julián Clavería, 8, 33006 Oviedo, Spain; ‡ Departamento de Química, Centro de Investigación en Síntesis Orgánica, Universidad de la Rioja, Madre de Dios, 51, 26006 Logroño, Spain

## Abstract

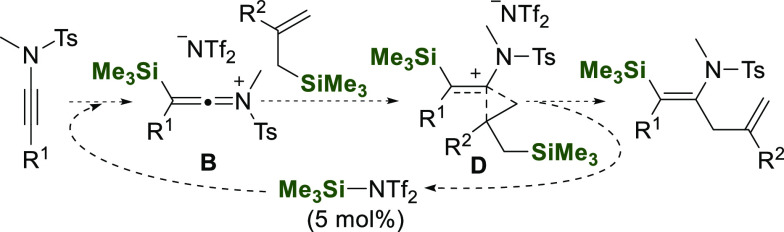

The regio- and stereoselective carbosilylation of tosylynamides with allylic trimethylsilanes takes place under mild conditions in the presence of catalytic TMSNTf_2_ or HNTf_2_ to give (*Z*)-α-allyl-β-trimethylsilylenamides with good yields. Theoretical calculations show the activation of the C–C triple bond of the ynamides by the trimethylsilylium ion and formation of a β-trimethylsilylketenimonium cation. Further transformations of the products demonstrate the synthetic utility of this reaction.

Silylium ion has been consolidated in the past two decades as a potent catalyst in organic synthesis.^1^ Its strong Lewis acid character is reflected in its high affinity not only to σ- but also to π-bases. This makes silylium ion a simpler and more sustainable alternative to catalytic metal salts or transition metal complexes for the activation of C–C multiple bonds. Since the pioneering work of Lambert et al., employing 1,1-disubstituted alkenes,^2^ several examples of silylium-catalyzed hydro-^3−6^ and carbosilylation^7^ of C–C double bonds have appeared. However, there are few precedents related to the activation of triple C–C bonds with silylium ion.^8^ In this context, Kawashima et al. recently described a silylium catalyzed intermolecular silylation of an arylalkyne^9^ to form a β-silyl stabilized vinylcation, that was subsequently intercepted by an intramolecular Friedel–Crafts ring closure.

Exploring new ways for the activation of electron rich triple C–C bonds, ynamides could be good candidates to broad the scope of silylium ion catalysis. The tendency of ynamides to be activated by electrophilic species such as acids and transition metals,^10^ and the polarization of their C–C triple bond allow many regioselective reactions.^10,11^ Moreover, the 1,2 functionalization of ynamides offers the possibility to obtain functionalized and highly substituted nitrogenated alkenes.^12^ Thus, the silylation of these compounds represents an entry to nitrogen-substituted vinylsilanes,^13^ of great value in organic synthesis.

Several methods to install a silyl group in one of the carbon atoms of ynamides have been described (Figure 1). Thus, α,β-silylmetalation and subsequent attack of an electrophile to the metal position is the most common method for this purpose (Figure 1A); for instance, the silylcupration^14^ and the Pd-catalyzed silylstannation^15^ of ynamides result in α-metalated (*Z*)-β-silylenamides; alternatively, Pd-catalyzed silylboration leads to β-metalated (*Z*)-α-silylenamides;^16^ finally, α-metalated (*E*)-β-silylenamides can be obtained from a *trans*-selective radical silylzincation of ynamides.^17^

**Figure 1:**
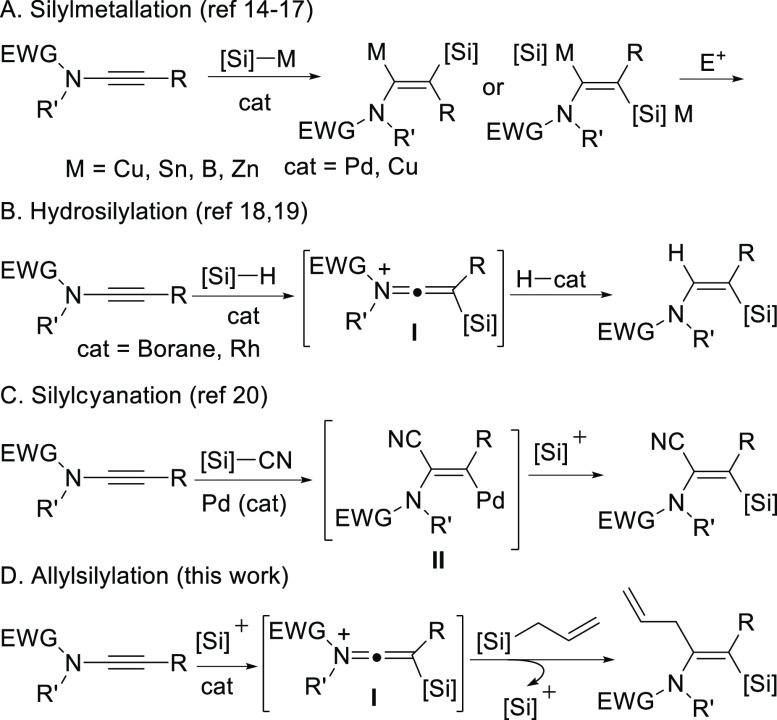
Ynamide silylation methods.

Apart from the silylmetalation approach, β-silyl-(*Z*)-enamides can also selectively be obtained by hydrosilylation of ynamides using a rhodium complex^18^ or tris(pentafluorophenyl)borane^19^ as catalyst; in both cases, the hydride abstraction from the silane by the corresponding catalyst leads to a silylium ion, responsible for the formation of a β-silyl ketenimonium intermediate **I** (Figure 1B). Very recently, a palladium catalyzed silylcyanation was described; the reaction proceeds in a stereo- and regioselective way through a β-palladium enamide intermediate **II** (Figure 1C).^20^

On the other hand, the precedented reactions of allylsilanes with C–C multiple bonds catalyzed by Brønsted or Lewis acids^21−23^ and the possibility of self-regeneration of catalytic silylium moved us to choose allylsilane derivatives as carbon nucleophile counterparts for our study on the catalytic carbosilylation of ynamides (Figure 1D). Therefore, herein we develop a regio- and stereoselective allylsilylation of ynamides, using catalytic silylium ion, an alternative to other species such as metal salts and transition metal complexes.

Our first experiments focused on the reaction between tosylynamide **1a** and allyltrimethylsilane **2a** in 1,2-dichloroethane (DCE) using a direct silylium ion freshly prepared source like *N*-trimethylsilyl bis(trifluoromethanesulfonyl)imide, TMSNTf_2_,^24^ or an acid like bis(trifluoromethanesulfonyl)imide, HNTf_2_, as initiators (10 mol %).^25^ To our delight, we obtained in both cases the corresponding allylsilylated enamide **3a**, in 50% and 35% yield, respectively, with complete regio- and stereoselectivity (Table 1, entries 1 and 2).

**Table 1:**
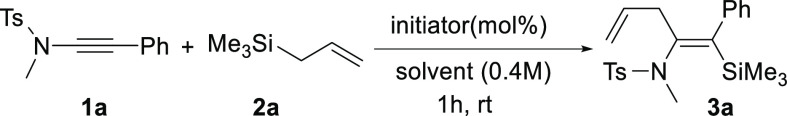
Optimization of the Reaction of Ynamide **1a** and Allylsilane **2a**^a^

entry	initiator (mol %)	**2a** (equiv)	solvent	yield^b^ (%)
1	TMSNTf_2_ (10)	2	DCE	50
2	HNTf_2_ (10)	2	DCE	35
3	TMSOTf (10)	2	DCE	
4	TMSNTf_2_ (10)	4	DCE	54
5	HNTf_2_ (10)	4	DCE	65
6	TMSNTf_2_ (5)	4	DCE	56
7	HNTf_2_ (5)	4	DCE	66, 71^c^
8	HNTf_2_ (5)	4	DCM	64
9	HNTf_2_ (5)	4	Et_2_O	16
10	HNTf_2_ (5)	4	toluene	28
11	HNTf_2_ (5)	4	THF	
12	HNTf_2_ (5)	4	CH_3_CN	

a **1a** (0.1 mmol, 1 equiv), **2a** (equiv), initiator (mol %), solvent (0.4 M).

b Isolated yield after flash chromatography purification on silica gel.

c 2 mmol scale.

We employed also trimethylsilyl trifluoromethanesulfonate, TMSOTf, as initiator in the same reaction conditions, but in this case, we observed only decomposition of the reagents (Table 1, entry 3). Then, the yields were improved by increasing the ratio of allylsilane **2a** to 4-fold excess (Table 1, entries 4,5); additionally, we observed that lowing the initiator loading to 5 mol % did not seem to affect the efficiency of the reaction (Table 1, entries 6 and 7). Furthermore, comparable yields were obtained with other halogenated solvents such as dichloromethane (Table 1, entry 8); however, the yields were significantly lower with diethyl ether or toluene (Table 1 entries 9 and 10), or simply the reaction did not afford any product when using THF or acetonitrile (Table 1, entries 11 and 12). Finally, the reaction was scaled-up to 2 mmol employing 5 mol % HNTf_2_ with an improved yield of 71% (Table 1, entry 7).

With the optimized conditions in hand, we examined the scope of the reaction. We employed either TMSNTf_2_ (method A) or HNTf_2_ (method B) as initiators with different β-aryl-*N*-methyl-*N*-tosylynamides **1** and allylsilane **2a** (Scheme 1). Thus, α-allyl-β-silyl-(*Z*)-enamides **3** were obtained with complete regio- and stereoselectivity and good yields (56–77%). In this way, it was proved that the reaction works well with different aryl-substituted ynamides bearing either electron-withdrawing (R = F, Br, CF_3_, products **3b**–**d**,**g**,**h**) or electron-donating (R = Me, MeO, products **3e**,**f**) substituents at different positions of the aryl moiety.

**Scheme 1:**
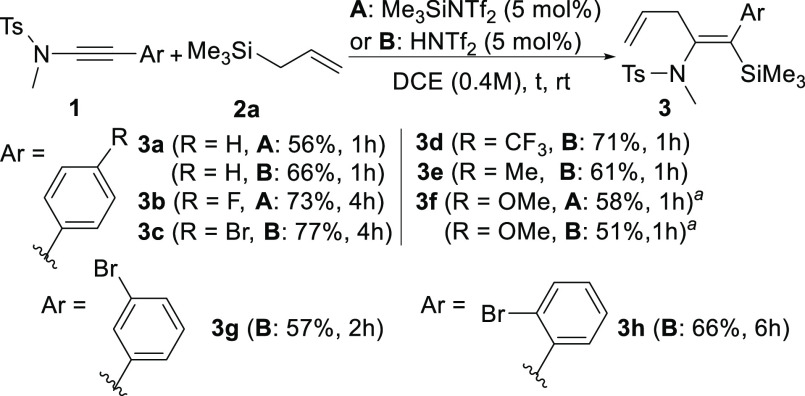
Reaction of Ynamides **1** and Allyltrimethylsilane **2a** 10 mol % initiator, DCE (0.2 M).

Then, we explored the reactivity of different 2-substituted allylsilanes **2** with a variety of β-aryl-substituted ynamides **1** (Scheme 2). The results were similar or even better than those previously obtained for the parent allyltrimethylsilane **2a** (R^2^ = H). Thus, trimethyl(2-methylallyl)silane **2b** gave good yields with diverse ynamides (Scheme 2, R^2^ = Me, products **3i**–**n**); furthermore, other 2-substituted allylsilanes like trimethyl-(2-phenylally)silane **2c** gave also excellent results (Scheme 2, R^2^ = Ph, products **3o**–**u**). Interestingly, product **3o** was obtained almost quantitatively (99%) when the reaction was performed in a 2 mmol scale (Scheme 2). In addition, the structures of products **3j** and **3q** were unambiguously confirmed by X-ray resolution.^26^ Continuing our study on 2-arylsubstituted allylsilanes, [2-(4-chlorophenyl)allyl]trimethylsilane **2d** (R^2^ = 4*-*ClC_6_H_4_) and [2-(4-*t*-buthylphenyl)allyl]trimethylsilane **2e** (R^2^ = ^*t*^BuC_6_H_4_) were also employed to obtain the corresponding silylenamides again with good yields (Scheme 2 products **3v**,**w** and **3x**, respectively). Regarding other substitution in the ynamide, we also checked a β-alkyl tosylynamide (R^1^ = *n*-butyl) with allylsilanes **2a**,**b** (R^2^ = H, Me) in similar reaction conditions, but in this case only complex mixtures were obtained.^27^ However, when an alkenyl β-substituted tosylynamide (Scheme 2, R^1^ = cinnamyl) was reacted in the same reaction conditions with allylsilane **2c** (R^2^ = Ph), the expected β-silyl-(*Z*)-enamide **3y** was obtained in 51% yield (Scheme 2).

**Scheme 2:**
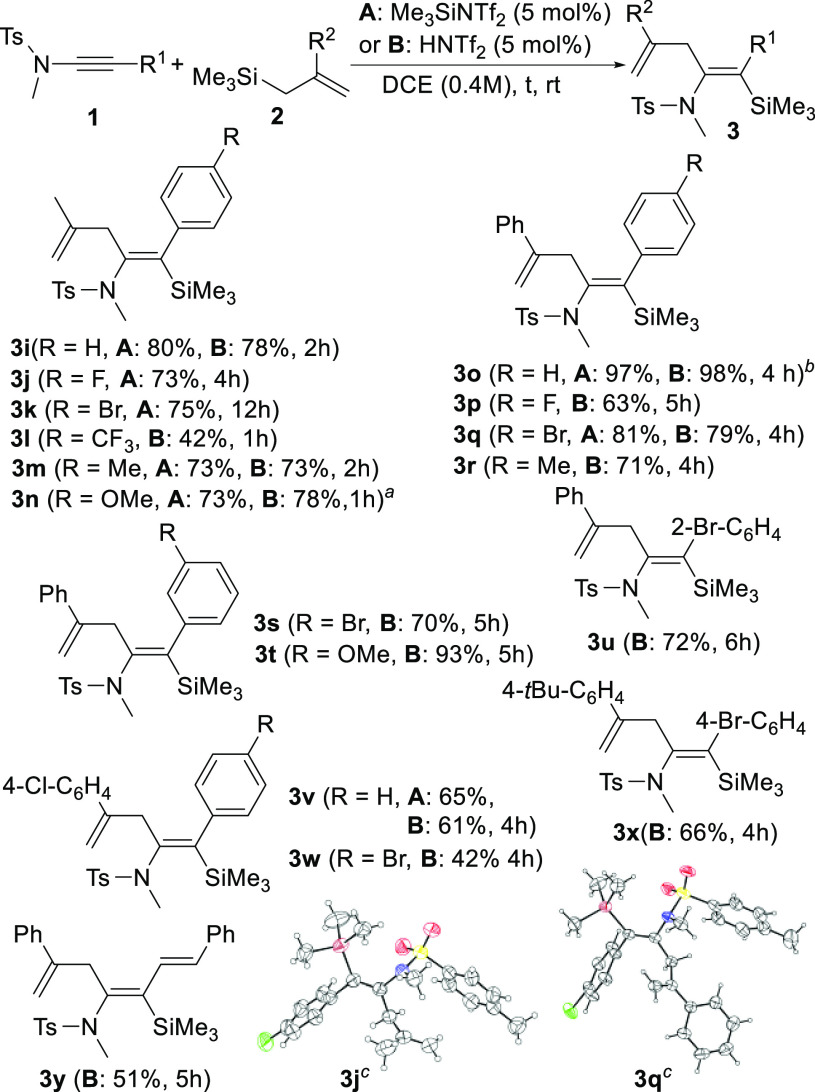
Reaction of Ynamides **1** with 2-Substituted Allyltrimethylsilanes **2** 10 mol % initiator, DCE (0.2 M). 99% 2 mol scale, method B. Ellipsoids at 50% of probability level.

To get some insight into the reaction mechanism, we performed computational studies at the PCM-M05-2X/6-31G*//M05-2X/6-31G* level.^26^ Starting from tosylynamide **1a** and TMSNTf_2_, the molecular geometry was fully optimized without any molecular symmetry constraint, leading to structure **A** (Figure 2), a coordination minimum that placed the Si–C_Ph_ distance at 4.065 Å, keeping the Si–N bond distance at 1.895 Å. Subsequently, the approach between Si and C_Ph_ (Si–C_Ph_ distance = 2.195 Å) induces an elongation between Si and N (Si–N distance = 2.538 Å), giving rise to transition state **TS1** (+10.3 kcal·mol^–1^), in which the SiMe_3_ moiety is rather flat (C–Si–C–C dihedral angle = 165.4°). **TS1** evolves to **B** (+6.2 kcal·mol^–1^), with formation of the Si–C_Ph_ bond (Si–C_Ph_ distance = 2.058 Å) and cleavage of the Si–N bond (Si–N distance = 2.906 Å). As also shown in Figure 2, the *anti*-approximation of allyltrimethylsilane **2a** to **B** gave the coordination minimum **C**_*anti*_ (+0.4 kcal·mol^–1^), with a distance of 4.093 Å between H_2_C_=_ and C_N_, which is reduced in the transition state **TS2**_*anti*_ (+8.8 kcal·mol^–1^) to 2.048 Å. **TS2**_*anti*_ led to the minimum **D**_*anti*_ (−1.2 kcal·mol^–1^), which in fact has a cyclopropyl structure (bond distances: H_2_C–C_N_ = 1.592 Å, HC–C_N_ = 1.565 Å, and H_2_C–HC = 1.464 Å). Finally, the attack of the Tf_2_N^–^ anion on the silicon atom acts as the driving force of the process, leading directly to the coordination minimum **E**_*anti*_ (−41.4 kcal·mol^–1^), formed by the allylsilylated enamide **3a** and TMSNTf_2_, without any intermediate being located. Likewise, *syn*-addition from the coordination minimum **C**_*syn*_ (+2.1 kcal·mol^–1^) leads to the minimum **D**_*syn*_ (−2.9 kcal·mol^–1^) through the transition state **TS2**_*syn*_ (+13.6 kcal·mol^–1^), which shows a distance of 2.300 Å between H_2_C_=_ and C_N_. The difference in the energy barriers to reach **TS2**_*anti*_ or **TS2**_*syn*_ (4.8 kcal·mol^–1^) could be explained by the β-silicon effect,^19^ which places the bond angles in **B** at 107° (Si–C_Ph_–C_N_) and 131° (Ph–C_Ph_–C_N_), which avoid *syn*-approximation and allow us to explain the experimentally found stereoselectivity.^26^

**Figure 2:**
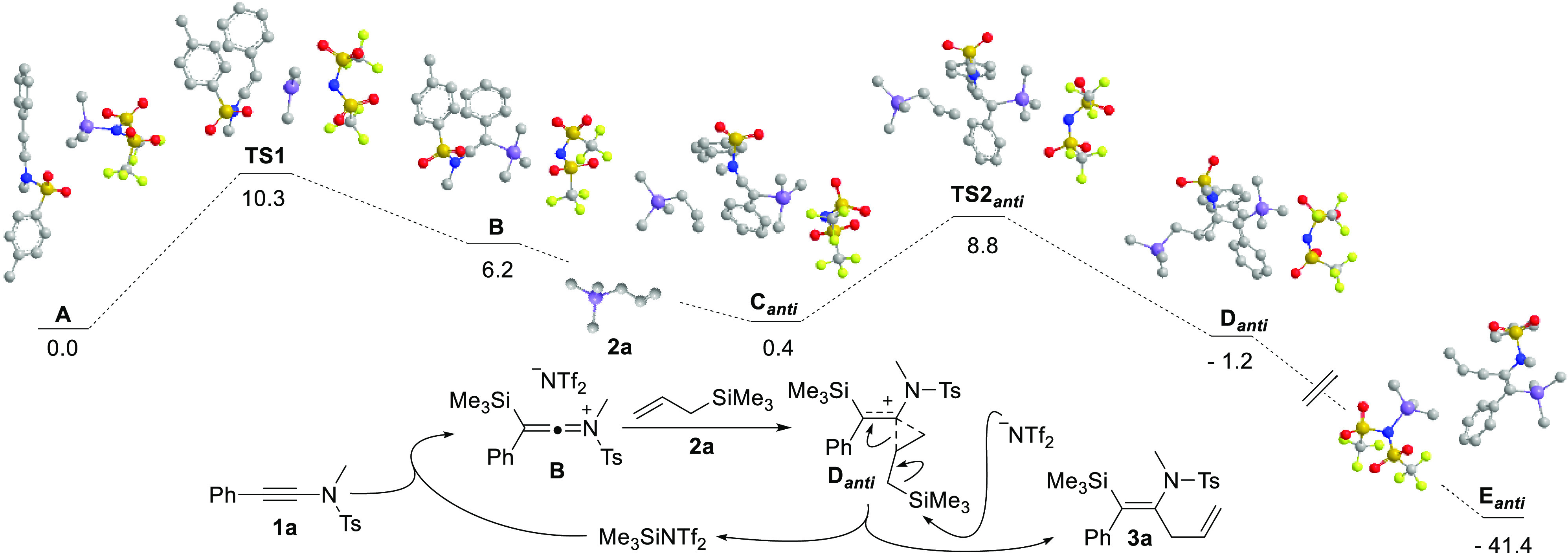
Calculated relative energy profile for the formation of β-silylenamides, in kcal·mol^–1^ (for the sake of comparison, the values shown for **A**, **TS1**, and **B** also include the energy value of **2a**) and overall reaction of the proposed catalytic cycle. H atoms have been omitted for clarity.

According to our calculations, the overall process would start from the reaction of **1a** with TMSNTf_2_ and formation of a β-silyl ketenimonium intermediate **B** and the Tf_2_N^–^ anion. Intermediate **B** would receive nucleophilic *anti-*attack of the C–C double bond of the allylic silane **2a** to give the intermediate **D**_*anti*_. The subsequent attack of the Tf_2_N^–^ anion on the silicon atom leads to β-silylenamide **3a** and TMSNTf_2_, which closes the catalytic cycle (Figure 2).

Finally, to illustrate the synthetic possibilities of enamides **3**, we carried out several transformations (Scheme 3). Thus, the reaction of β-silylenamide **3o** with a fluoride source, such as tetrabutylammonium fluoride, led to the desilylated enamide **4** (86%) or, alternatively, the coupling product **5** (72%) if the reaction was performed in the presence of 4-bromobenzaldehyde (Scheme 3). The allyl group can also intervene in other transformations; thus, the presence of a catalytic amount of a Brønsted acid (HNTf_2_, 1 mol %) gave the 1,2-dihydronaphthalene derivative **6** (65%) because of an intramolecular aromatic electrophilic substitution of the carbocation intermediate formed by previous protonation of the 2-phenylallyl substituent (Scheme 3).

**Scheme 3:**
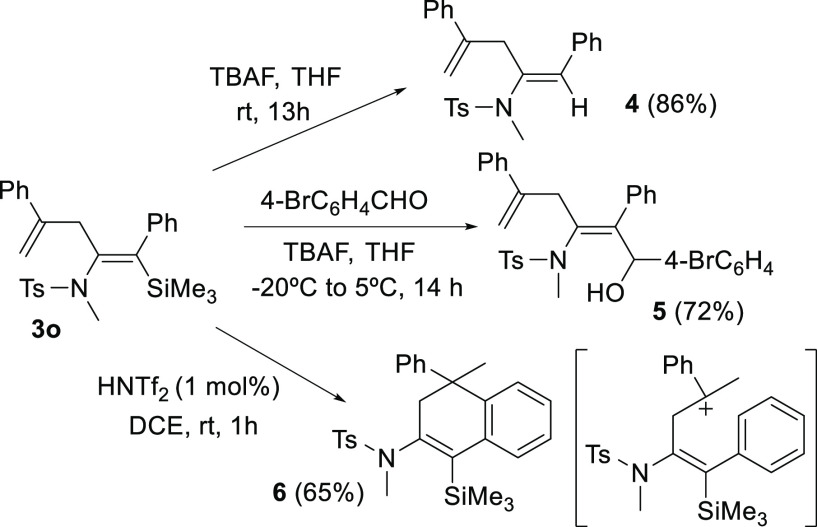
Further Transformations of silylenamide **3o**

In summary, we have described a regio- and stereoselective carbosilylation of tosylynamides **1** catalyzed by silylium ion. The reaction uses different allylsilanes **2** as the source of the carbon nucleophile and the silicon electrophile. The silylium ion activates the triple C–C bond of the ynamide to produce an electrophilic β-silylketenimonium intermediate **B**, the subsequent nucleophilic attack by the allylsilane **2** produces the regeneration of the silylium ion to close the catalytic cycle. Theoretical calculations support this mechanistic picture. This versatile reaction leads to (*Z*)-β-silylenamides **3**, interesting building blocks as demonstrated by the possibility of further transformations. Finally, this reaction represents a novel example of catalytic activation of electron rich alkynes by silylium ion.

## Data Availability

The data underlying this study are available in the published article and its online Supporting Information.

## Supporting Information Available

Experimental details, materials and methods, characterization data, NMR spectra for all compounds, X-ray diffraction experiments and computational studies data (PDF)

## Dedication

Dedicated to the memory of Professor Gregorio Asensio.
