# Supplementary material for: Silylium-Catalyzed Regio- and Stereoselective Carbosilylation of Ynamides with Allylic Trimethylsilanes
Source: Org Lett. 2023 Feb 7;25(6):1020–4. doi: 10.1021/acs.orglett.3c00221 (PMC9942199; doi:10.1021/acs.orglett.3c00221)
Supplement: Supplementary file 1 — ol3c00221_si_001.pdf [file ol3c00221_si_001.pdf]

Silylium-Catalyzed Regio- and Stereoselective Carbosilylation of Ynamides with Allylic Trimethylsilanes

Paz Yepes,[a] Ángel L. Suárez–Sobrino,[§] Miguel A. Rodríguez[b] and Alfredo Ballesteros,[a]*

[a]Departamento de Química Orgánica e Inorgánica; Instituto de Química Organometálica "Enrique Moles"
Universidad de Oviedo
Julián Clavería, 8, 33006, Oviedo (Spain)

[b]Departamento de Química; Centro de Investigación en Síntesis Orgánica
Universidad de la Rioja
Madre de Dios 51, 26006, Logroño (Spain)

Corresponding author email address: abg@uniovi.es

Supporting Information

1.- General Information...S2

2.- Experimental Procedures and Characterization Data..................................S2

3.- NMR Spectra ……………………………………………………………...S16

4.- X-Ray Crystal Structure Determination..S68

5.- Theoretical Calculations..S70

6.- References …………………………………………………………...S84

1.- General Information

All reactions were carried out using oven-dried glassware under an atmosphere of argon (99.999 %). Solvents were dried by standard methods and distilled prior to use. The solvents used in column chromatography, hexane and ethyl acetate were obtained from commercial suppliers and used without further distillation. TLC was performed on aluminum-backed plates coated with silica gel 60 with F254 indicator, using UV light as a visualizing agent as well as vanillin ethanol solution as developing agent. Flash chromatography was performed on silica gel 60 (230-400 mesh). ^{1}H NMR, ^{13}C NMR, ^{19}F and ^{29}Si spectra were measured in $CDCl_3$ or DMSO at room temperature (unless otherwise noted) on a Bruker DPX-300, Bruker AV-300 MHz, Bruker NAV-400 with TMS (δ = 0.0 ppm) as internal standard. Data are reported as follows: chemical shift (ppm), multiplicity (s: singlet, d: doublet, t: triplet, q quadruplet, brd: broad), coupling constants and integration. Carbon multiplicities were assigned by DEPT techniques.
High-resolution mass spectra (HRMS) were obtained by electron electrospray ionization (ESI) techniques with a VG AutoSpec M mass Spectrometers and a microTOF focus (Bruker Daltonics, Bremen Germany).
Melting points (m.p.) of recrystallized samples were measured in a Buchi-Tottoli apparatus and were not corrected. Crystal measurements were performed in an Oxford Diffraction Xcalibur Nova diffractometer.

2.- Experimental Procedures and Characterization Data

Ynamides 1

Ynamides **1** were synthesized according to chemical procedures described in the literature.[1] All ynamides used were known[2-9] and their spectral data were consistent with those reported in the literature.

Ynamide 1a[2]

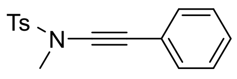

^{1}H NMR (300 MHz, $CDCl_3$) δ = 7.87 (d, *J* = 8.2 Hz, 2H), 7.45–7.25 (m, 7H), 3.18 (s, 3H), 2.48 (s, 3H).

Ynamide 1b[3]

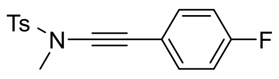

^{1}H NMR (300 MHz, $CDCl_3$) δ = 7.75 (d, *J* = 8.3 Hz, 2H), 7.37–7.23 (m, 4H), 6.97–6.83 (m, 2H), 3.07 (s, 3H), 2.34 (s, 3H).

Ynamide 1c[3]

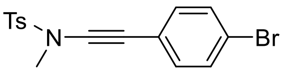

^{1}H NMR (300 MHz, $CDCl_3$) δ = 7.84 (d, *J* = 8.2 Hz, 2H), 7.48–7.35 (m, 4H), 7.30–7.17 (d, 2H), 3.17 (s, 3H), 2.49 (s, 3H).

Ynamide 1d[4]

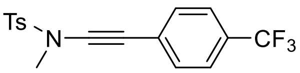

^{1}H NMR (300 MHz, $CDCl_3$) δ = 7.86 (d, *J* = 8.3 Hz, 2H), 7.56 (d, *J* = 8.1 Hz, 2H), 7.46 (d, *J* = 8.1 Hz, 2H), 7.40 (d, *J* = 7.9 Hz, 2H), 3.20 (s, 3H), 2.49 (s, 3H).

Ynamide 1e[3]

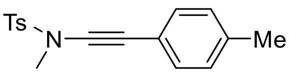

^{1}H NMR (300 MHz, $CDCl_3$) δ = 7.86 (d, *J* = 8.3 Hz, 2H), 7.39 (d, *J* = 7.9, 2H), 7.28 (d, *J* = 8.0 Hz, 2H), 7.12 (d, *J* = 7.8, 2H), 3.16 (s, 3H), 2.48 (s, 3H), 2.36 (s, 3H).

Ynamide 1f[3]

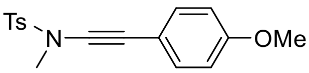

^{1}H NMR (300 MHz, $CDCl_3$) δ = 7.86 (d, *J* = 8.4 Hz, 2H, 7.39 (d, *J* = 7.9 Hz, 2H), 7.33 (d, *J* = 8.8 Hz, 2H), 6.84, (d, *J* = 8.7 Hz, 2H), 3.83 (s, 3H), 3.16 (s, 3H), 2.49 (s, 3H).

Ynamide 1g[5]

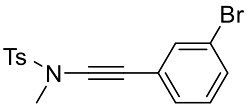

^{1}H NMR (300 MHz, $CDCl_3$) δ = 7.84 (d, *J* = 8.4 Hz, 2H), 7.50 (t, *J* = 1.8 Hz, 1H), 7.46-7.36 (m, 3H), 7.30 (d, *J* = 7.8 Hz, 1H), 7.17 (t, *J* = 7.9 Hz, 1H), 3.17 (s, 3H), 2.48 (s, 3H). ^{13}C NMR (75 MHz, $CDCl_3$) δ = 145.1 (C), 133.8 (CH), 133.1 (C), 130.9 (C), 129.9 (CH), 129.7 (CH), 127.8 (CH), 124.8 (C), 122.1 (C), 85.3 (C), 67.9 (C), 39.2 (CH_3), 21.7 (CH_3). HRMS (ESI) m/z: $[M + Na]^+$ Calcd for $C_{16}H_{14}BrNNaO_2S$ 385.9821; Found 385.9824.

Ynamide 1h[6]

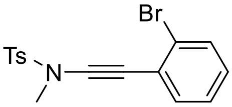

^{1}H NMR ($CDCl_3$) δ = 7.92 (d, *J* = 8.1 Hz, 2H), 7.56 (d, *J* = 7.8 Hz, 1H), 7.46-7.34 (m, 3H), 7.32–7.21 (m, 1H), 7.19–7.09 (m, 1H), 3.21 (s, 3H), 2.48 (s, 3H).

Ynamide 1i[7]

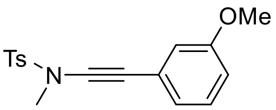

^{1}H NMR (300 MHz, $CDCl_3$) δ = 7.86 (d, *J* = 8.3 Hz, 2H), 7.39 (d, *J* = 7.9 Hz, 2H), 7.22 (t, *J* = 7.9 Hz, 1H), 6.97 (d, *J* = 7.5 Hz, 1H), 6.91 (s, 1H), 6.89–6.83 (m, 1H), 3.82 (s, 3H), 3.17 (s, 3H), 2.44 (s, 3H).

Ynamide 1j[8]

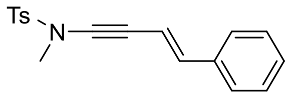

^{1}H NMR (300 MHz, $CDCl_3$) δ = 7.85 (d, *J* = 8.2 Hz, 2H), 7.42–7.23 (m, 7H), 6.86 (d, *J* = 16.2 Hz, 1H), 6.24 (d, *J* = 16.2 Hz, 1H), 3.14 (s, 3H), 2.49 (s, 3H).

Ynamide 1k[9]

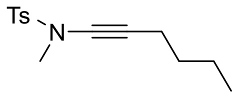

^{1}H NMR (300 MHz, $CDCl_3$) δ = 7.77 (d, *J* = 8.3 Hz, 2H), 7.35 (d, *J* = 8.0 Hz, 2H), 3.00 (s, 3H), 2.45 (s, 3H), 2.23 (t, *J* = 6.7 Hz, 2H), 1.53–1.29 (m, 4H), 0.89 (t, *J* = 7.2 Hz, 3H).

Allylic silanes 2

Allyltrimethylsilane **2a** and trimethyl(2-methylallyl)silane **2b** were purchased; (2-arylallyl)trimethylsilanes **2c-e** were synthesized by the methodology described in the literature.[10] These compounds were known and their spectral data were consistent with those reported in the literature.[10,11]

Allylic silane 2c[10]

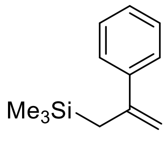

^{1}H NMR (300 MHz, $CDCl_3$) δ = 7.48-7.21 (m, 5H), 7.38–7.26 (m, 4H), 5.16 (brds, 1H), 4.90 (s, 1H), 2.05 (s, 2H), – 0.07 (s, 9H). ^{13}C NMR (75 MHz, $CDCl_3$) δ = 146.8 (C), 142.9 (C), 128.2 (CH), 127.3 (CH), 126.5 (CH), 110.2 (CH_2), 26.3 (CH_2), – 1.3 (CH_3).

Allylic silane 2d[11]

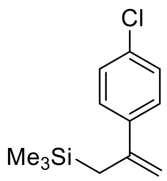

^{1}H NMR (300 MHz, $CDCl_3$) δ = 7.35 (d, *J* = 8.5Hz, 2H), 7.28 (d, *J* = 8.6 Hz, 2H), 5.13 (brds, 1H), 4.89 (brds, 1H), 2.00 (s, 2H), – 0.08 (s, 3H). ^{13}C NMR (75 MHz, $CDCl_3$) δ = 145.5 (C), 141.2 (C), 137.9 (C), 128.2 (CH), 127.6 (CH), 110.6 (CH_2), 21.0 (CH_2), – 1.4 (CH_3).

Allylic silane 2e[10]

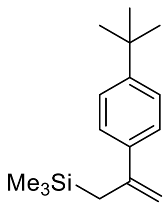

^{1}H NMR (300 MHz, $CDCl_3$) δ = 7.41-7.25 (m, 4H), 5.15 (brds, 1H), 4.85 (brds, 1H), 2.79–2.60 (m, 1H), 2.44 (s, 3H), 2.02 (s, 2H), 1.33 (s, 3H), – 0.06 (s, 3H). ^{13}C NMR (75 MHz, $CDCl_3$) δ = 150.3 (C), 146.3 (C), 139.8 (C), 126.0 (CH), 125.0 (CH), 109.4 (CH_2), 34.6 (C), 31.5 (CH_3), 26.1 (CH_2), – 1.2 (CH_3).

General procedure for the preparation of α-allyl-β-silyl enamides 3. Methods A and B.

A solution of allylsilane **2** (0.4 mmol, 4 equiv.) and the corresponding catalyst: $TMSNTf_2$ (0,05 mL of a 0.1 M solution in dichloroethane, 0.005 mmol, 5 mol%, method A) or $HNTf_2$ (0,05 mL of a 0.1 M solution in dichloroethane, 0,005 mmol, 5 mol%, method B) in 0,25 mL dichloroethane (0,4 M) was stirred under argon atmosphere for 10 min at rt. Then, the corresponding ynamide **1** (0.1 mmol, 1 equiv) was added and the mixture stirred. The reaction was monitored by TLC until the complete disappearance of the ynamide **1**. Then, solid K_2CO_3 (5 mg, 35 mol%) was added, and the solvent removed. The resulting crude was purified by column chromatography (SiO_2, hexanes/ethyl acetate 5:1).

Scale-up preparation of 3a: The same reaction conditions were used employing 2 mmol (570 mg) of ynamide **1a**, 8 mmol (930 mg) of allyltrimethylsilane **2c** and 0.1 mmol (5 mol%) of $HNTf_2$ (1 mL of a 0,1 M solution in dichloroethane) in 5 mL of dichloromethane (0,4 M), to get 568 mg of pure **3a** (71%).

Scale-up preparation of 3o: The same reaction conditions were used employing 2 mmol (570 mg) of ynamide **1a** 8 mmol (1.52 g) of phenylallyltrimethylsilane **2c** and 0.1 mmol (5 mol%) of $HNTf_2$ (1 mL of a 0,1 M solution in dichloroethane) in 5 mL of dichloromethane (0,4 M), to get 942 mg of pure **3o** (99%) after purification by column chromatography (SiO_2, hexanes/ethyl acetate 5:1).

Enamide 3a

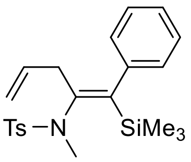

The product was obtained, after 1 h of reaction time and column chromatography purification (SiO_2, hexanes/ethyl acetate 5:1) in 56% (22 mg) (method A, 21 mg) or 66% (method B, 26 mg) yield as a crystalline white solid; m. p. 67–69 ºC; ^{1}H NMR (300 MHz, $CDCl_3$) δ = 7.88-7.65 (m, 2H), 7.38–7.26 (m, 4H), 7.23–7.18 (m, 1H), 7.08–6.88 (m, 2H), 5.52–5.22 (m, 1H), 4.66 (dd, J = 10.0, 1.7 Hz, 1H), 4.30 (dd, J = 17.0, 1.7 Hz, 1H), 3.10 (s, 3H), 2.79–2.60 (m, 1H), 2.44 (s, 3H), 2.34–2.16 (m, 1H), 0.19 (s, 9H); ^{13}C NMR (75 MHz, $CDCl_3$) δ =148.9 (C), 145.6 (C), 143.3 (C), 141.9 (C), 136.8 (C), 136.0 (CH), 129.5 (CH), 128.0 (CH), 127.9 (CH), 127.5 (CH), 125.8 (CH), 116.1 (CH_2), 39.2 (CH_3), 36.6 (CH_2), 21.5 (CH_3), –0.3 (CH_3); ^{29}Si NMR (75 MHz, $CDCl_3$) δ = – 6.0; HRMS (ESI) m/z: $[M + H]^+$ Calcd for $C_{22}H_{30}NO_2SSi$ 400.1761; Found 400.1773.

Enamide 3b

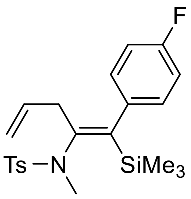

The product was obtained, after 4 h of reaction time and column chromatography purification (SiO_2, hexanes/ethyl acetate 5:1) in 73% yield (method A, 30 mg) as a crystalline white solid; m. p. 84–86 ºC; ^{1}H NMR (300 MHz, $CDCl_3$) δ = 7.76 (d, J = 8.2 Hz, 2H), 7.33 (d, J = 7.3 Hz, 2H), 7.12–6.81 (m, 4H), 5.47–5.24 (m, 1H), 4.67 (dd, J = 10.0, 1.3 Hz, 1H), 4.32 (dd, J = 17.0, 1.3 Hz, 1H), 3.08 (s, 3H), 2.75–2.58 (m, 1H), 2.45 (s, 3H), 2.33–2.13

(m, 1H), 0.18 (s, 9 H); ^{13}C NMR (75 MHz, $CDCl_3$) δ = 161.3 (C, d, *J* = 244.2 Hz), 148.2 (C), 146.1 (C), 143.4 (C), 137.6 (C, d, *J* = 3.0 Hz), 136.6 (C), 135.7 (CH), 129.6 (CH), 129.1 (CH, d, *J* = 7.7 Hz), 127.9 (CH), 116.3 (CH_2), 115.0 (CH, d, *J* = 21.4 Hz), 39.0 (CH_3), 36.5 (CH_2), 21.5 (CH_3), – 0.3 (CH_3); ^{29}Si NMR (75 MHz, $CDCl_3$) δ = – 6.0; ^{19}F NMR (282 MHz, $CDCl_3$) δ = –117.3; HRMS (ESI) m/z: $[M + Na]^+$ Calcd for $C_{22}H_{28}FNNaO_2SSi$ 440.1486; Found 440.1489.

Enamide 3c

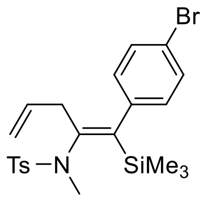

The product was obtained, after 4 h of reaction time and column chromatography purification (SiO_2, hexanes/ethyl acetate 5:1) in 77% yield (method B, 37 mg) as a crystalline white solid; m. p. 98–100 ºC; ^{1}H NMR (300 MHz, $CDCl_3$) δ = 7.76 (d, *J* = 8.0 Hz, 2H), 7.44 (d, *J* = 8 Hz, 2H), 7.33 (d, *J* = 8.0 Hz, 2H), 6.95–6.80 (m, 2H), 5.45–5.20 (m, 1H), 4.60 (dd, *J* = 10.1, 1.6 Hz, 1H), 4.33 (dd, *J* = 17.0, 1.6 Hz, 1H), 3.07 (s, 3H), 2.75-2.55 (m, 1H), 2.45 (s, 3H), 2.33–2.10 (m, 1H), 0.18 (s, 9H); ^{13}C NMR (75 MHz, $CDCl_3$) δ =147.9 (C), 147.6 (C), 145.9 (C), 143.4 (C), 140.9 (C), 135.6 (CH), 131.2 (CH), 129.6 (CH), 129.4 (CH), 127.9 (CH), 119.8 (C), 116.4 (CH_2), 38.9 (CH_3), 36.5 (CH_2), 21.5 (CH_3), – 0.3 (CH_3); ^{29}Si NMR (75 MHz, $CDCl_3$) δ = – 6.0; HRMS (ESI) m/z: $[M + H]^+$ Calcd for $C_{22}H_{29}BrNO_2SS$ 478.0872; Found 478.0866.

Enamide 3d

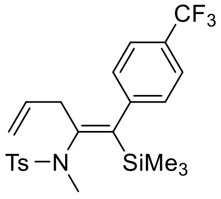

The product was obtained, after 1 h of reaction time and column chromatography purification (SiO_2, hexanes/ethyl acetate 5:1) in 71% yield (method B, 33 mg) as a crystalline white solid; m. p. 84–86 ºC; ^{1}H NMR (300 MHz, $CDCl_3$) δ = 7.77 (d, *J* = 8.2 Hz, 2H), 7.58 (d, *J* = 7.9 Hz, 2H), 7.34 (d, *J* = 8.0 Hz, 2H), 7.23–7.02 (m, 2H), 5.43–5.25 (m, 1H), 4.69 (dd, *J* = 10.1, 1.6 Hz, 1H), 4.33 (dd, *J* = 17.0, 1.6 Hz, 1H), 3.09 (s, 3H), 2.69–2.51 (m, 1H), 2.45 (s, 3H), 2.36–2.15 (m, 1H), 0.19 (s, 9 H); ^{13}C NMR (75 MHz, $CDCl_3$) δ = 148.1 (C), 145.9 (C), 145.8 (C), 143.5 (C), 136.5 (C), 135.4 (CH), 129.7 (CH), 128.2 (C, q, *J* = 32.6 Hz), 127.9 (CH), 127.8 (CH), 125.0 (CH), 124.3 (C, q, *J* = 270 Hz), 116.6 (CH_2), 38.9 (CH_3), 36.6 (CH_2), 21.5 (CH_3), – 0.3 (CH_3); ^{29}Si NMR (75 MHz, $CDCl_3$) δ = – 5.6; ^{19}F NMR (282 MHz, $CDCl_3$) δ = – 62.3; HRMS (ESI) m/z: $[M + H]^+$ Calcd for $C_{23}H_{29}F_3NO_2SSi$ 468.1635; Found 468.1644.

Enamide 3e

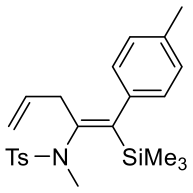

The product was obtained after 1 h of reaction time and column chromatography purification (SiO_2, hexanes/ethyl acetate 5:1) in 61% yield (method B, 25 mg) as a crystalline white solid; m. p. 90–92 ºC ^{1}H NMR (300 MHz, $CDCl_3$) δ = 7.79 (d, *J* = 8.2 Hz, 2H), 7.33 (d, *J* = 7.9 Hz, 2H), 7.13 (d, *J* = 7.6 Hz, 2H), 6.89 (d, *J* = 7.5 Hz, 2H), 6.51–5.27 (m, 1H), 4.66 (d, *J* = 10.0 Hz, 1H), 4.31 (d, *J* = 17.0 Hz, 1H), 3.11 (s, 3H), 2.88–2.64 (m,

1H), 2.44 (s, 3H), 2.37–2.16 (m, 1H), 2.43 (s, 3H), 0.20 (s, 9H); ^{13}C NMR (75 MHz, $CDCl_3$) δ =148.7 (C), 145.6 (C), 143.3 (C), 138.9 (C), 136.8 (C), 136.2 (CH), 135.2 (C), 129.6 (CH), 128.8 (CH), 127.9 (CH), 127.5 (CH), 116.1 (CH_2), 39.2 (CH_3), 36.6 (CH_2), 21.5 (CH_3), 21.1, (CH_3), – 0.2 (CH_3); ^{29}Si NMR (75 MHz, $CDCl_3$) δ = – 6.3; HRMS (ESI) m/z: [M + H]$^+$ Calcd for $C_{23}H_{32}NO_2SSi$ 414.1918; Found 414.1924.

Enamide 3f

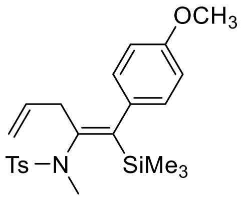

The product was obtained after 1 h of reaction time in DCE (0.2 M) with 10 mol% catalyst and column chromatography purification (SiO_2, hexanes/ethyl acetate 5:1) in 58% yield (method A, 25 mg) or 51% (method B, 22 mg) as a white gum; ^{1}H NMR (300 MHz, $CDCl_3$) δ = 7.77 (d, *J* = 8.3 Hz, 2H), 7.33 (d, *J* = 8.0 Hz, 2H), 6.98–6.85 (m, 4H), 5.57–5.30 (m, 1H), 4.65 (dd, *J* = 10.0, 1.6 Hz, 1H), 4.30 (dd, *J* = 17.0, 1.6 Hz, 1H), 3.82 (s, 3H), 3.09 (s, 3H), 2.83–2.64 (m, 1H), 2.45 (s, 3H), 2.30–2.13 (m, 1H), 0.17 (s, 9 H); ^{13}C NMR (75 MHz, $CDCl_3$) δ = 157.7 (C), 148.4 (C), 145.9 (C), 143.3 (C), 136.8 (C), 136.1 (CH), 134.1 (C), 129.5 (CH), 128.6 (CH), 127.9 (CH), 116.0 (CH_2), 113.5 (CH), 55.2 (CH_3), 39.1 (CH_3), 36.5 (CH_2), 21.5 (CH_3), – 0.3 (CH_3); ^{29}Si NMR (75 MHz, $CDCl_3$) δ = – 6.2; HRMS (ESI) m/z: [M + Na]$^+$ Calcd for $C_{23}H_{31}NNaO_3SSi$ 452.1686; Found 452.1690.

Enamide 3g

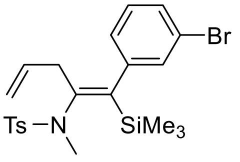

The product was obtained, after 2 h of reaction time and column chromatography purification (SiO_2, hexanes/ethyl acetate 5:1) in 57% yield (method B, 27 mg) as a white gum; ^{1}H NMR (300 MHz, $CDCl_3$) δ = 7.77 (d, *J* = 8.3 Hz, 2H), 7.39–7.30 (m, 3H), 7.26–7.06 (m, 2H), 7.03–6.83 (m, 1H), 5.48–5.22 (m, 1H), 4.70 (d, *J* = 10.1, 1H), 4.31 (d, *J* = 16.9 Hz, 1H), 3.08 (s, 3H), 2.75–2.54 (m, 1H), 2.45 (s, 3H), 2.37–2.17 (m, 1H), 0.19 (s, 9H); ^{13}C NMR (75 MHz, $CDCl_3$) δ =147.7 (C), 146.2 (C), 144.1 (C), 143.4 (C), 136.5 (C), 135.4 (CH), 130.4 (CH), 129.6 (CH), 128.9 (CH), 127.9 (CH), 126.3 (CH), 122.1 (C), 116.5 (CH_2), 39.0 (CH_3), 36.7 (CH_2), 21.5 (CH_3), – 0.3 (CH_3); ^{29}Si NMR (75 MHz, $CDCl_3$) δ = – 5.7; HRMS (ESI) m/z: [M + H]$^+$ Calcd for $C_{22}H_{29}BrNO_2SSi$ 478.0866; Found 478.0875.

Enamide 3h

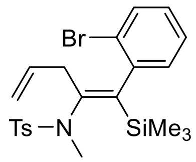

The product was obtained, after 6 h of reaction time and column chromatography purification (SiO_2, hexanes/ethyl acetate 5:1) in 66 % yield (method B, 32 mg) as a crystalline white solid; m. p. 84–86 °C; ^{1}H NMR (300 MHz, $CDCl_3$) δ = 7.82 (d, *J* = 8.0 Hz, 2H), 7.64–7.49 (m, 1H), 7.40–7.20 (m, 1H), 7.34 (d, *J* = 8.0 Hz, 2H), 7.19–6.95 (m, 2H), 5.66–5.23 (m, 1H), 4.59 (d, *J* = 10.0 Hz, 1H), 4.16 (brd, *J* = 16.9, 1H), 3.14 (s, 3H), 2.74-2.53 (m, 2H), 2.45 (s, 3H), 0.21 (s, 9 H); ^{13}C NMR (75 MHz, $CDCl_3$) δ = 147.6 (C), 143.4 (C), 142.7 (C), 136.6 (C), 134.7

(CH), 132.6 (CH), 129.5 (CH), 129.2 (CH), 128.2 (CH), 127.6 (CH), 127.4 (CH), 122.7 (C), 116.7 (CH_2), 39.9 (CH_3), 37.7 (CH_2), 21.5 (CH_3), – 0.2 (CH_3); ^{29}Si NMR (75 MHz, $CDCl_3$) δ = – 6.2; HRMS (ESI) m/z: $[M + H]^+$ Calcd for $C_{22}H_{29}BrNO_2SSi$ 478.0866; Found 478.0864.

Enamide 3i

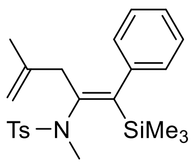

The product was obtained, after 2 h of reaction time and column chromatography purification (SiO_2, hexanes/ethyl acetate 5:1) in 80% (method A, 33 mg) or 78 % (method B, 32 mg) yield as a crystalline white solid; m. p. 118–119 ºC, ^{1}H NMR (300 MHz, $CDCl_3$) δ = 7.79 (d, *J* = 8.2 Hz, 2H), 7.37–7.24 (m, 4H), 7.23–7.15 (m, 1H), 7.06–6.95 (m, 2H), 4.44 (s, 1H), 3.94 (m, 1H), 3.12 (s, 3H), 2.74–2.54 (m, 1H), 2.44 (s, 3H), 2.37–2.19 (m, 1H), 1.39 (s, 3H), 0.19 (s, 9H); ^{13}C NMR (75 MHz, $CDCl_3$) δ =149.9 (C), 144.6 (C), 143.3 (C), 142.5 (C), 142.0 (C), 137.3 (C), 129.5 (CH), 127.9 (CH), 127.7 (CH), 125.7 (CH), 116.1 (CH_2), 39.8 (CH_2), 38.6 (CH_3), 22.4 (CH_3), 21.5 (CH_3), – 0.2 (CH_3); ^{29}Si NMR (75 MHz, $CDCl_3$) δ = – 6.1; HRMS (ESI) m/z: $[M + H]^+$ Calcd for $C_{23}H_{32}NO_2SSi$ 414.1918; Found 414.1925.

Enamide 3j

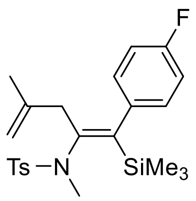

The product was obtained after 4 h of reaction time and column chromatography purification (SiO_2, hexanes/ethyl acetate 5:1) in 73 % yield (method A, 32 mg) as a crystalline white solid; m. p. 128–129 ºC; ^{1}H NMR (300 MHz, $CDCl_3$) δ = 7.77 (d, *J* = 8.4 Hz, 2H), 7.34 (d, *J* = 7.0 Hz, 2H), 7.07–6.89 (m, 4H), 4.46 (s, 1H), 4.00 (s, 1H), 3.09 (s, 3H), 2.70–2.41 (m, 1H), 2.44 (s, 3H), 2.32–2.13 (m, 1H), 1.37 (s, 3H), 0.19 (s, 9H); ^{13}C NMR (75 MHz, $CDCl_3$) δ = 161.2 (C, d, *J* = 244.3 Hz), 149.1 (C), 145.2 (C), 143.3 (C), 142.3 (C), 137.7 (C, d, *J* = 3.4 Hz), 137.1 (C), 129.5 (CH), 129.1 (CH, d, *J* = 7.7 Hz), 127.9 (CH), 114.8 (CH, d, *J* = 21.1Hz), 112.6 (CH_2), 39.7 (CH_2), 38.3 (CH_3), 22.4 (CH_3), 21.5 (CH_3), – 0.3 (CH_3); ^{29}Si NMR (75 MHz, $CDCl_3$) δ = – 5.9; ^{19}F NMR (282 MHz, $CDCl_3$) δ = –117.4; HRMS (ESI) m/z: $[M + Na]^+$ Calcd for $C_{23}H_{30}FNNaO_2SSi$ 454.1643; Found 454.1645.

Enamide 3k

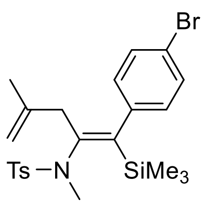

The product was obtained, after 12 h of reaction time and column chromatography purification (SiO_2, hexanes/ethyl acetate 5:1) in 75% yield (method A, 37 mg) as a crystalline white solid; m. p. 105–106 ºC; ^{1}H NMR (300 MHz, $CDCl_3$) δ = 7.76 (d, *J* = 8.3 Hz, 2H), 7.42 (d, *J* = 8.5 Hz, 2H), 7.33 (d, *J* = 9.1 Hz, 2H), 6.69 (d, *J* = 7.6 Hz, 2H), 4.47 (s, 1H), 4.00 (s, 1H), 3.08 (s, 3H), 2.68–2.49 (m, 1H), 2.44 (s, 3H), 2.34–2.10 (m, 1H), 1.37 (s, 3H), 0.19 (s, 9H); ^{13}C NMR (75 MHz, $CDCl_3$) δ =148.8 (C), 144.8 (C), 143.3 (C), 142.2 (C), 140.9 (C), 136.9 (C), 131.0 (CH), 129.5 (CH), 129.3 (CH), 127.8 (CH), 119.7 (C), 112.7 (CH_2), 39.7 (CH_2), 38.2 (CH_3), 22.4 (CH_3),

21.5 (CH_3), – 0.3 (CH_3); ^{29}Si NMR (75 MHz, $CDCl_3$) δ = – 5.9; HRMS (ESI) m/z: $[M + H]^+$ Calcd for $C_{23}H_{31}BrNO_2SSi$ 492.1023; Found 492.1030.

Enamide 3l

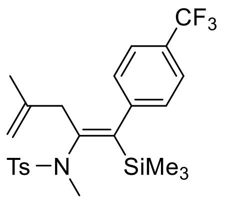

The product was obtained, after 1 h of reaction time and column chromatography purification (SiO_2, hexanes/ethyl acetate 5:1) in 42% yield (method B, 20 mg) as a crystalline white solid; m. p. 111–113 ºC: ^{1}H NMR (300 MHz, $CDCl_3$) δ = 7.77 (d, J = 8.0 Hz, 2H), 7.56 (d, J = 7.9 Hz, 2H), 7.33 (d, J = 7.9 Hz, 2H), 7.14 (d, J = 7.6 Hz, 2H), 4.49 (s, 1H), 4.03 (s, 1H), 3.10 (s, 3H), 2.61–2.38 (m, 1H), 2.44 (s, 3H), 2.34–2.15 (m, 1H), 1.36 (s, 3H), 0.20 (s, 9H); ^{13}C NMR (75 MHz, $CDCl_3$) δ =149.1 (C), 146.0 (C), 144.9 (C), 143.4 (C), 142.1 (C), 136.9 (C), 129.6 (CH), 128.1 (C, q, J = 32.2 Hz), 128.0 (CH), 127.9 (CH), 124.9 (CH), 124.3 (C, q, J = 270.2 Hz), 112.8 (CH_2), 39.8 (CH_2), 38.2 (CH_3), 22.4 (CH_3), 21.5 (CH_3), – 0.3 (CH_3); ^{29}Si NMR (75 MHz, $CDCl_3$) δ = – 5.9; ^{19}F NMR (282 MHz, $CDCl_3$) δ = – 62.3; HRMS (ESI) m/z: $[M + H]^+$ Calcd for $C_{24}H_{31}F_3NO_2SSi$ 482.179; Found 482.1793.

Enamide 3m

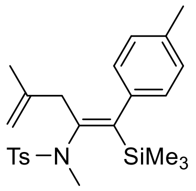

The product was obtained, after 2 h of reaction time and column chromatography purification (SiO_2, hexanes/ethyl acetate 5:1) in 73% yield (method A and B, 31 mg) as a crystalline white solid; m. p. 97–99 ºC; ^{1}H NMR (300 MHz, $CDCl_3$) δ = 7.70 (d, J = 8.3 Hz, 2H), 7.32 (d, J = 8.1 Hz, 2H), 7.10 (d, J = 7.6 Hz, 2H), 6.90 (d, J = 7.6 Hz, 2H), 4.44 (s, 1H), 3.94 (s, 1H), 3.12 (s, 3H), 2.80–2.56 (m, 1H), 2.43 (s, 3H), 2.33 (s, 3H), 2.30–2.19 (m, 1H), 1.39 (s, 3H), 0.20 (s, 9H); ^{13}C NMR (75 MHz, $CDCl_3$) δ =149.7 (C), 144.6 (C), 143.0 (C), 142.5 (C), 138.8 (C), 137.3 (C), 135.0 (C), 129.4 (CH), 128.4 (CH), 127.8 (CH), 127.4 (CH), 112.5 (CH_2), 39.7 (CH_2), 38.5 (CH_3), 22.3 (CH_3), 21.4 (CH_3), 21.0 (CH_3), – 0.3 (CH_3); ^{29}Si NMR (75 MHz, $CDCl_3$) δ = – 6.4; HRMS (ESI) m/z: $[M + H]^+$ Calcd for $C_{24}H_{34}NO_2SSi$ 428.2074; Found 428.2080.

Enamide 3n

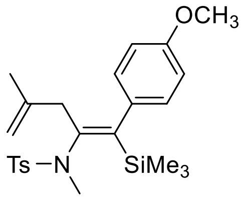

The product was obtained, after 1 h of reaction time in DCE (0.2 M) with 10 mol% catalyst and column chromatography purification (SiO_2, hexanes/ethyl acetate 5:1) in 73% (method A, 32 mg) or 78 % (method B, 35 mg) as a white gum; ^{1}H NMR (300 MHz, $CDCl_3$) δ = 7.78 (d, J = 8.3 Hz, 2H), 7.32 (d, J = 7.9 Hz, 2H), 6.91 (d, J = 8.1 Hz, 2H), 6.84 (d, J = 9.0 Hz, 2H), 4.43 (s, 1H), 3.93 (s, 1H), 3.81 (s, 3H), 3.10 (s, 3H), 2.75–2.58 (m, 1H), 2.44 (s, 3H), 2.34–2.14 (m, 1H), 1.38 (s, 3H), 0.19 (s, 9H); ^{13}C NMR (75 MHz, $CDCl_3$) δ =157.7 (C), 149.5 (C), 145.0 (C), 143.1 (C), 142.6 (C), 137.3 (C), 134.2 (C), 129.5 (CH), 128.7 (CH), 127.9 (CH), 113.3 (CH),

112.5 (CH_2), 55.1 (CH_3), 39.7 (CH_2), 36.5 (CH_3), 22.4 (CH_3), 21.5 (CH_3), – 0.2 (CH_3); ^{29}Si NMR (75 MHz, $CDCl_3$) δ = – 6.2; HRMS (ESI) m/z: $[M + Na]^+$ Calcd for $C_{24}H_{33}NNaO_3SSi$ 466.1843; Found 466.1841.

Enamide 3o

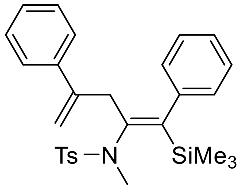

The product was obtained after 4 h of reaction time and column chromatography purification (SiO_2, hexanes/ethyl acetate 5:1) in 97% (method A, 46 mg) or 98% (method B, 47 mg) yield as a crystalline white solid; m. p. 100–101 ºC; ^{1}H NMR (300 MHz, $CDCl_3$) δ = 7.77 (d, *J* = 8.0 Hz, 2H), 7.46–6.88 (m, 10H), 6.85–6.67 (m, 2H), 5.12 (s, 1H), 4.77 (s, 1H), 3.20-2.94 (m, 1H), 3.04 (s, 3H), 2.69–2.47 (m, 1H), 2.42 (s, 3H), 0.20 (s, 9H); ^{13}C NMR (75 MHz, $CDCl_3$) δ =150.8 (C), 144.1 (C), 143.6 (C), 143.3 (C), 141.7 (C), 141.0 (C), 136.8 (C), 129.6 (CH), 127.9 (CH), 127.85 (CH), 127.8 (CH), 127.6 (CH), 127.3 (CH), 125.9 (CH), 125.8 (CH), 114.4 (CH_2), 38.6 (CH_3), 36.9 (CH_2), 21.5 (CH_3), – 0.2 (CH_3); ^{29}Si NMR (75 MHz, $CDCl_3$) δ = – 5.7; HRMS (ESI) m/z: $[M + H]^+$ Calcd for $C_{28}H_{34}NO_2SS$ 476.2074; Found 476.2071.

Enamide 3p

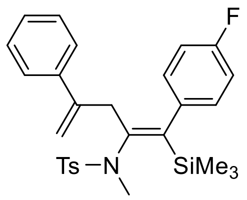

The product was obtained, after 5 h of reaction time and column chromatography purification (SiO_2, hexanes/ethyl acetate 5:1) in 63% yield (method B, 31 mg) as a crystalline white solid; m. p. 137–138 ºC; ^{1}H NMR (300 MHz, $CDCl_3$) δ = 7.77 (d, *J* = 8.2 Hz, 2H), 7.30 (d, *J* = 7.9 Hz, 2H), 7.22–7.10 (m, 3H), 7.07–6.88 (m, 4H), 6.85–6.71 (m, 2H), 5.14 (s, 1H), 4.77 (s, 1H), 3.14–2.95 (m, 1H), 3.04 (s, 3H), 2.71–2.48 (m, 1H), 2.43 (s, 3H), 0.19 (s, 9H); ^{13}C NMR (75 MHz, $CDCl_3$) δ = 161.3 (C, d, *J* = 244.3 Hz), 149.9 (C), 145.0 (C), 144.2 (C), 143.4 (C), 140.9 (C), 137.4 (C, d, *J* = 3.4 Hz), 136.7 (C), 129.6 (CH), 129.1 (CH, d, *J* = 7.7 Hz), 128.0 (CH), 127.8 (CH), 127.4 (CH), 125.8 (CH), 114.8 (CH, d, *J* = 21.1Hz), 114.4 (CH_2), 38.4 (CH_3), 36.8 (CH_2), 21.5 (CH_3), – 0.2 (CH_3); ^{29}Si NMR (75 MHz, $CDCl_3$) δ = – 5.6; ^{19}F NMR (282 MHz, $CDCl_3$) δ = – 117.4; HRMS (ESI) m/z: $[M + H]^+$ Calcd for $C_{28}H_{33}FNO_2SSi$ 494.1980; Found 494.1979.

Enamide 3q

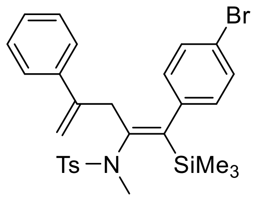

The product was obtained, after 4 h of reaction time and column chromatography purification (SiO_2, hexanes/ethyl acetate 5:1) in 81% (method A, 45 mg) or 79% yield (method B, 44 mg) as a crystalline white solid; m. p. 134–135 ºC; ^{1}H NMR (300 MHz, $CDCl_3$) δ = 7.75 (d, *J* = 8.2 Hz, 2H), 7.42 (d, *J* = 8.0 Hz, 2H), 7.29 (d, *J* = 6.9 Hz, 2H), 7.24–7.08 (m, 3H), 6.97–6.83 (m, 2H), 6.77 (d, *J* = 6.7 Hz, 2H), 5.13 (s, 1H), 4.75 (s, 1H), 3.15–2.89 (m, 1H), 3.02 (s, 3H), 2.68–2.47 (m, 1H), 2.42 (s, 3H), 0.20 (s, 9H); ^{13}C NMR (75 MHz, $CDCl_3$) δ =149.8 (C), 145.0 (C), 144.0 (C), 143.4 (C), 140.8 (C), 140.7 (C), 136.6 (C), 131.0 (CH), 129.7 (CH), 129.4 (CH), 128.0 (CH), 127.8 (CH), 127.5 (CH), 125.8 (CH), 120.0 (C), 114.4 (CH_2), 38.4 (CH_3), 36.9 (CH_2), 21.5 (CH_3), –

0.2 (CH_3); ^{29}Si NMR (75 MHz, $CDCl_3$) δ = – 5.6; HRMS (ESI) m/z: $[M + Na]^+$ Calcd for $C_{28}H_{32}BrNNaO_2SSi$ 576.0999; Found 576.1008.

Enamide 3r

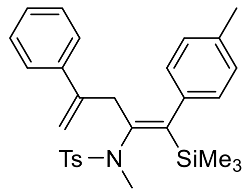

The product was obtained, after 4 h of reaction time and column chromatography purification (SiO_2, hexanes/ethyl acetate 5:1) in 71% yield (method B, 35 mg) as a crystalline white solid; m. p. 132–134 ºC; ^{1}H NMR (300 MHz, $CDCl_3$) δ = 7.77 (d, *J* = 8.1 Hz, 2H), 7.28 (d, *J* = 8.1 Hz, 2H), 7.23–7.06 (m, 5H), 6.98–6.87 (m, 2H), 6.80 (d, *J* = 6.1 Hz, 2H), 5.14 (s, 1H), 4.77 (s, 1H), 3.23–3.05 (m, 1H), 3.03 (s, 3H), 2.69–2.48 (m, 1H), 2.42 (s, 3H), 2.34 (s, 3H), 0.21 (s, 9H); ^{13}C NMR (75 MHz, $CDCl_3$) δ =150.7 (C), 145.1 (C), 143.6 (C), 143.2 (C), 140.9 (C), 138.6 (C), 136.8 (C), 135.4 (C), 129.6 (CH), 128.6 (CH), 127.9 (CH), 127.8 (CH), 127.5 (CH), 127.3 (CH), 125.9 (CH), 114.3 (CH_2), 38.6 (CH_3), 36.8 (CH_2), 21.5 (CH_3), 21.1 (CH_3), – 0.1 (CH_3); ^{29}Si NMR (75 MHz, $CDCl_3$) δ = – 5.9; HRMS (ESI) m/z: $[M + Na]^+$ Calcd for $C_{29}H_{35}NNaO_2SSi$ 512.2050; Found 512.2069.

Enamide 3s

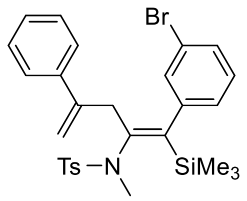

The product was obtained after 5 h of reaction time and column chromatography purification (SiO_2, hexanes/ethyl acetate 5:1) in 70% yield (method B, 39 mg) as a white gum; ^{1}H NMR (300 MHz, $CDCl_3$) δ = 7.77 (d, *J* = 8.2 Hz, 2H), 7.39–7.26 (m, 4H), 7.23–7.12 (m, 4H), 7.00–6.87 (m, 1H), 6.83–6.71 (m, 2H), 5.11 (s, 1H), 4.66 (s, 1H), 3.17–2.87 (m, 1H), 3.03 (s, 3H), 2.72–2.56 (m, 1H), 2.43 (s, 3H), 0.20 (s, 9H); ^{13}C NMR (75 MHz, $CDCl_3$) δ =149.5 (C), 145.1 (C), 144.5 (C), 143.9 (C), 143.4 (C), 140.8 (C), 136.7 (C), 130.5 (CH), 129.7 (CH), 129.5 (CH), 129.1 (CH), 128.0 (CH), 127.8 (CH), 127.5 (CH), 126.3 (CH), 125.9 (CH), 121.9 (C), 114.7 (CH_2), 38.6 (CH_3), 37.2 (CH_2), 21.5 (CH_3), – 0.2 (CH_3); ^{29}Si NMR (75 MHz, $CDCl_3$) δ = – 5.3; HRMS (ESI) m/z: $[M + Na]^+$ Calcd for $C_{28}H_{32}BrNNaO_2SSi$ 576.0999; Found 576.1014.

Enamide 3t

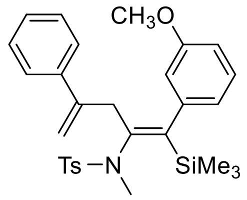

The product was obtained after 5 h of reaction time and column chromatography purification (SiO_2, hexanes/ethyl acetate 5:1) in 93% yield (method B, 55 mg) as white gum; ^{1}H NMR (300 MHz, $CDCl_3$) δ = 7.77 (d, *J* = 8.3 Hz, 2H), 7.37–7.09 (m, 7H), 6.93–6.74 (m, 3H), 6.70–6.59 (m, 1H), 5.14 (s, 1H), 4.77 (s, 1H), 3.81 (s, 3H), 3.24–3.00 (m, 1H), 3.05 (s, 3H), 2.76–2.50 (m, 1H), 2.42 (s, 3H), 0.22 (s, 9H); ^{13}C NMR (75 MHz, $CDCl_3$) δ =159.1 (C), 150.5 (C), 145.3 (C), 143.7 (C), 143.3 (C), 143.2 (C), 140.9 (C), 136.8 (C), 129.6 (CH), 128.9 (CH), 127.9 (CH), 127.8 (CH), 127.3 (CH), 125.9 (CH), 120.2 (CH), 114.5

(CH_2), 113.4 (CH), 111.3 (CH), 55.2 (CH_3), 38.7 (CH_3), 37.0 (CH_2), 21.5 (CH_3), – 0.1 (CH_3); ^{29}Si NMR (75 MHz, $CDCl_3$) δ = – 5.8; HRMS (ESI) m/z: $[M + H]^+$ Calcd for $C_{29}H_{36}NO_3SSi$ 506.2180; Found 506.2196.

Enamide 3u

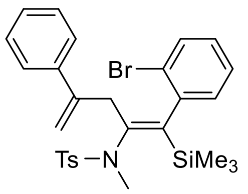

The product was obtained, after 6 h of reaction time and column chromatography purification (SiO_2, hexanes/ethyl acetate 5:1) in 72% yield (method B, 40 mg) as a crystalline white solid; m. p. 110–112 ºC; ^{1}H NMR (300 MHz, DMSO) δ = 7.73 (d, *J* = 7.9 Hz, 2H), 7.59 (d, *J* = 7.9 Hz, 1H), 7.39–7.23 (m, 3H), 7.21–6.97 (m, 5H), 6.78–6.62 (m, 2H), 3.67–3.43 (m, 2H), 3.00 (s, 3H), 2.97–2.63 (m, 2H), 2.33 (s, 3H), 0.16 (s, 9H); ^{13}C NMR (75 MHz, DMSO, 383K) δ =147.5 (C), 146.0 (C), 142.8 (C), 142.5 (C), 140.8 (C), 140.3 (C), 136.2 (C), 131.7 (CH), 129.2 (CH), 128.7 (CH), 127.2 (CH), 127.1 (CH), 126.8 (CH), 126.3 (CH), 126.1 (CH), 124.7 (CH), 121.6 (C), 114.2 (CH_2), 38.4 (CH_3), 37.2 (CH_2), 20.0 (CH_3), – 0.7 (CH_3); ^{29}Si NMR (75 MHz, $CDCl_3$) δ = – 4.8; HRMS (ESI) m/z: $[M + Na]^+$ Calcd for $C_{28}H_{32}BrNNaO_2SSi$ 576.0999; Found 576.1016.

Enamide 3v

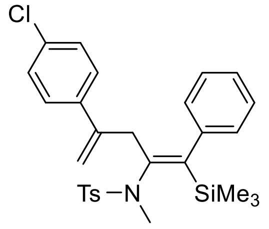

The product was obtained, after 4 h of reaction time and column chromatography purification (SiO_2, hexanes/ethyl acetate 5:1) in 65% (method A, 33 mg) or 61% (method B, 31 mg) as a crystalline white solid; m. p. 106–108 ºC; ^{1}H NMR (300 MHz, $CDCl_3$) δ = 7.75 (d, *J* = 7.9 Hz, 2H), 7.35–7.15 (m, 5H), 7.10 (d, *J* = 8.4 Hz, 2H), 7.05–6.93 (m, 2H), 6.70 (d, J = 8.5 Hz, 2H), 5.09 (s, 1H), 4.75 (s, 1H), 3.26–2.88 (m, 1H), 3.04 (s, 3H), 2.70–2.48 (m, 1H), 2.42 (s, 3H), 0.20 (s, 9H); ^{13}C NMR (75 MHz, $CDCl_3$) δ =151.0 (C), 144.2 (C), 143.8 (C), 143.5 (C), 141.8 (C), 139.6 (C), 137.0 (C), 133.2 (C), 129.7 (CH), 128.2 (CH), 128.0 (CH), 127.9 (CH), 127.8 (CH), 127.3 (CH), 126.1 (CH), 115.2 (CH_2), 39.0 (CH_3), 37.1 (CH_2), 21.6 (CH_3), – 0.1 (CH_3); ^{29}Si NMR (75 MHz, $CDCl_3$) δ = – 5.7; HRMS (ESI) m/z: $[M + H]^+$ Calcd for $C_{28}H_{33}ClNO_2SSi$ 510.1684; Found 510.1691.

Enamide 3w

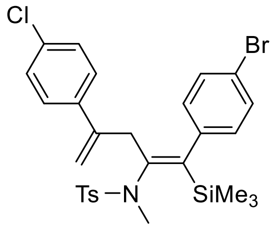

The product was obtained, after 4 h of reaction time and column chromatography purification (SiO_2, hexanes/ethyl acetate 5:1) in 42% (method B, 25 mg) as a crystalline white solid; m. p. 113–115 ºC; ^{1}H NMR (300 MHz, $CDCl_3$) δ = 7.73 (d, *J* = 8.2 Hz, 2H), 7.42 (d, *J* = 8.1 Hz, 2H), 7.27 (d, *J* = 8.0 Hz, 2H), 7.12 (d, *J* = 8.5 Hz, 2H), 6.95–6.81 (m, 2H), 6.70 (d, *J* = 8.6 Hz, 2H), 5.11 (s, 1H), 4.76 (s, 1H), 3.15–2.94 (m, 1H), 3.02 (s, 3H), 2.70–2.46 (m, 1H), 2.43 (s, 3H), 0.20 (s, 9H); ^{13}C NMR (75 MHz, $CDCl_3$) δ =149.9 (C), 144.0 (C), 143.9 (C), 143.5 (C), 140.6 (C), 139.2 (C), 136.6 (C), 133.3 (CH), 131.1 (CH), 129.6 (CH), 129.3 (CH), 128.1 (CH), 127.7 (CH), 127.1 (CH), 120.0 (C), 115.0 (CH_2), 38.7 (CH_3), 36.9 (CH_2),

21.5 (CH_3), – 0.2 (CH_3); HRMS (ESI) m/z: $[M + H]^+$ Calcd for $C_{28}H_{32}BrClNO_2SSi$ 588.0789; Found 588.0788.

Enamide 3x

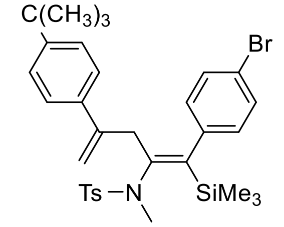

The product was obtained after 4 h of reaction time and column chromatography purification (SiO_2, hexanes/ethyl acetate 5:1) in 66% yield (Method B, 40 mg) as a yellow oil; ^1H NMR (300 MHz, $CDCl_3$) δ = 7.77 (d, *J* = 7.9 Hz, 2H), 7.40 (d, *J* = 8.0 Hz, 2H), 7.31 (d, *J* = 8.0 Hz, 2H), 7.18 (d, *J* = 8.0 Hz, 2H), 7.01-6.82 (m, 2H), 6.74 (d, *J* = 8.0 Hz, 2H), 5.18 (s, 1H), 4.81 (s, 1H), 3.11-2.79 (m, 1H), 3.02 (s, 3H), 2.62-2.36 (m, 1H), 2.45 (s, 3H), 1.30 (s, 9H), 0.20 (s, 9H); ^{13}C NMR (75 MHz, $CDCl_3$) δ =150.6 (C), 149.8 (C), 144.6 (C), 143.8 (C), 143.4 (C), 140.7 (C), 137.7 (C), 136.5 (C), 131.0 (CH), 129.7 (CH), 129.3 (CH), 127.8 (CH), 125.0 (CH), 124.9 (CH), 119.9 (C), 113.5 (CH_2), 38.1 (CH_3), 36.6 (CH_2), 34.4 (C), 31.2 (CH_3), 21.5 (CH_3), - 0.2 (CH_3); ^{29}Si NMR (75 MHz, $CDCl_3$) δ = – 5.6; HRMS (ESI) m/z: $[M + H]^+$ Calcd for $C_{32}H_{41}BrNO_2SSi$ 610.1805; Found 610.1822.

Enamine 3y

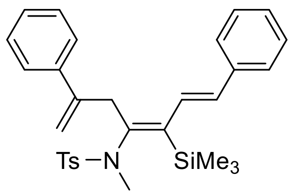

The product was obtained, after 5 h of reaction time and column chromatography purification (SiO_2, hexanes/ethyl acetate 5:1) in 51% yield (method B, 26 mg) as a white gum. ^1H NMR (300 MHz, $CDCl_3$) δ = 7.75 (d, *J* = 8.0 Hz, 2H), 7.36–7.18 (m, 10H), 7.10–6.99 (m, 2H), 6.79 (d, *J* = 16.6 Hz, 1H), 6.56 (d, *J* = 16.6 Hz, 1H), 5.35 (d, *J* = 1.6 Hz, 1H), 4.96 (d, *J* = 1.6 Hz, 1H), 3.56–3.39 (m, 1H), 2.95 (s, 3H), 2.54–2.34 (m, 1H), 2.45 (s, 3H), 0.40 (s, 9H); ^{13}C NMR (75 MHz, $CDCl_3$) δ =146.1 (C), 143.9 (C), 143.4 (C), 142.9 (C), 140.9 (C), 137.6 (C), 136.3 (C), 131.8 (CH), 129.6 (CH), 129.2 (CH), 128.6 (CH), 128.2 (CH), 127.8 (CH), 127.6 (CH), 127.4 (CH), 126.2 (CH), 125.8 (CH), 113.6 (CH_2), 37.0 (CH_3), 35.7 (CH_2), 21.5 (CH_3), – 0.9 (CH_3); HRMS (ESI) m/z: $[M + H]^+$ Calcd for $C_{30}H_{36}NO_2SSi$ 502.2231; Found 502.2239.

Preparation of desilylated enamide 4. Silylenamide **3o** (24 mg, 0.05 mmol, 1 equiv) was solved in 0.5 mL of THF (0.1 M) under inert atmosphere and molecular sieves. Then, a 1 M solution of TBAF in THF (0.1 mL, 0.1 mmol, 2 equiv) was added dropwise until colour change from colourless to red. Then, the mixture was stirred overnight at room temperature. Then, the reaction was quenched with water and extracted with ethyl ether (3 x 10 mL). After solvent removal the crude was purified by column chromatography (SiO_2, hexanes/ethyl acetate 5:1) to get 17 mg (86%) of desilylated enamide **4**.

Enamide 4

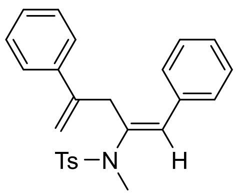

Yellow oil; ^{1}H NMR (300 MHz, $CDCl_3$) δ = 7.74 (d, *J* = 8.0 Hz, 2H), 7.42–7.10 (m, 12H), 5.60 (s, 1H), 5.41 (s, 1H), 5.29 (s 1H), 3.86 (s, 2H), 2.82 (s, 3H), 2.46 (s, 3H); ^{13}C NMR (75 MHz, $CDCl_3$) δ =144.7 (C), 143.5 (C), 141.6 (C), 140.7 (C), 135.3 (C), 133.4 (C), 130.2 (CH), 129.3 (CH), 128.5 (CH), 128.4 (CH), 128.2 (CH), 128.1 (CH), 127.6 (CH), 127.5 (CH), 126.3 (CH), 114.9 (CH_2), 39.2 (CH_3), 39.0 (CH_2), 21.6 (CH_3); HRMS (ESI) m/z: $[M + Na]^+$ Calcd for $C_{25}H_{25}NNaO_2S$ 426.1504; Found 426.1511.

Preparation of coupling product 5. In a round bottomed flask under inert atmosphere and molecular sieves silylated enamide **3o** (67 mg, 0.14 mmol, 1 equiv) and 4-bromobenzaldehyde (130 mg, 0.7 mmol, 3 equiv) were dissolved in 0.8 mL of THF (0.17 M). The solution was cooled to – 20 ºC and then 0.15 mL of 1M THF solution of TBAF (0.15 mmol, 1,1 equiv) were added dropwise. The resulting mixture was allowed to warm to room temperature and stirred overnight, then quenched with water and extracted with ethyl ether (3 x 10 mL). After solvent removal, the resulting crude was purified by column chromatography (SiO_2, hexanes/ethyl acetate, 5 :1) to get 59 mg (72%) of coupling product **5**.

Compound 5

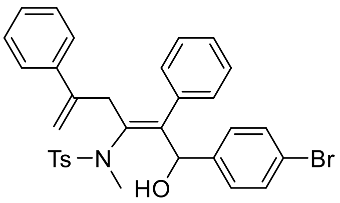

Yellow gum; ^{1}H NMR (300 MHz, $CDCl_3$, 338K) δ = 7.90–7.76 (m, 2H), 7.43–7.09 (m, 11H), 7.03–6.71 (m, 5H), 6.18 (s, 1H), 5.16 (s, 1H), 4.82 (s, 1H), 3.16–3.07 (m, 1H), 3.11 (s, 3H), 2.77–2.63 (m, 1H) 2.44 (s, 3H); ^{13}C NMR (75 MHz, $CDCl_3$) δ =148.5 (C), 144.7 (C), 144.1 (C), 140.2 (C), 139.8 (C), 136.1 (C), 130.9 (CH), 130.0 (CH), 129.6 (CH), 128.2 (CH), 128.1 (CH), 127.7 (CH), 127.6 (CH), 127.5 (CH), 127.4 (CH), 125.9 (CH), 120.8 (C), 114.7 (CH_2), 72.1 (CH), 37.7 (CH_3), 36.5 (CH_2), 21.6 (CH_3); HRMS (ESI) m/z: $[M + Na]^+$ Calcd for $C_{32}H_{30}BrNNaO_3S$ 610.1022; Found 610.1041.

Preparation of cyclization product 6. Silylenamide **3o** (48 mg, 0.1 mmol, 1 equiv) dissolved in 0.15 mL of dichloroethane (0.7 M) was added, under inert atmosphere, to a 0,1 M solution of $HNTf_2$ (0.1 mL, 0.01 mmol, 1 mol%) in dichloroethane. The mixture was stirred at rt for 1 h and 5 mg (36 mmol) of K_2CO_3 were added. The solvent was removed, and the crude was purified by column chromatography (SiO_2, hexanes/ethyl acetate, 5:1) to get 31 mg (65%) of product **6**.

Compound 6

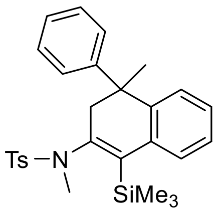

Crystalline white solid; m. p. 88–89 ºC; ^{1}H NMR (300 MHz, $CDCl_3$) δ = 7.68 (d, *J* = 7.9 Hz, 2H), 7.59–7.47 (m, 1H), 7.45–7.06 (m, 8H), 6.94–6.73 (m, 2H), 2.47 (s, 3H), 2.42 (s,

3H), 2.22 (d, *J* = 16.0 Hz, 1H), 1.96 (d, *J* = 16.0 Hz, 1H), 1.62 (s, 3H), 0.30 (s, 9H); ^{13}C NMR (75 MHz, $CDCl_3$) δ =146.1 (C), 144.2 (C), 143.4 (C), 140.7 (C), 140.2 (C), 138.6 (C), 136.4 (C), 129.6 (CH), 128.6 (CH), 127.7 (CH), 127.6 (CH), 127.4 (CH), 126.8 (CH), 126.3 (CH), 125.2 (CH), 42.1 (C), 40.8 (CH_2), 35.9 (CH_3), 27.7 (CH_3), 21.6 (CH_3), –1.34 (CH_3); ^{29}Si NMR (75 MHz, $CDCl_3$) δ = – 5.7; HRMS (ESI) m/z: [M + Na]$^+$ Calcd for $C_{28}H_{33}NNaO_2SSi$ 498.1893; Found 498.1901.

3.- NMR Spectra

^{1}H NMR (300 MHz, $CDCl_3$) spectrum of 1a

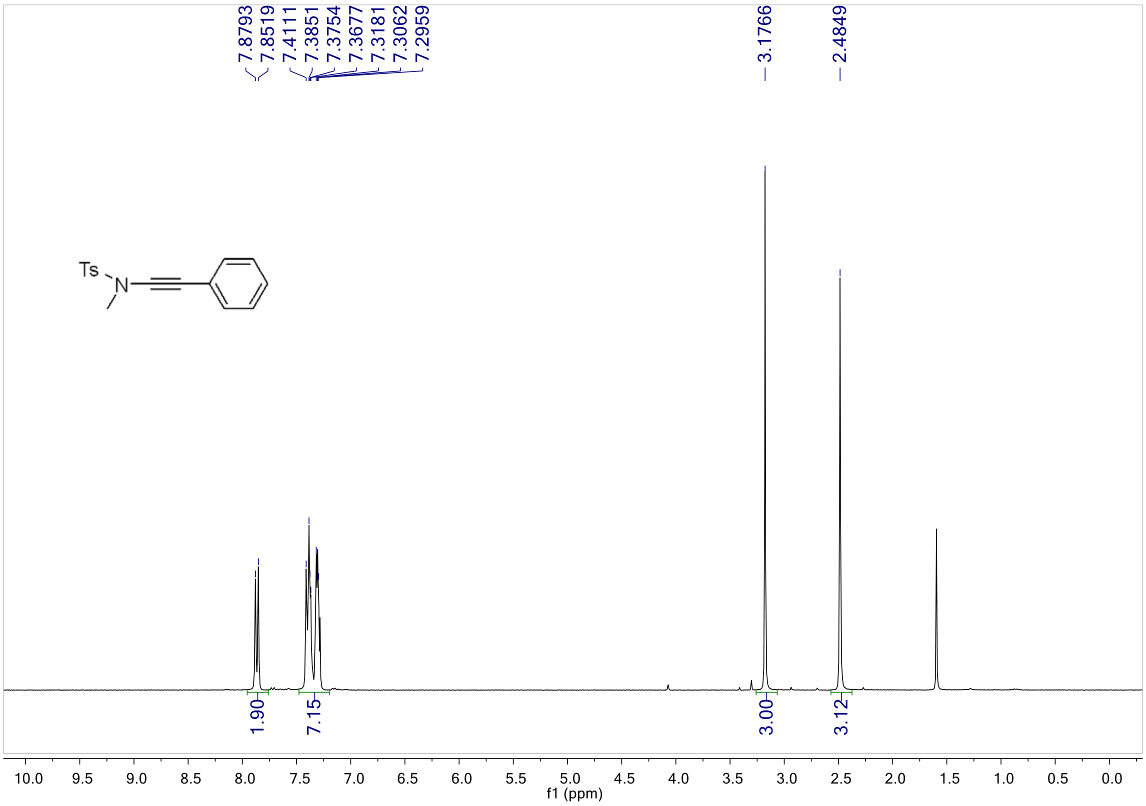

^{1}H NMR (300 MHz, $CDCl_3$) spectrum of 1b

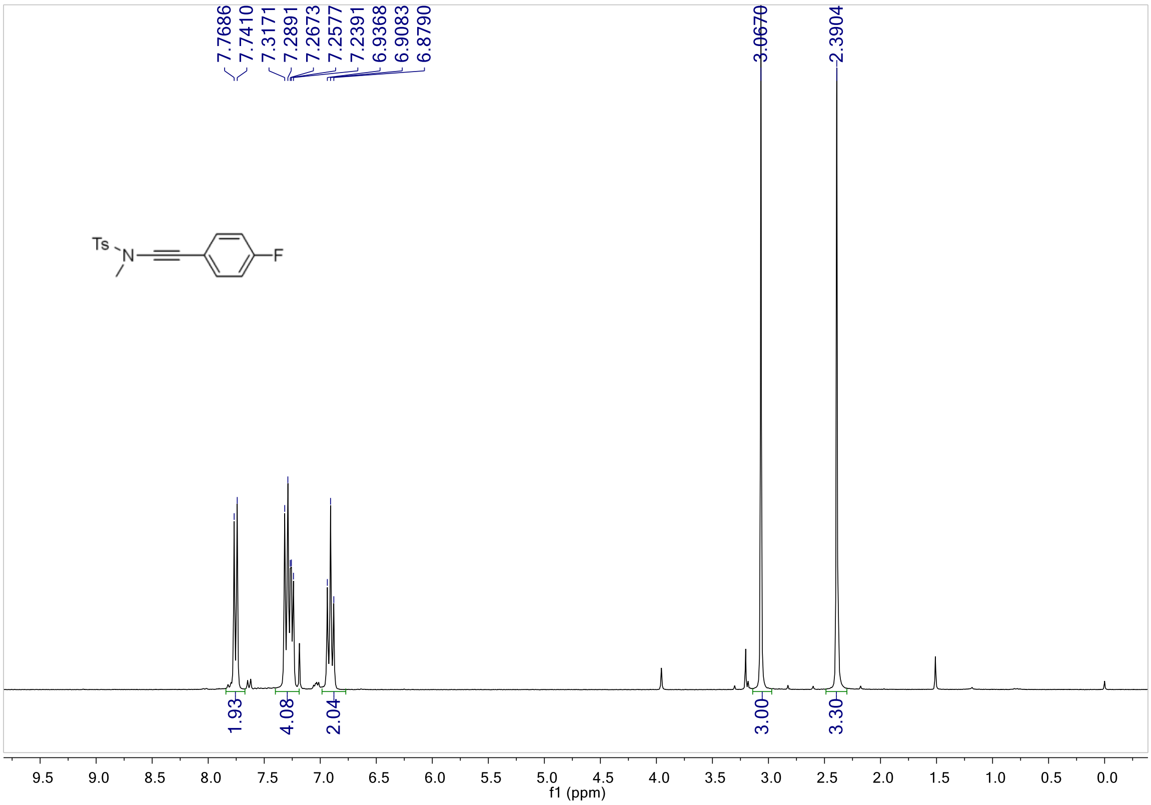

^{1}H NMR (300 MHz, $CDCl_3$) spectrum of 1c

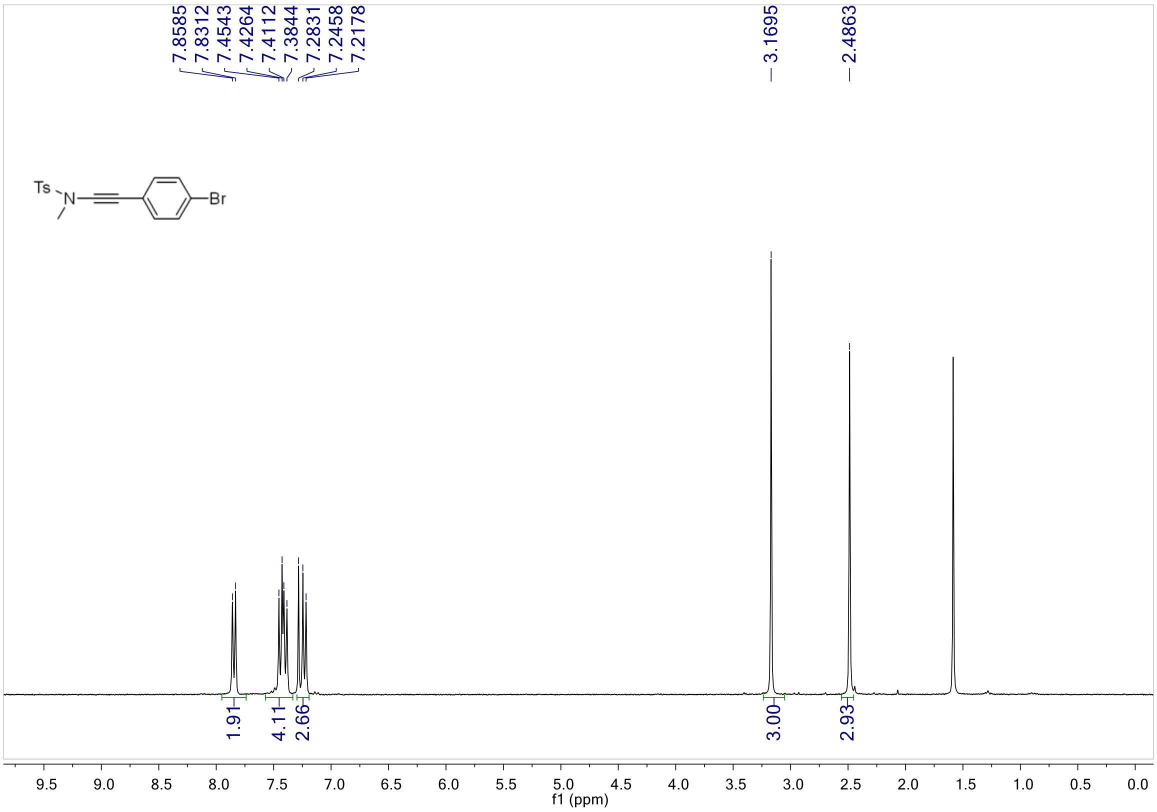

^{1}H NMR (300 MHz, $CDCl_3$) spectrum of 1d

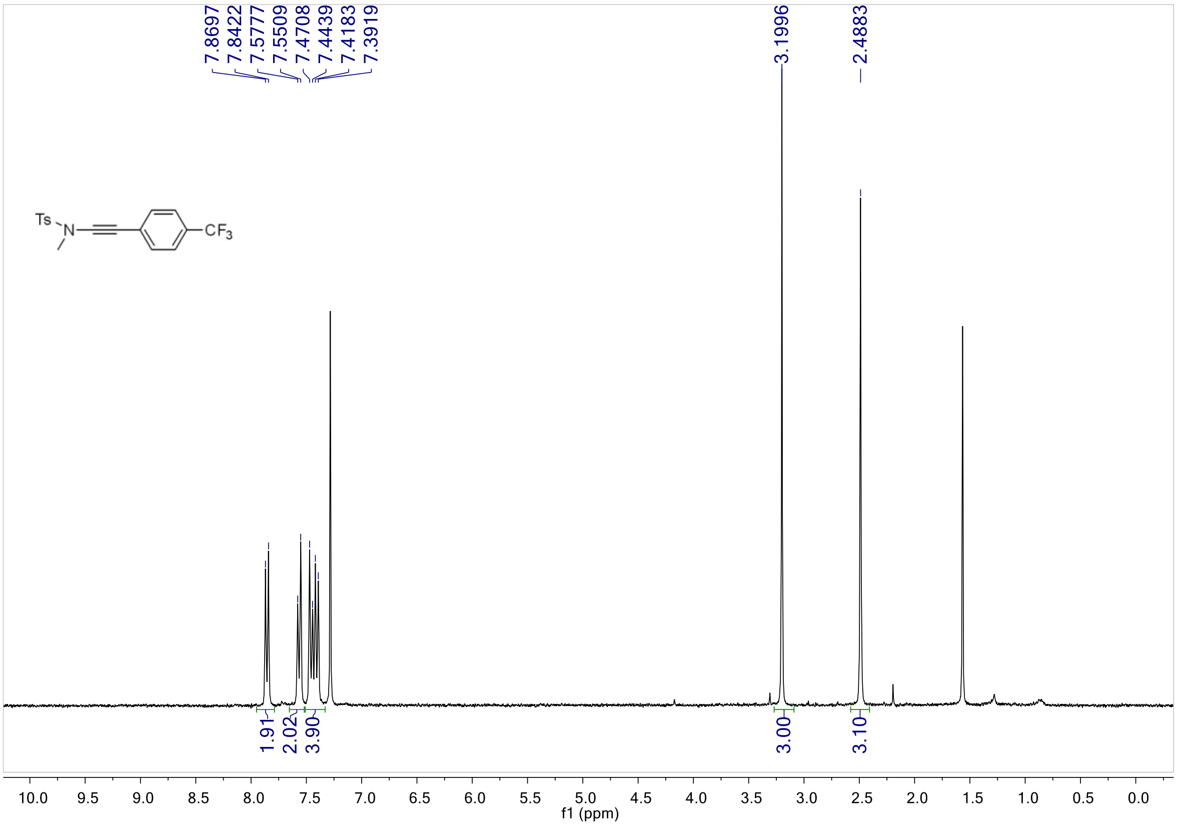

^{1}H NMR (300 MHz, $CDCl_3$) spectrum of 1e

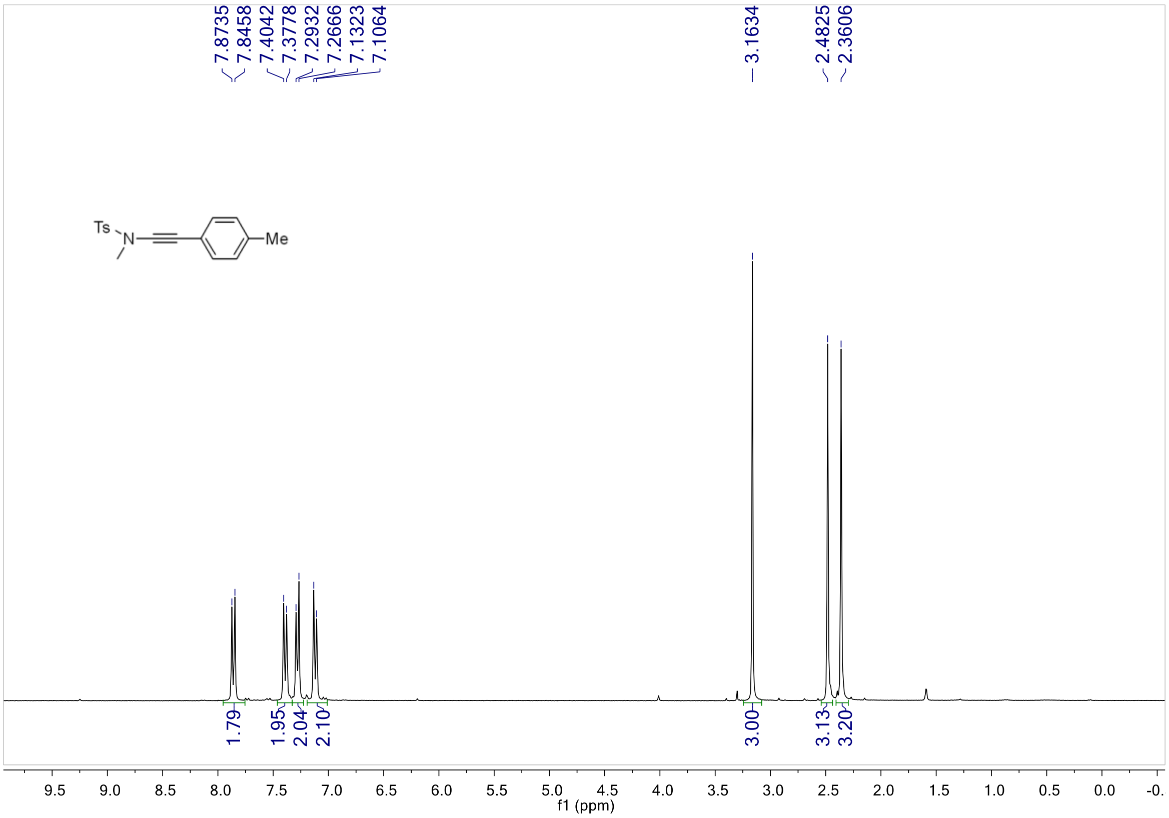

^{1}H NMR (300 MHz, $CDCl_3$) spectrum of 1f

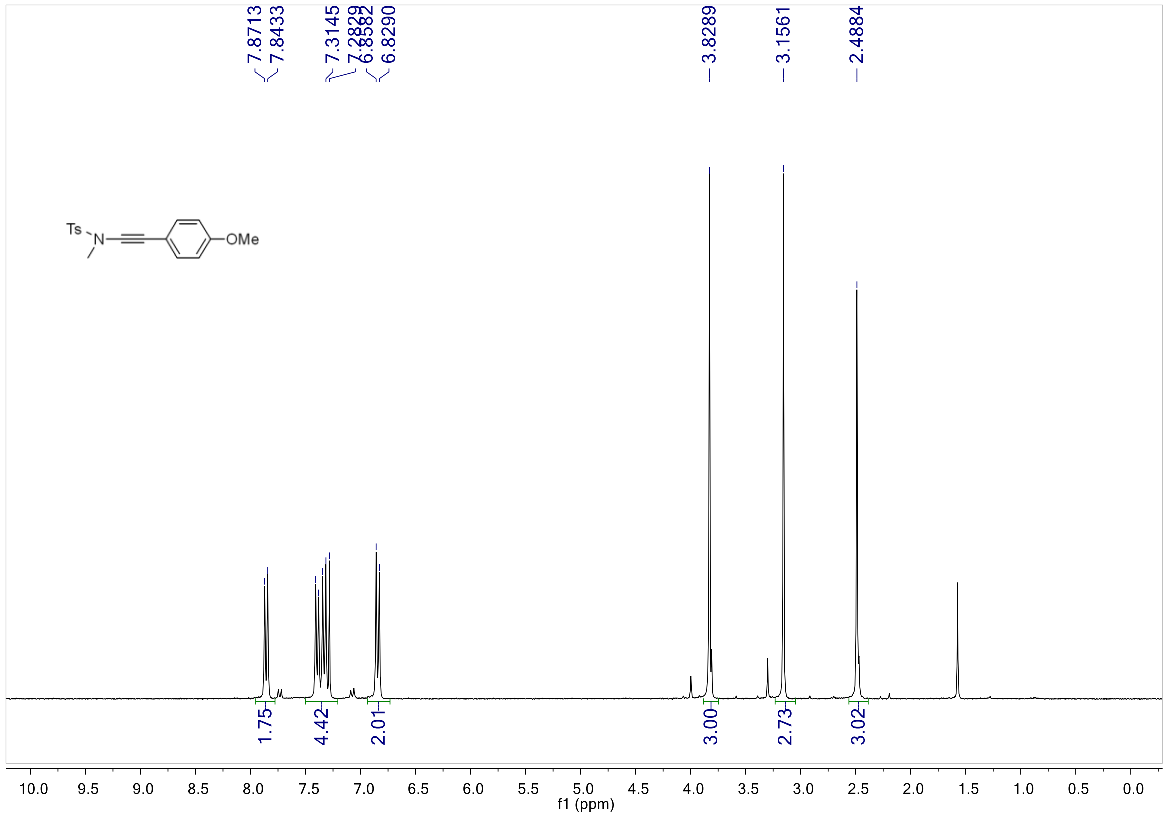

^{1}H NMR (300 MHz, $CDCl_3$) spectrum of 1g

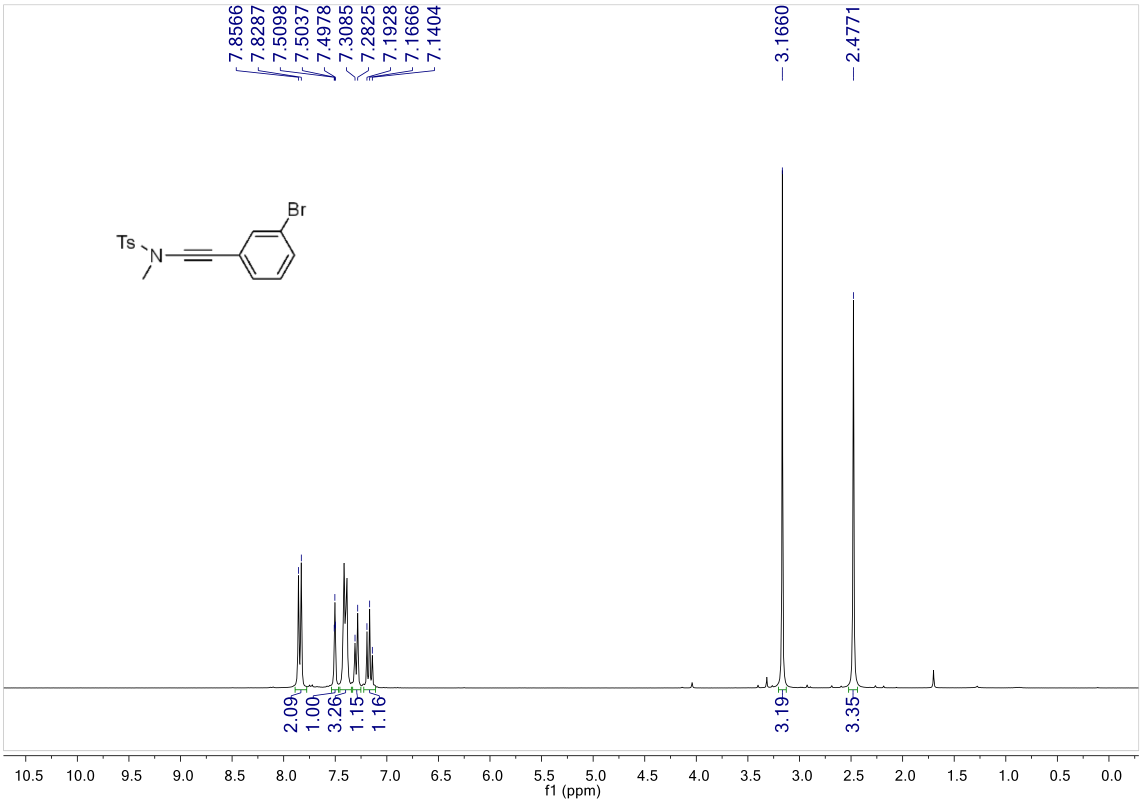

^{13}C NMR (75 MHz, $CDCl_3$) spectrum of 1g

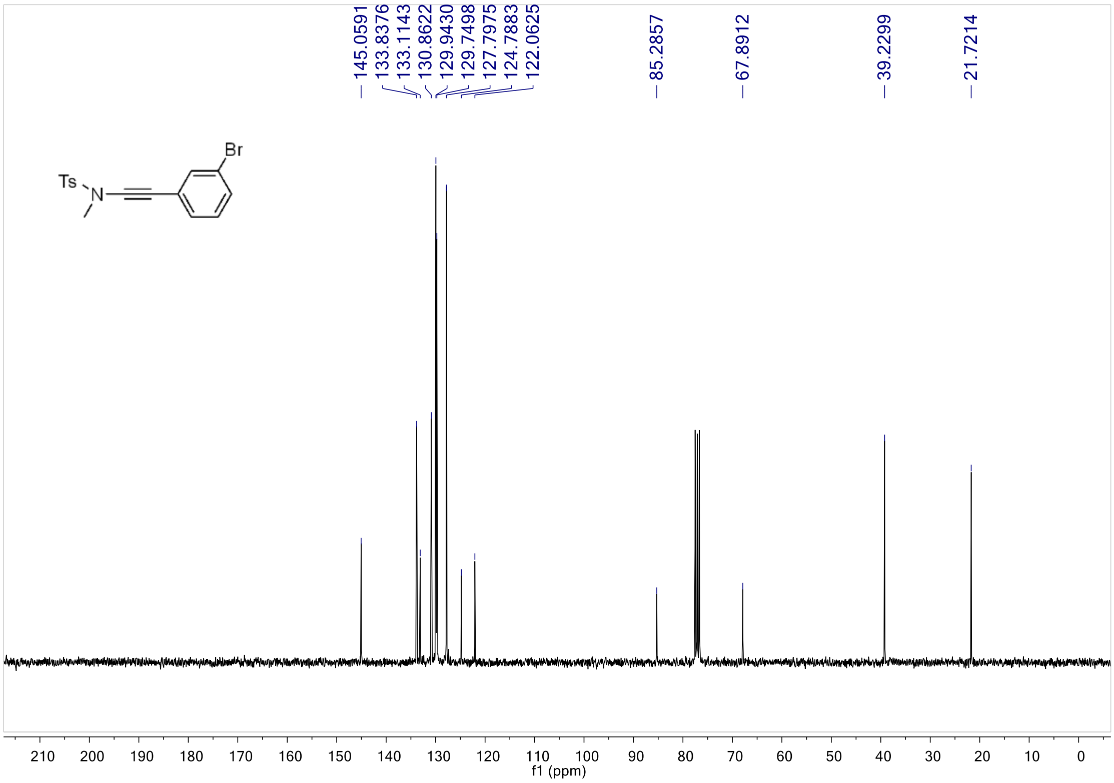

^{1}H NMR (300 MHz, $CDCl_3$) spectrum of 1h

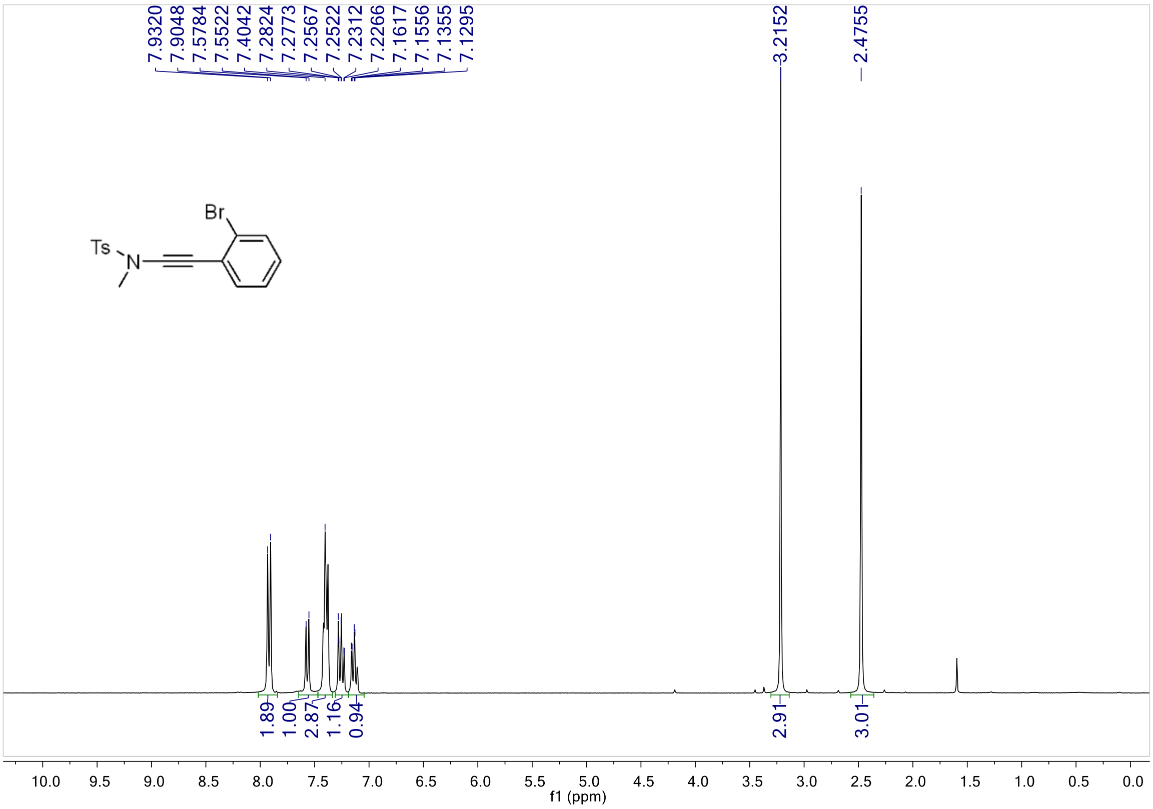

^{1}H NMR (300 MHz, $CDCl_3$) spectrum of 1i

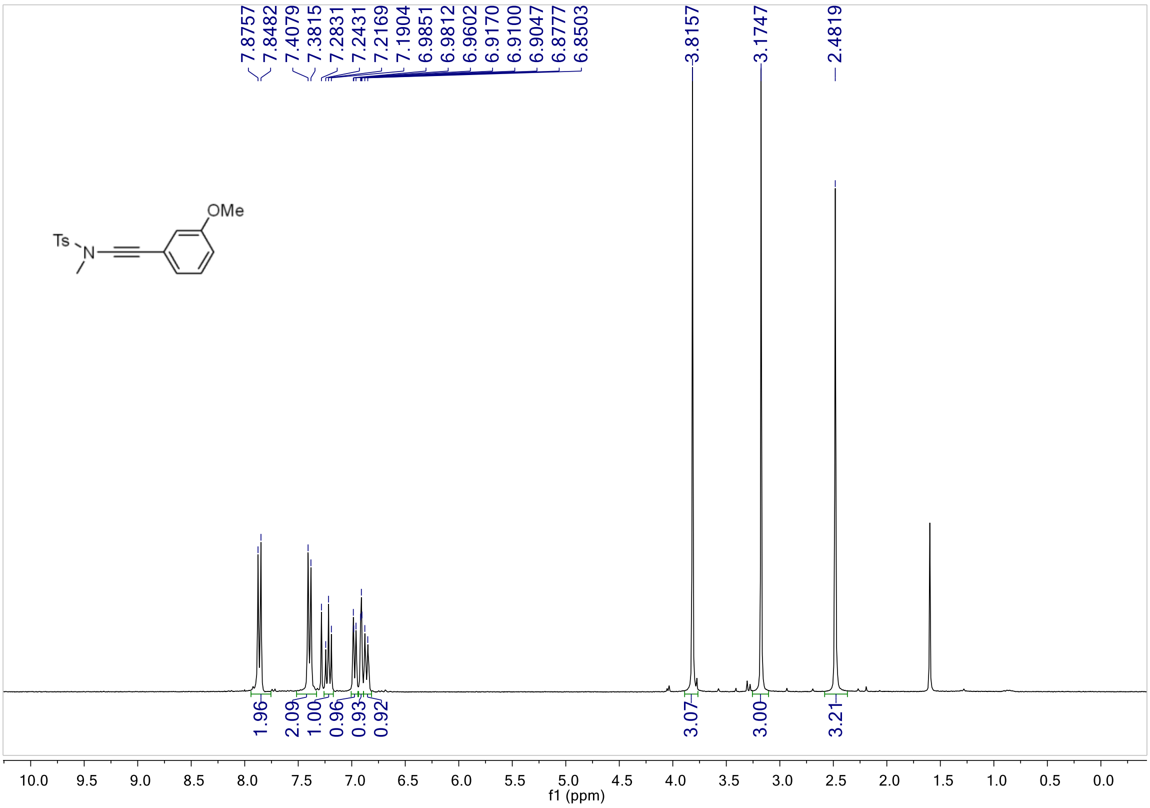

^{1}H NMR (300 MHz, CDCl3) spectrum of 1j

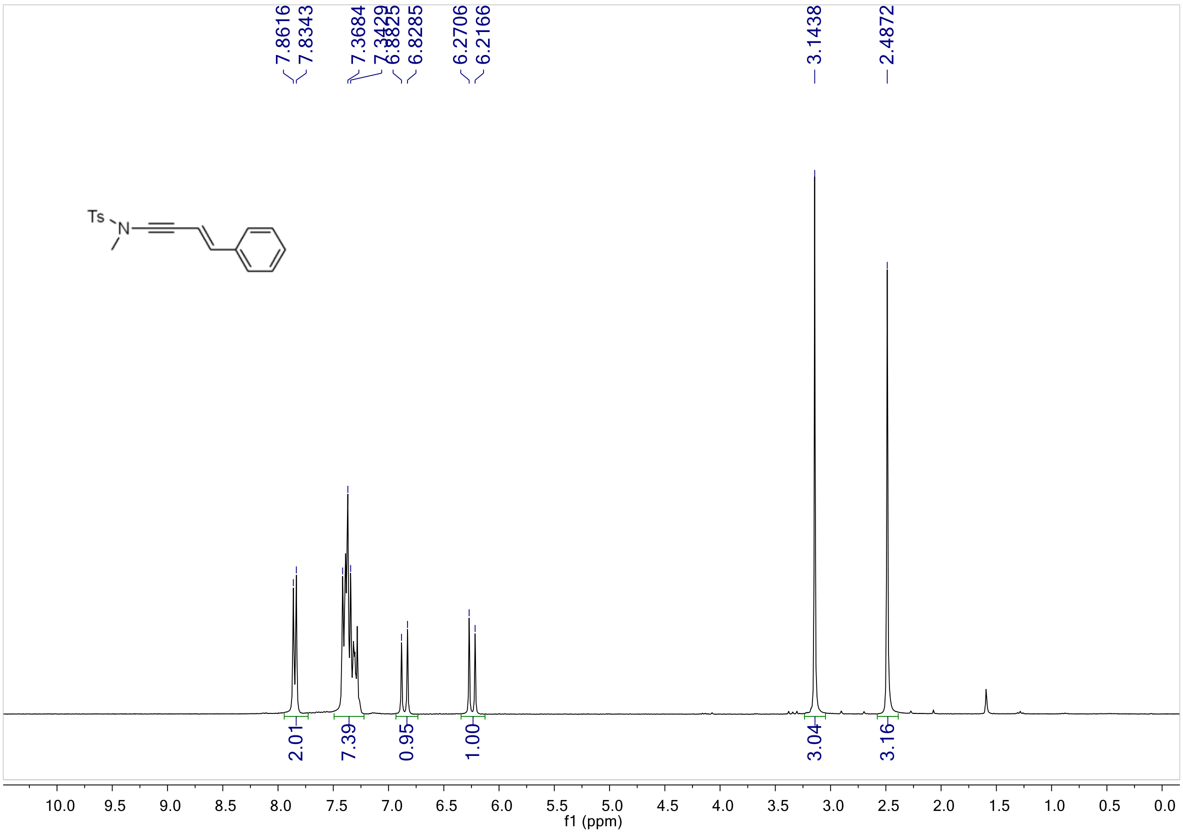

^{1}H NMR (300 MHz, CDCl3) spectrum of 1k

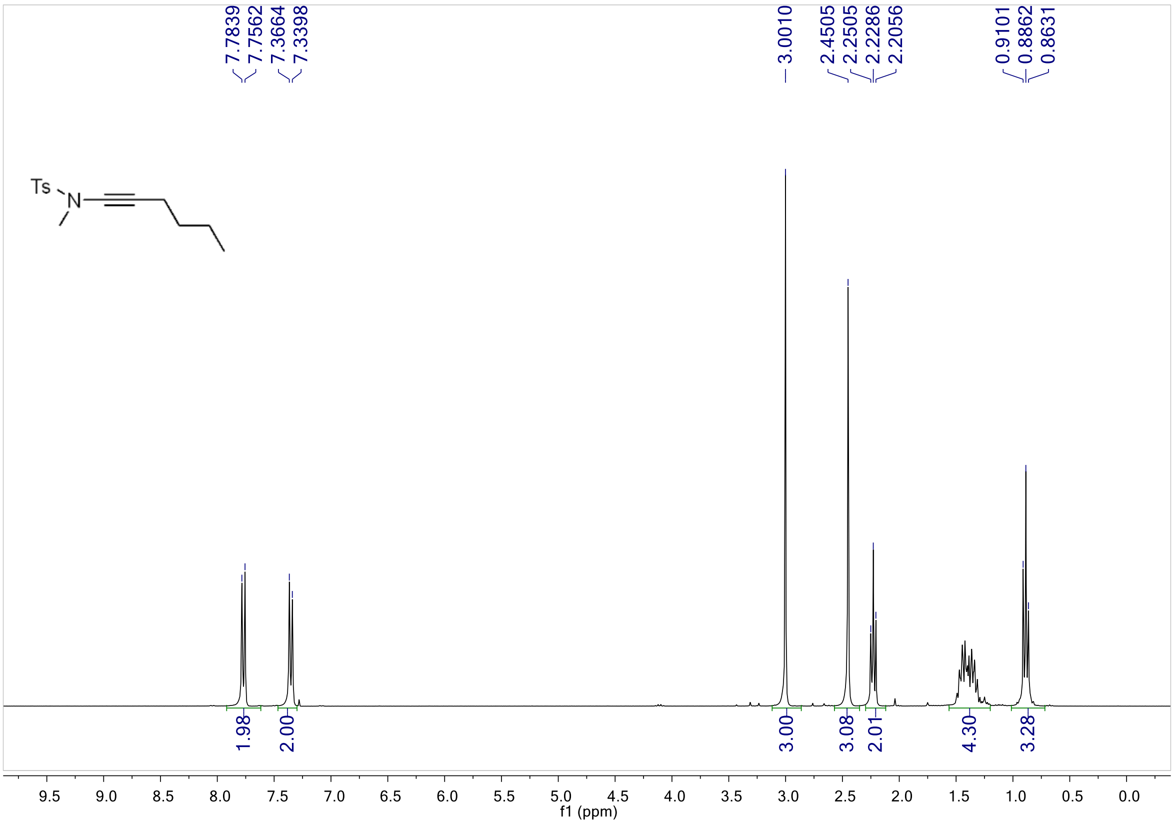

^{1}H NMR (300 MHz, $CDCl_3$) spectrum of 2c

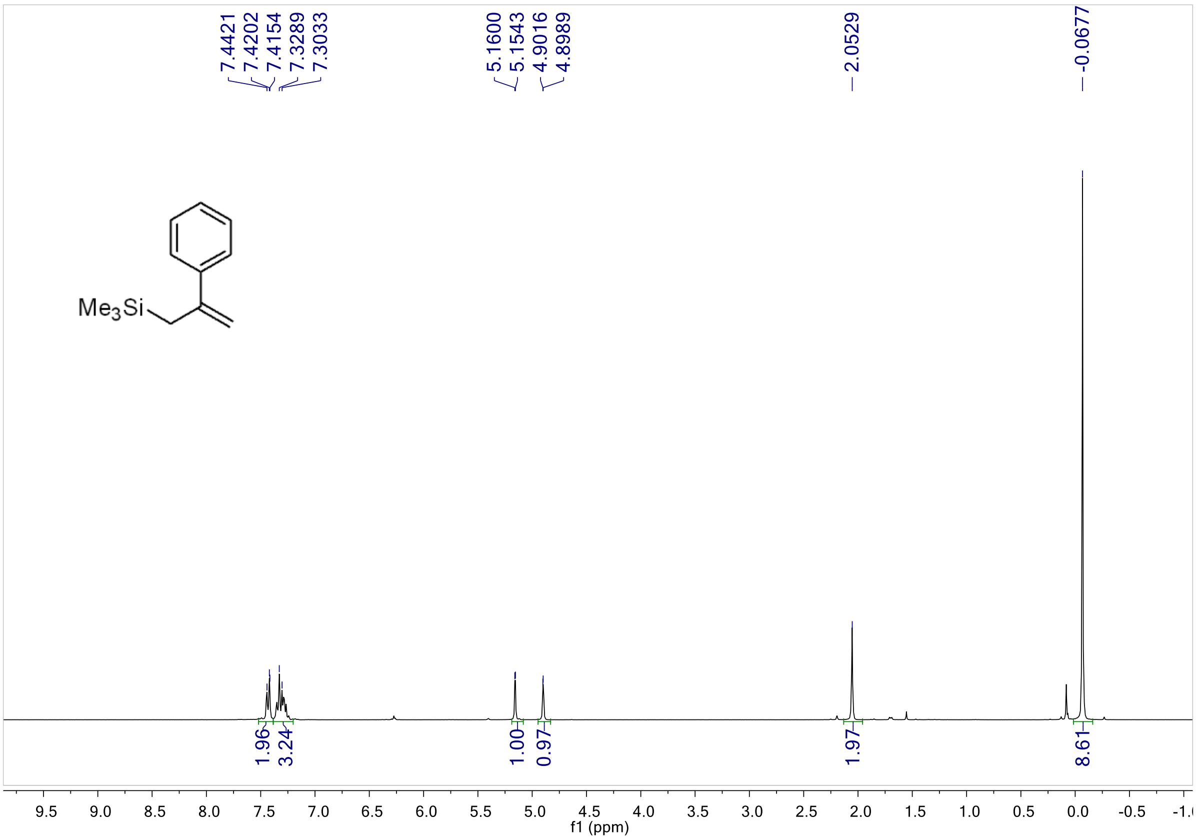

^{13}C NMR (75 MHz, $CDCl_3$) spectrum of 2c

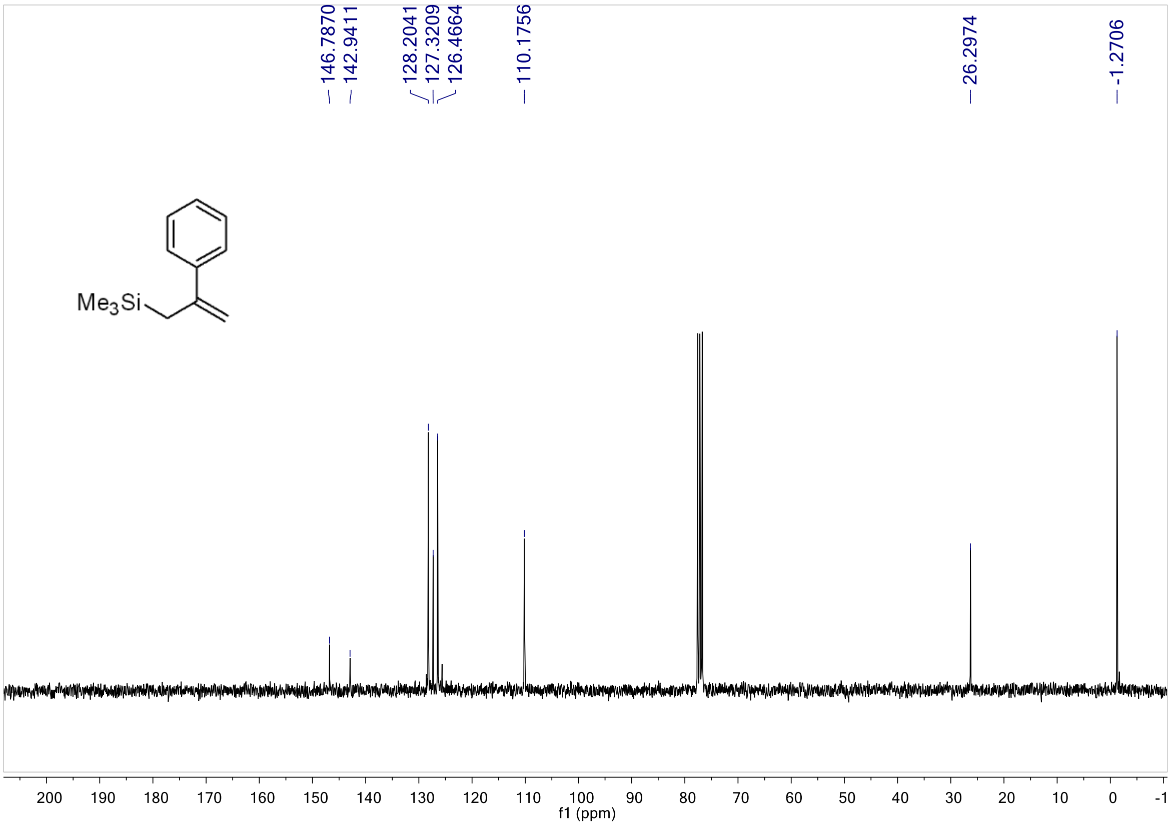

^{1}H NMR (300 MHz, $CDCl_3$) spectrum of 2d

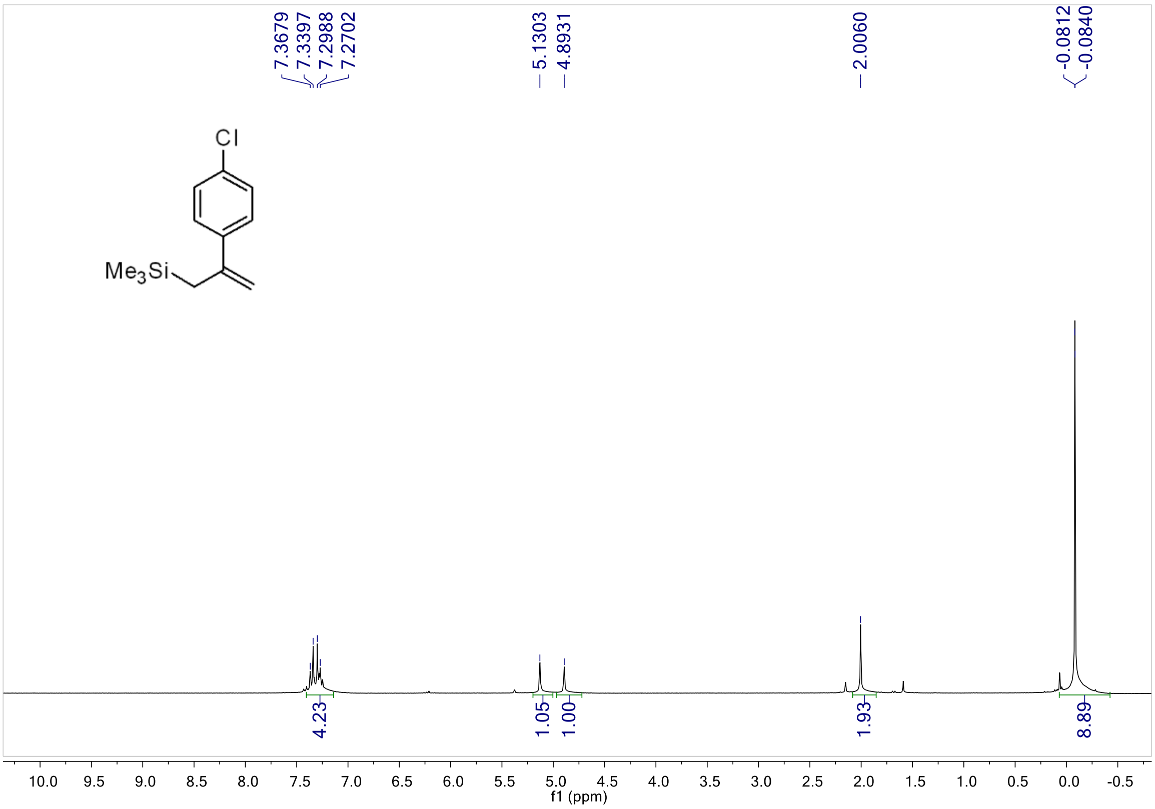

^{13}C NMR (75 MHz, $CDCl_3$) spectrum of 2d

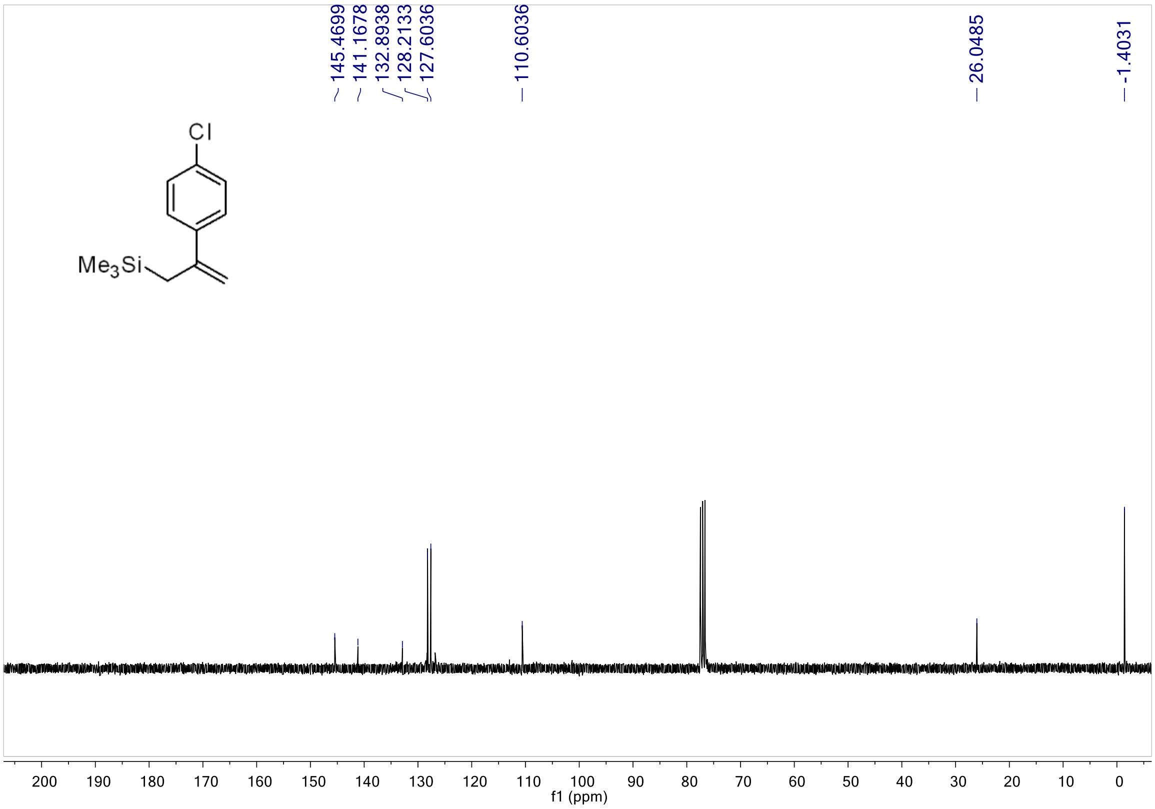

^{1}H NMR (300 MHz, $CDCl_3$) spectrum of 2e

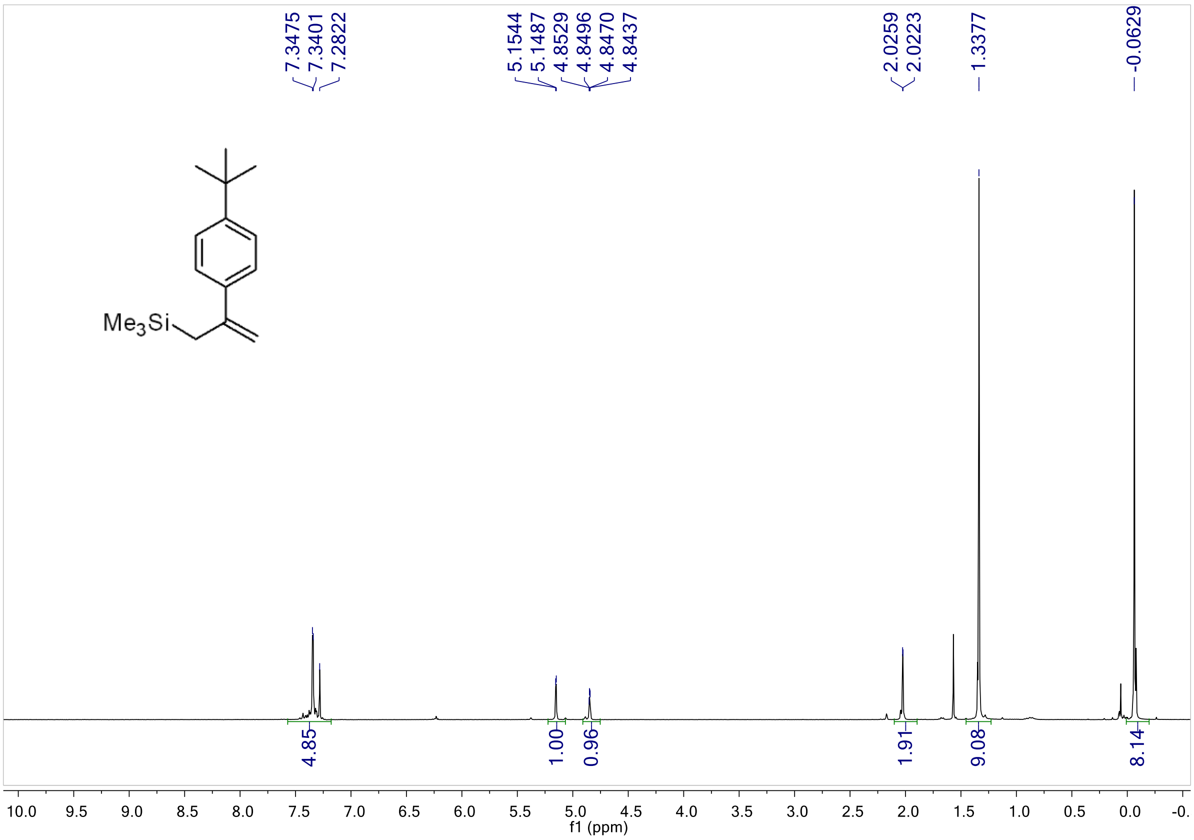

^{13}C NMR (75 MHz, $CDCl_3$) spectrum of 2e

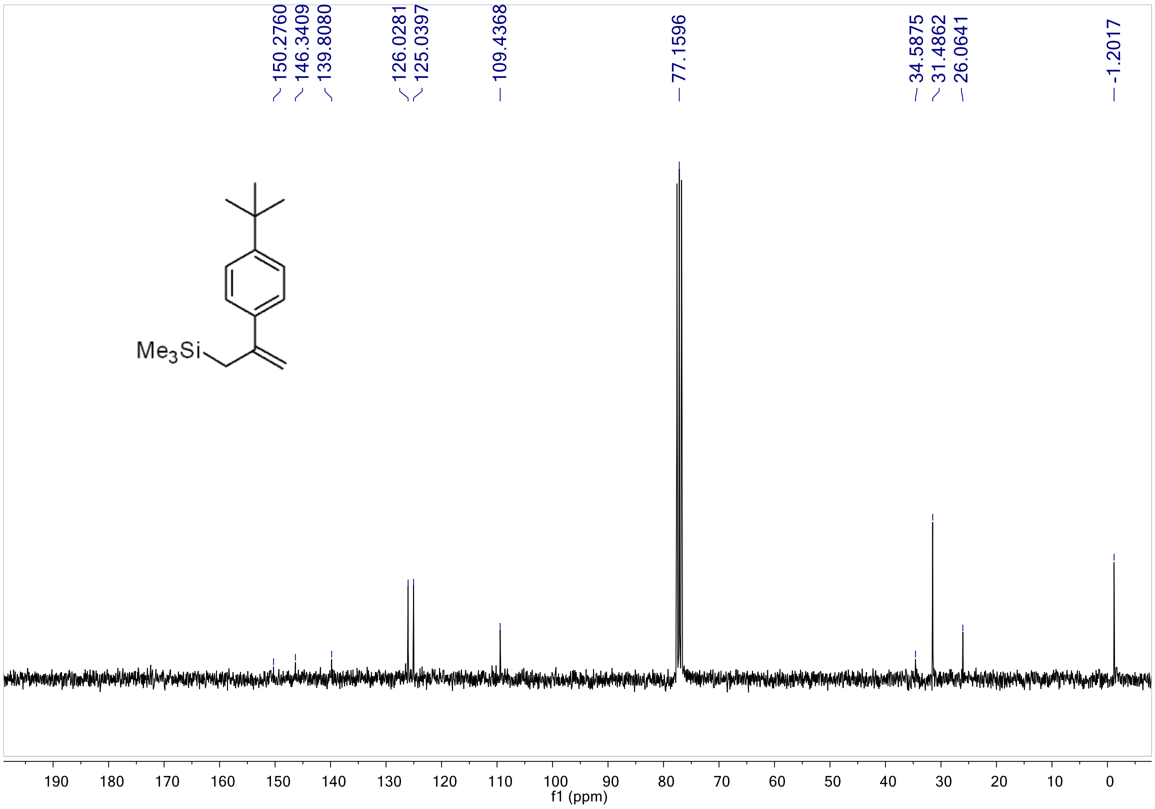

^{1}H NMR (300 MHz, $CDCl_3$) spectrum of 3a

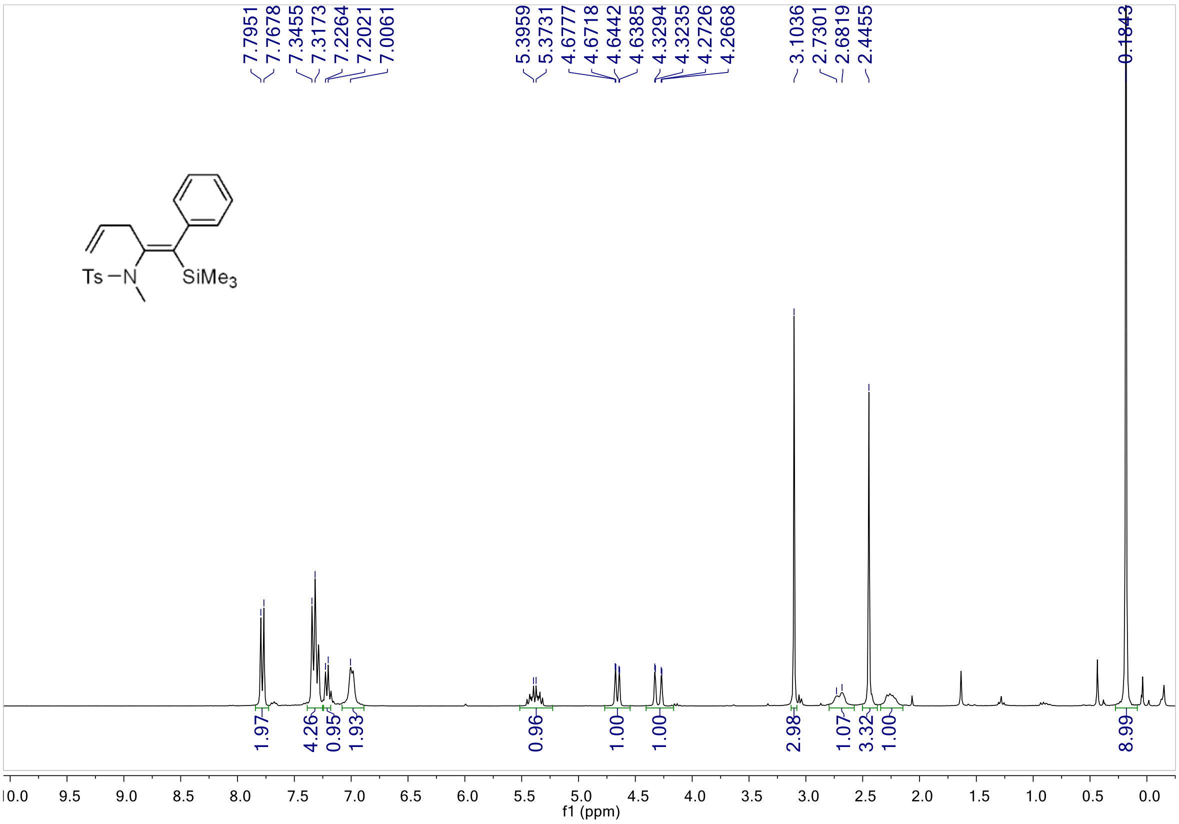

^{13}C NMR (75 MHz, $CDCl_3$) spectrum of 3a

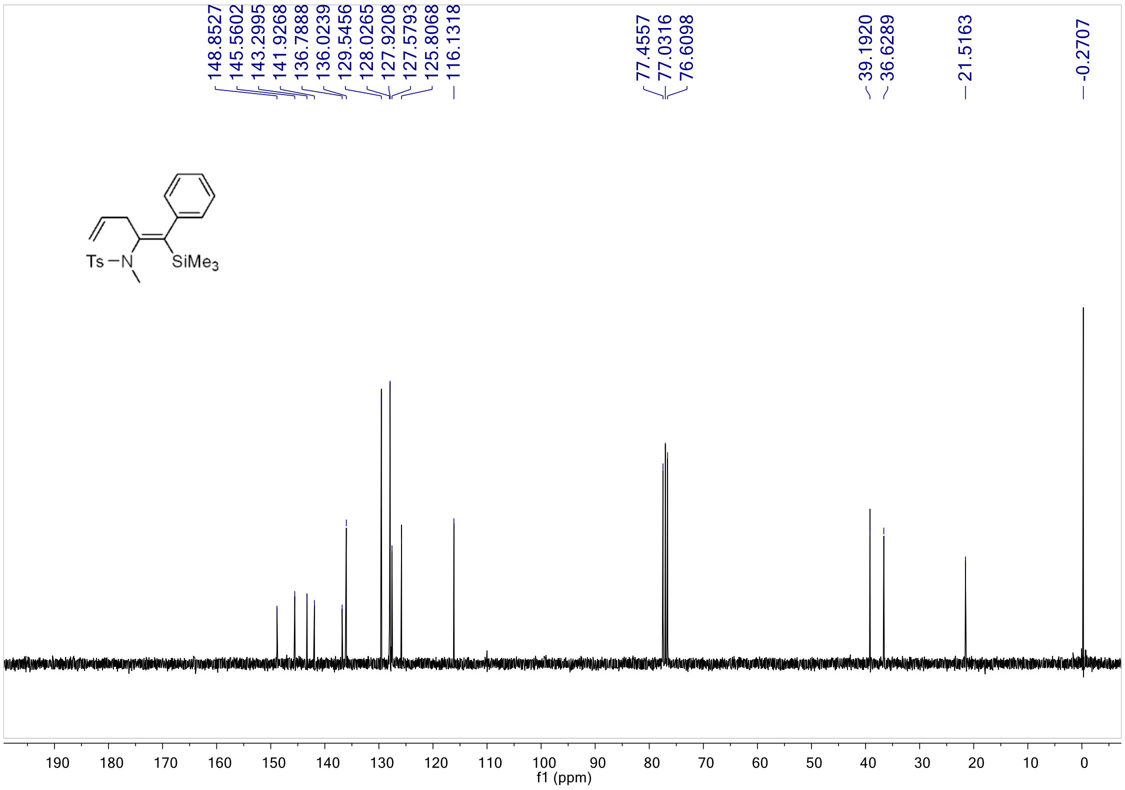

^{29}Si NMR (75 MHz, CDCl$_3$) spectrum of 3a

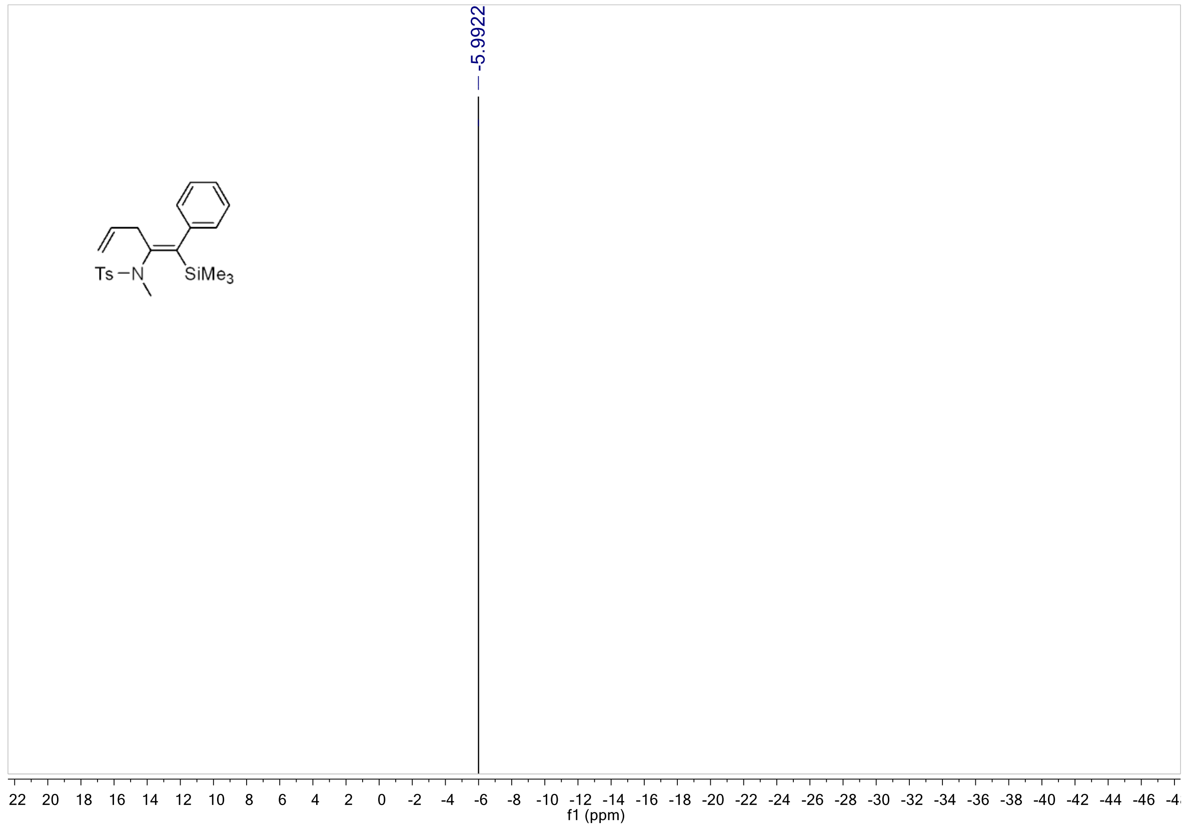

^{1}H NMR (300 MHz, CDCl$_3$) spectrum of 3b

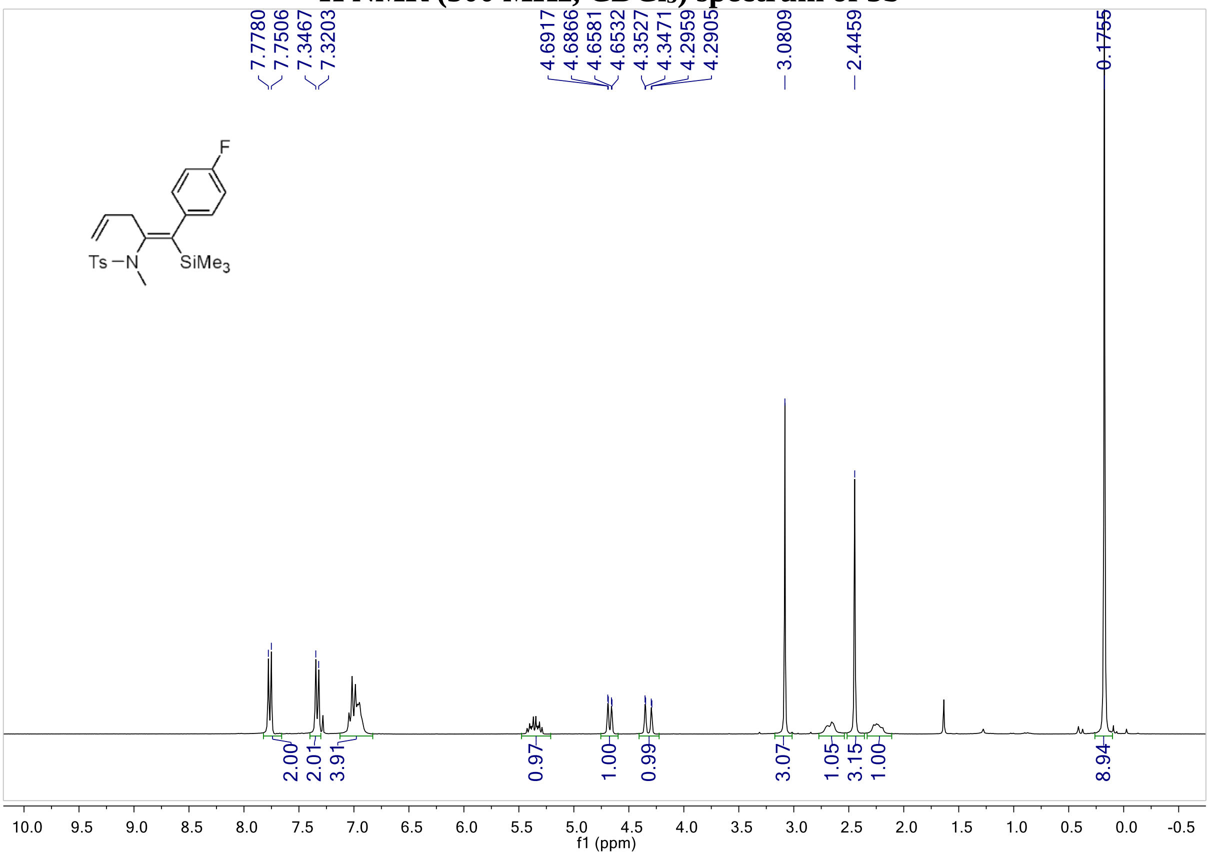

^{13}C NMR (75 MHz, CDCl$_3$) spectrum of 3b

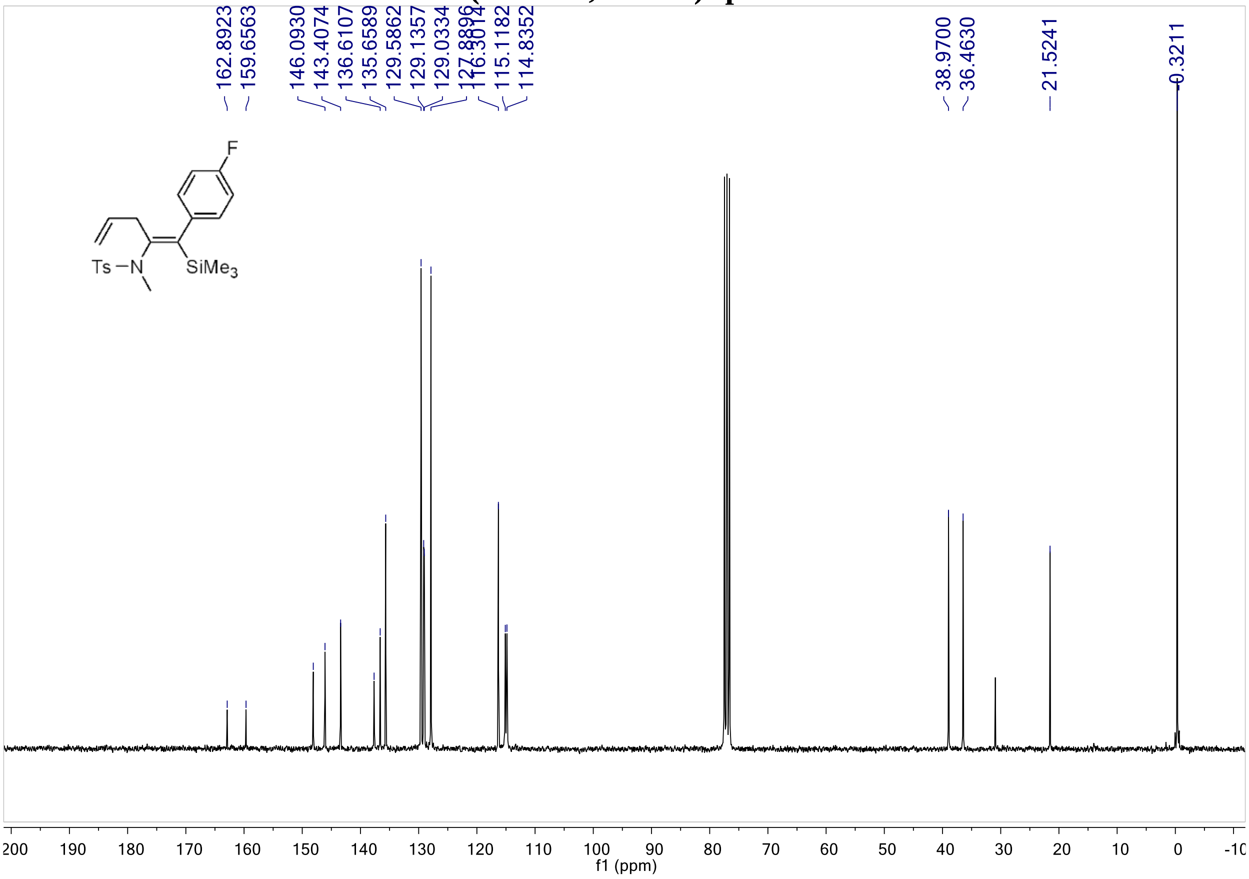

^{29}Si NMR (75 MHz, CDCl$_3$) spectrum of 3b

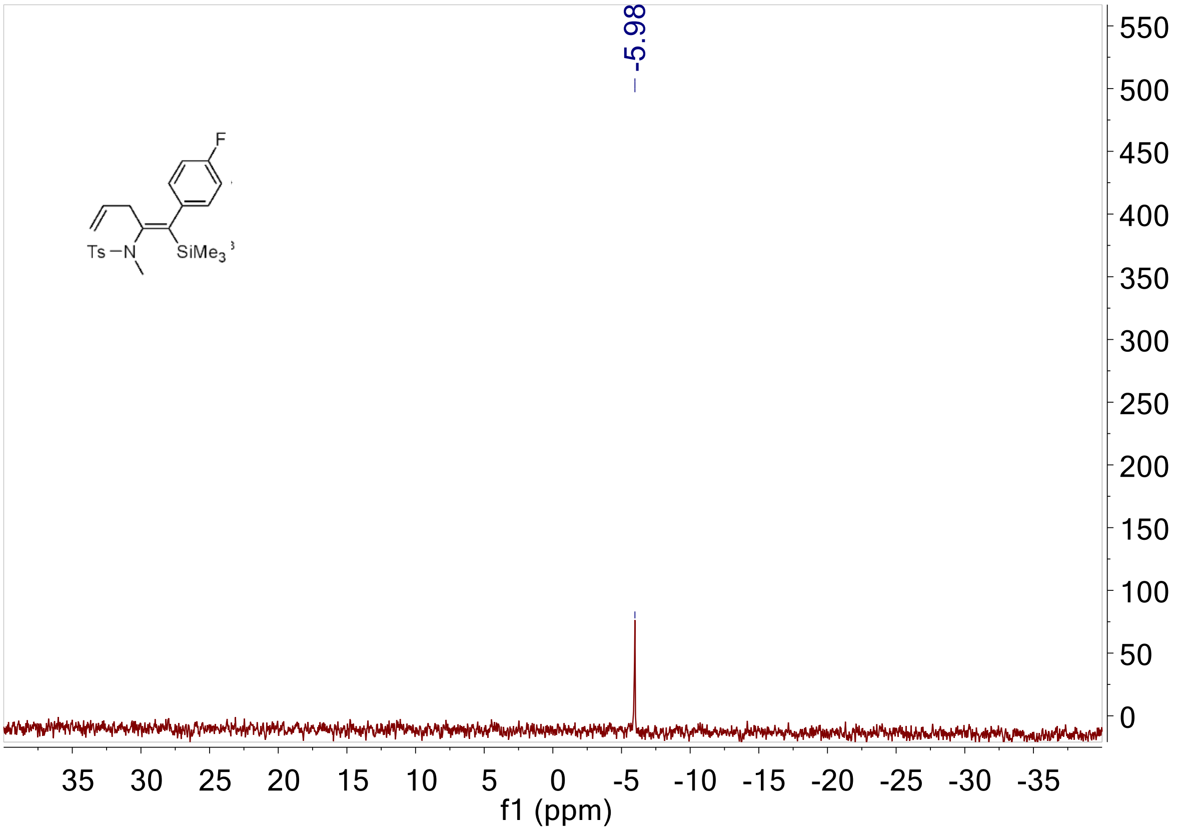

^{19}F NMR (282 MHz, $CDCl_3$) spectrum of 3b

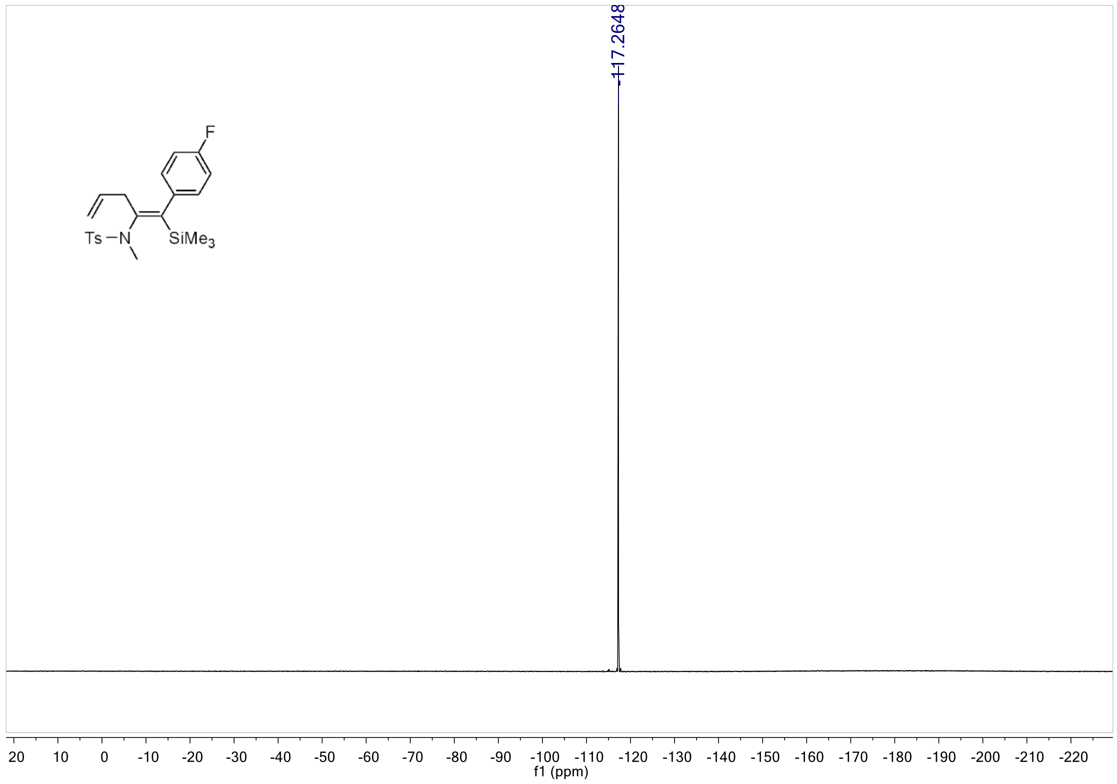

^{1}H NMR (300 MHz, $CDCl_3$) spectrum of 3c

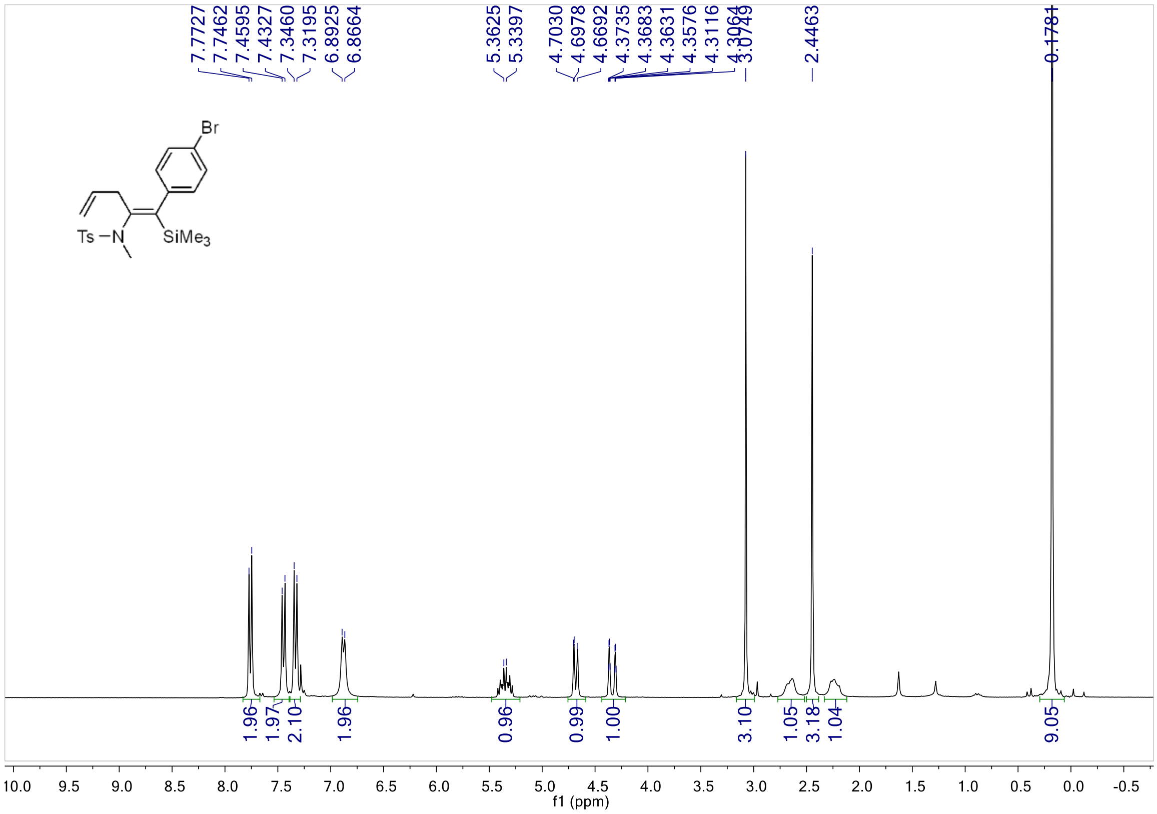

^{13}C NMR (75 MHz, CDCl$_3$) spectrum of 3c

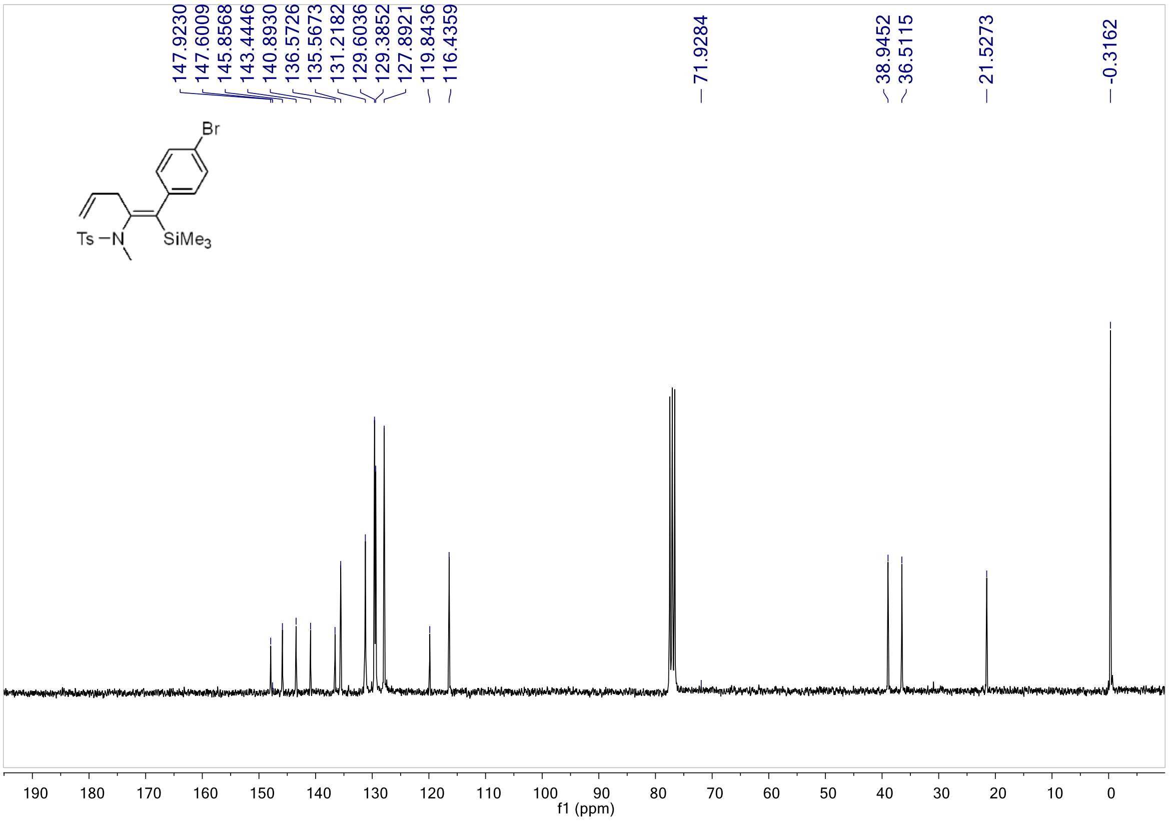

^{29}Si NMR (75 MHz, CDCl$_3$) spectrum of 3c

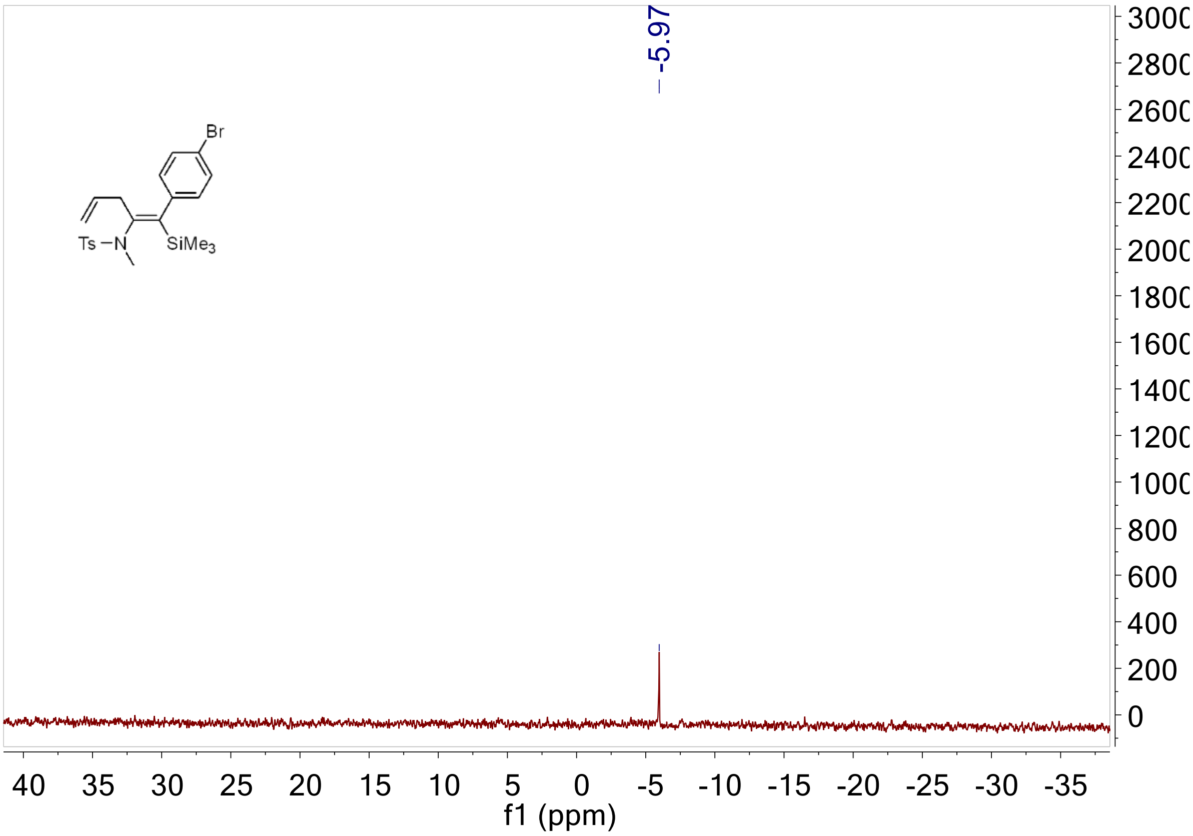

^{1}H NMR (300 MHz, $CDCl_3$) spectrum of 3d

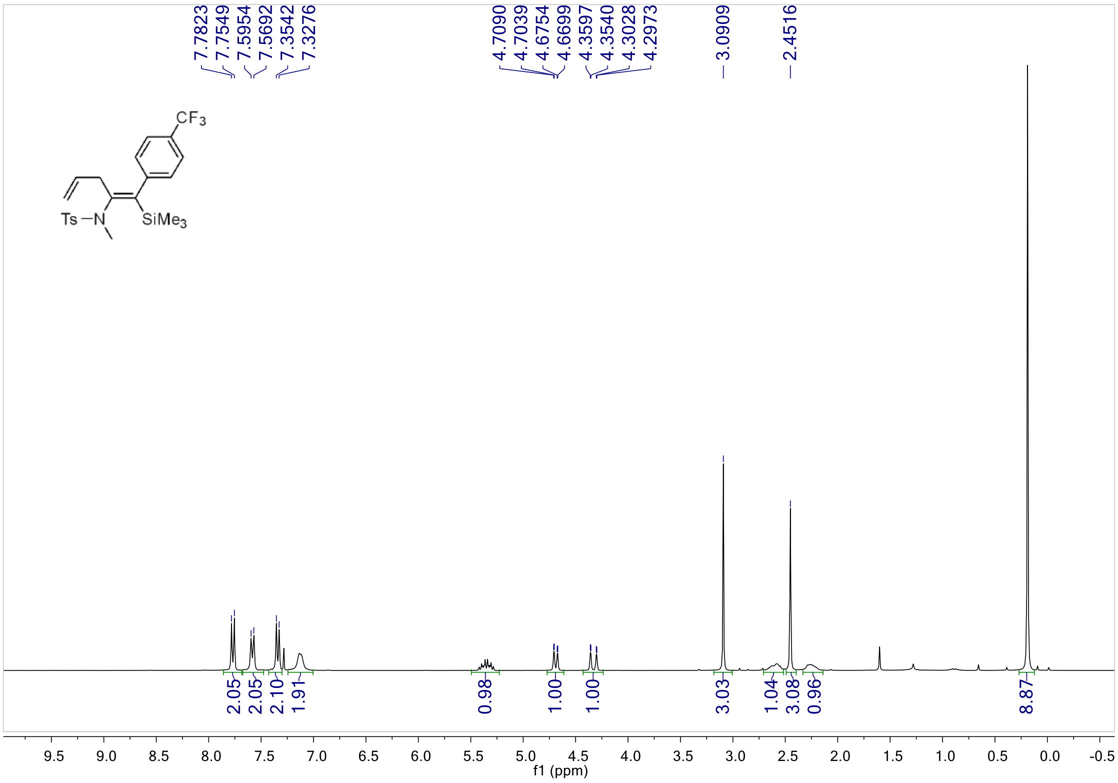

^{13}C NMR (75 MHz, $CDCl_3$) spectrum of 3d

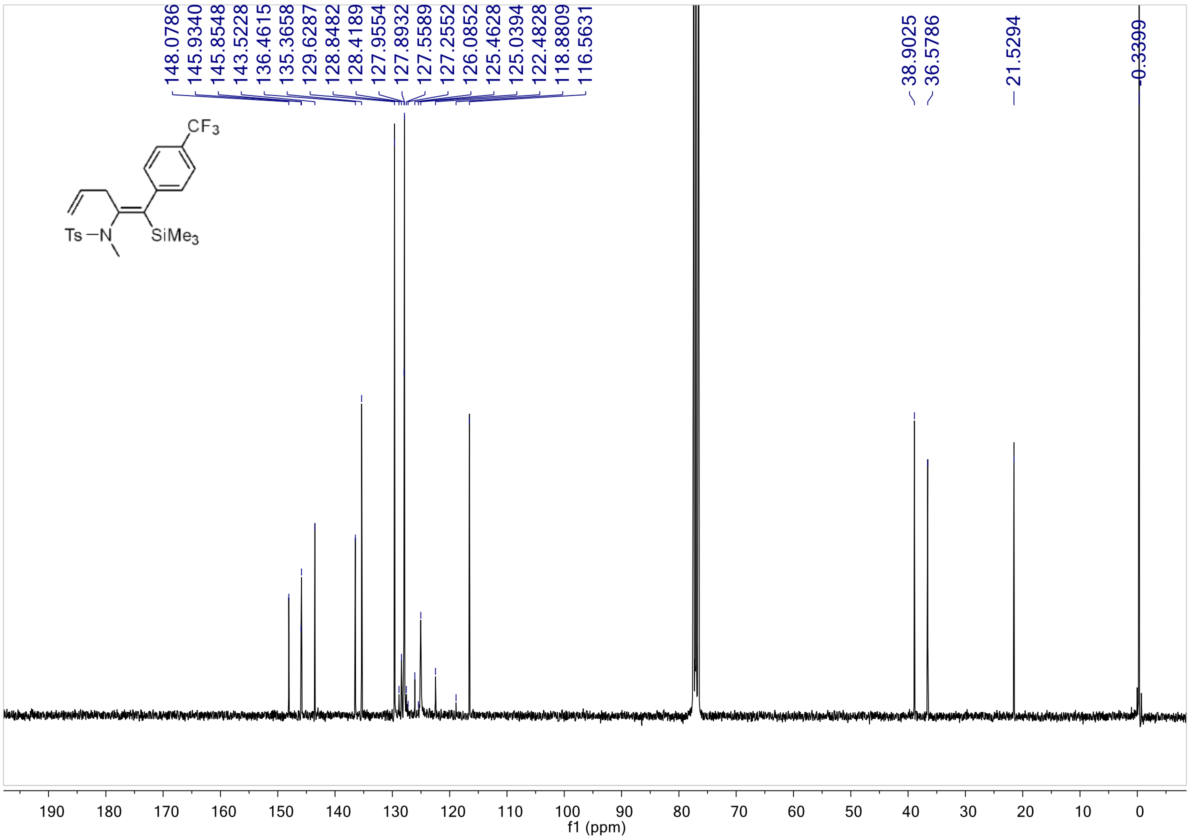

^{29}Si NMR (75 MHz, $CDCl_3$) spectrum of 3d

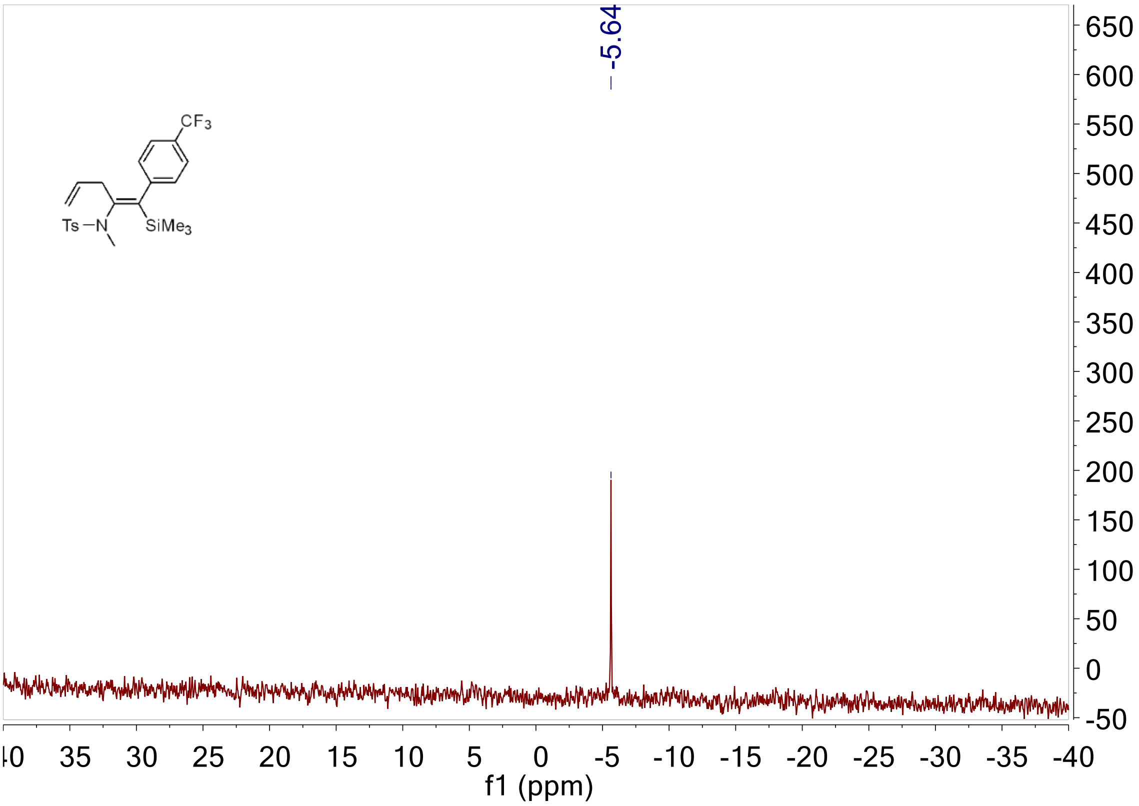

^{19}F NMR (282 MHz, $CDCl_3$) spectrum of 3d

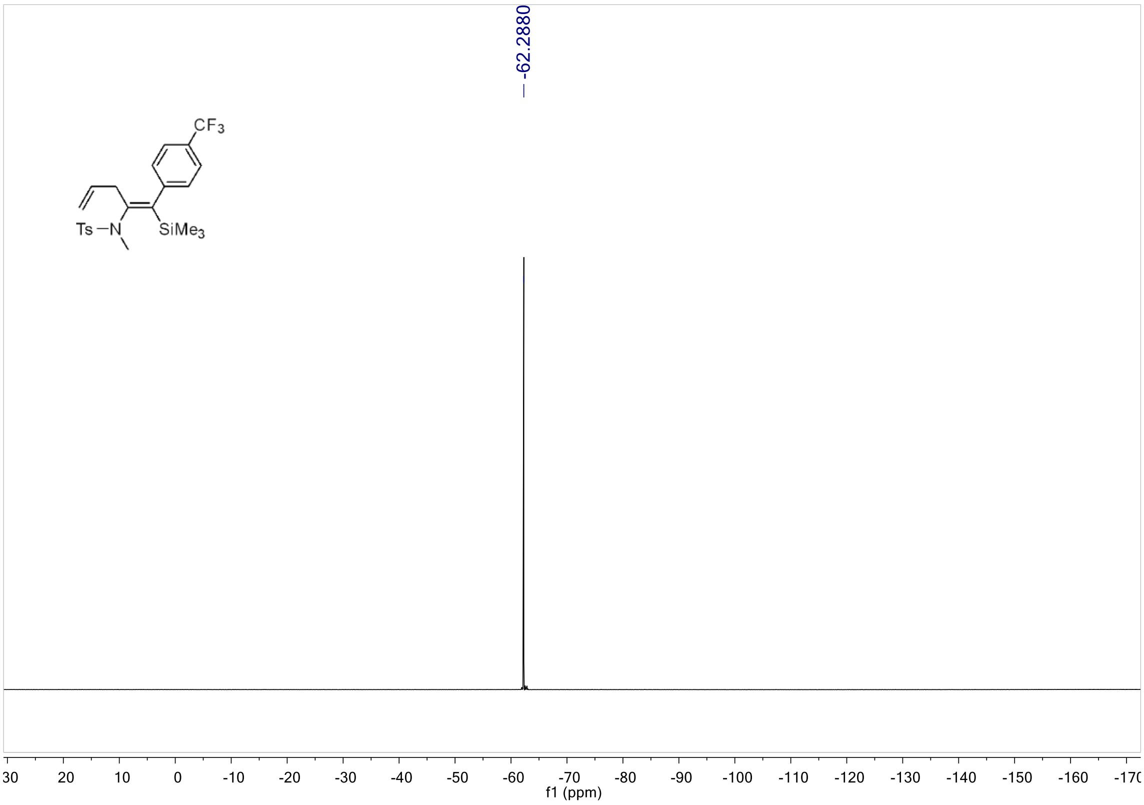

^{1}H NMR (300 MHz, $CDCl_3$) spectrum of 3e

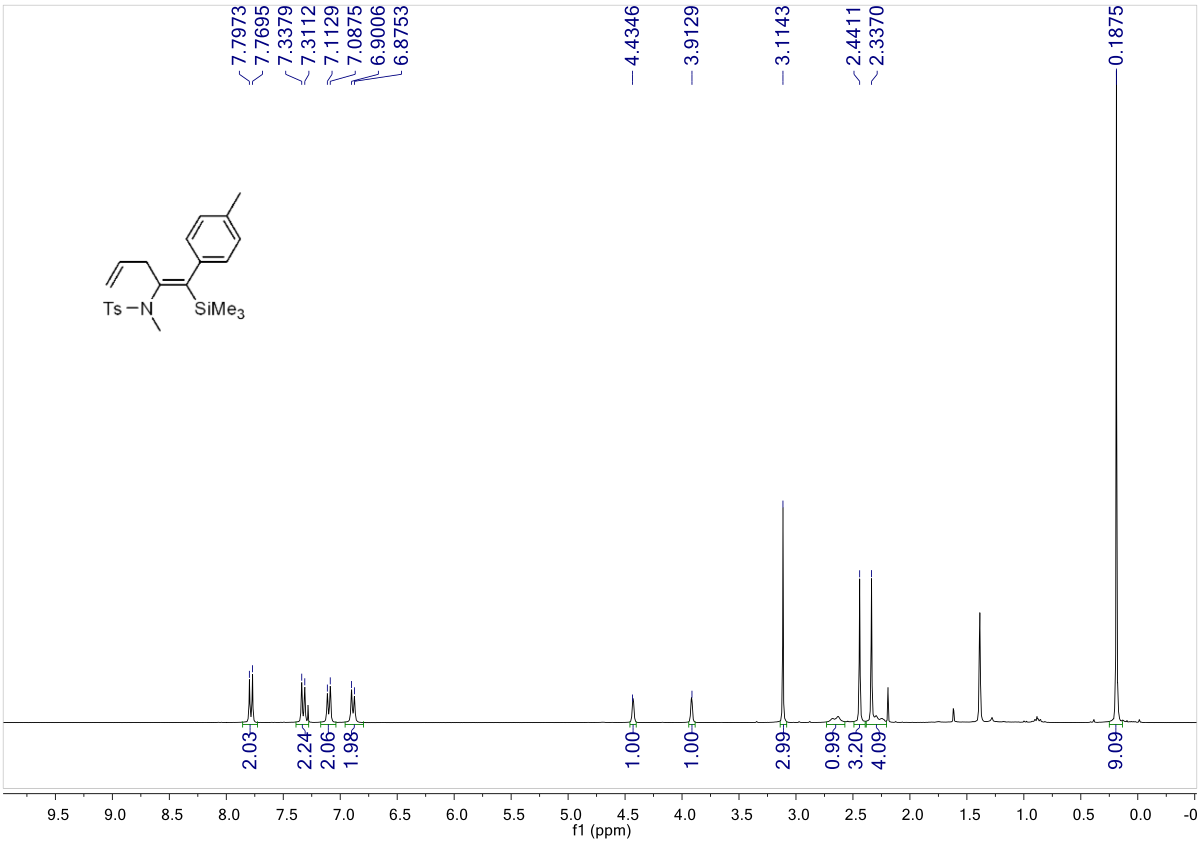

^{13}C NMR (75 MHz, $CDCl_3$) spectrum of 3e

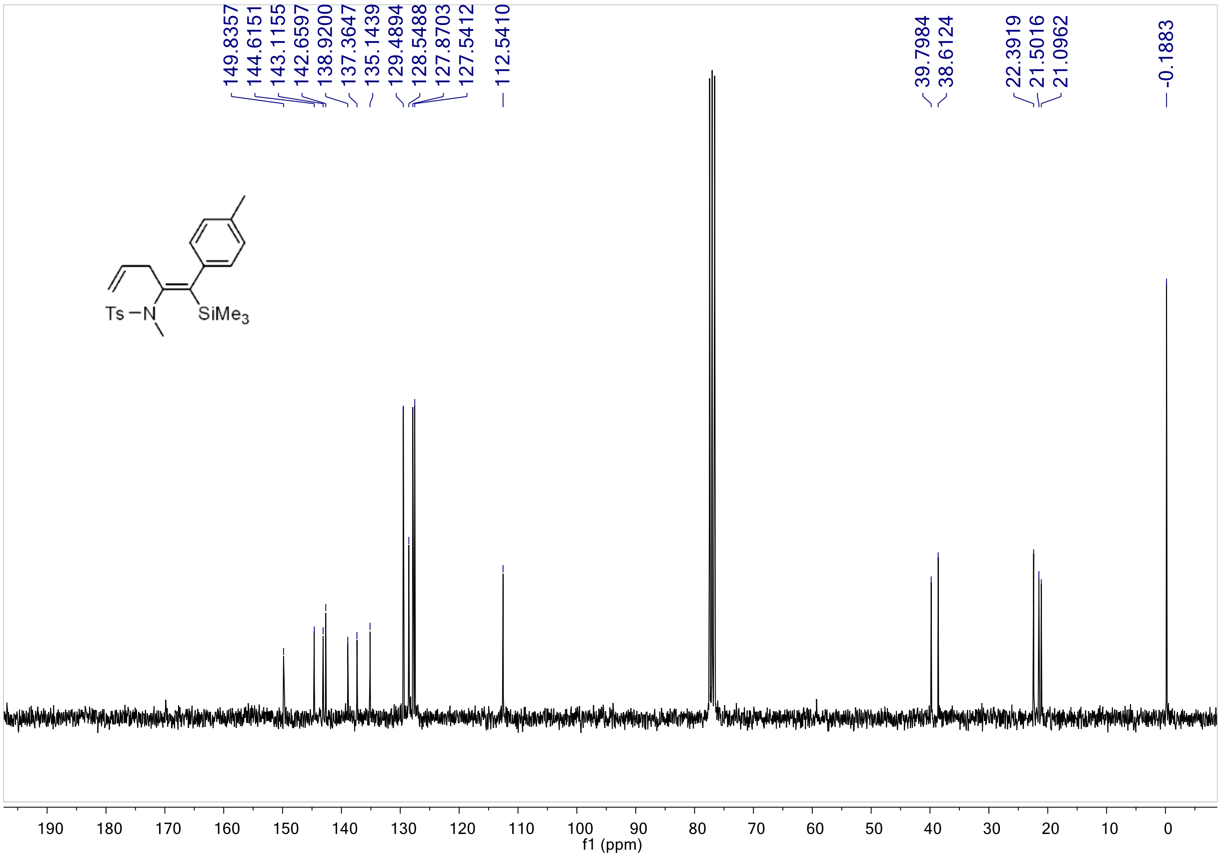

^{29}Si NMR (75 MHz, $CDCl_3$) spectrum of 3e

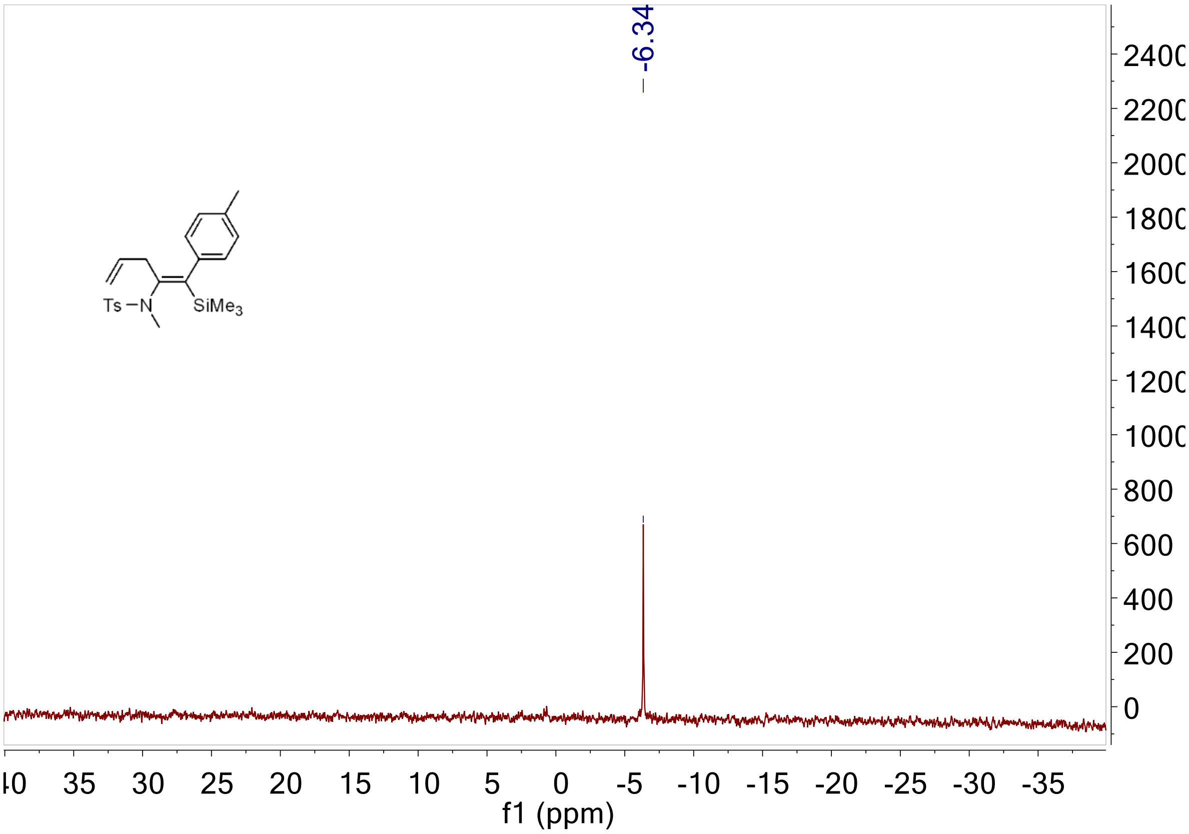

^{1}H NMR (300 MHz, $CDCl_3$) spectrum of 3f

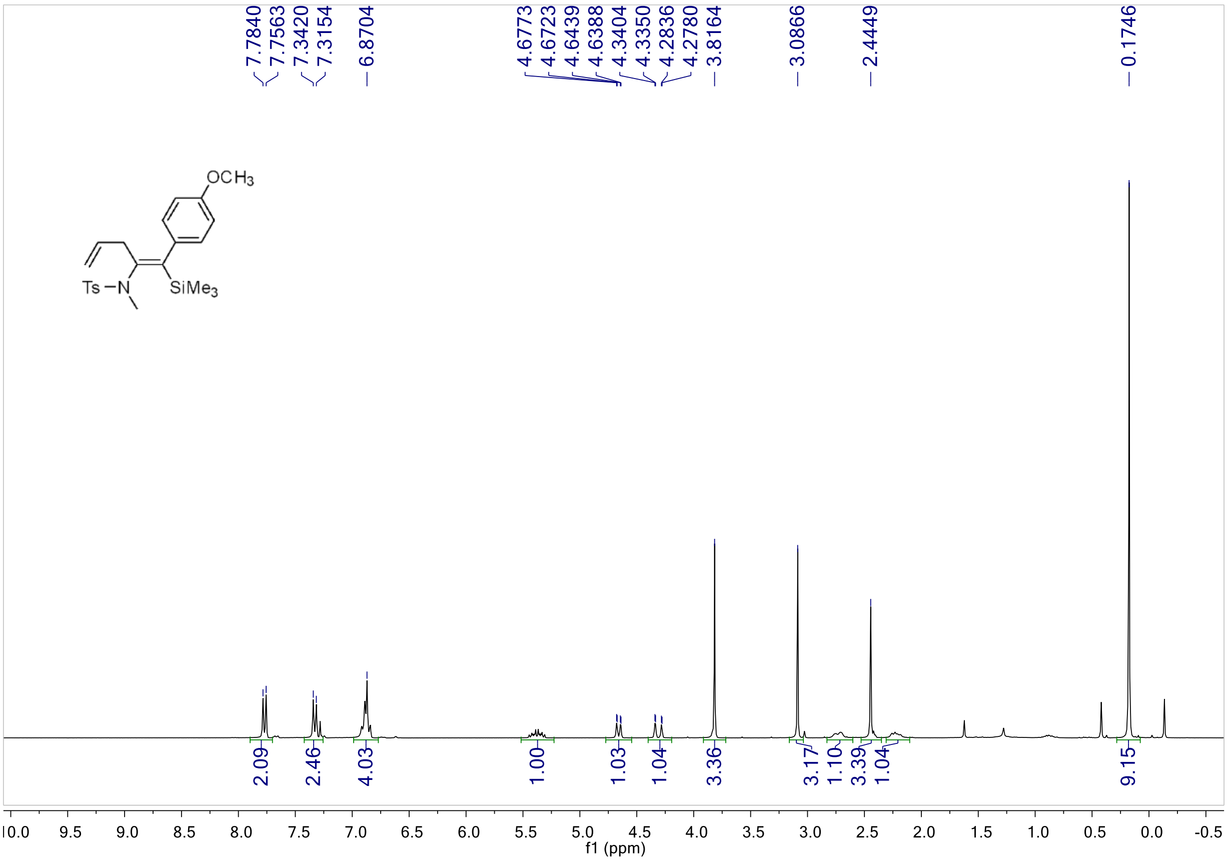

^{13}C NMR (75 MHz, $CDCl_3$) spectrum of 3f

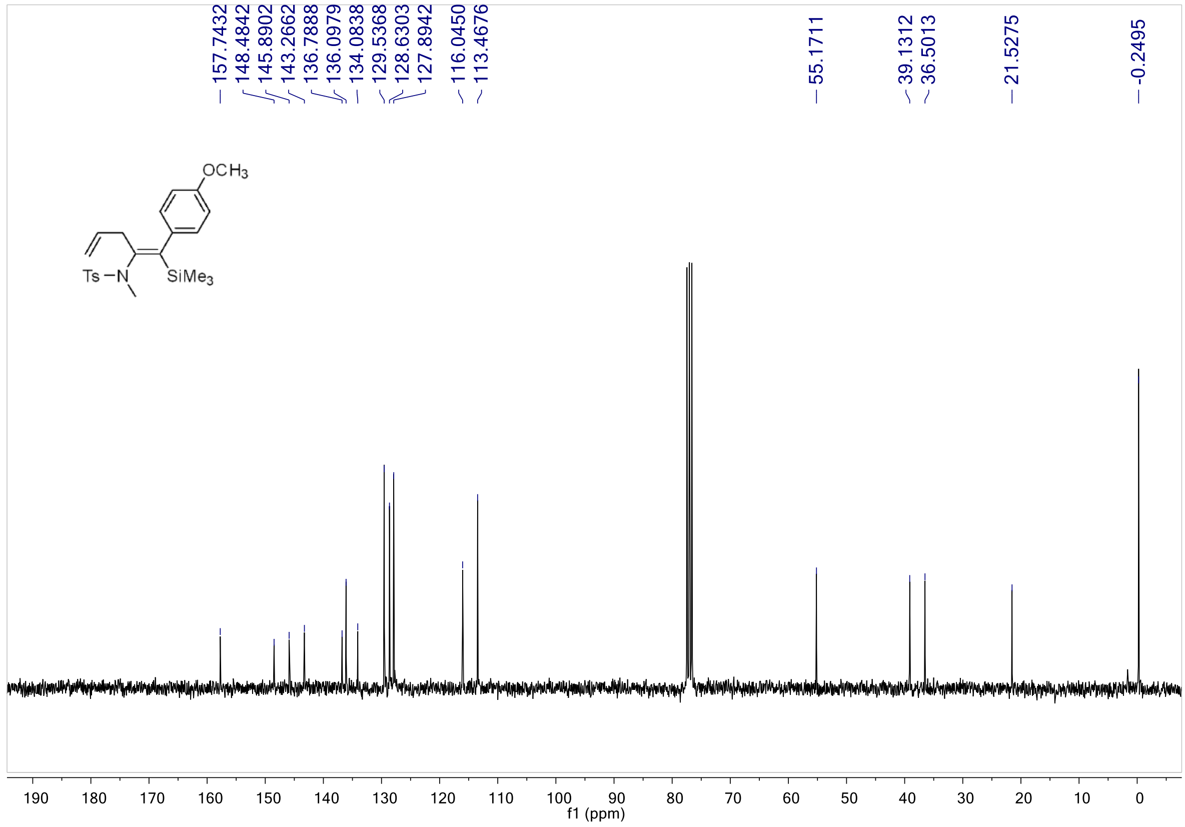

^{29}Si NMR (75 MHz, $CDCl_3$) spectrum of 3f

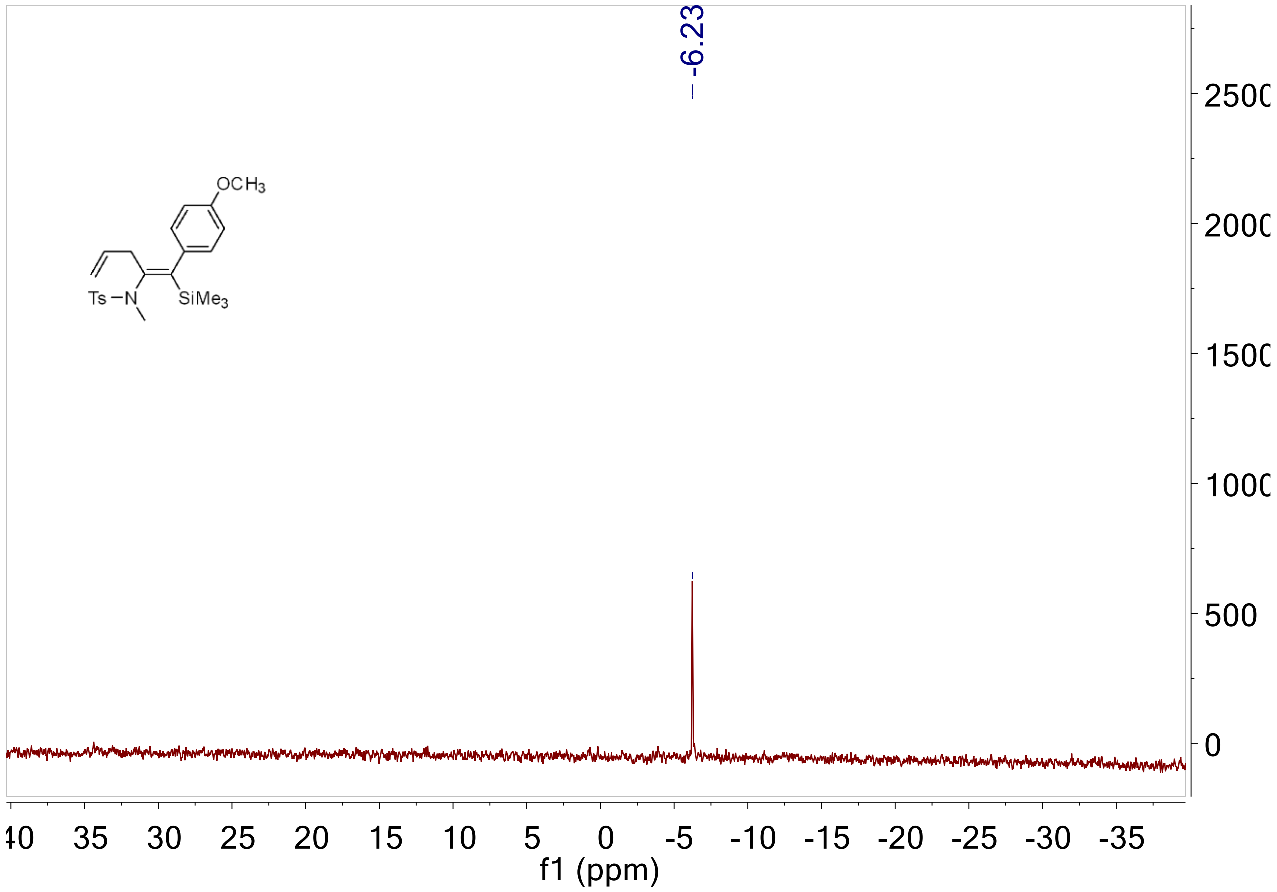

^{1}H NMR (300 MHz, CDCl3) spectrum of 3g

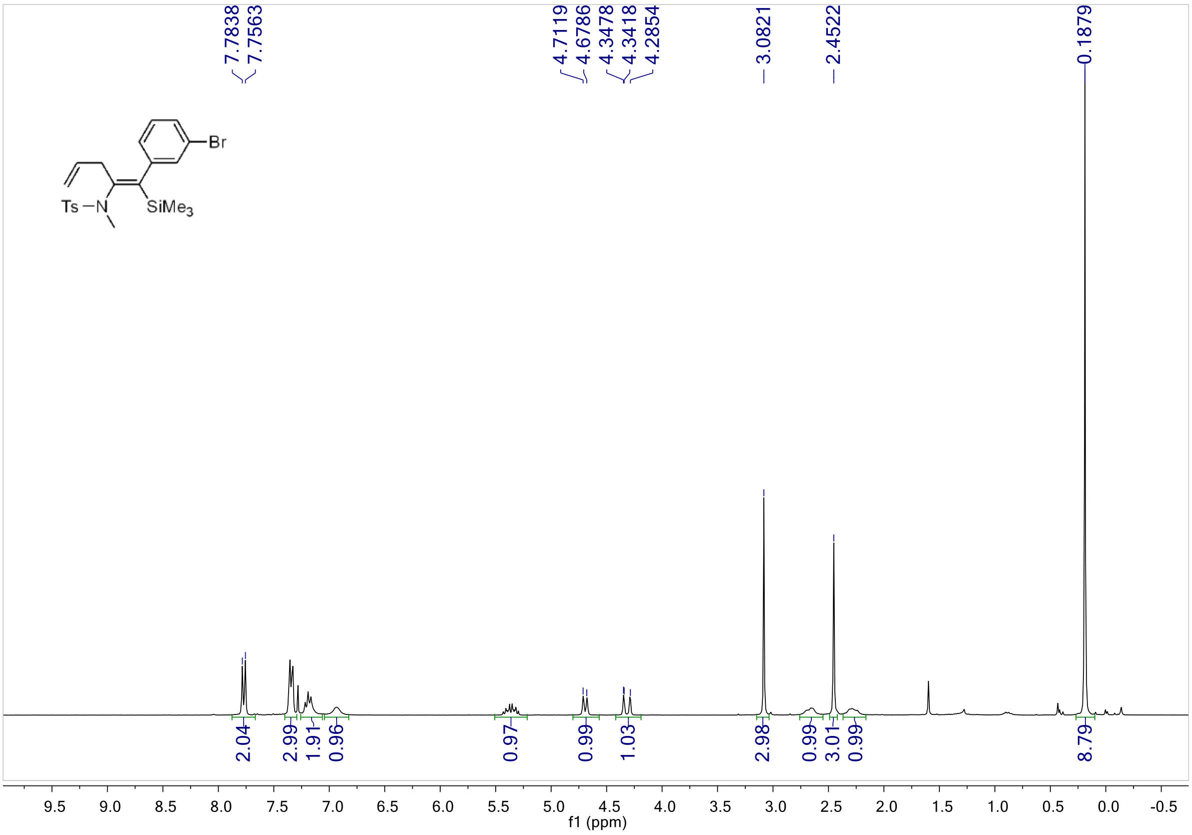

^{13}C NMR (75 MHz, CDCl3) spectrum of 3g

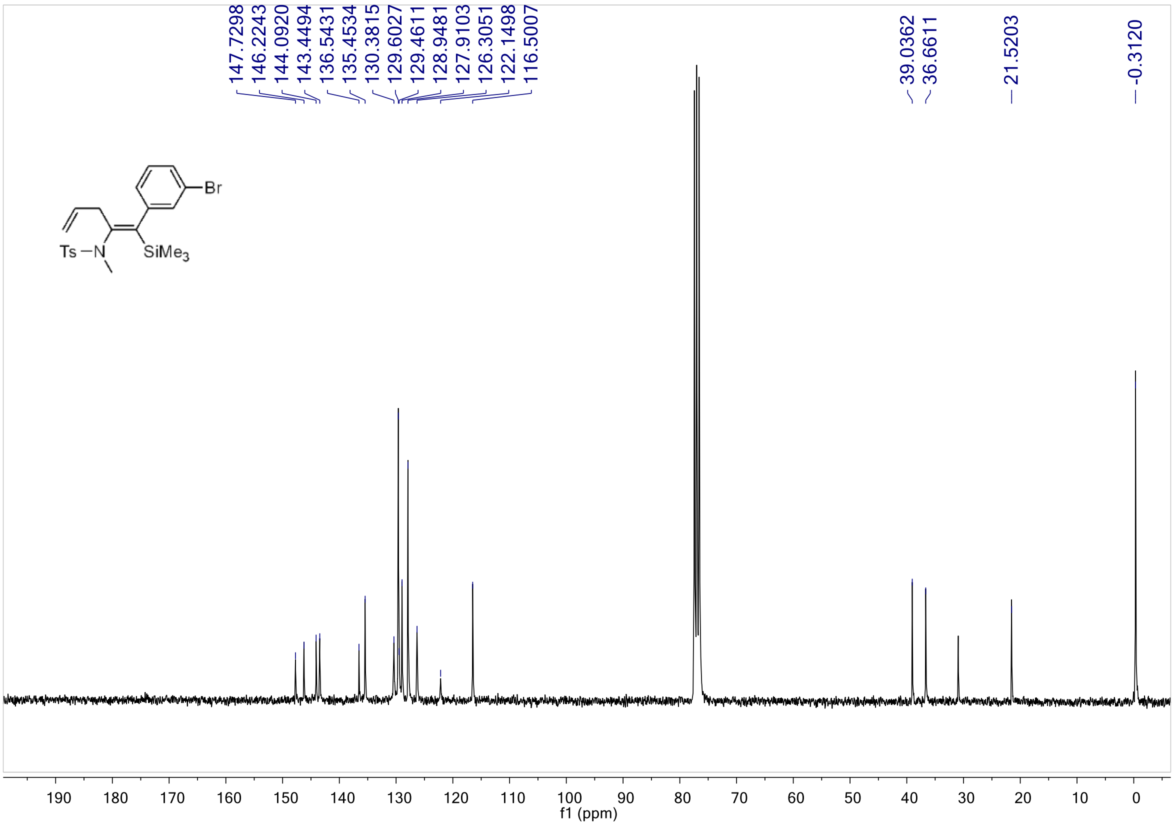

^{29}Si NMR (75 MHz, $CDCl_3$) spectrum of 3g

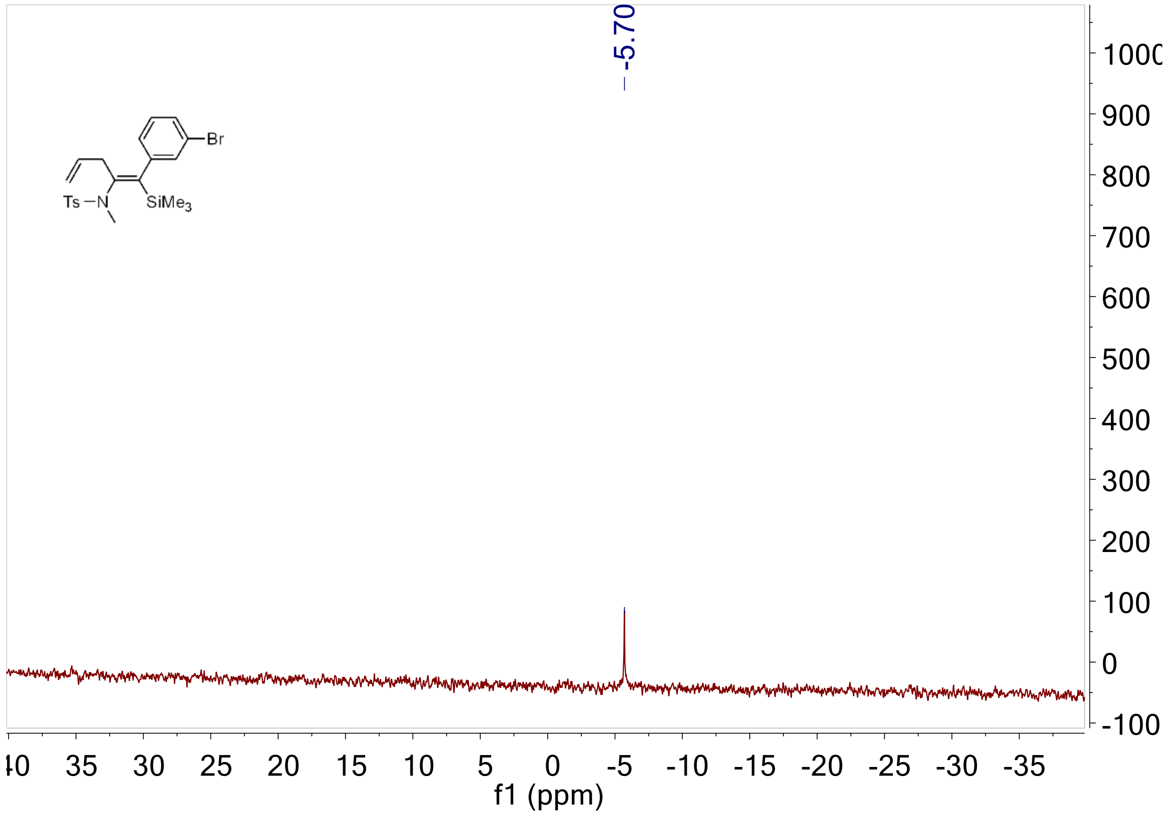

^{1}H NMR (300 MHz, $CDCl_3$) spectrum of 3h

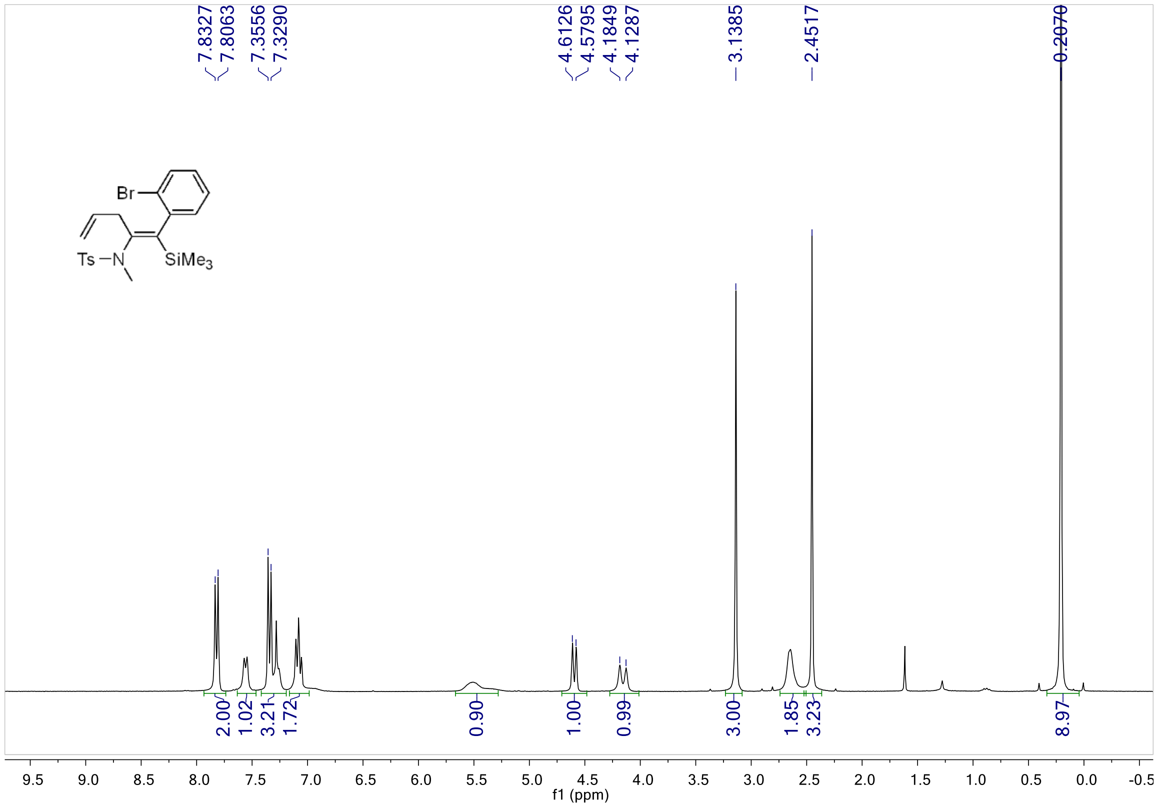

^{13}C NMR (75 MHz, $CDCl_3$) spectrum of 3h

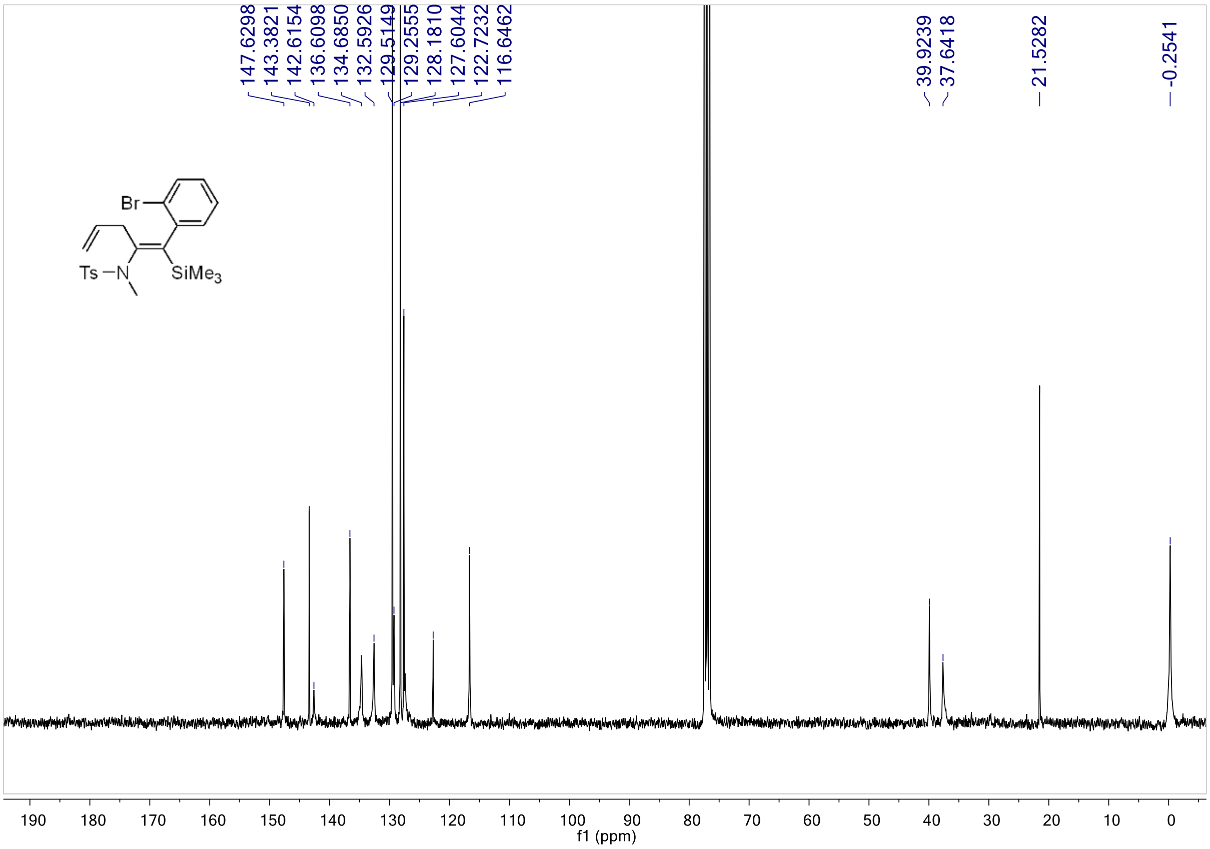

^{29}Si NMR (75 MHz, $CDCl_3$) spectrum of 3h

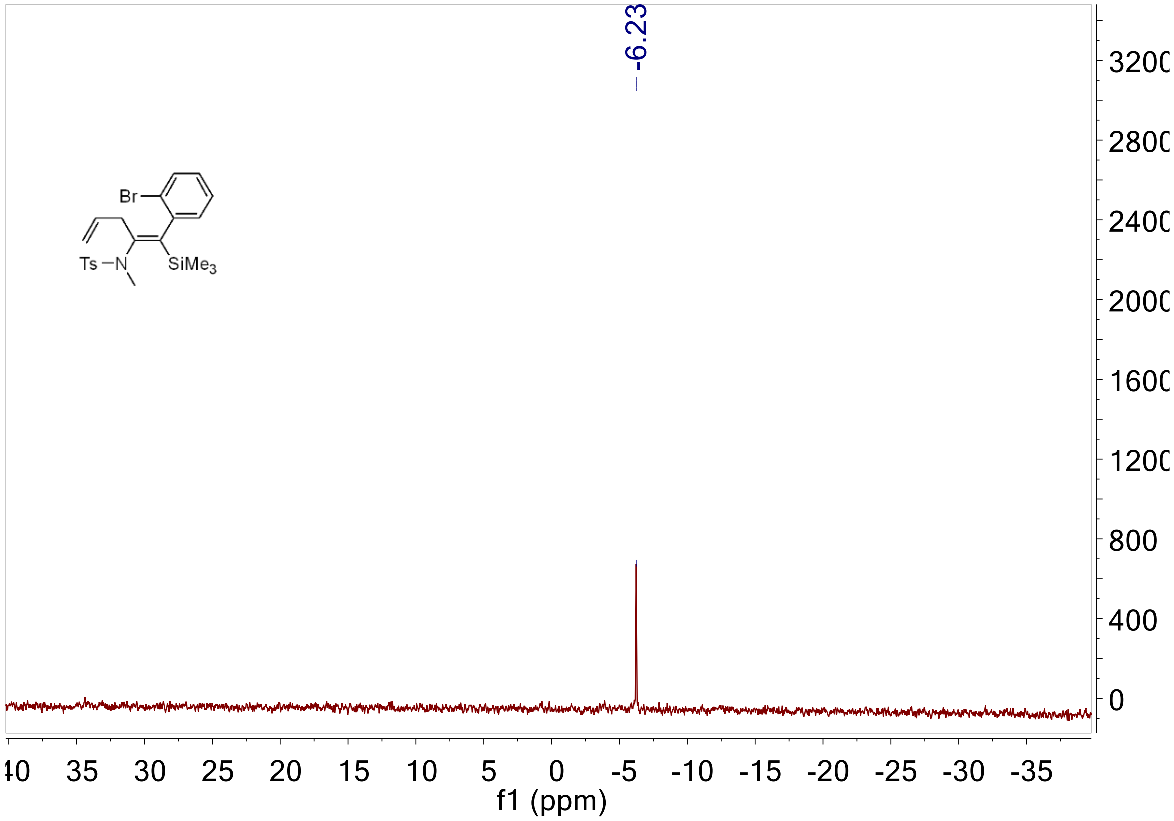

^{1}H NMR (300 MHz, $CDCl_3$) spectrum of 3i

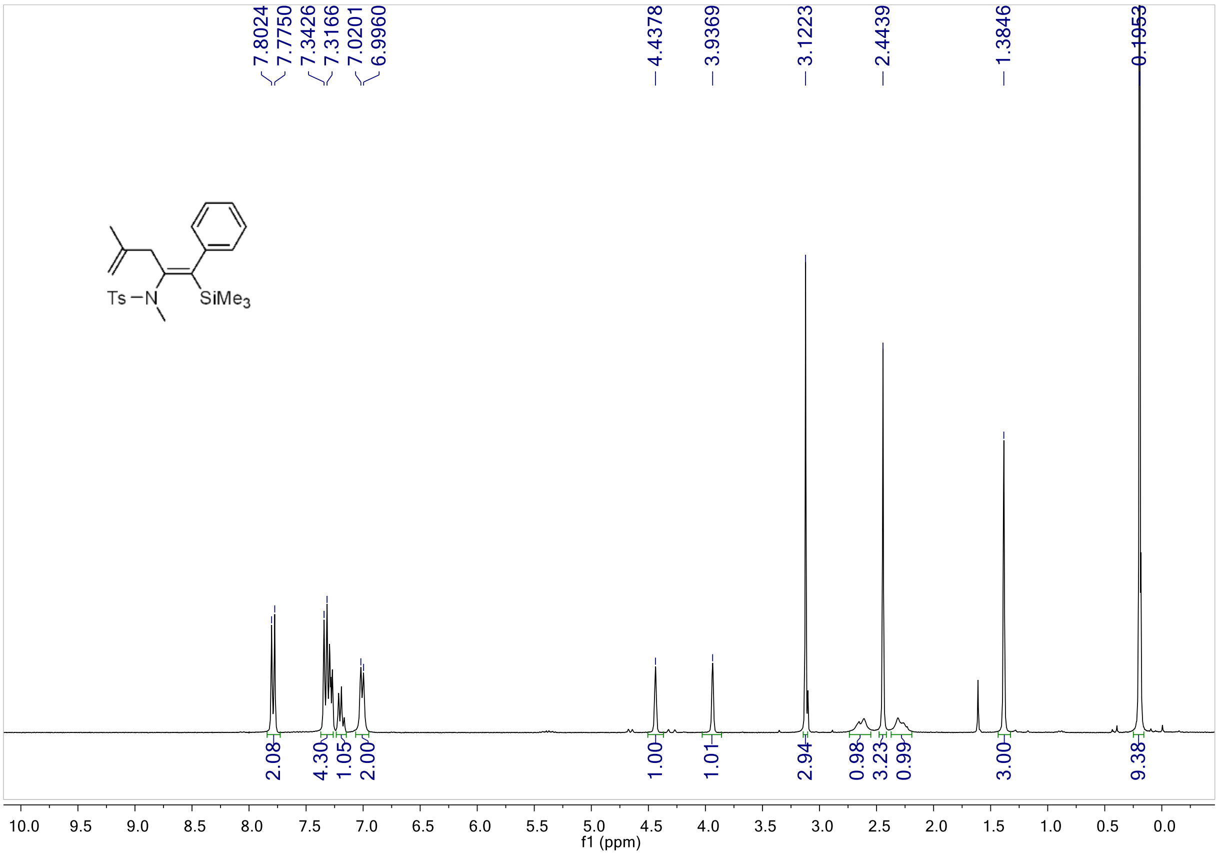

^{13}C NMR (75 MHz, $CDCl_3$) spectrum of 3i

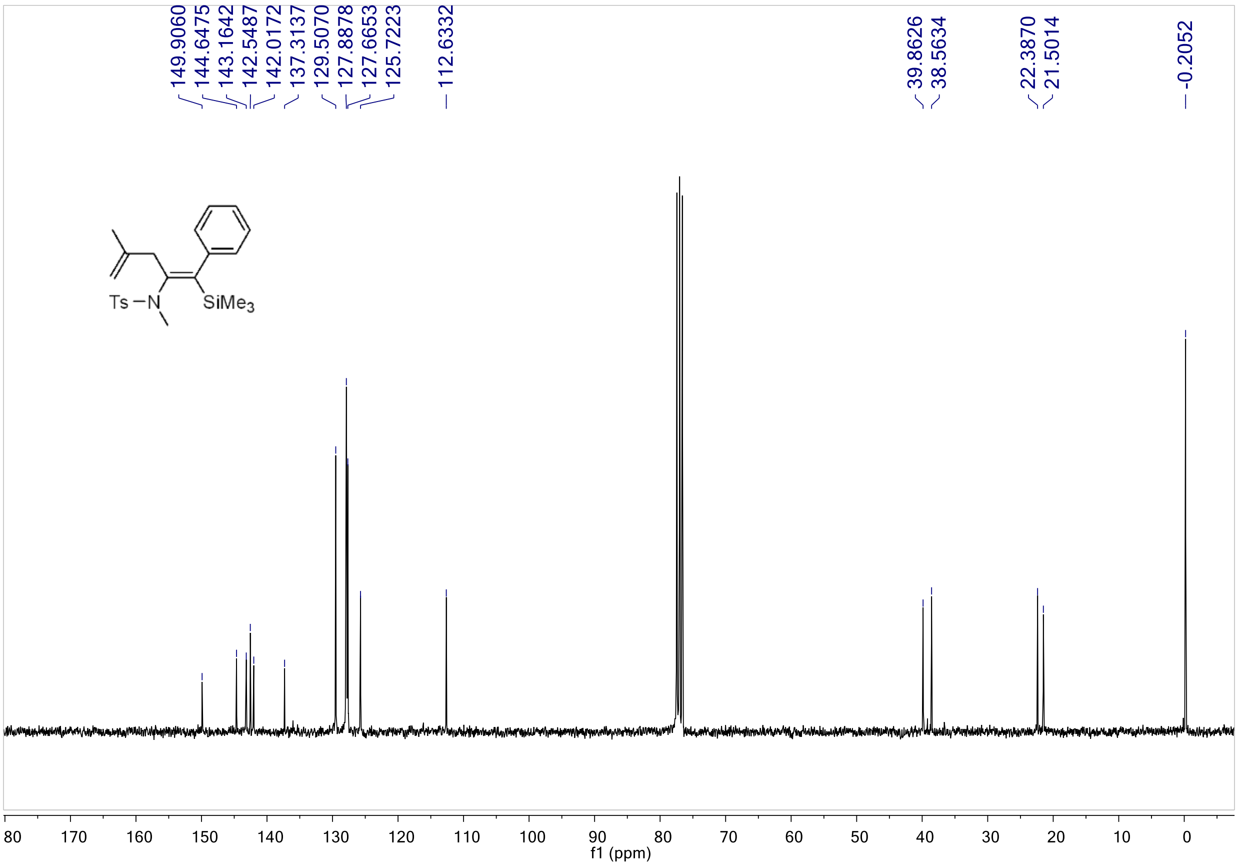

^{29}Si NMR (75 MHz, $CDCl_3$) spectrum of 3i

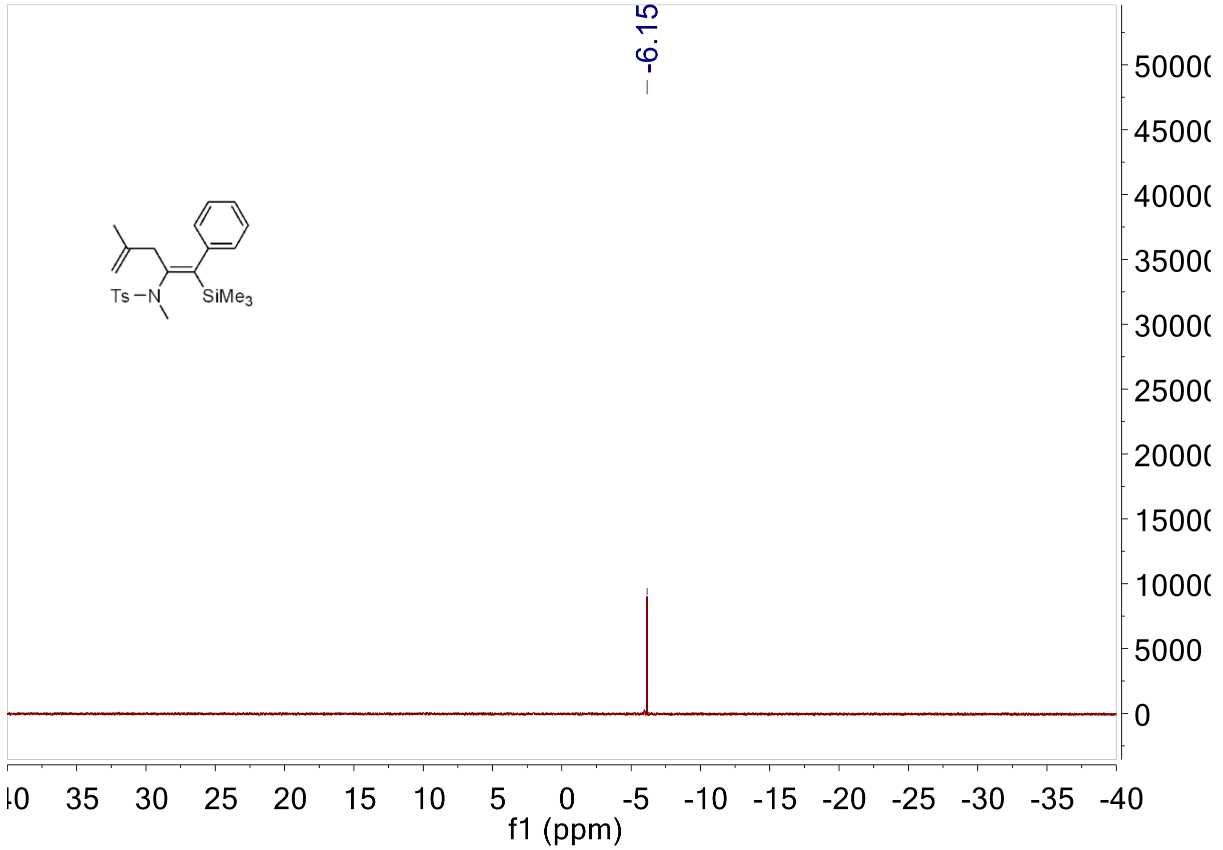

^{1}H NMR (300 MHz, $CDCl_3$) spectrum of 3j

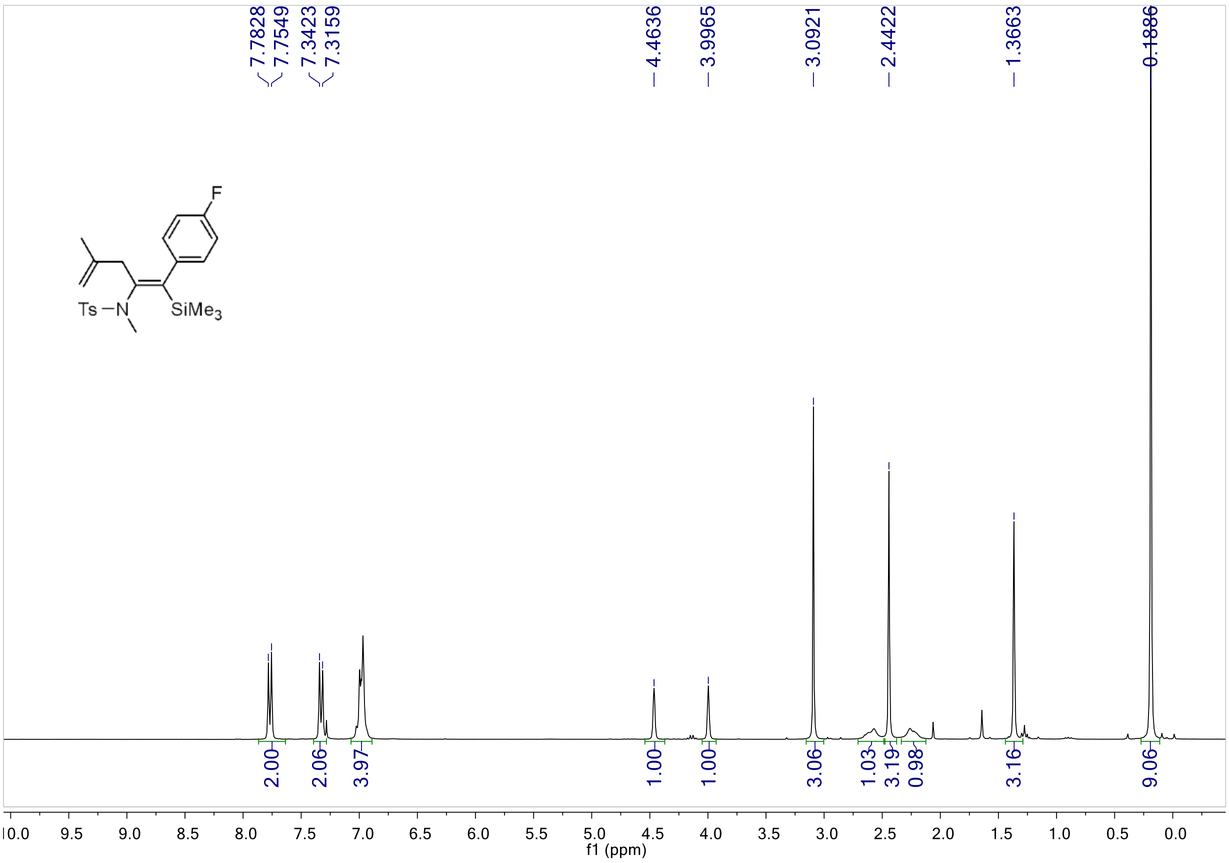

^{13}C NMR (75 MHz, CDCl3) spectrum of 3j

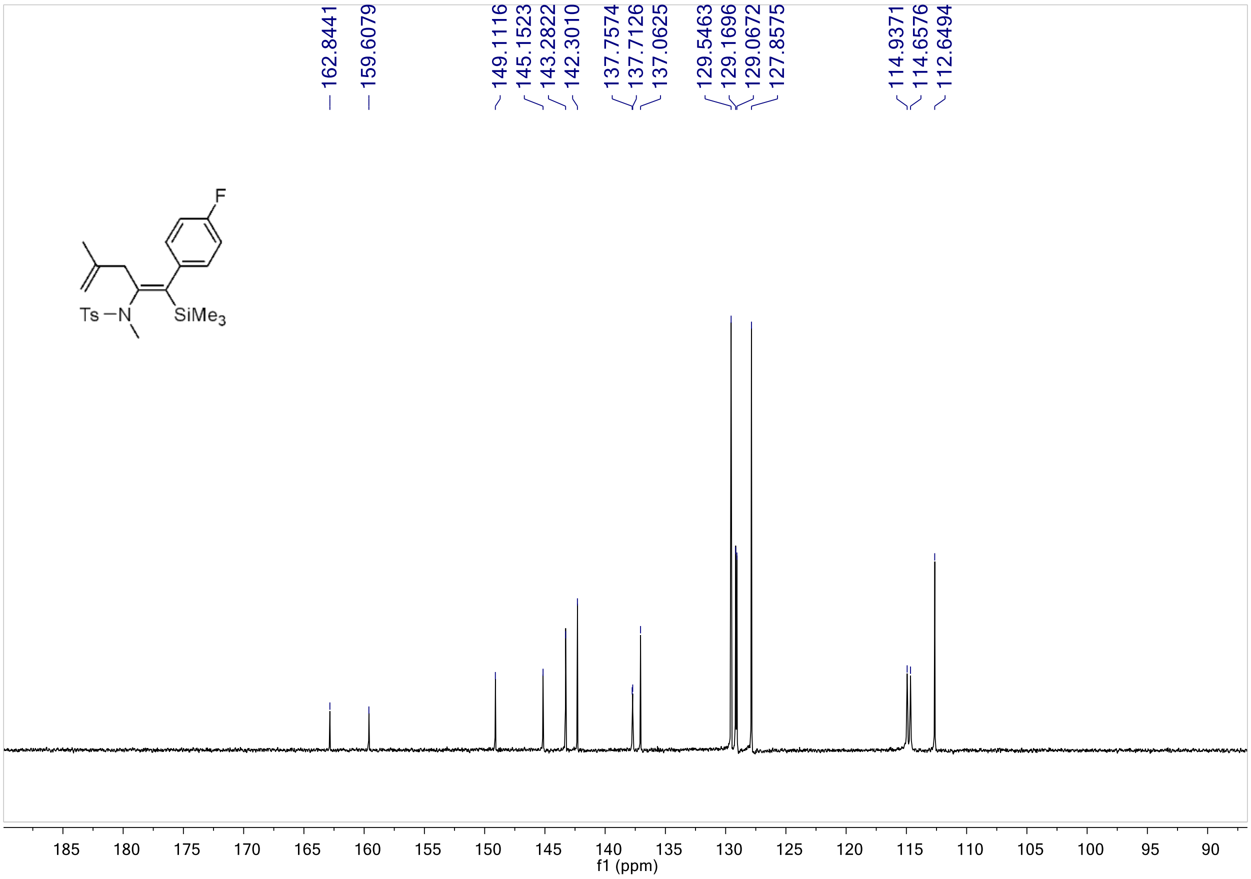

^{29}Si NMR (75 MHz, CDCl3) spectrum of 3j

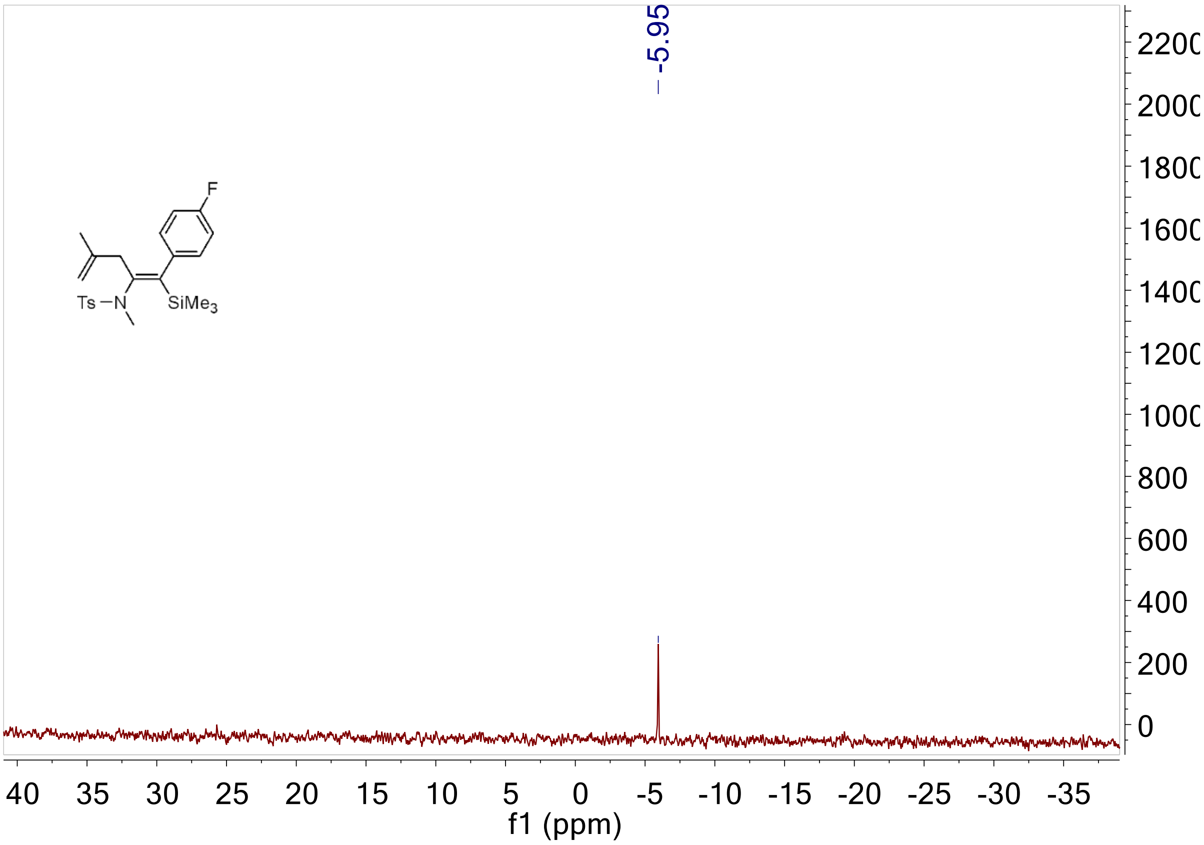

^{19}F NMR (282 MHz, CDCl$_3$) spectrum of 3j

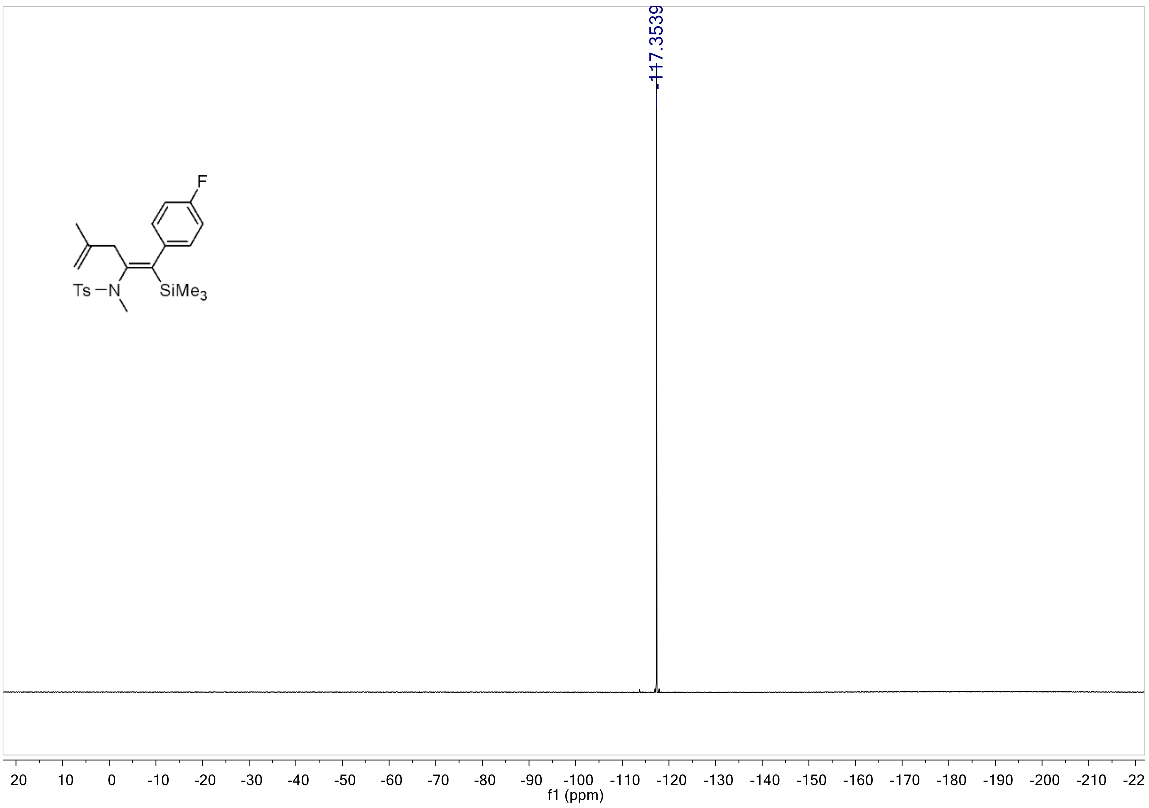

^{1}H NMR (300 MHz, CDCl$_3$) spectrum of 3k

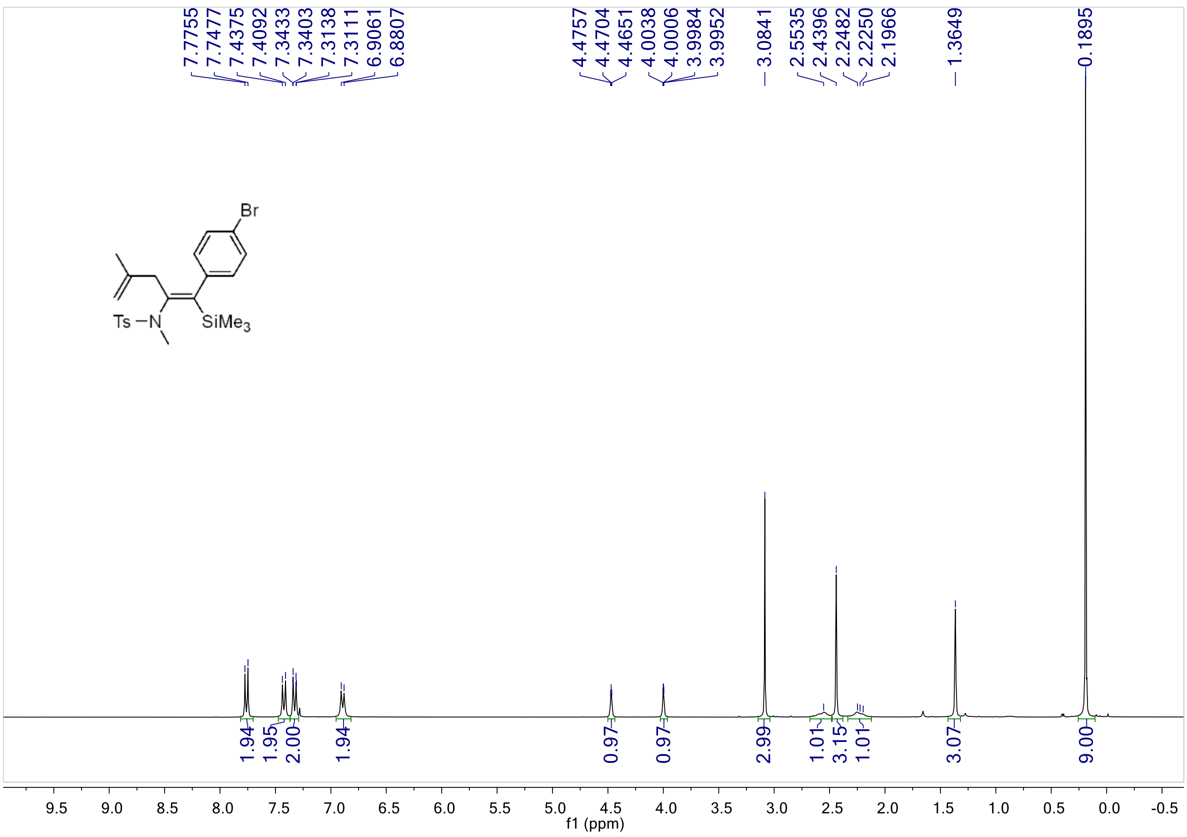

^{13}C NMR (75 MHz, $CDCl_3$) spectrum of 3k

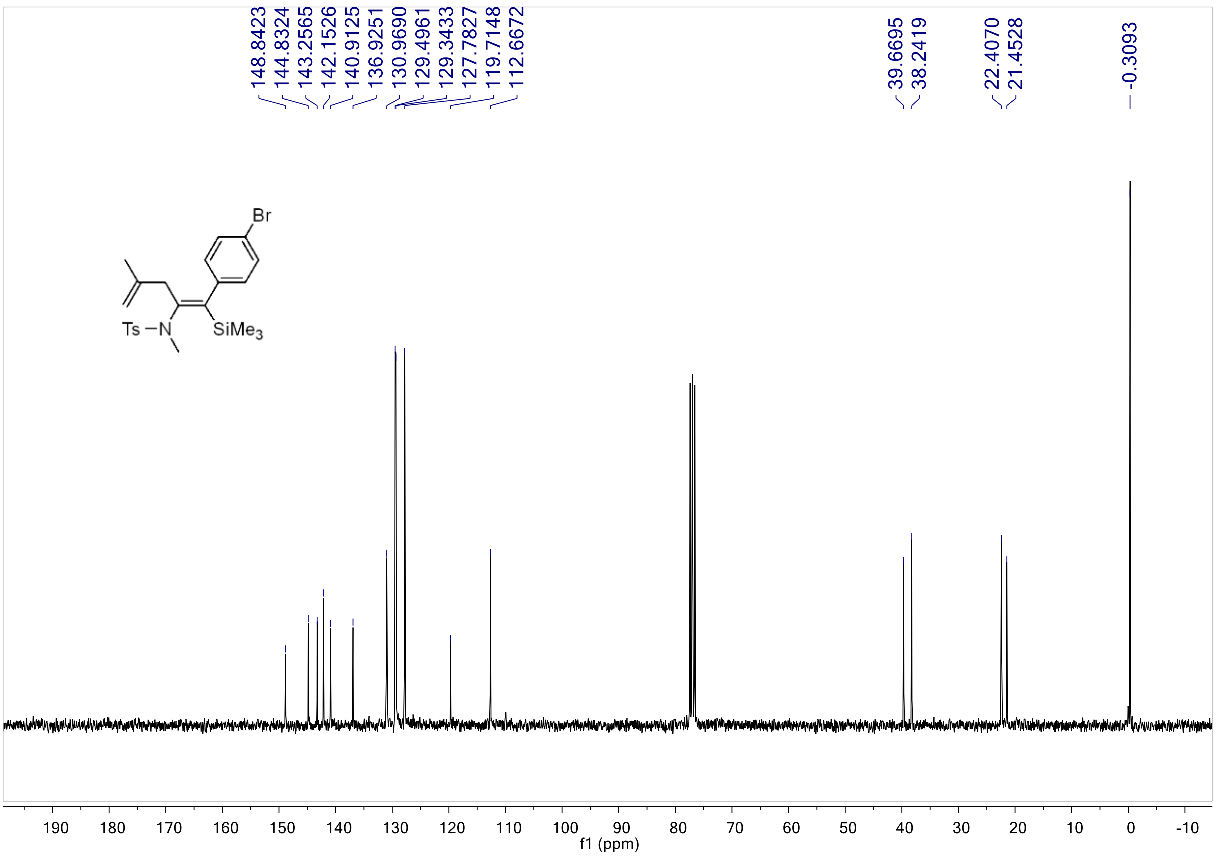

^{29}Si NMR (75 MHz, $CDCl_3$) spectrum of 3k

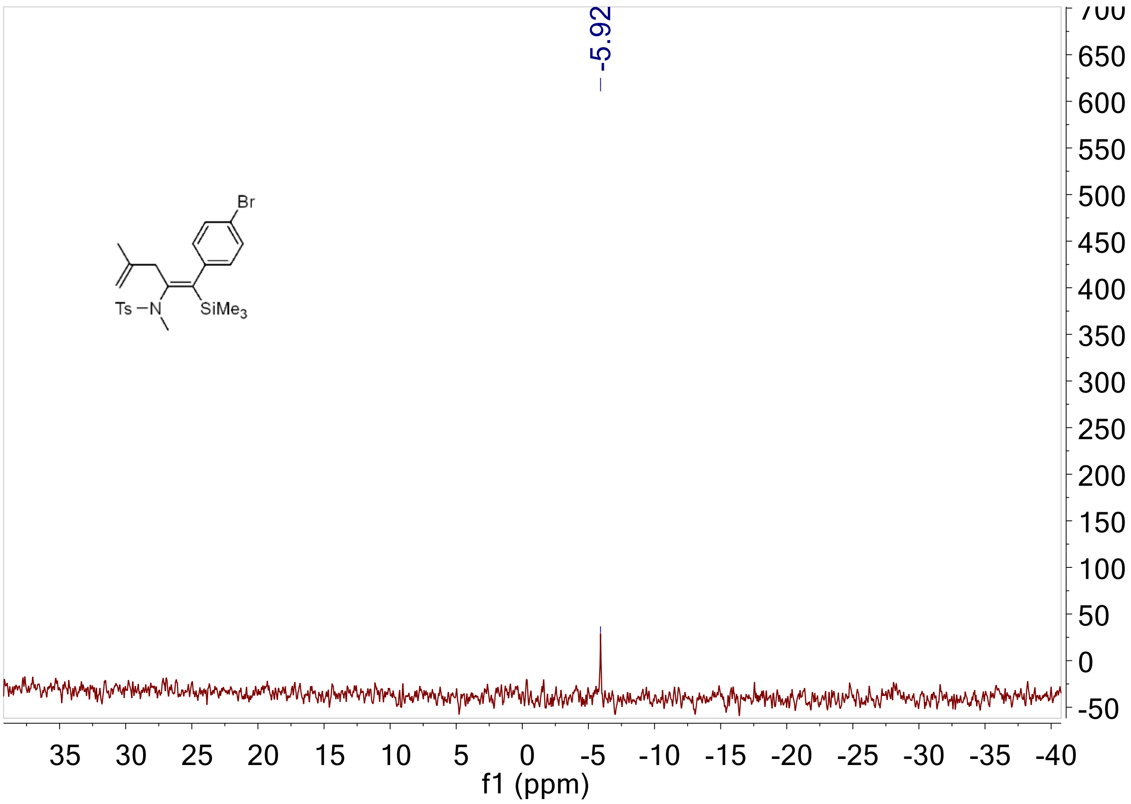

^{1}H NMR (300 MHz, CDCl$_3$) spectrum of 3l

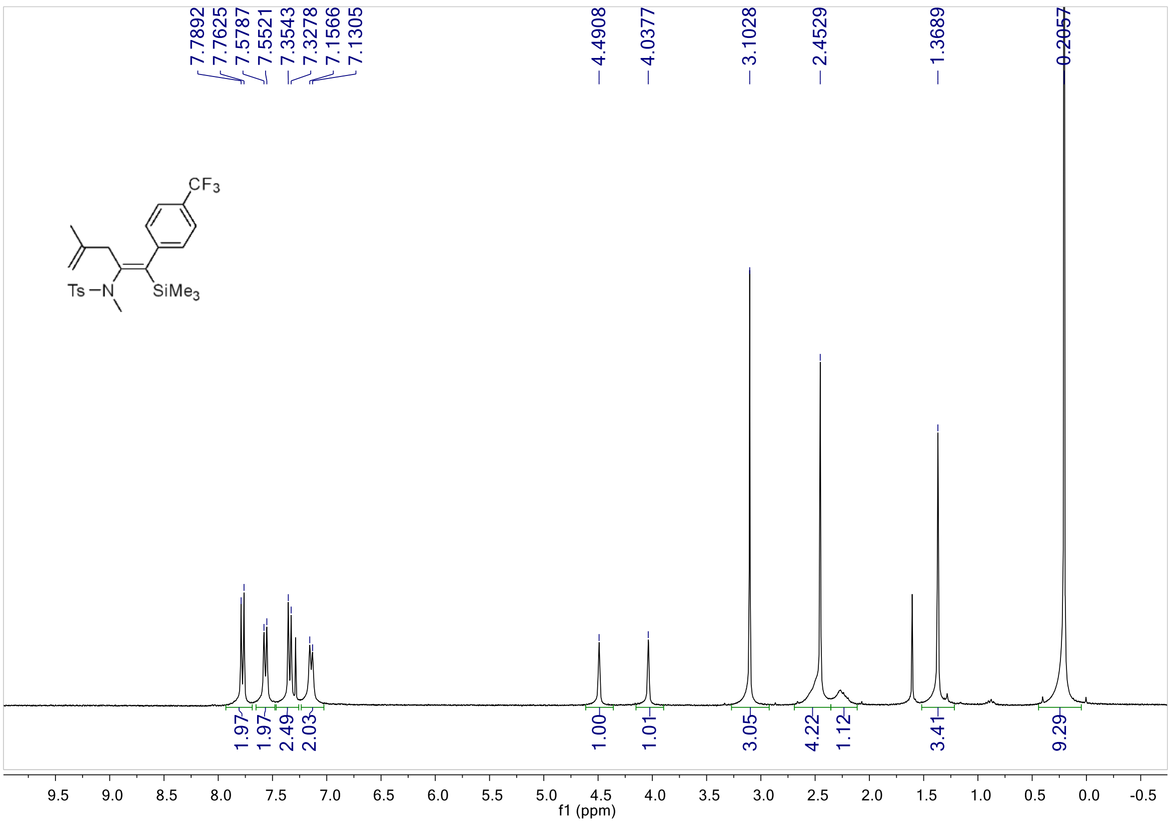

^{13}C NMR (75 MHz, CDCl$_3$) spectrum of 3l

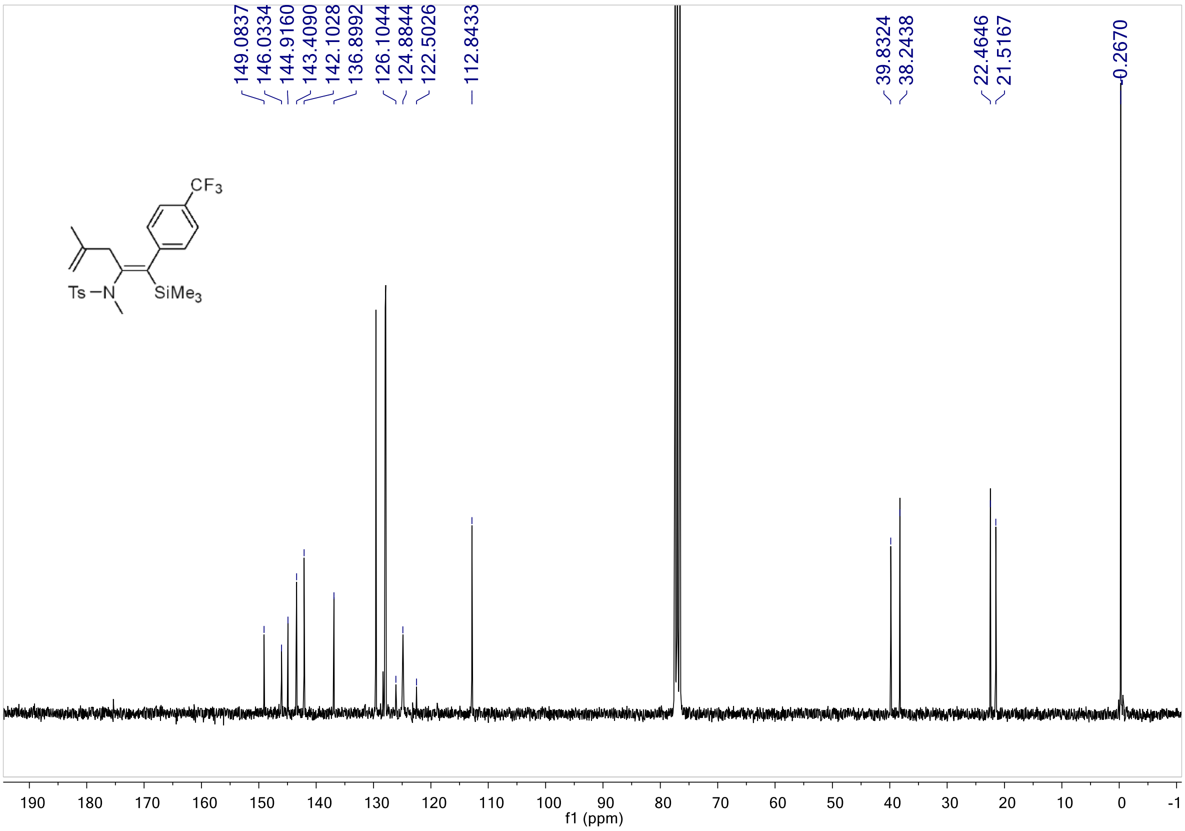

^{29}Si NMR (75 MHz, CDCl$_3$) spectrum of 3l

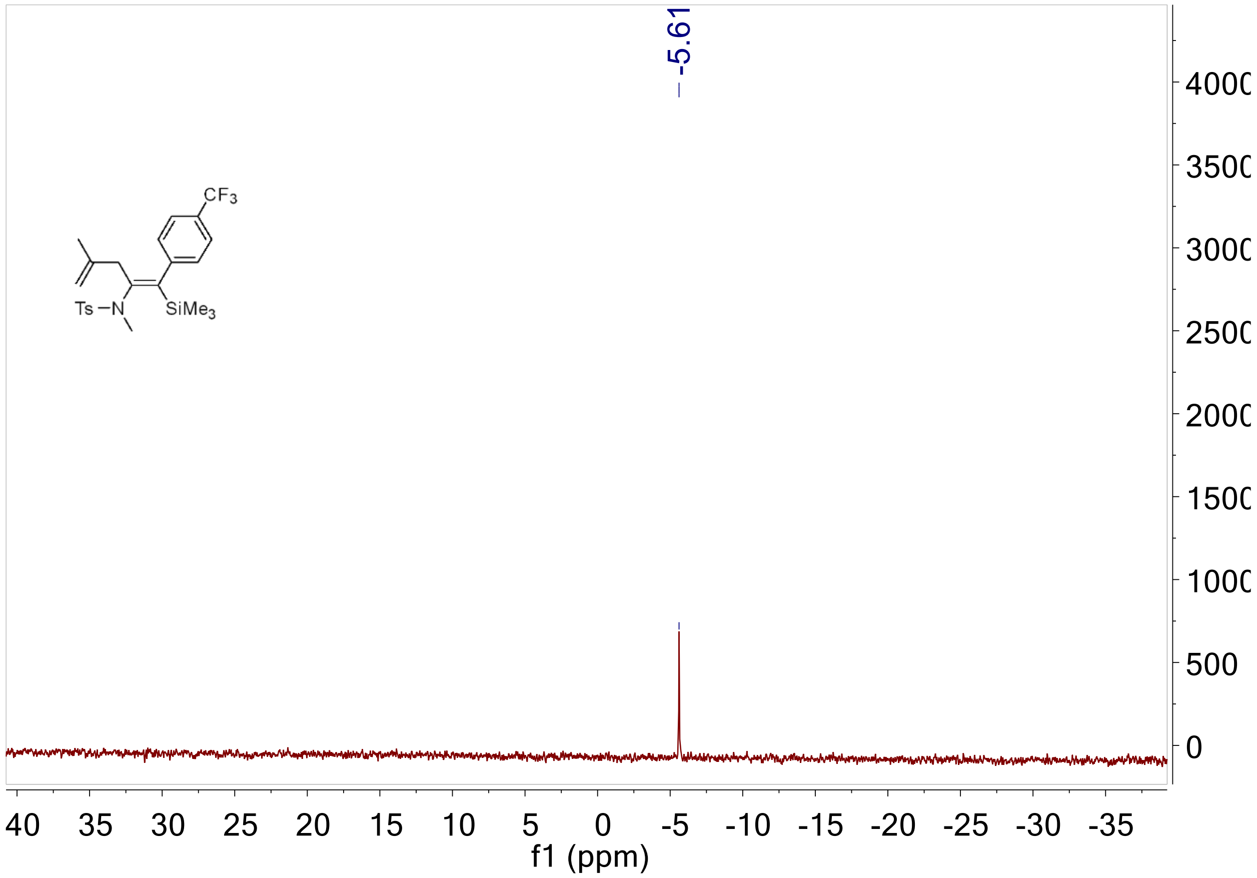

^{19}F NMR (282 MHz, CDCl$_3$) spectrum of 3l

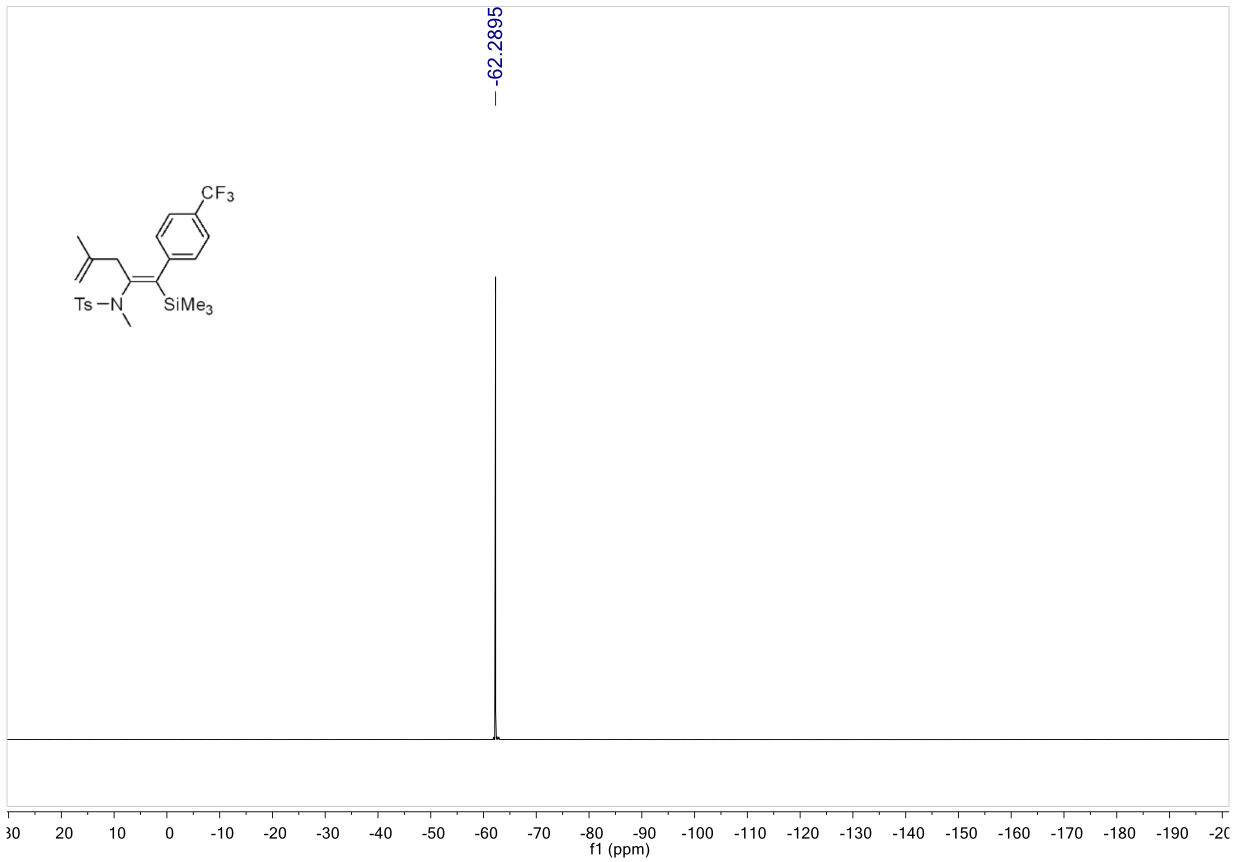

^{1}H NMR (300 MHz, CDCl3) spectrum of 3m

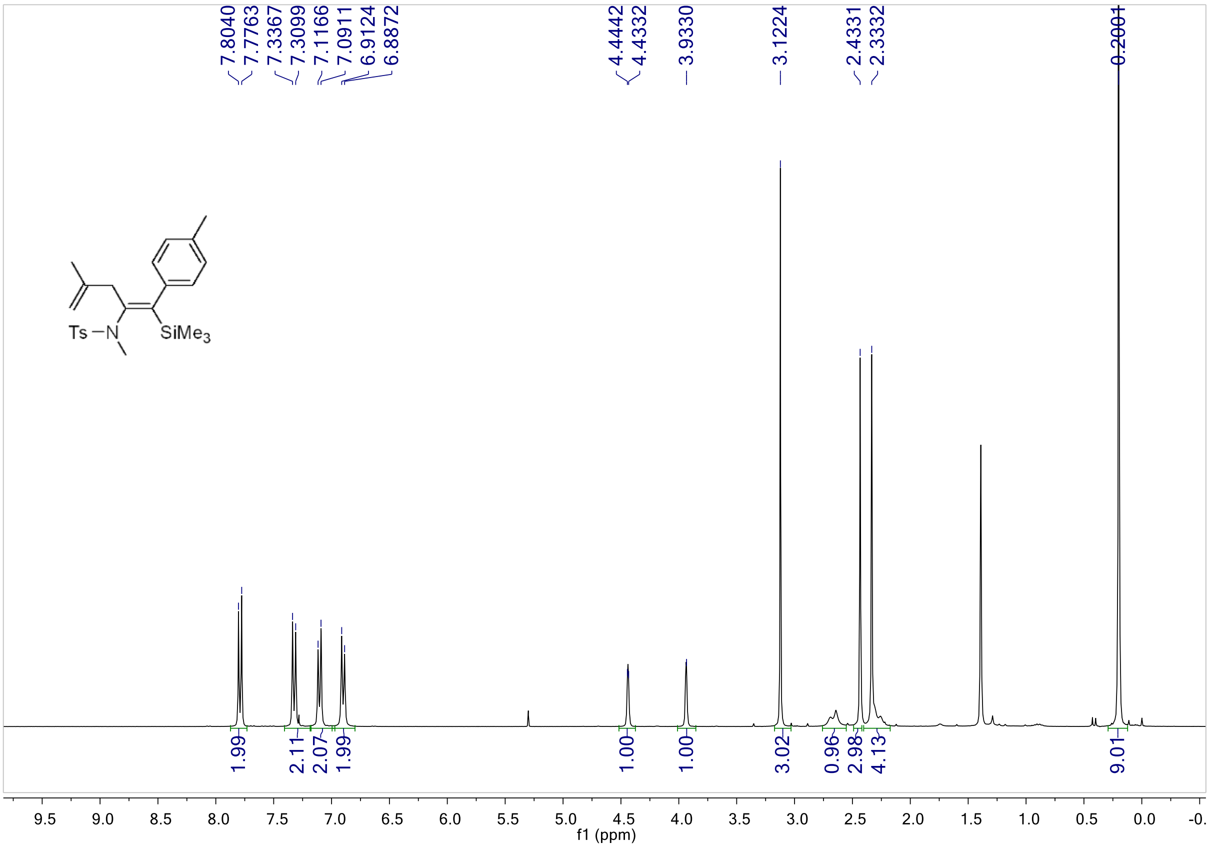

^{13}C NMR (75 MHz, CDCl3) spectrum of 3m

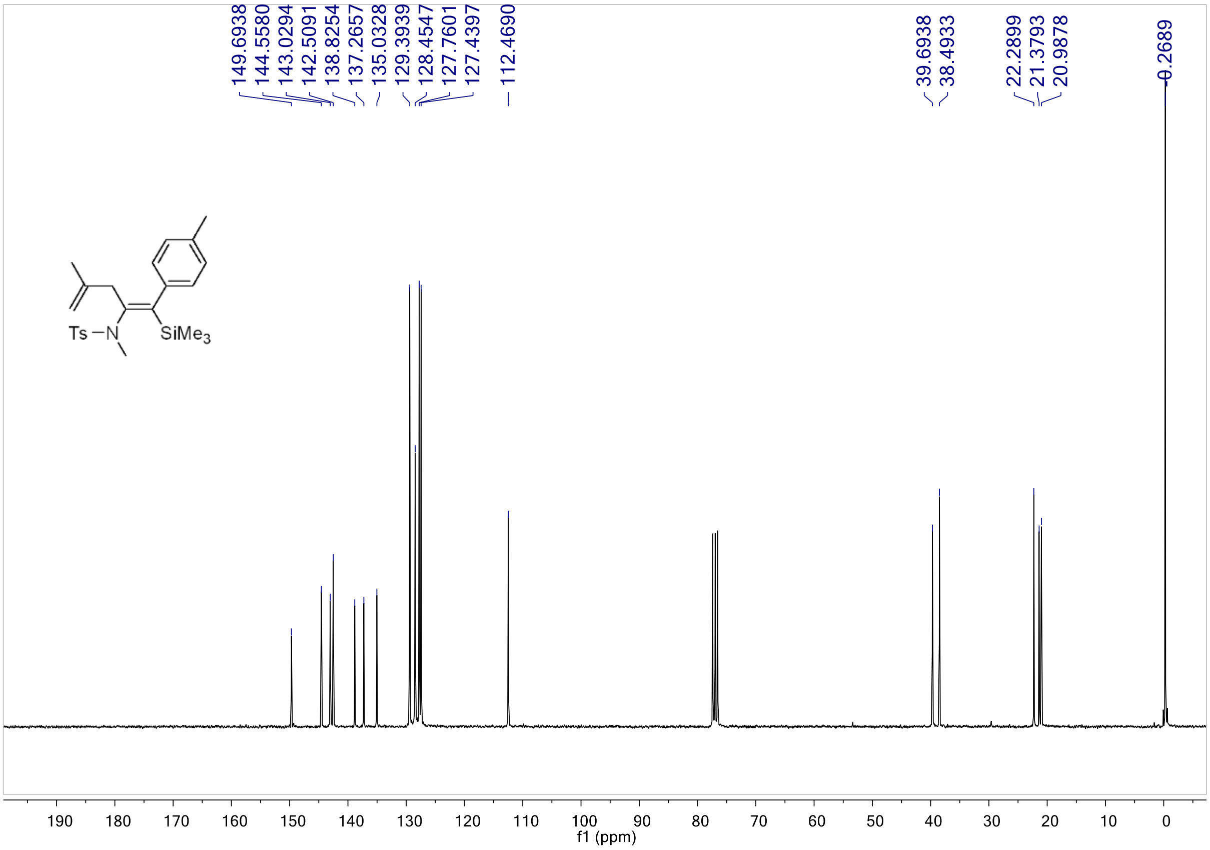

^{29}Si NMR (75 MHz, CDCl$_3$) spectrum of 3m

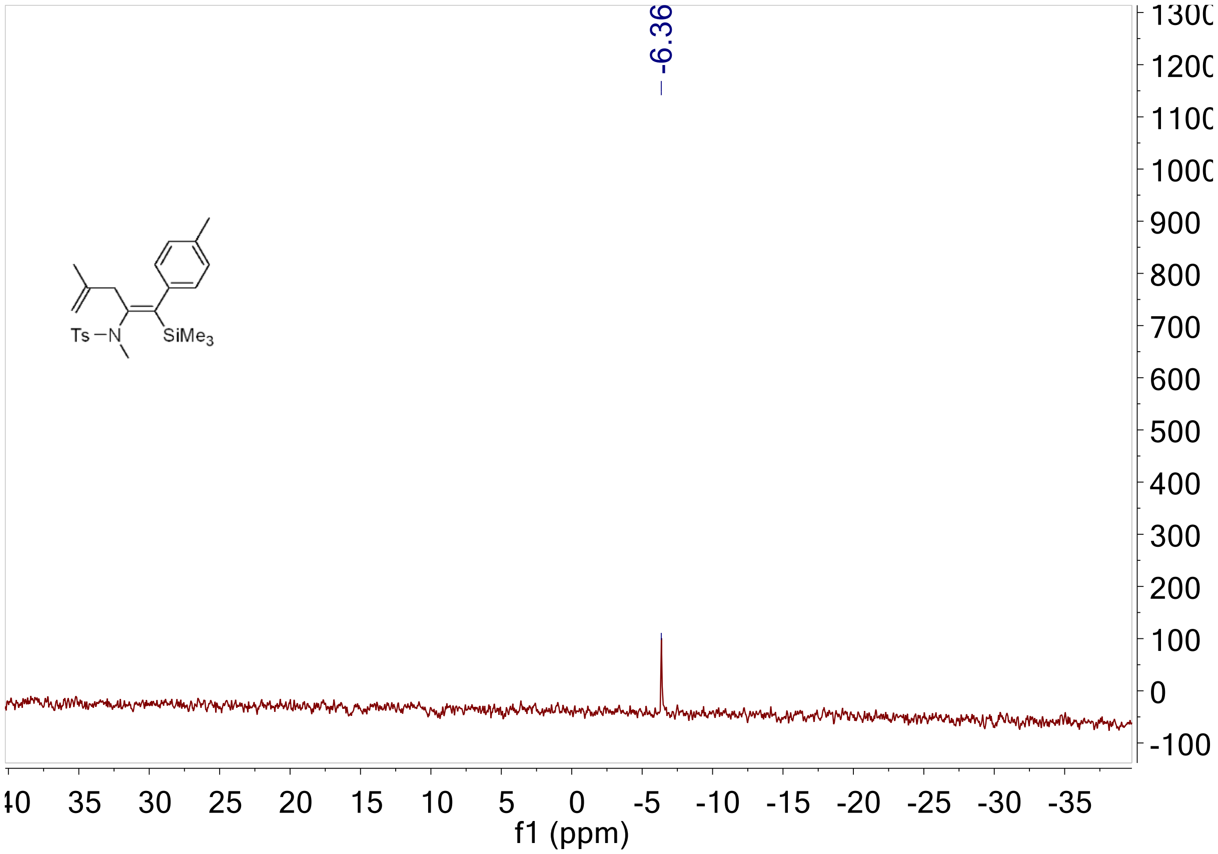

^{1}H NMR (300 MHz, CDCl$_3$) spectrum of 3n

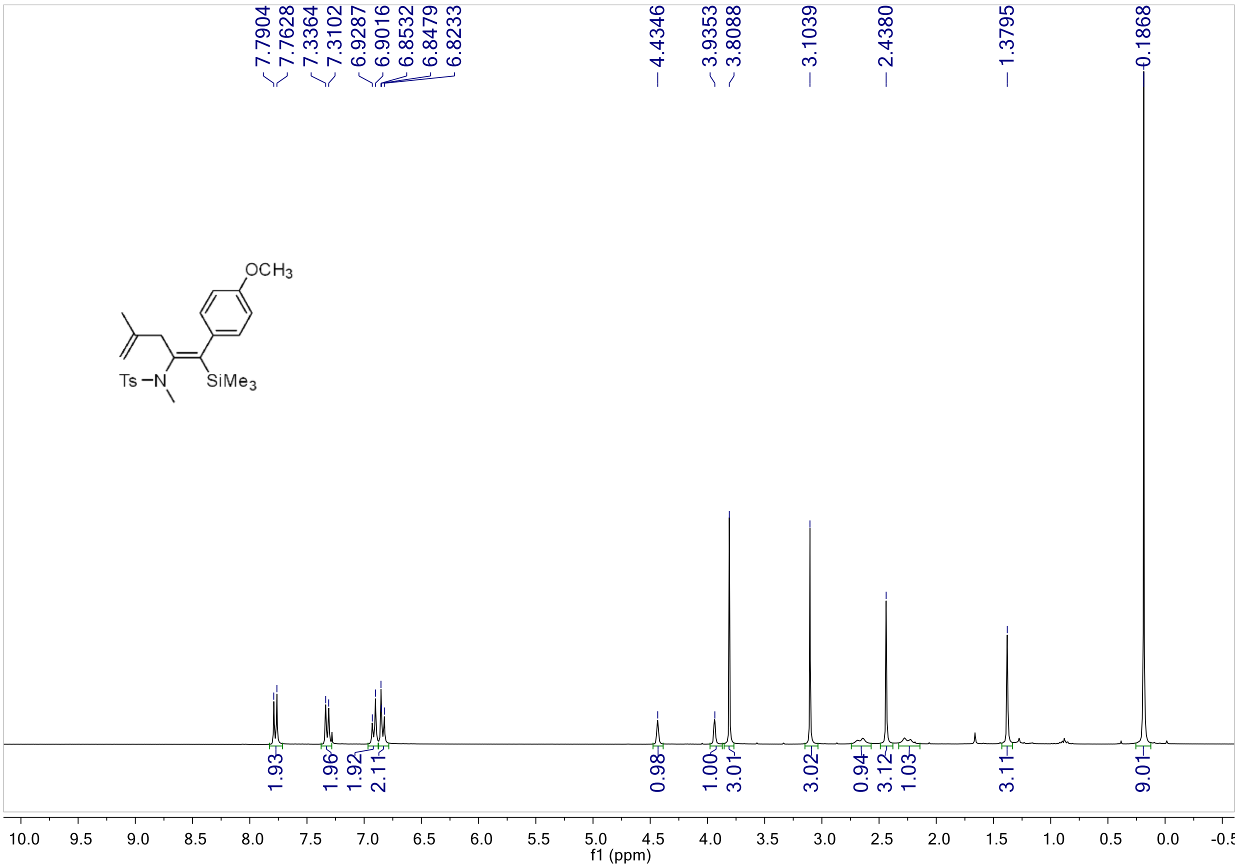

^{13}C NMR (75 MHz, $CDCl_3$) spectrum of 3n

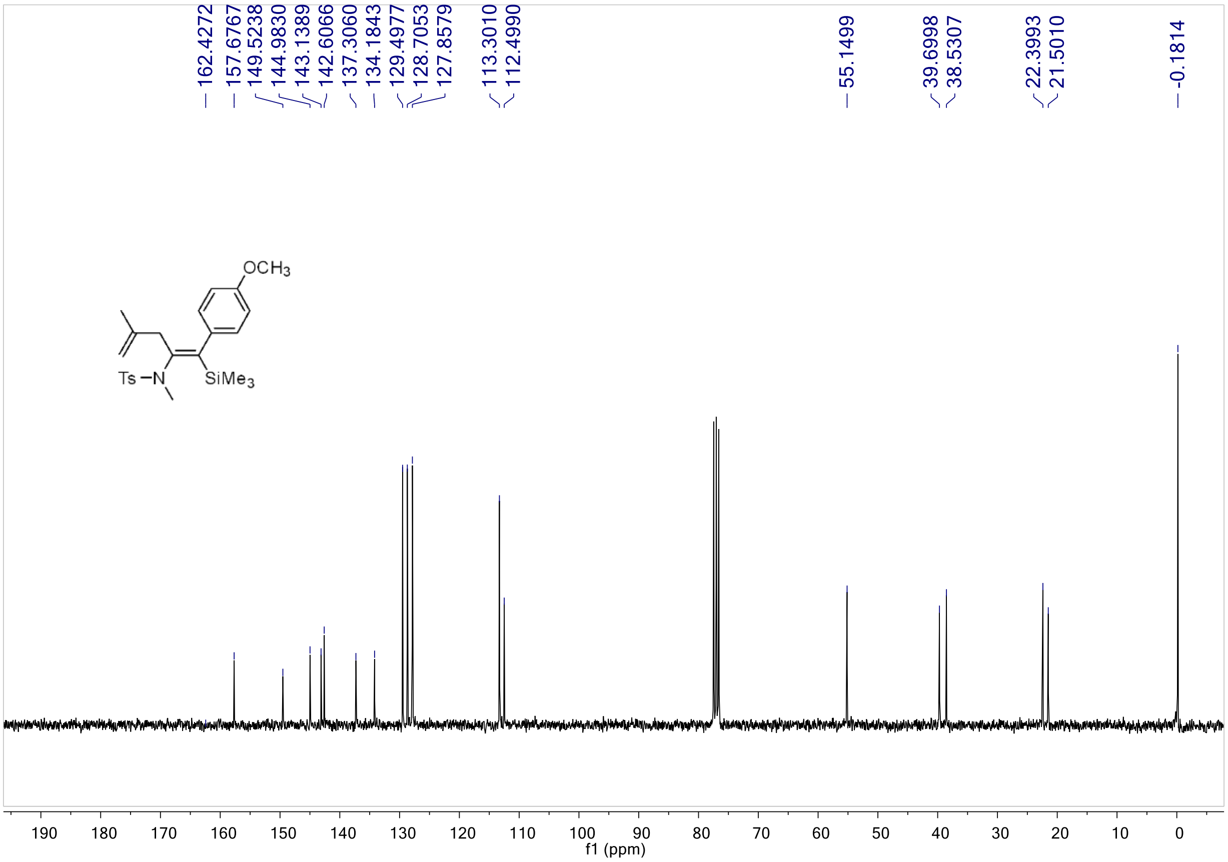

^{29}Si NMR (75 MHz, $CDCl_3$) spectrum of 3n

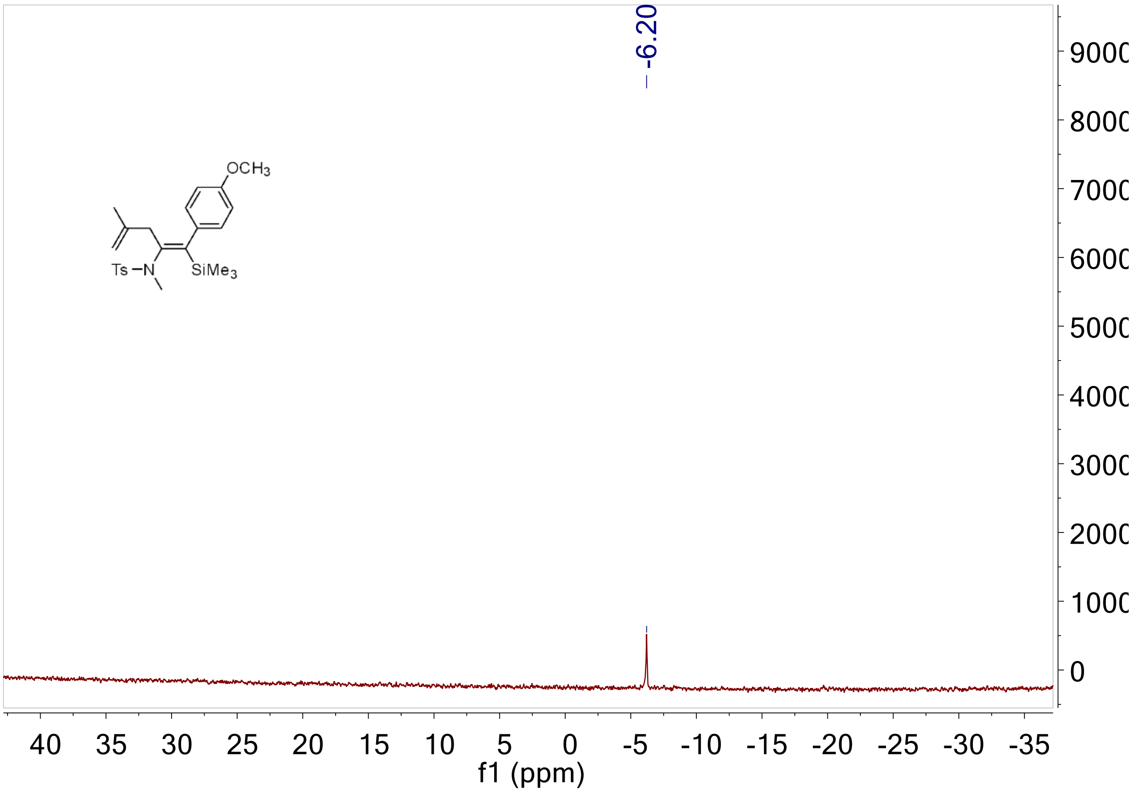

^{1}H NMR (300 MHz, $CDCl_3$) spectrum of 3o

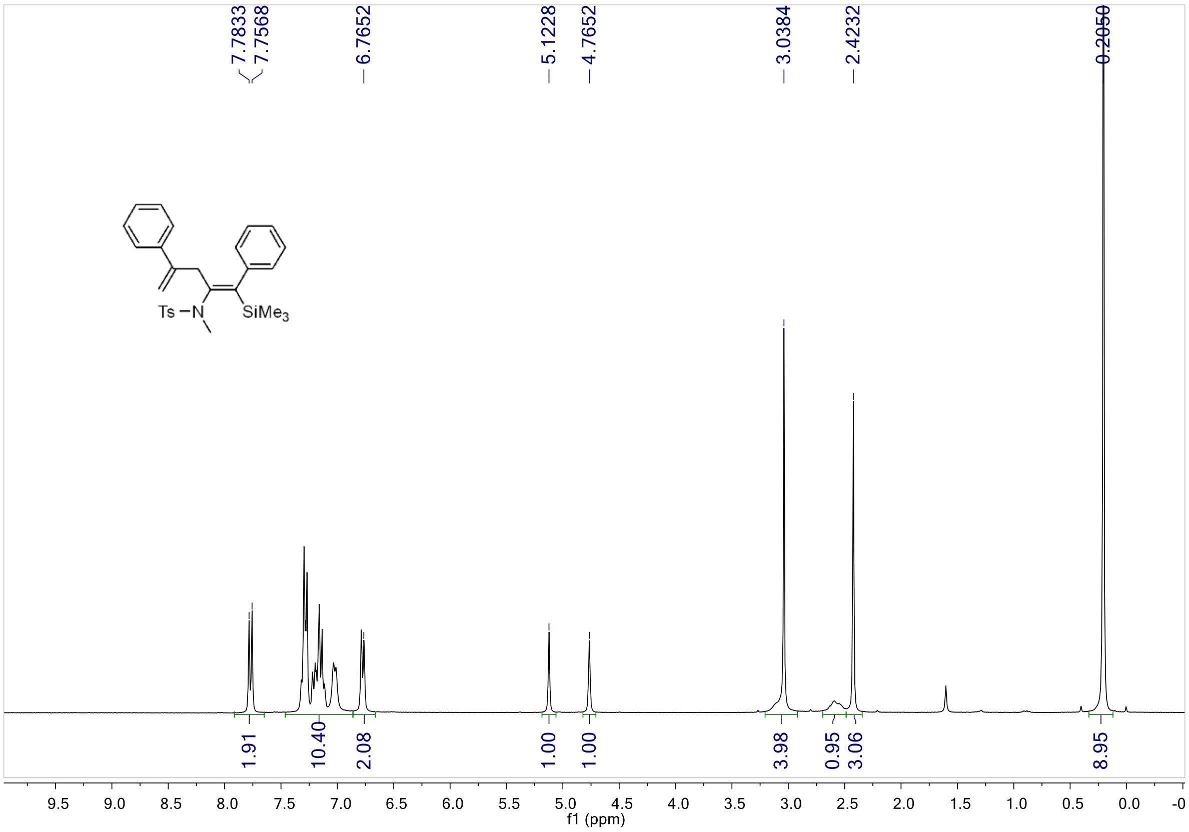

^{13}C NMR (75 MHz, $CDCl_3$) spectrum of 3o

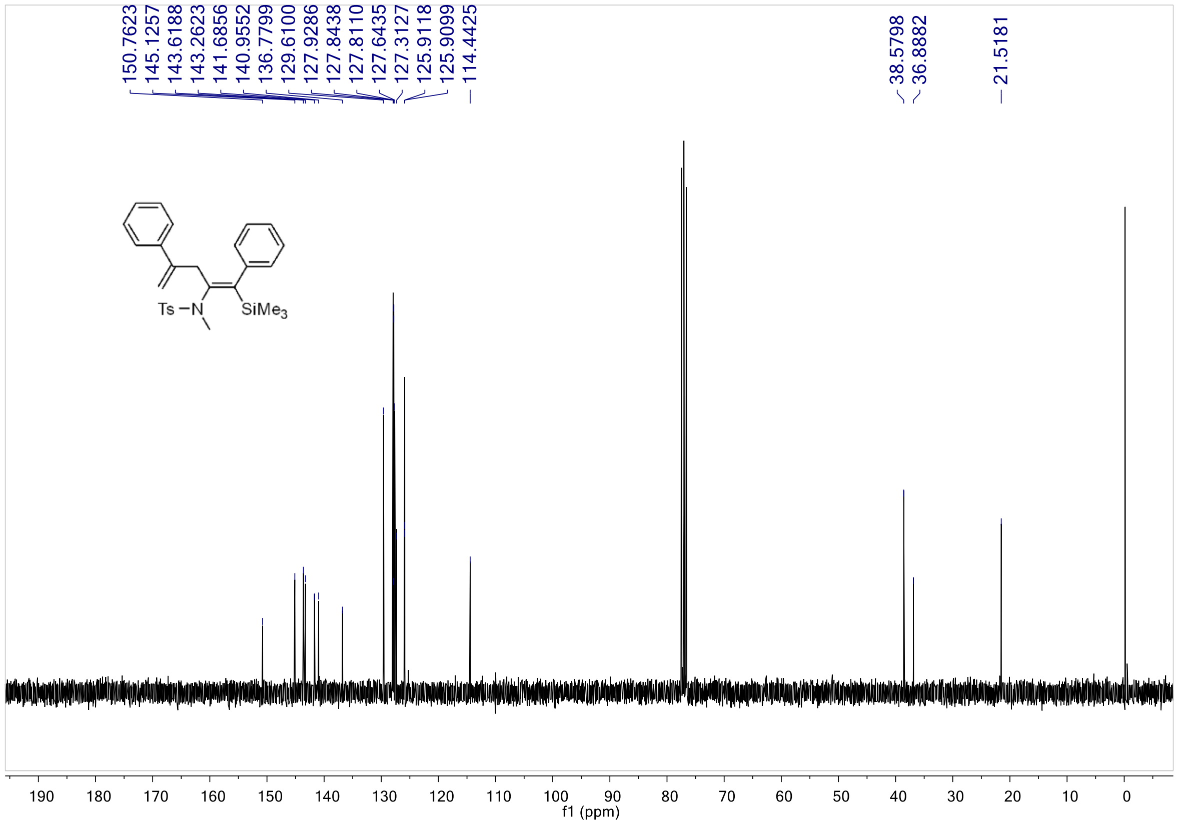

^{29}Si NMR (75 MHz, CDCl$_3$) spectrum of 3o

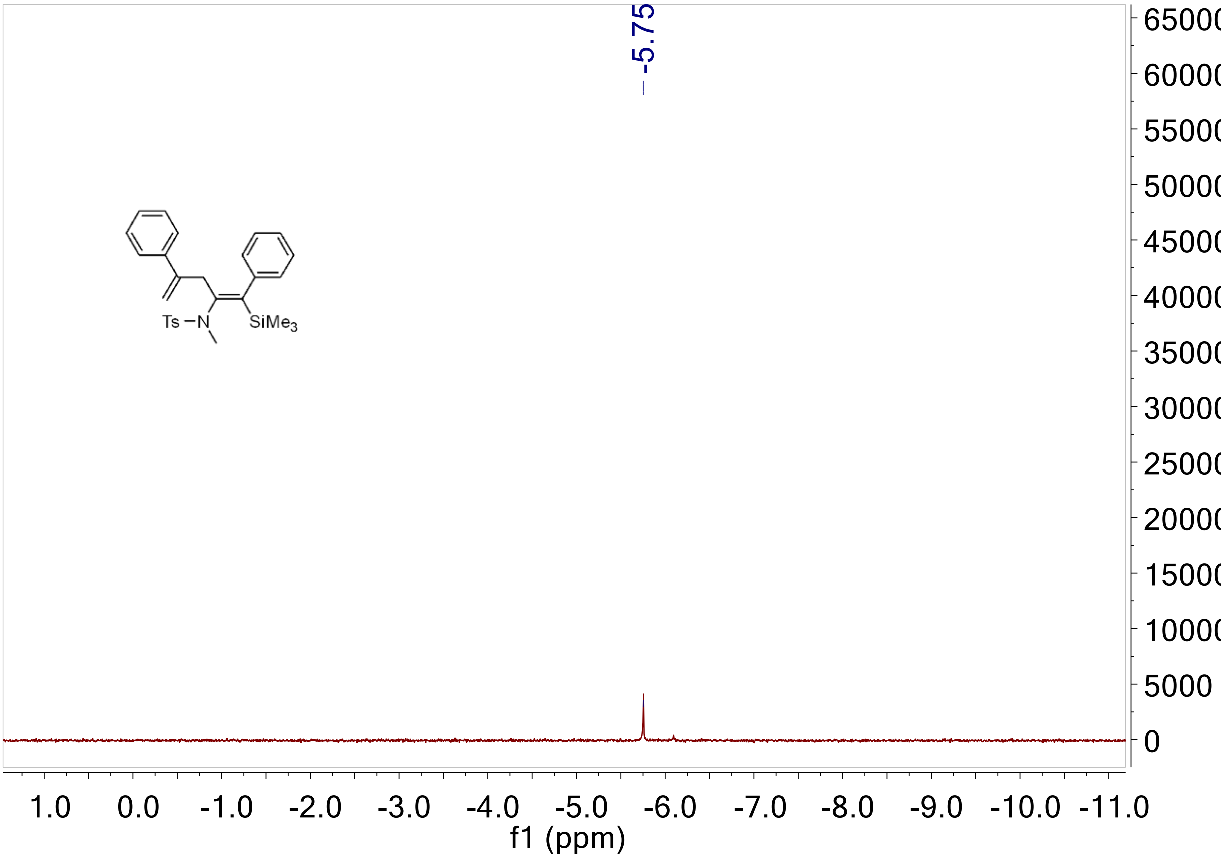

^{1}H NMR (300 MHz, CDCl$_3$) spectrum of 3p

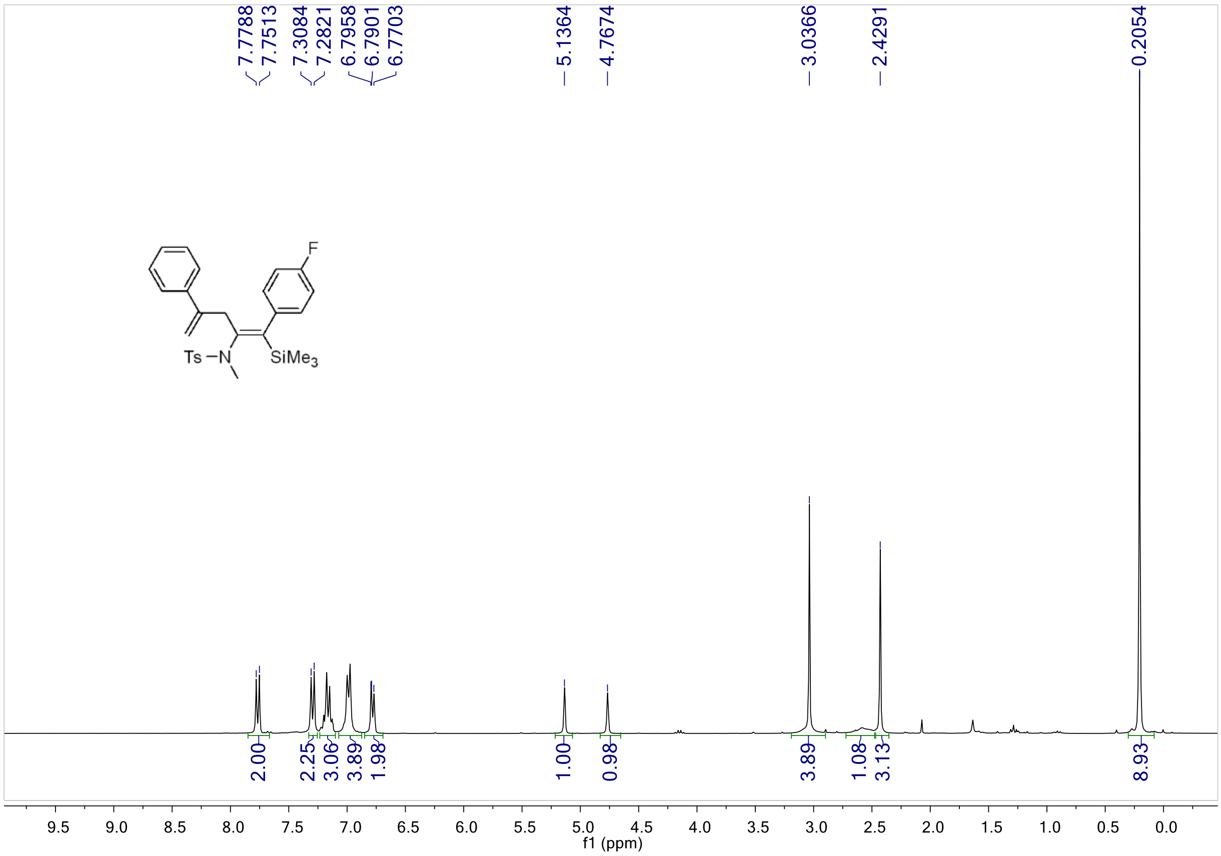

^{13}C NMR (75 MHz, CDCl3) spectrum of 3p

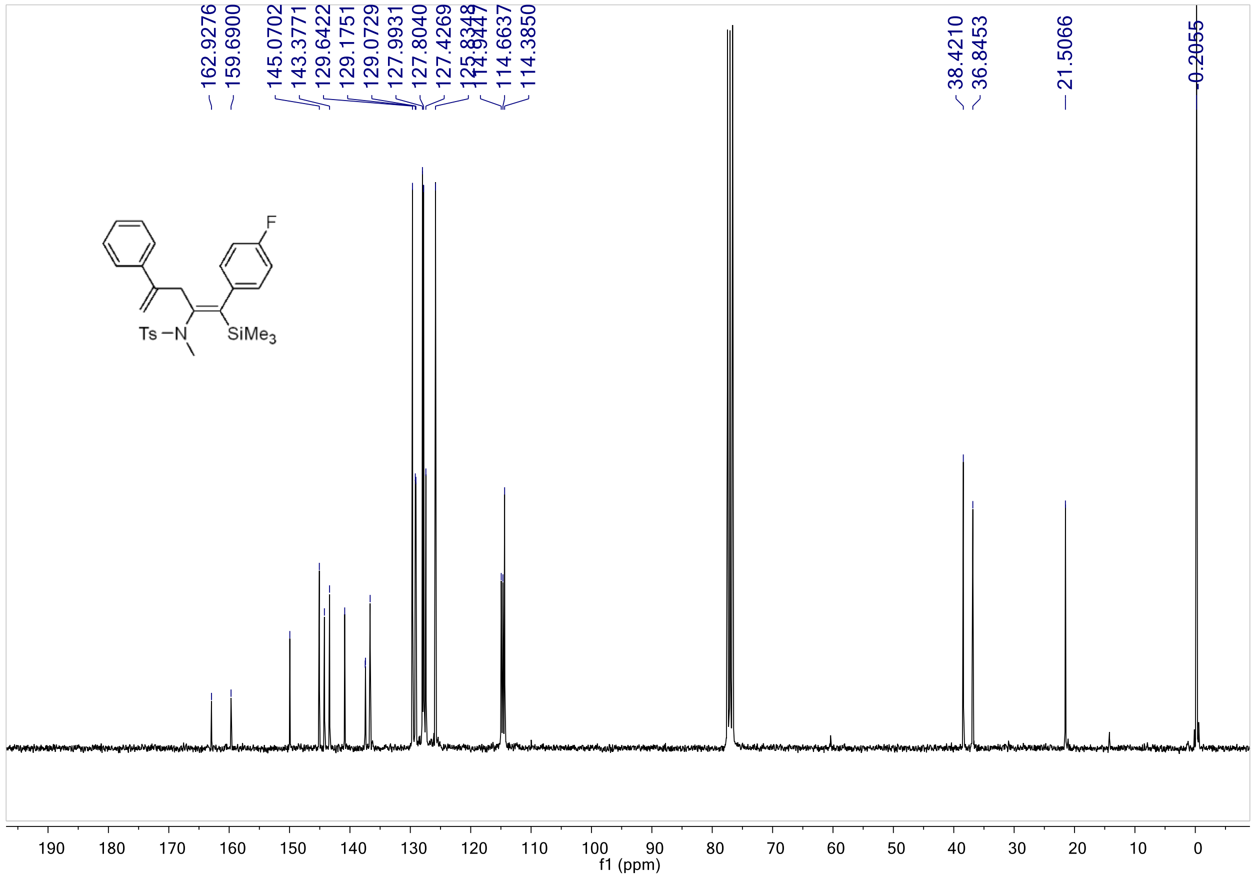

[28]Si NMR (75 MHz, CDCl3) spectrum of 3p

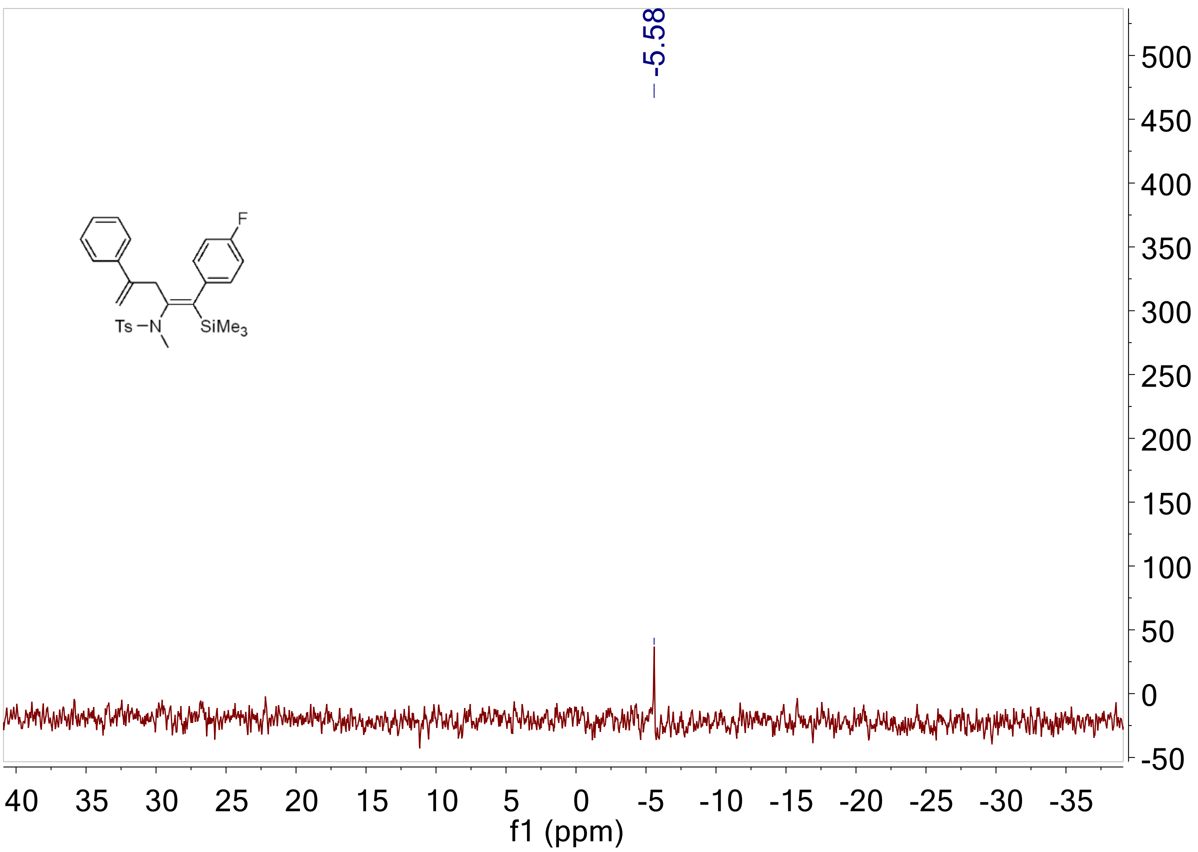

^{19}F NMR (282 MHz, $CDCl_3$) spectrum of 3p

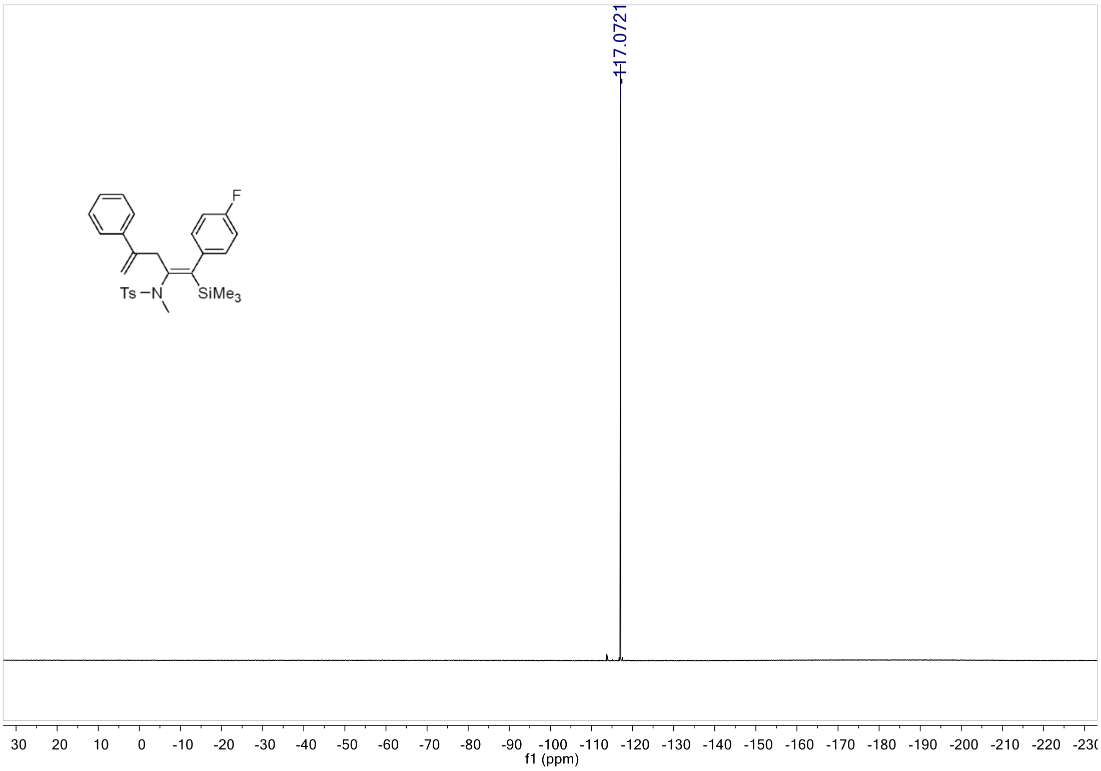

^{1}H NMR (300 MHz, $CDCl_3$) spectrum of 3q

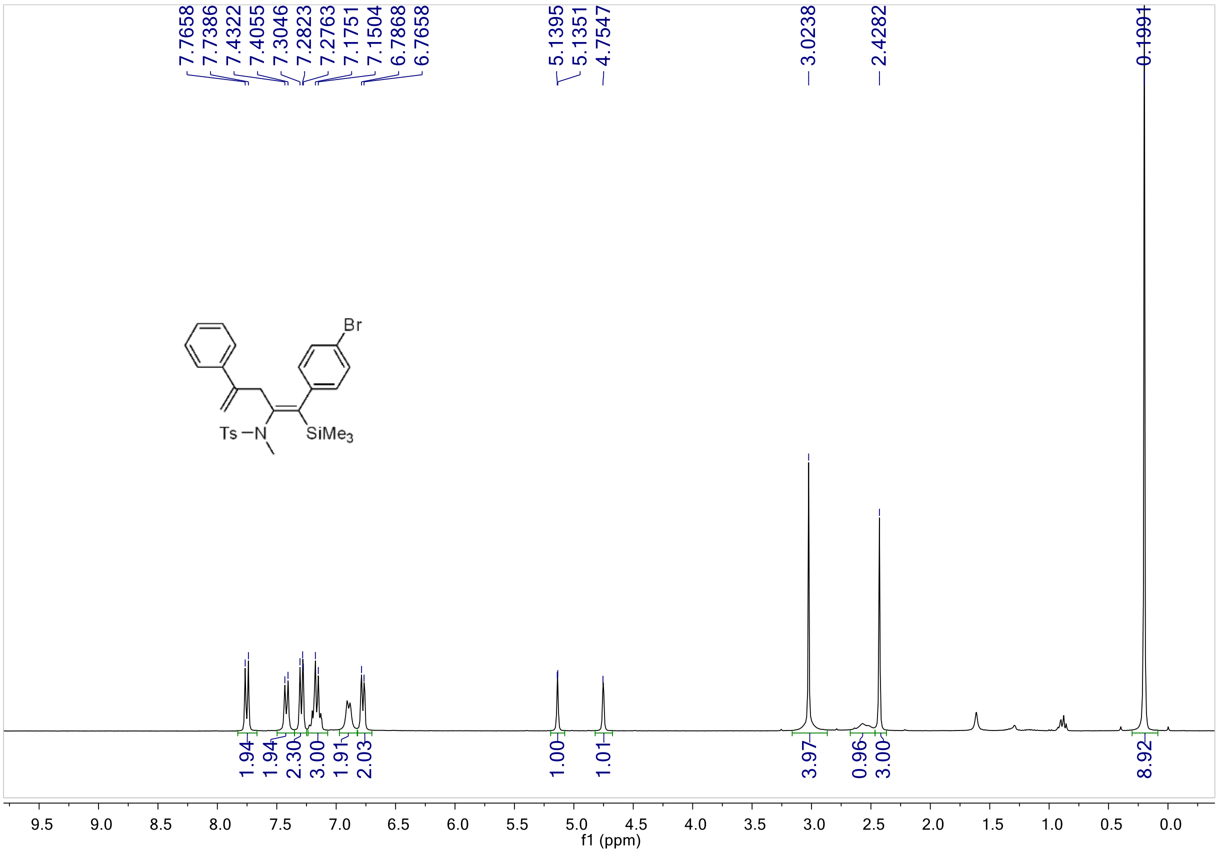

^{13}C NMR (75 MHz, $CDCl_3$) spectrum of 3q

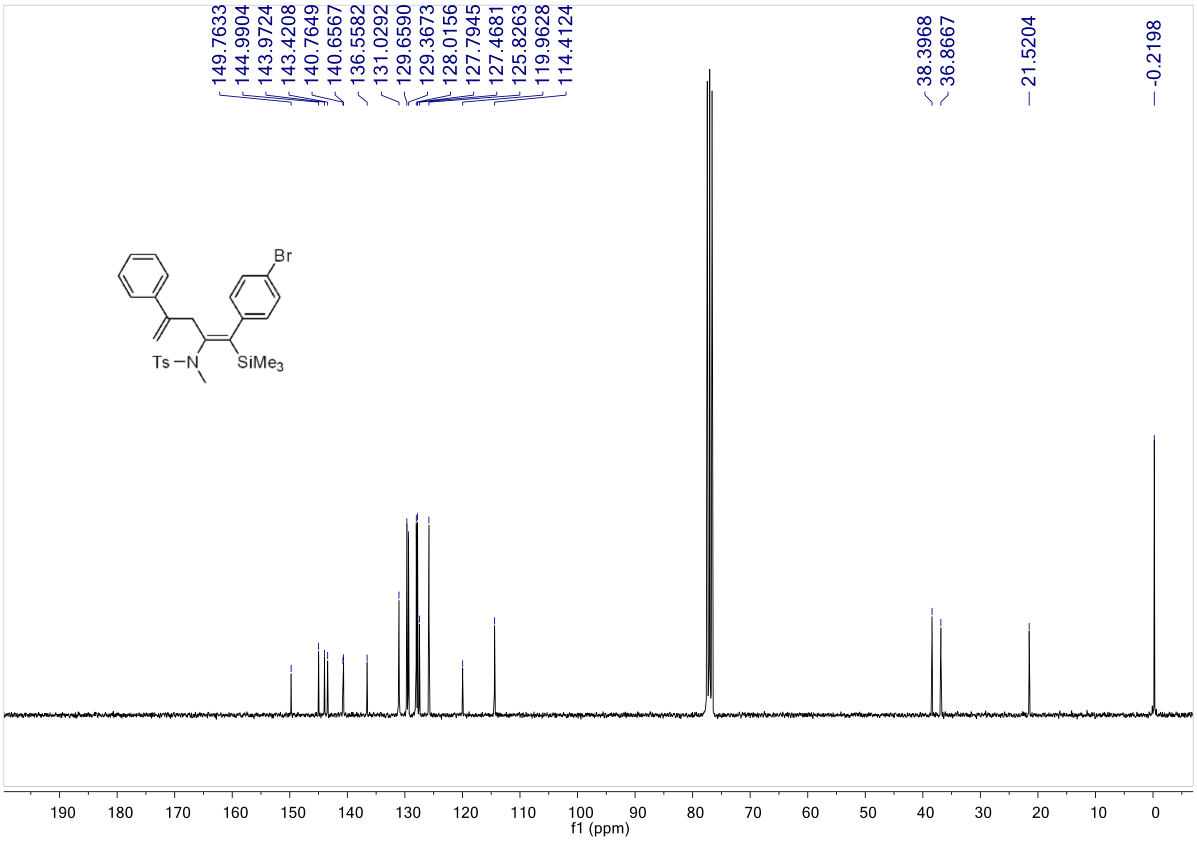

^{29}Si NMR (75 MHz, $CDCl_3$) spectrum of 3q

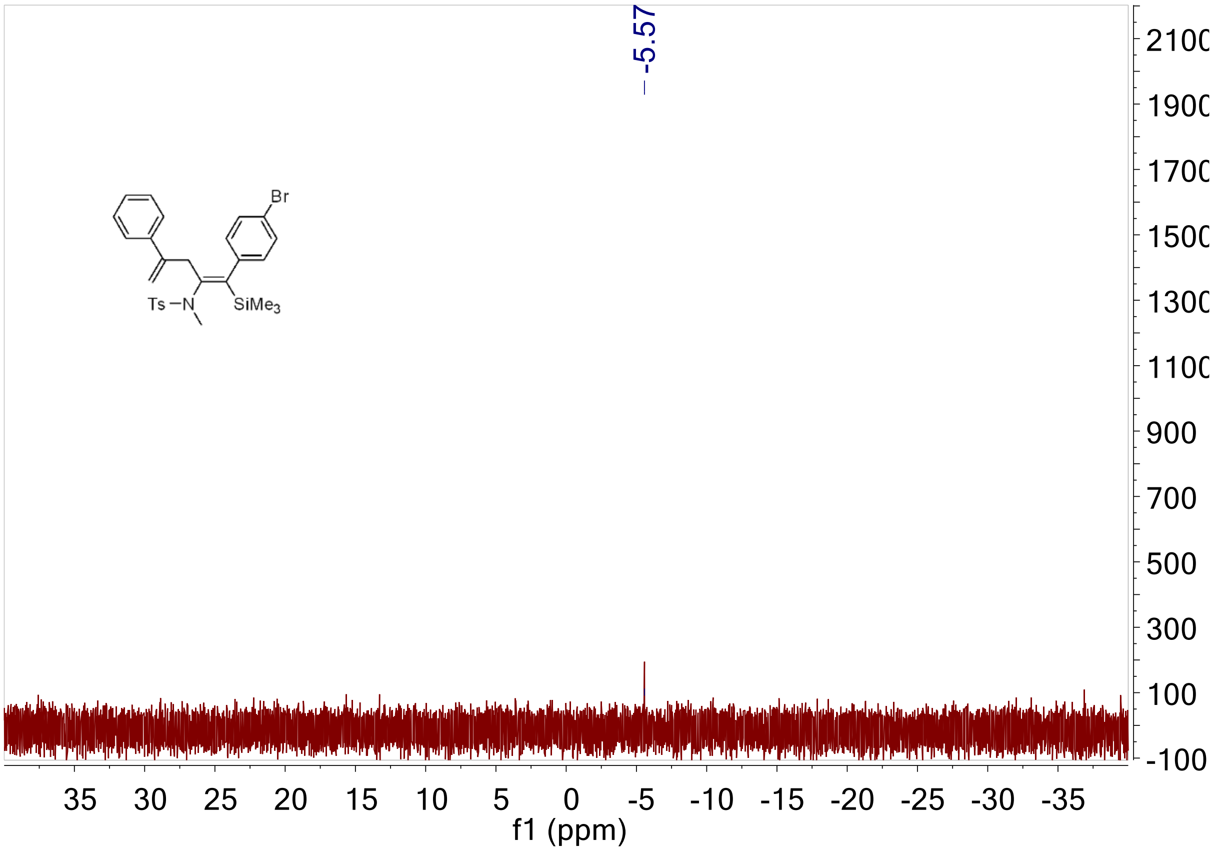

^{1}H NMR (300 MHz, $CDCl_3$) spectrum of 3r

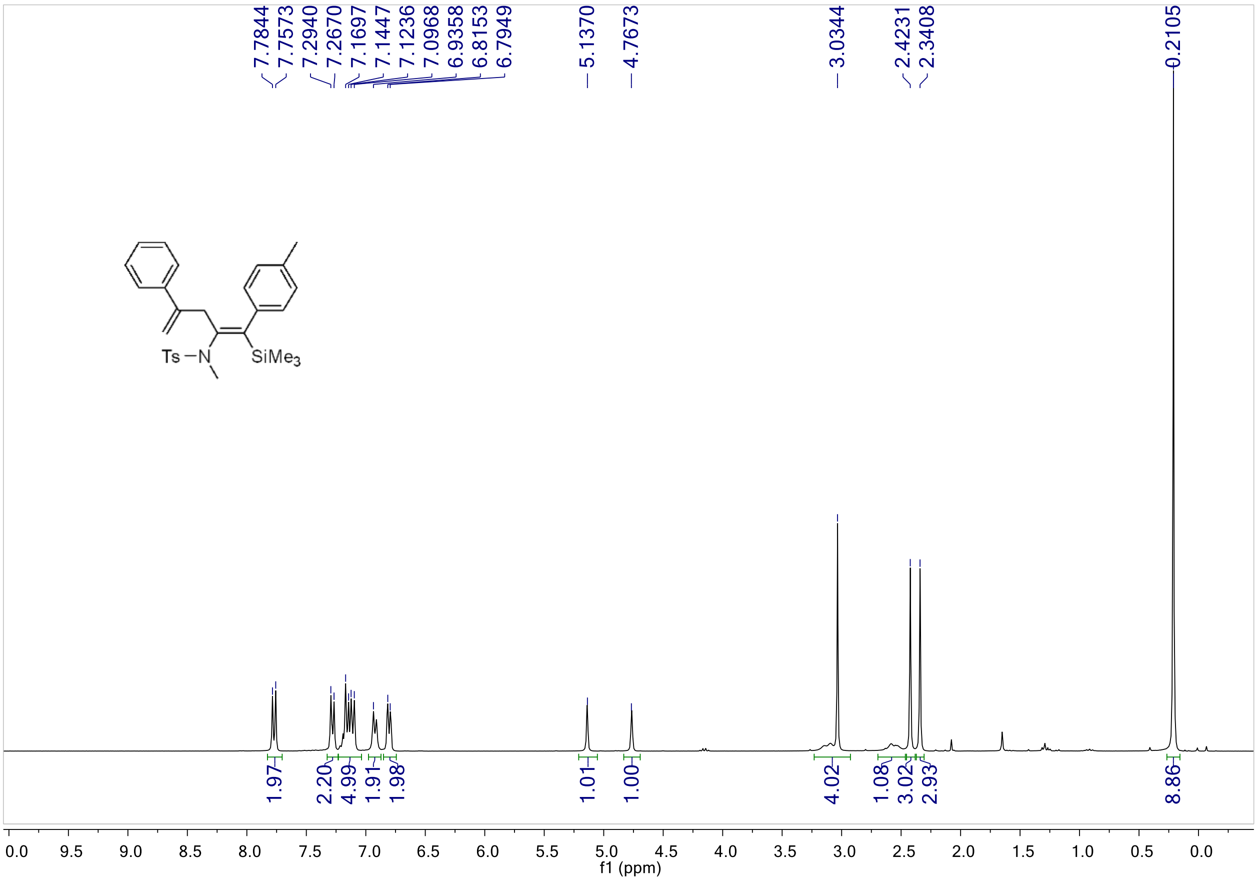

^{13}C NMR (75 MHz, $CDCl_3$) spectrum of 3r

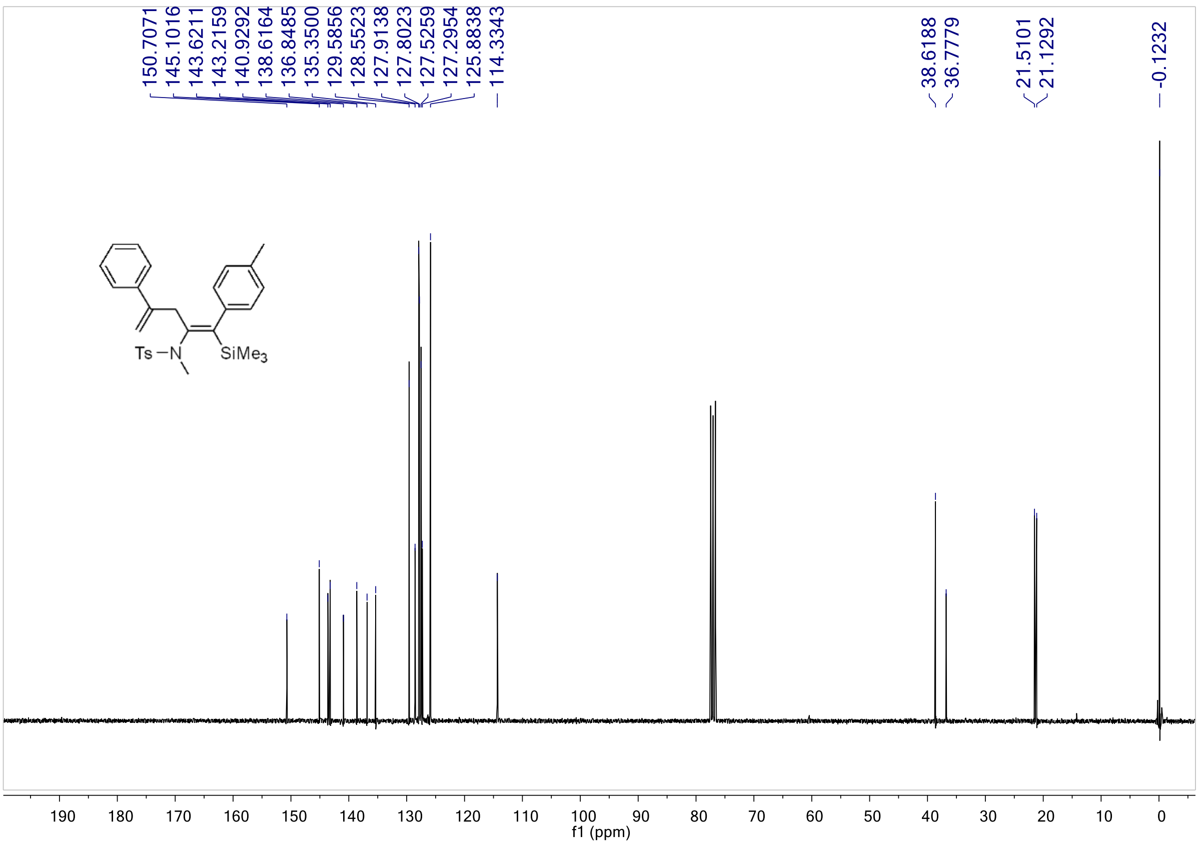

^{29}Si NMR (75 MHz, $CDCl_3$) spectrum of 3r

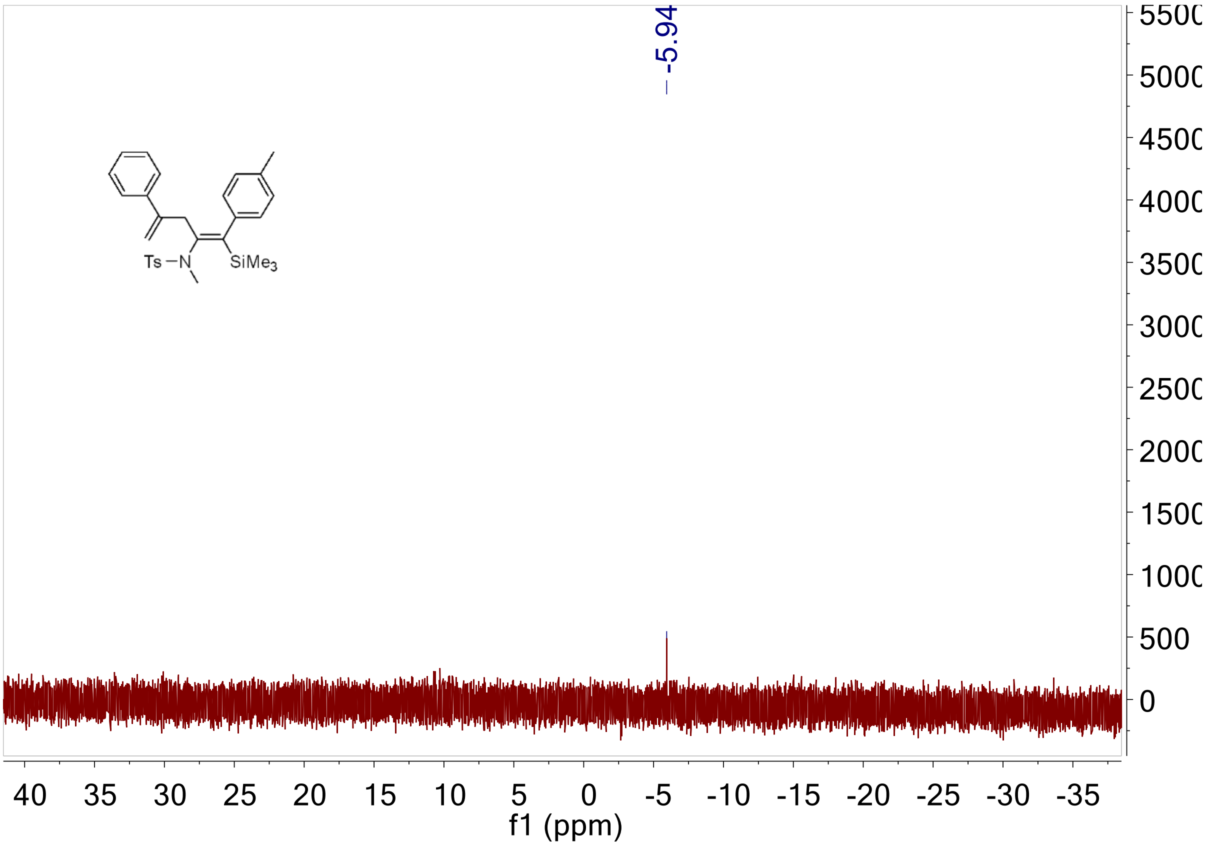

^{1}H NMR (300 MHz, $CDCl_3$) spectrum of 3s

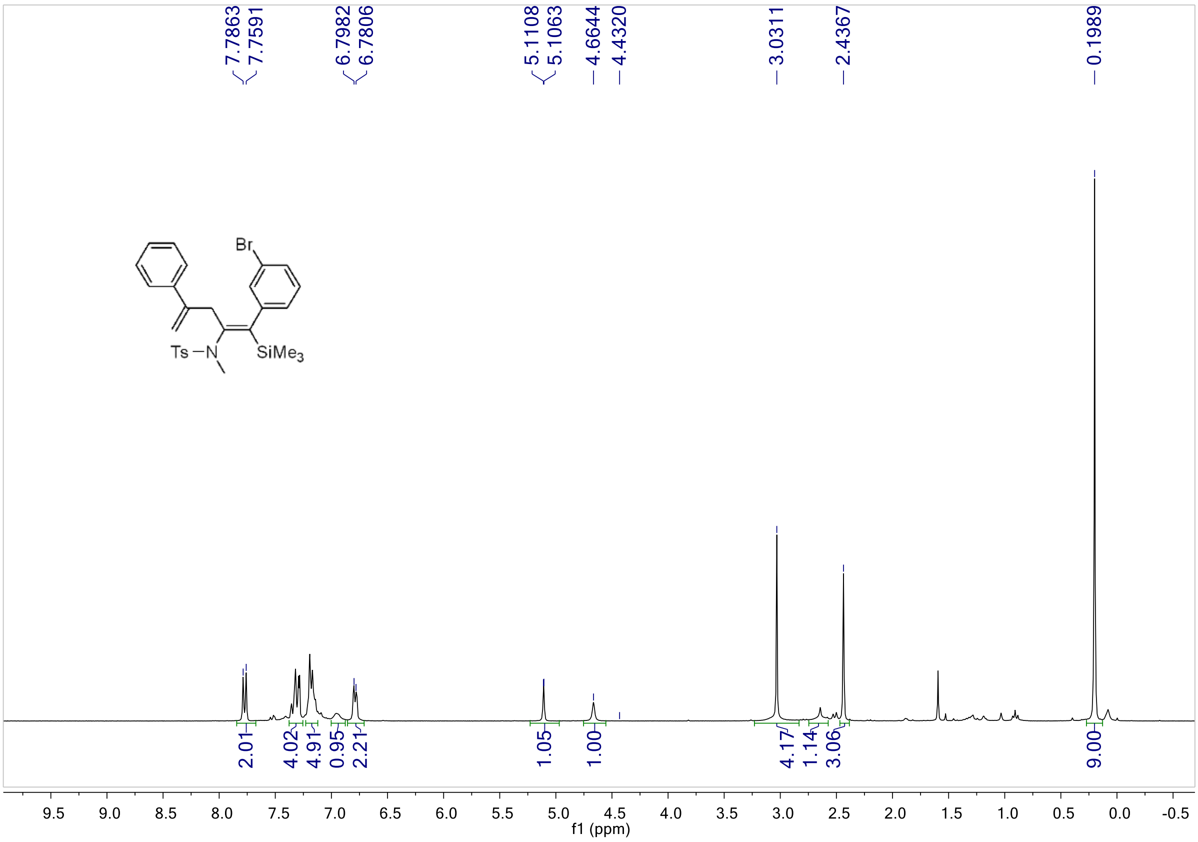

^{13}C NMR (75 MHz, $CDCl_3$) spectrum of 3s

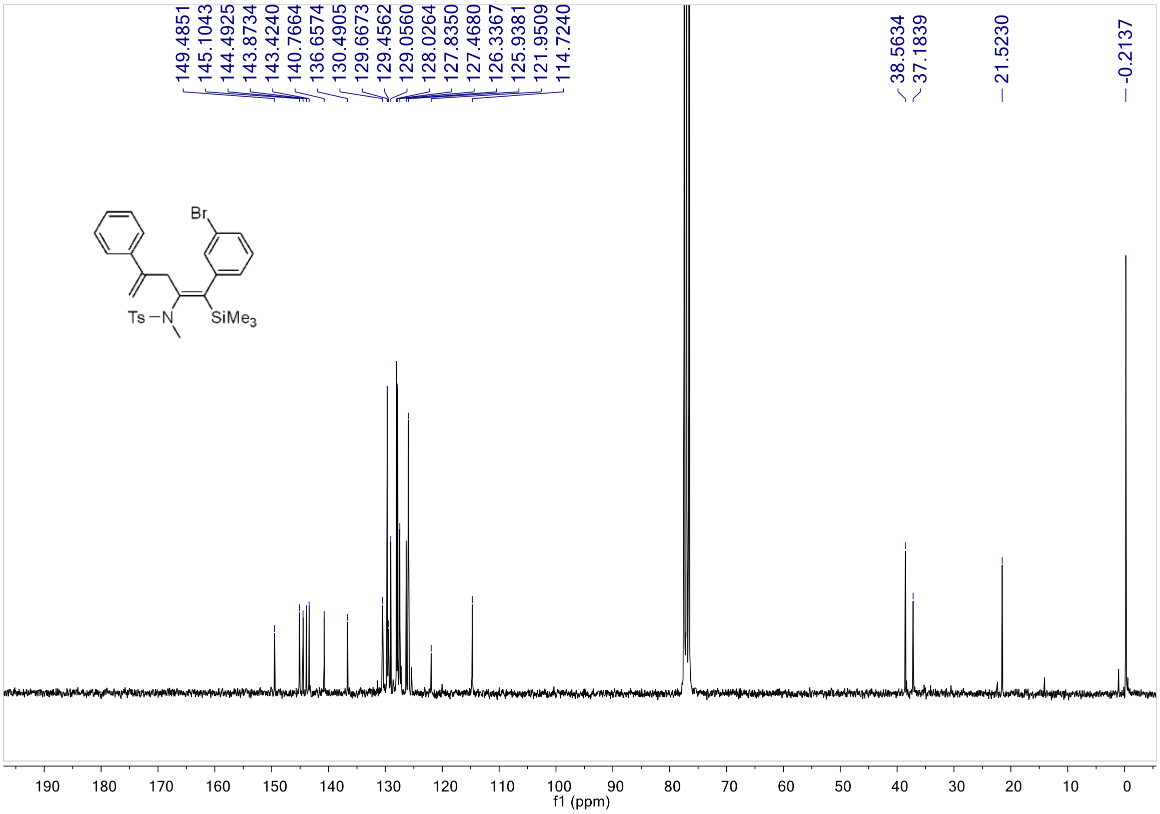

^{29}Si NMR (75 MHz, $CDCl_3$) spectrum of 3s

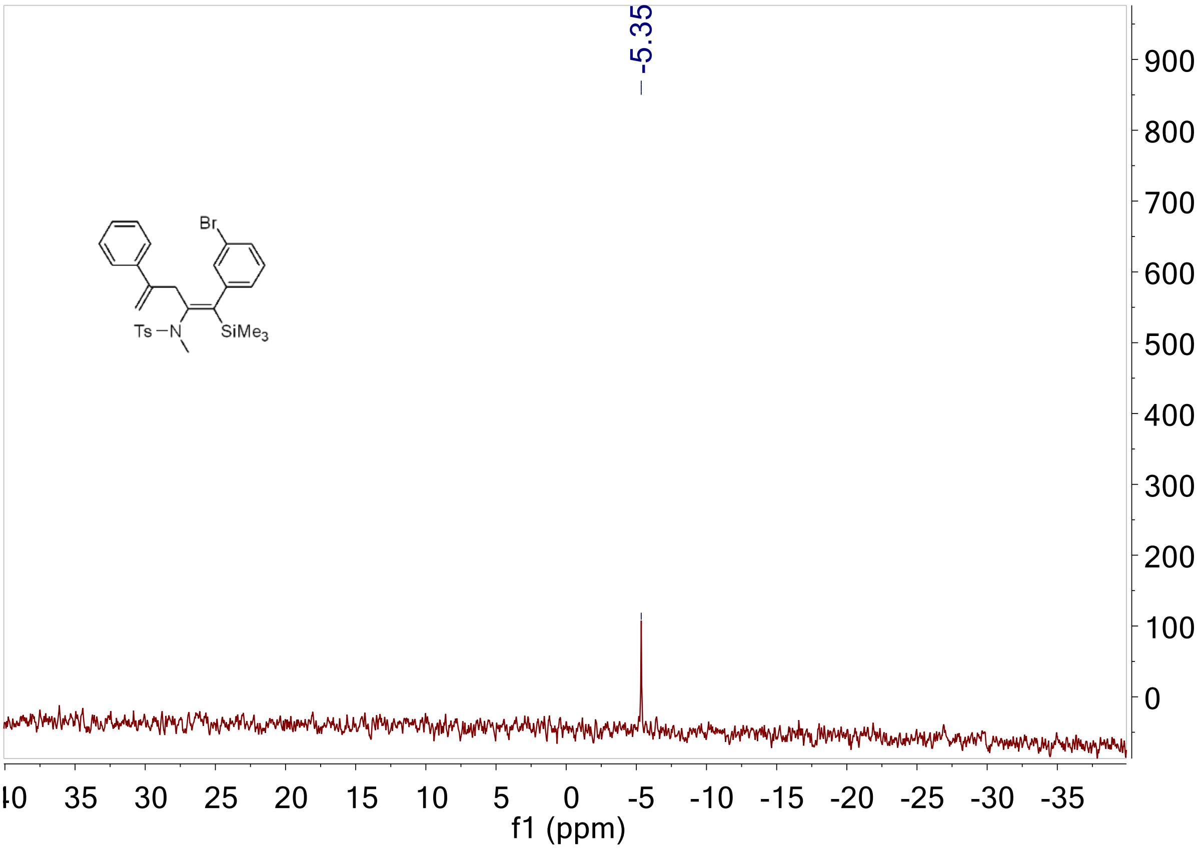

^{1}H NMR (300 MHz, $CDCl_3$) spectrum of 3t

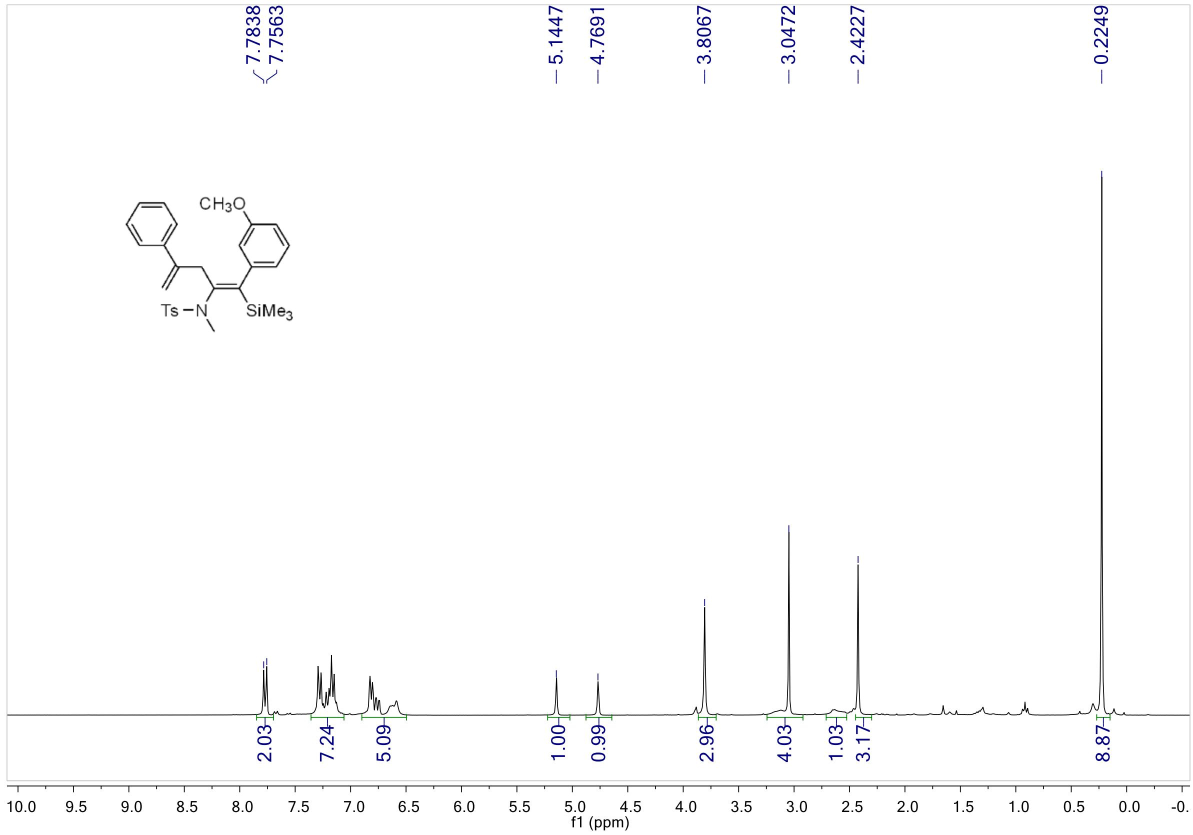

^{13}C NMR (75 MHz, $CDCl_3$) spectrum of 3t

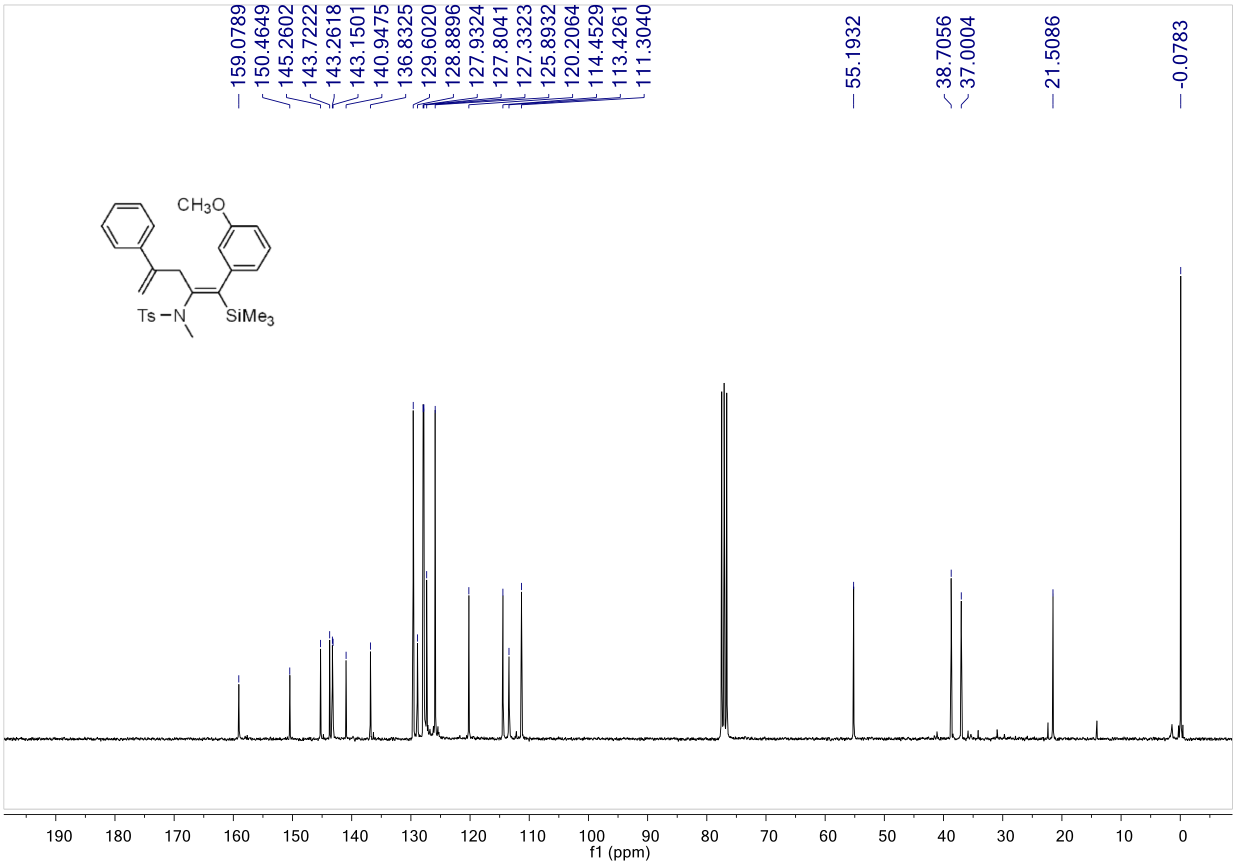

^{29}Si NMR (75 MHz, $CDCl_3$) spectrum of 3t

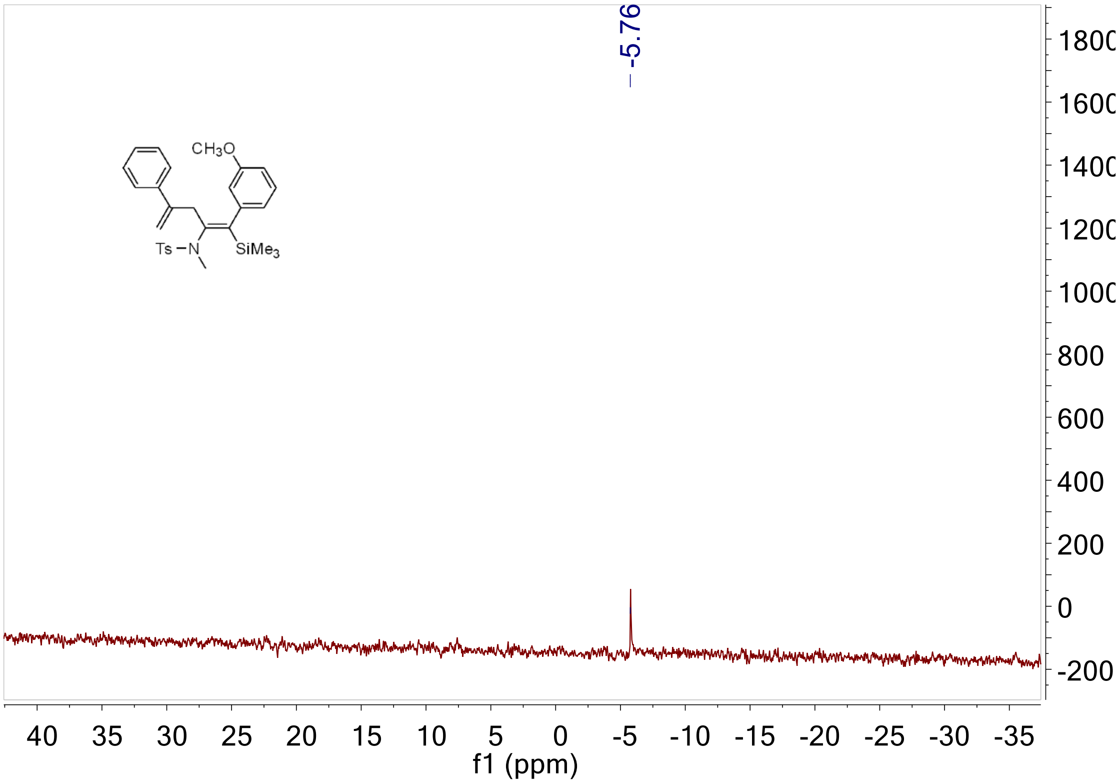

^{1}H NMR (300 MHz, DMSO) spectrum of 3u

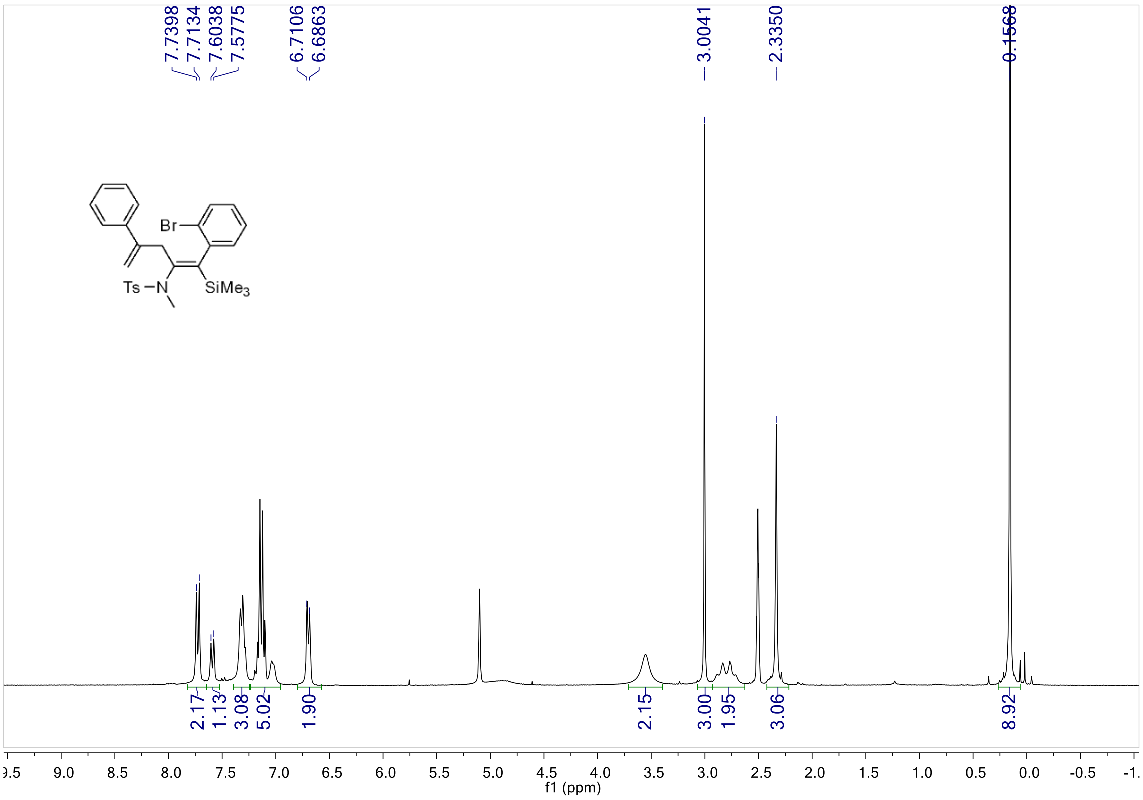

^{13}C NMR (75 MHz, DMSO, 383 K) spectrum of 3u

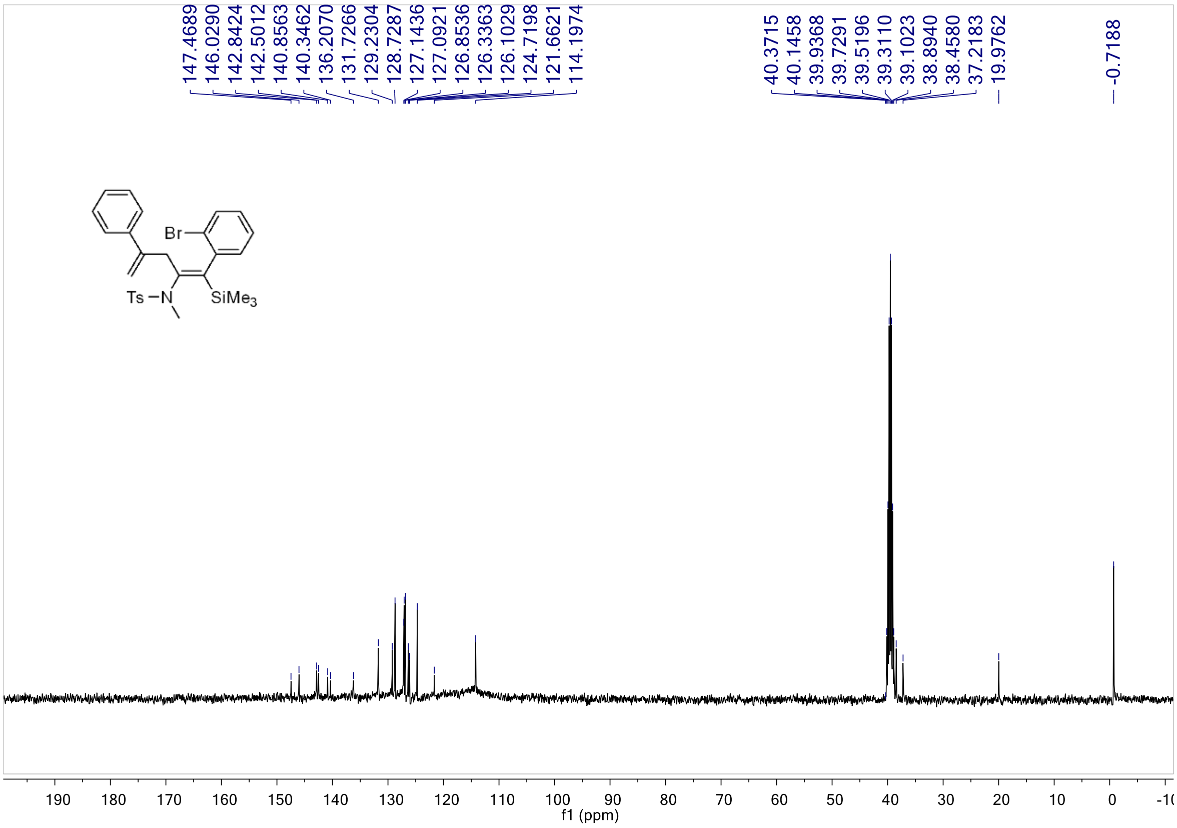

^{29}Si NMR (75 MHz, $CDCl_3$) spectrum of 3u

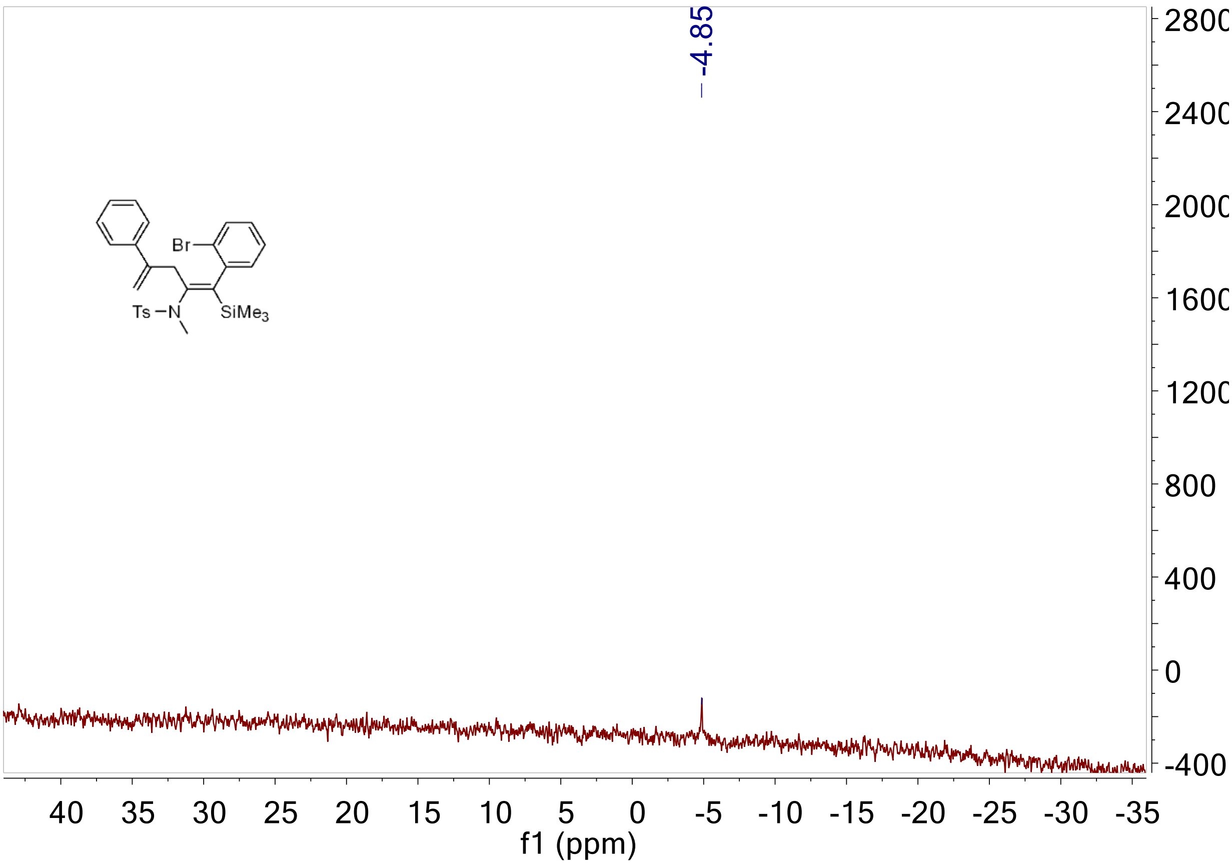

^{1}H NMR (300 MHz, $CDCl_3$) spectrum of 3v

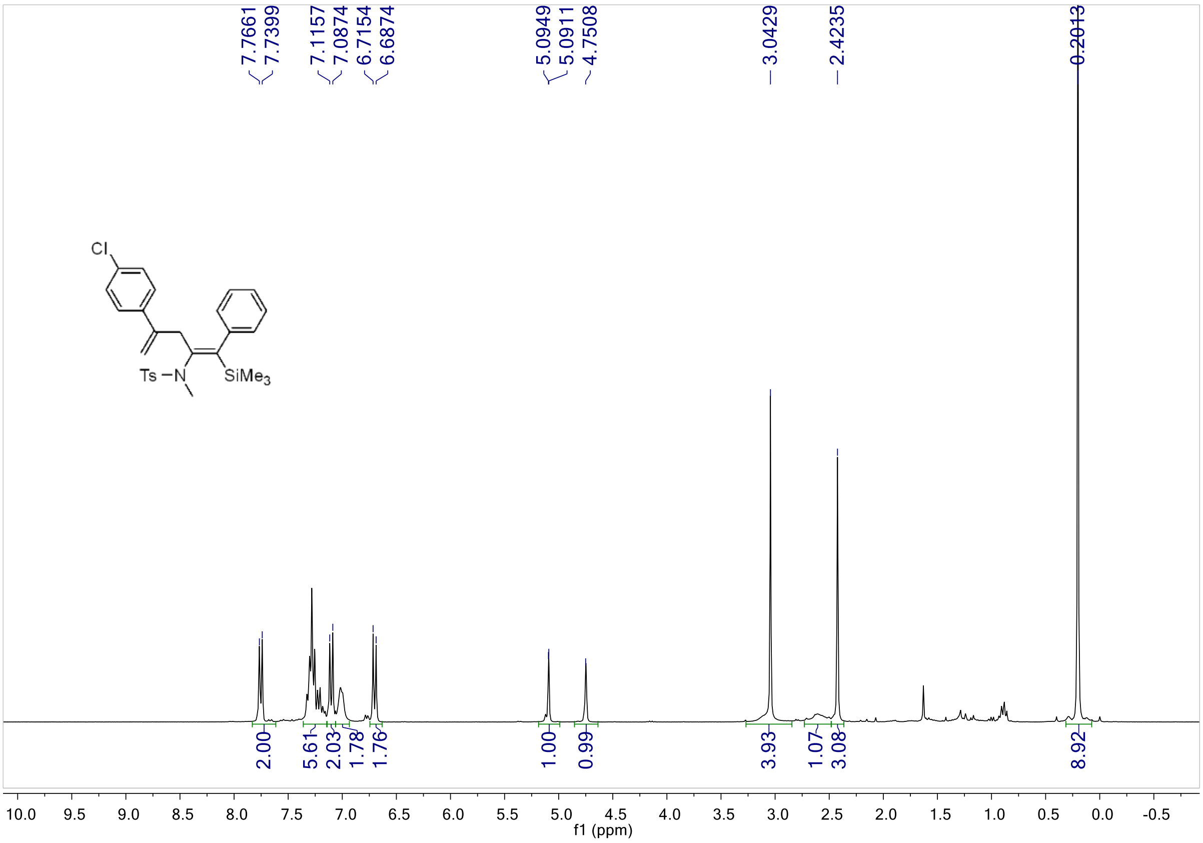

^{13}C NMR (75 MHz, $CDCl_3$) spectrum of 3v

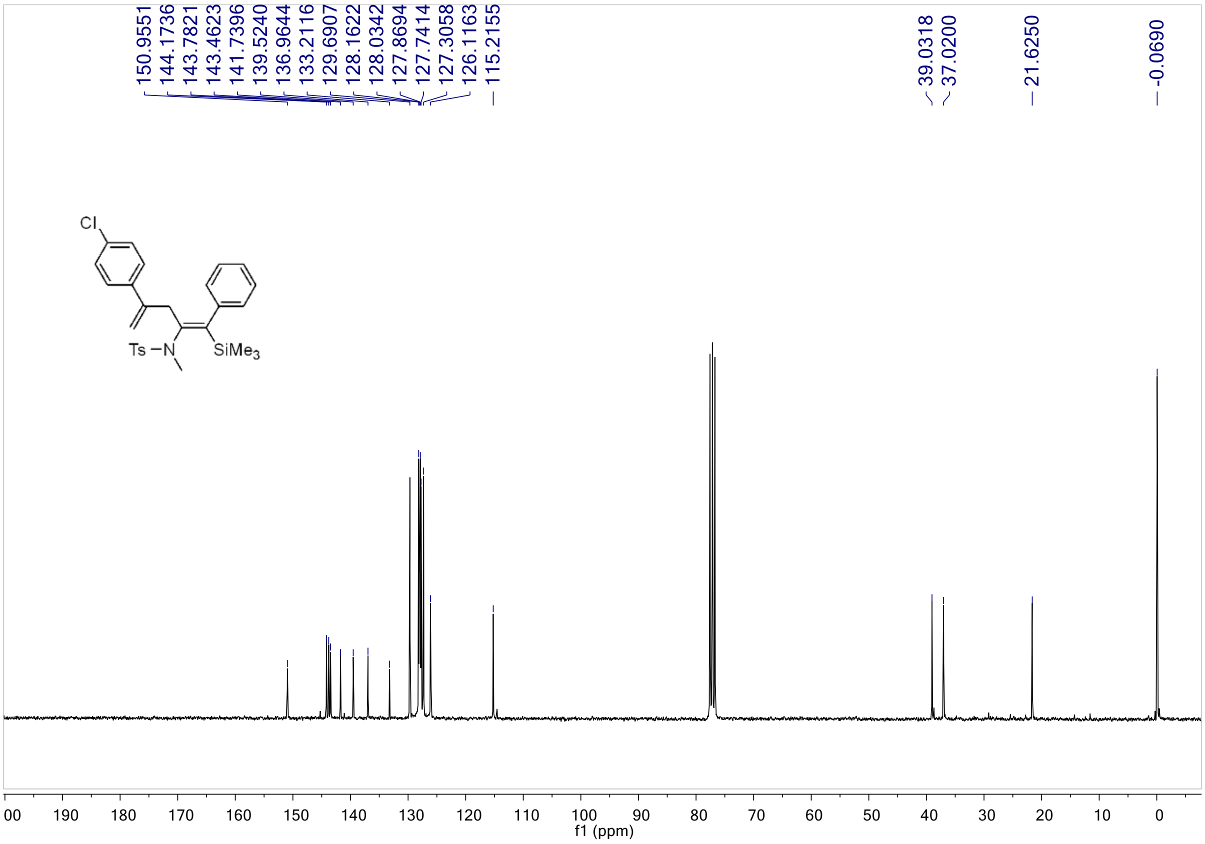

^{28}Si NMR (75 MHz, $CDCl_3$) spectrum of 3v

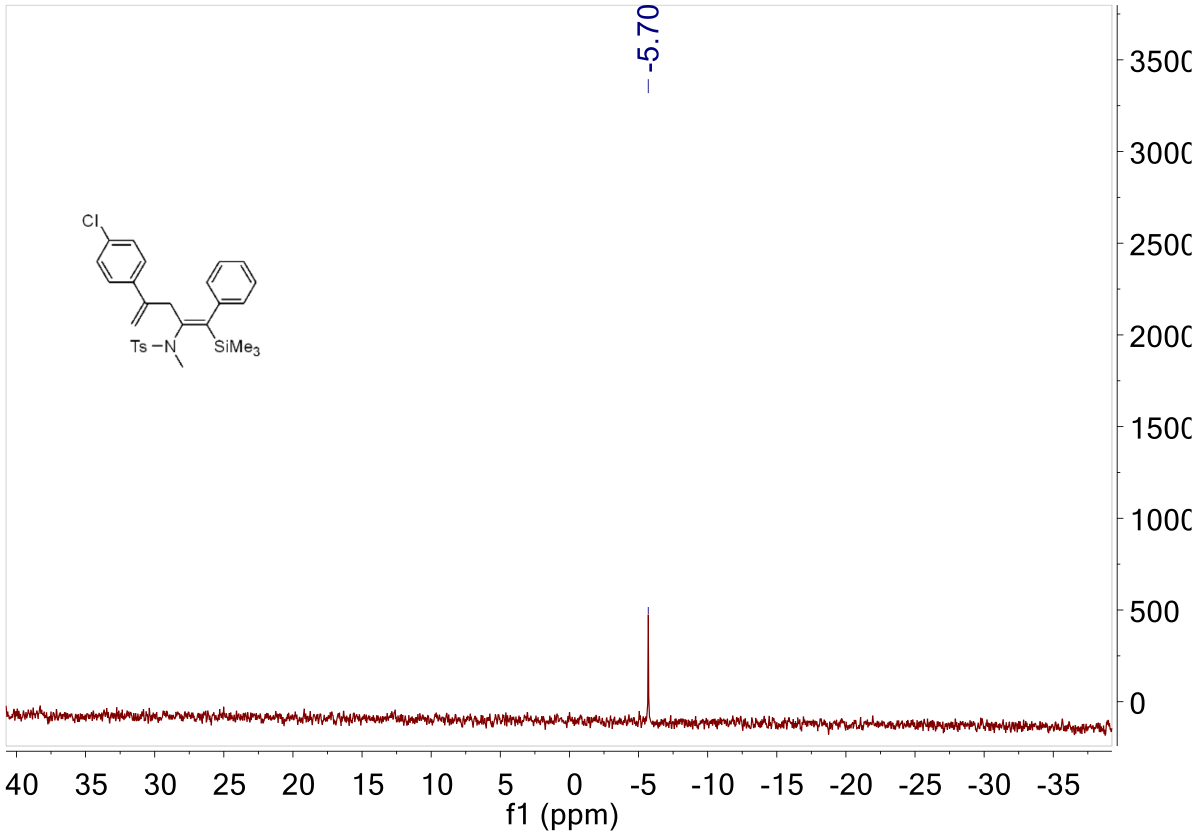

^{1}H NMR (300 MHz, $CDCl_3$) spectrum of 3w

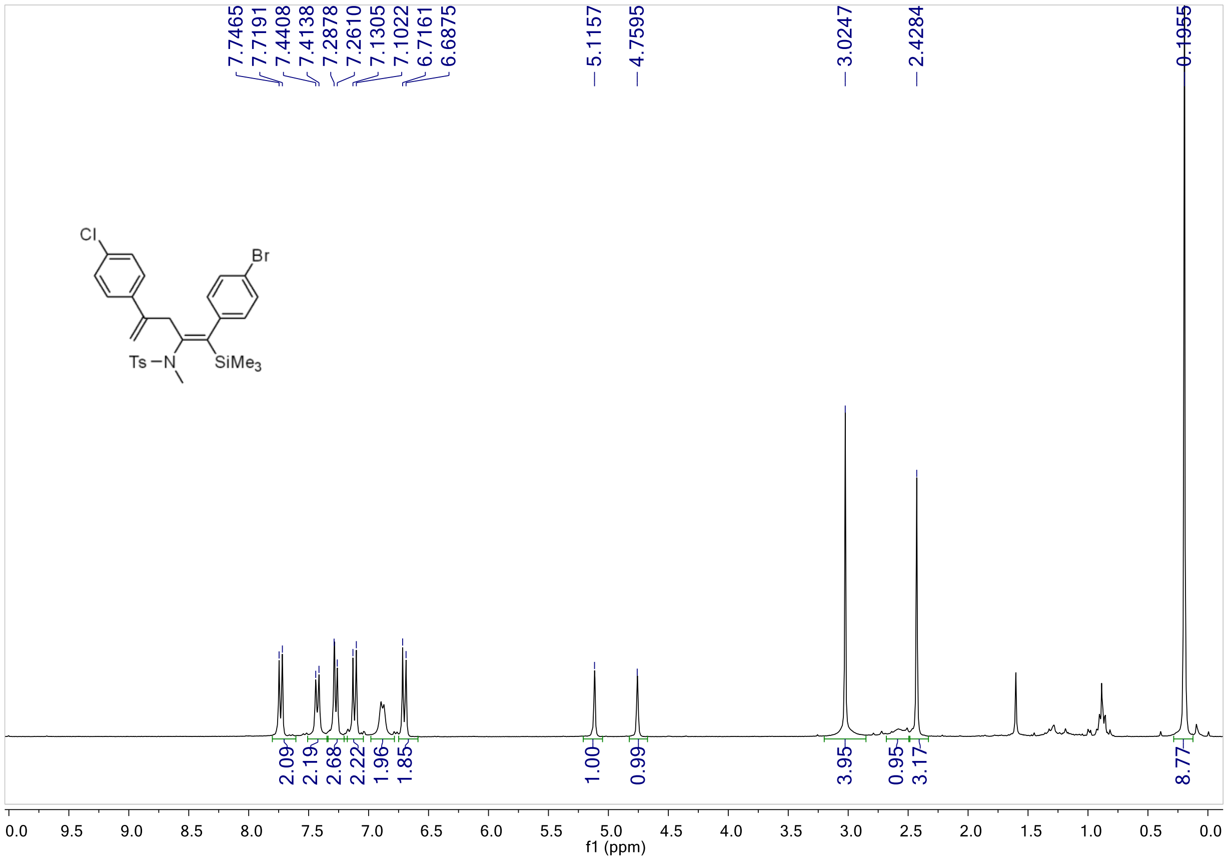

^{13}C NMR (75 MHz, $CDCl_3$) spectrum of 3w

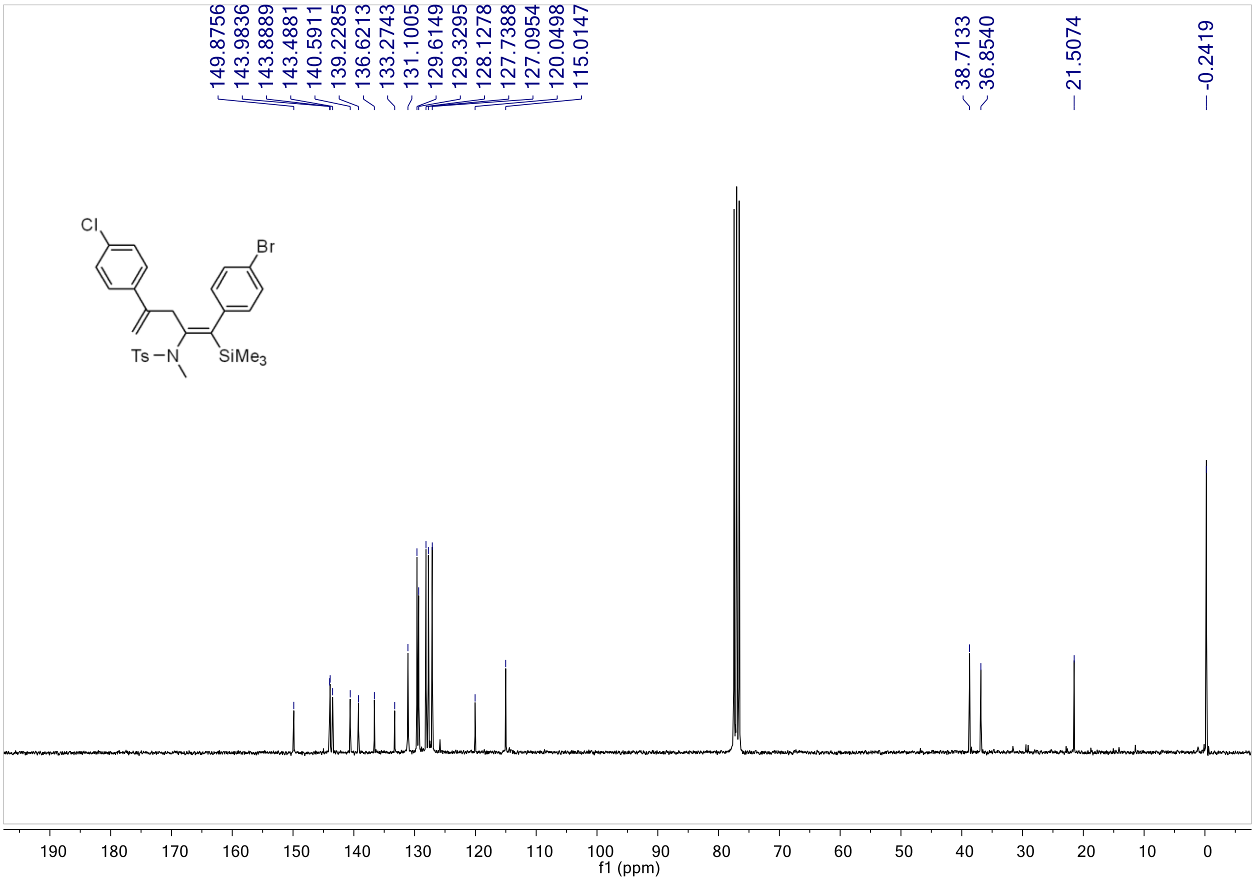

^{1}H NMR (300 MHz, $CDCl_3$) spectrum of 3x

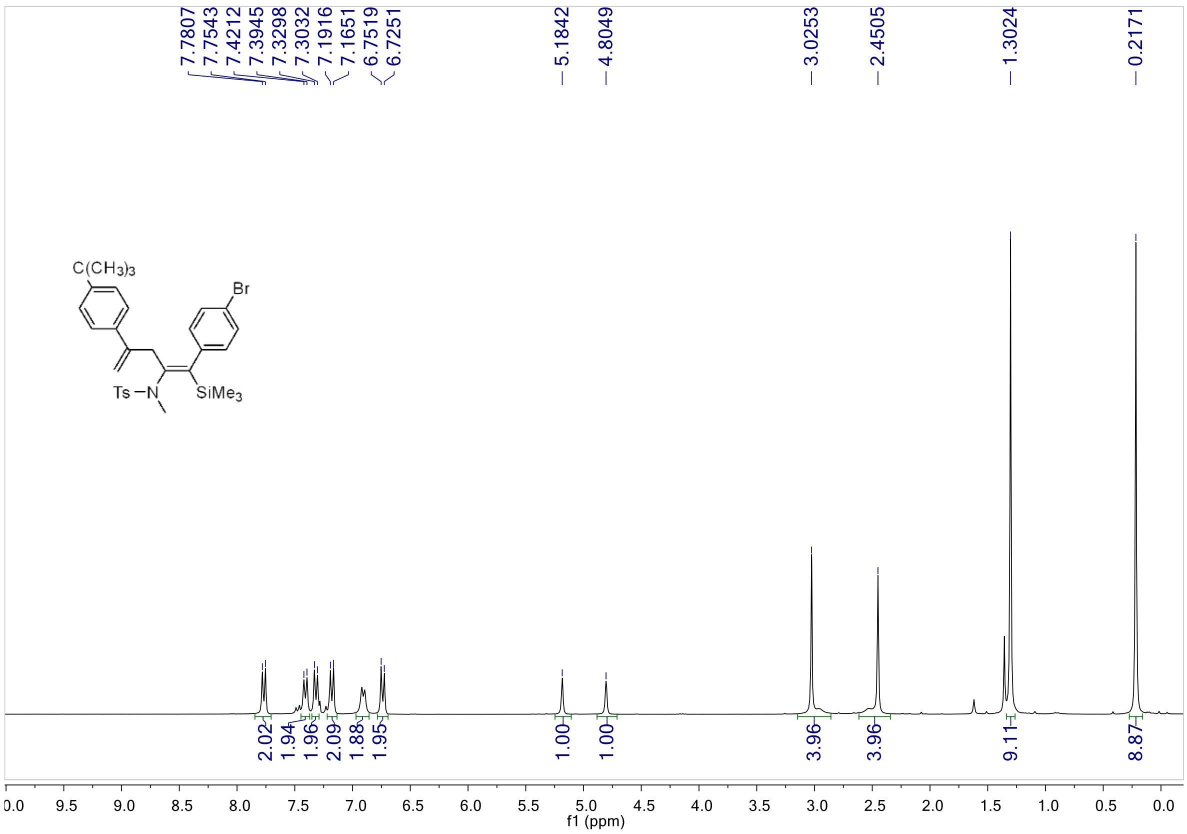

^{13}C NMR (75 MHz, CDCl$_3$) spectrum of 3x

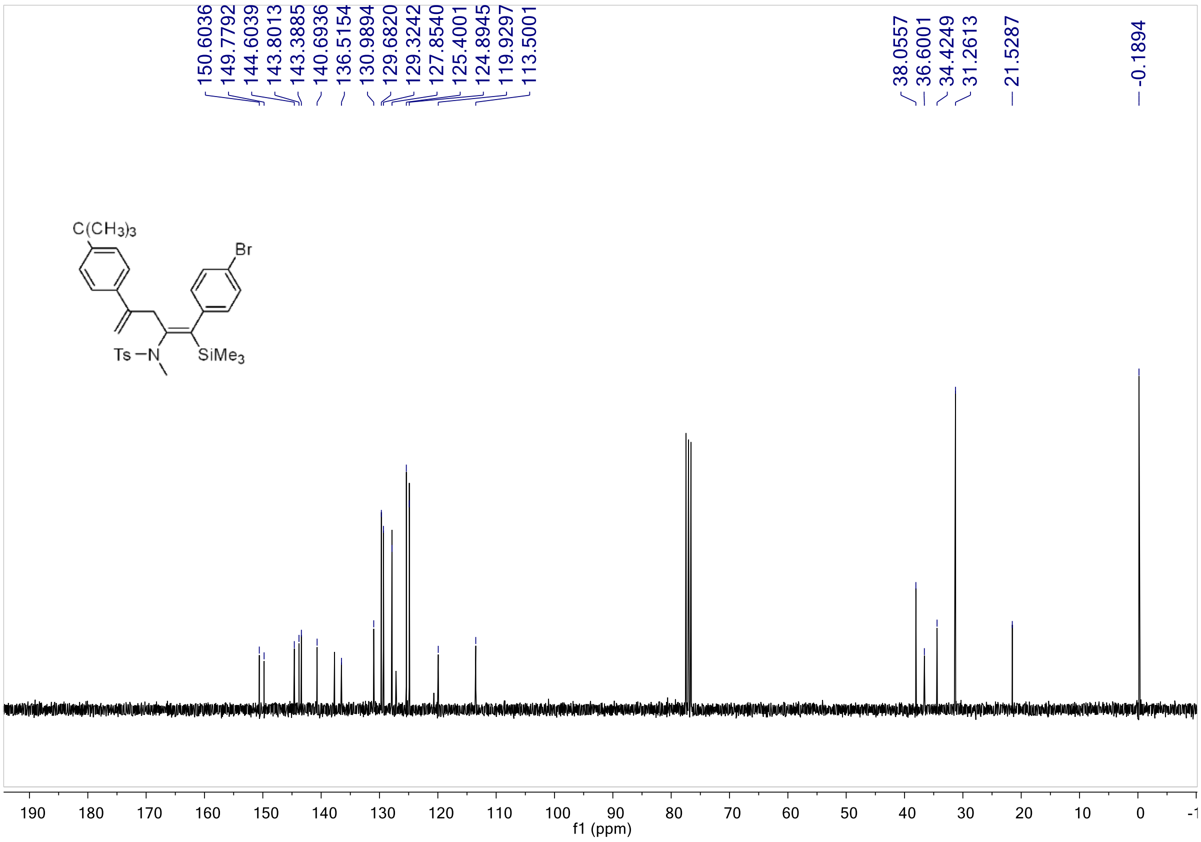

^{29}Si NMR (75 MHz, CDCl$_3$) spectrum of 3x

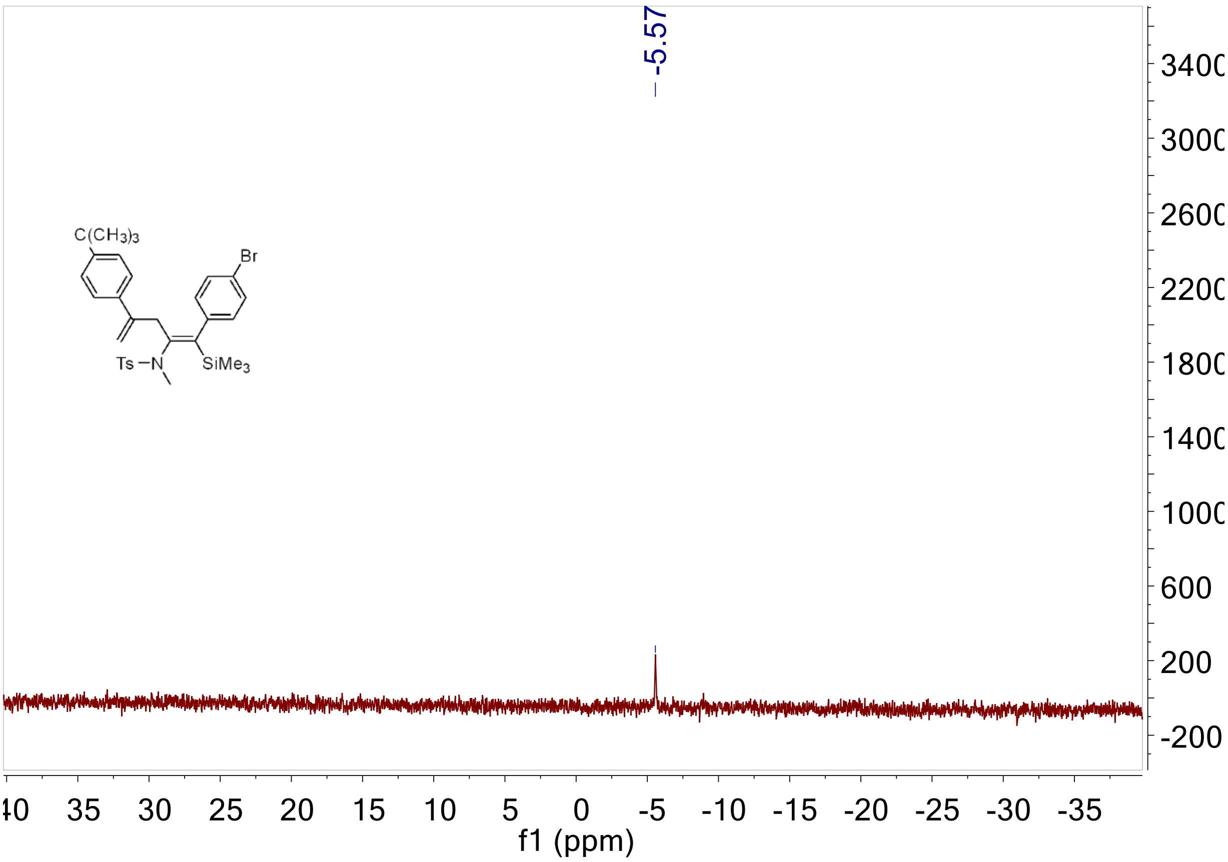

^{1}H NMR (300 MHz, $CDCl_3$) spectrum of 3y

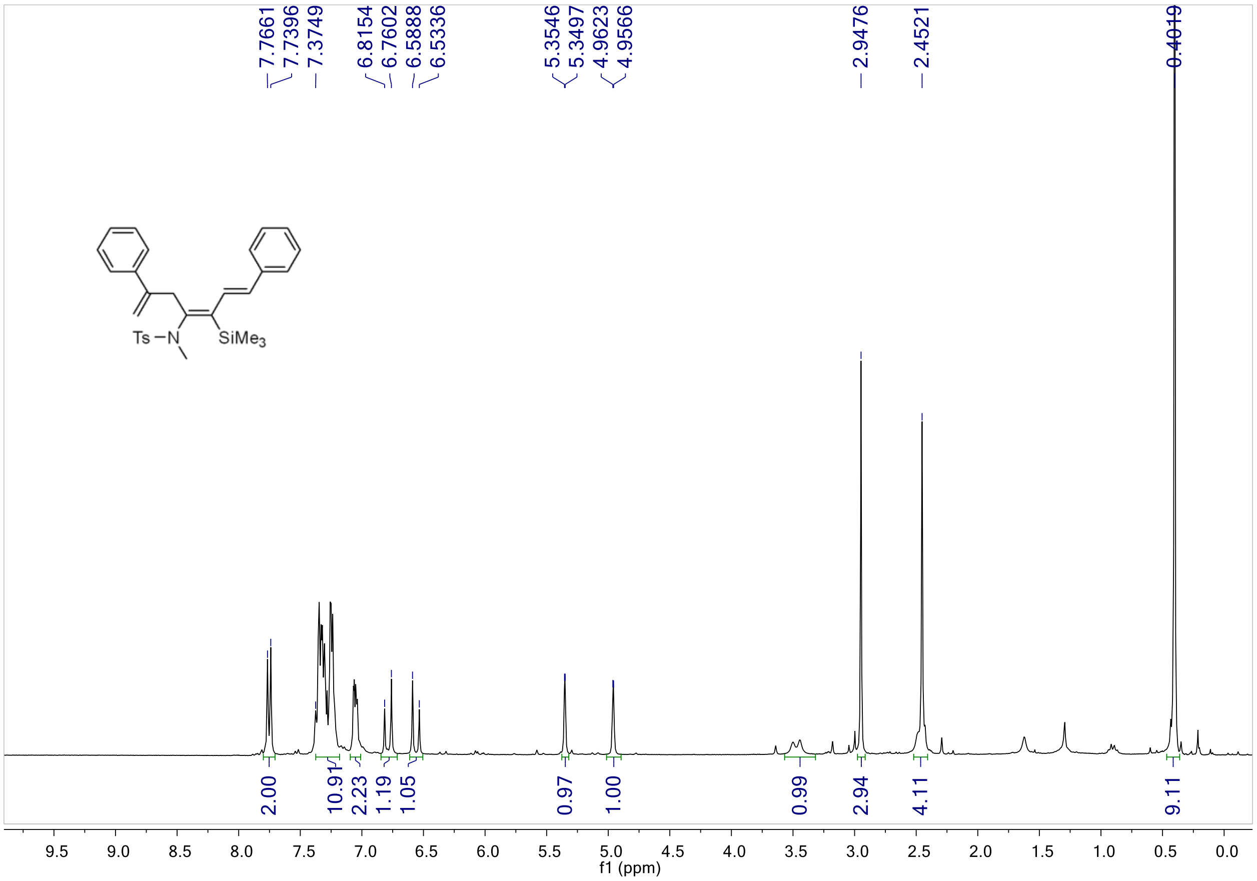

^{13}C NMR (75 MHz, $CDCl_3$) spectrum of 3y

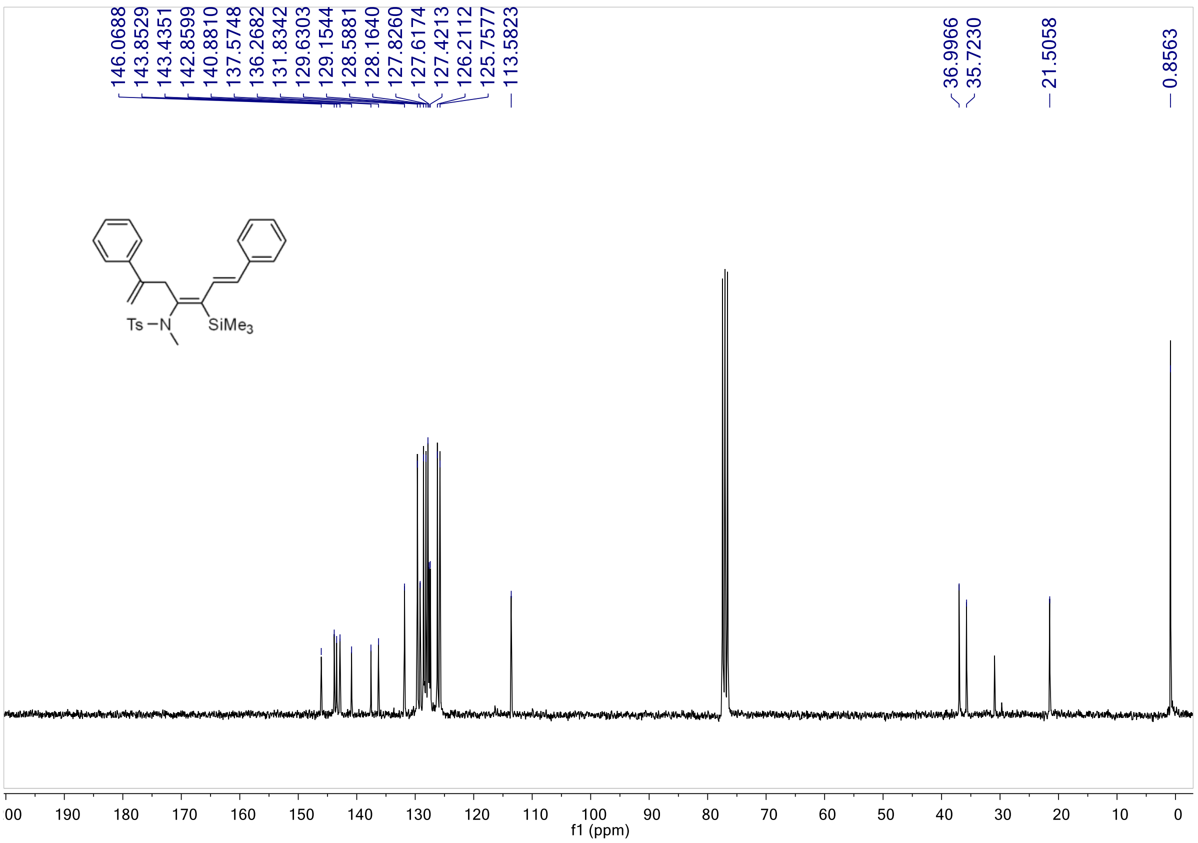

^{1}H NMR (300 MHz, CDCl3) spectrum of 4

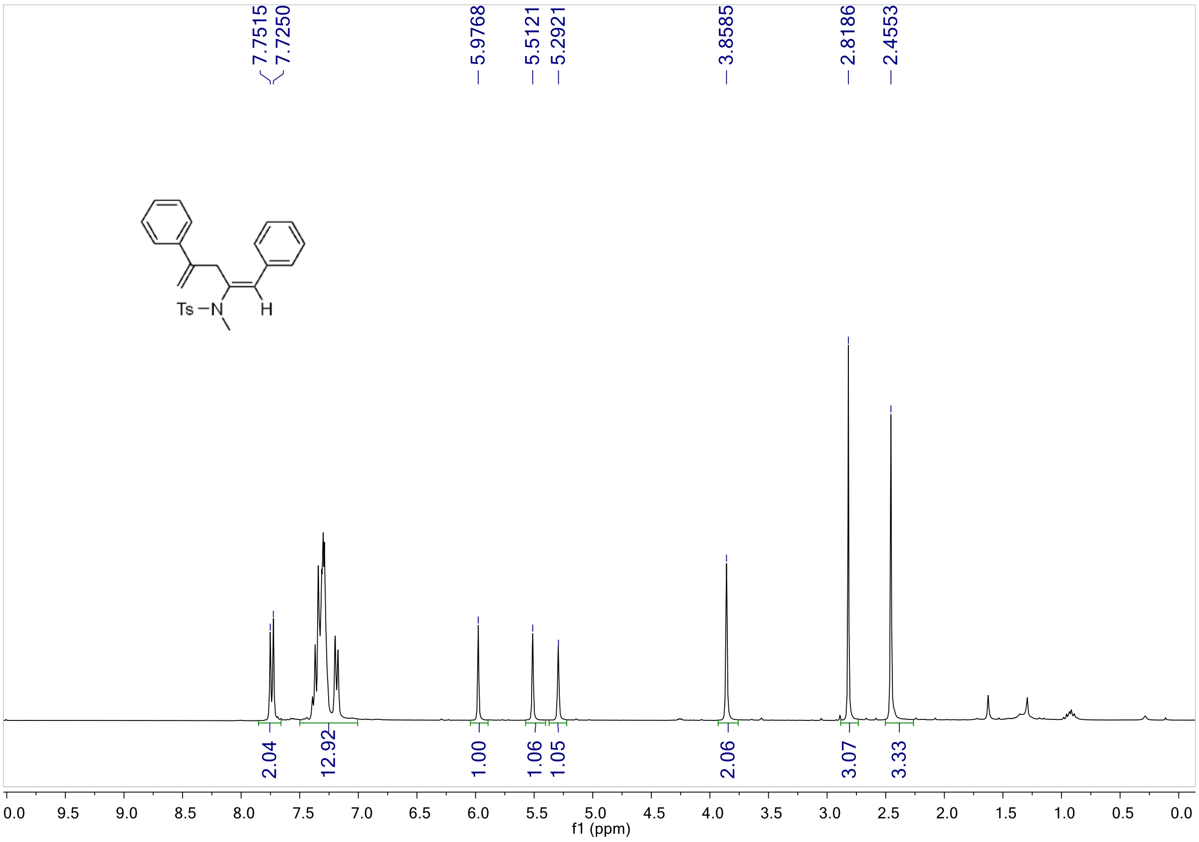

^{13}C NMR (75 MHz, CDCl3) spectrum of 4

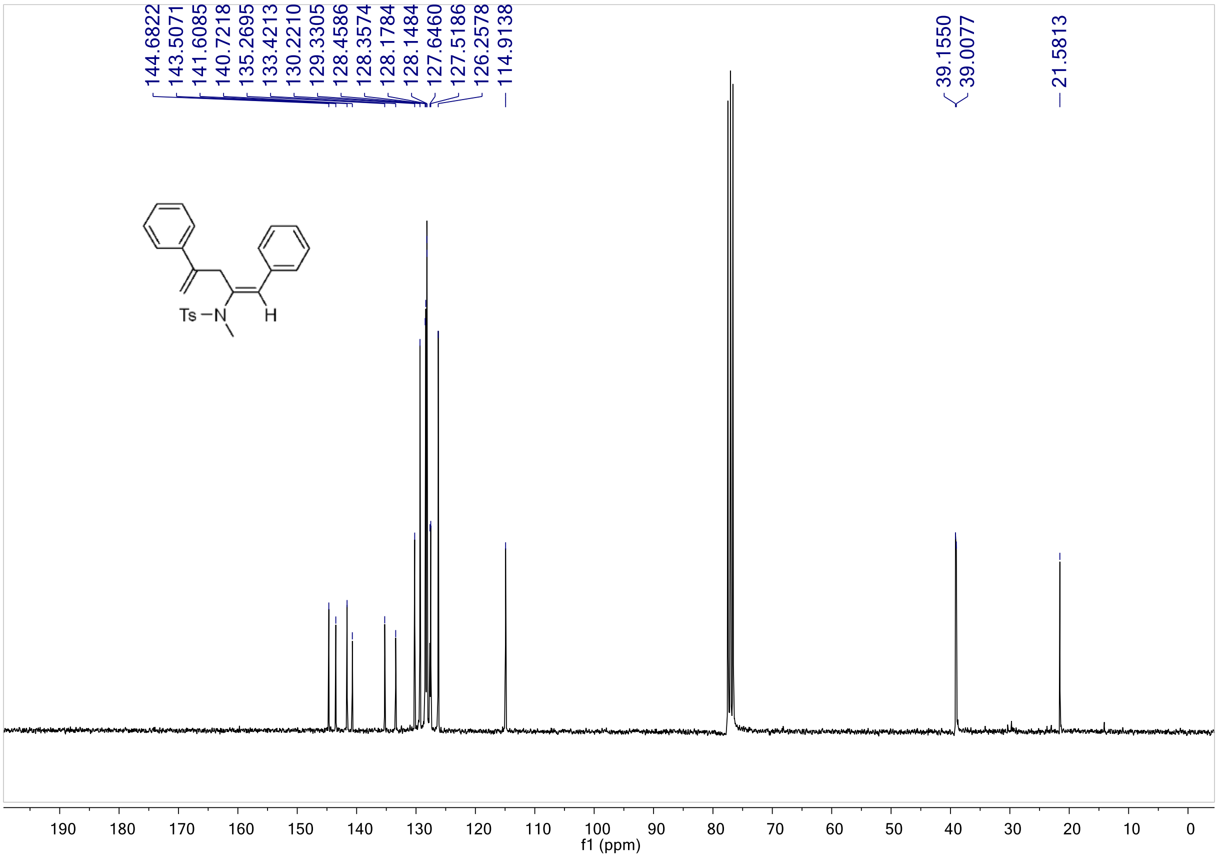

^{1}H NMR (300 MHz, CDCl$_3$, 338 K) spectrum of 5

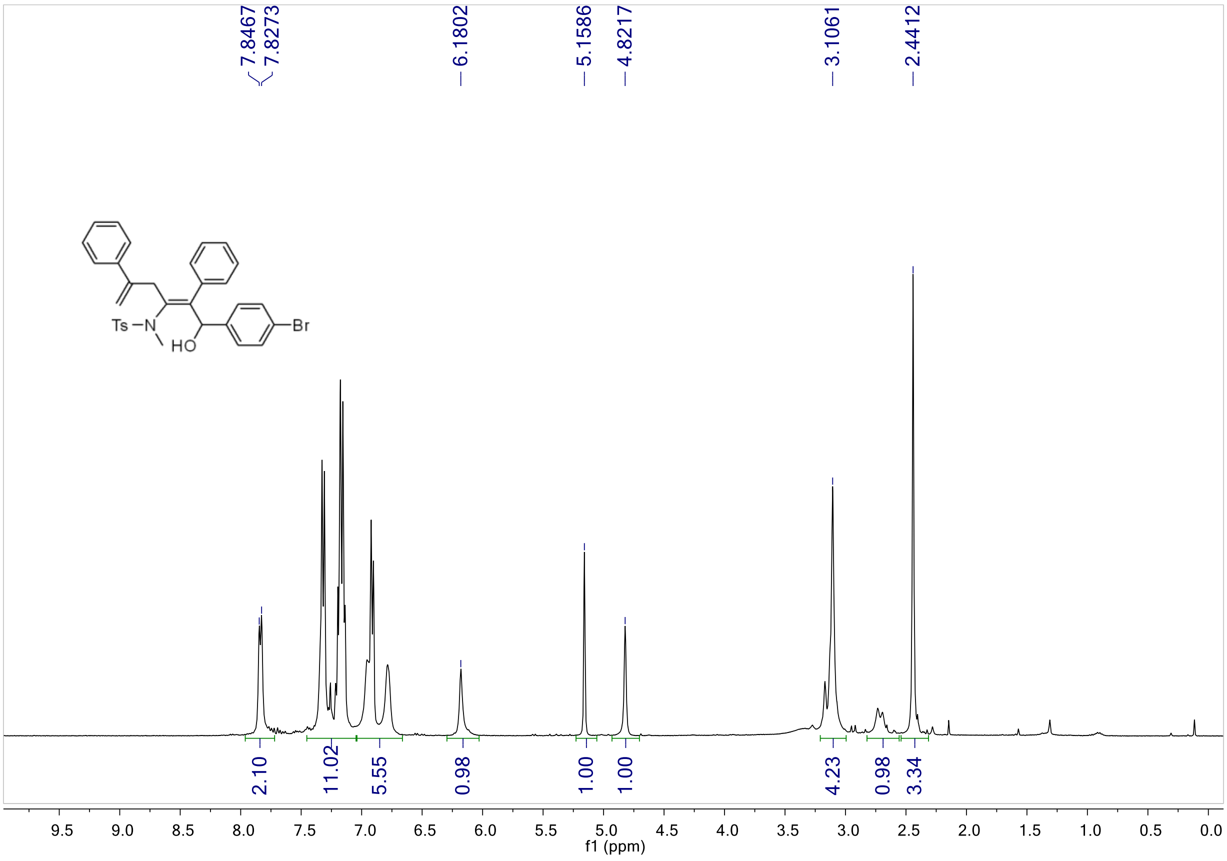

^{13}C NMR (75 MHz, CDCl$_3$) spectrum of 5

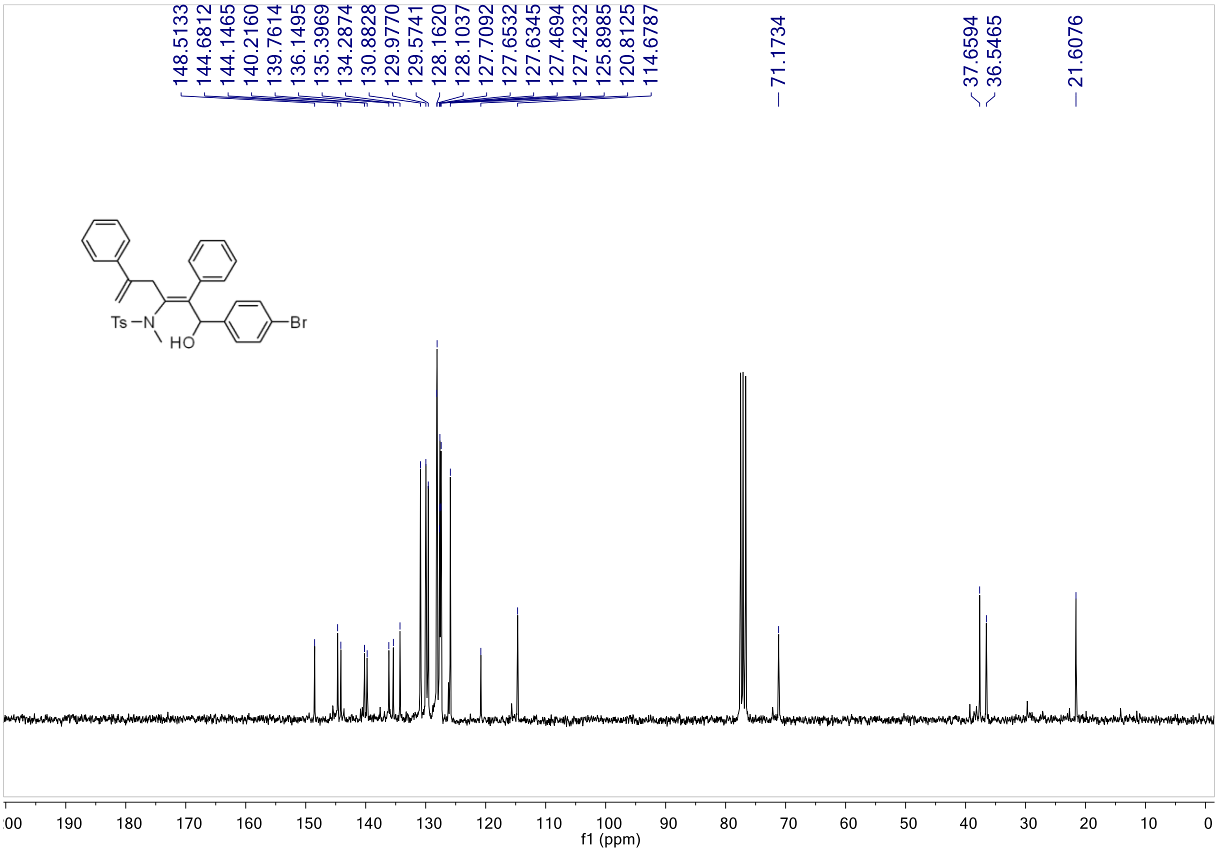

^{1}H NMR (300 MHz, CDCl3) spectrum of 6

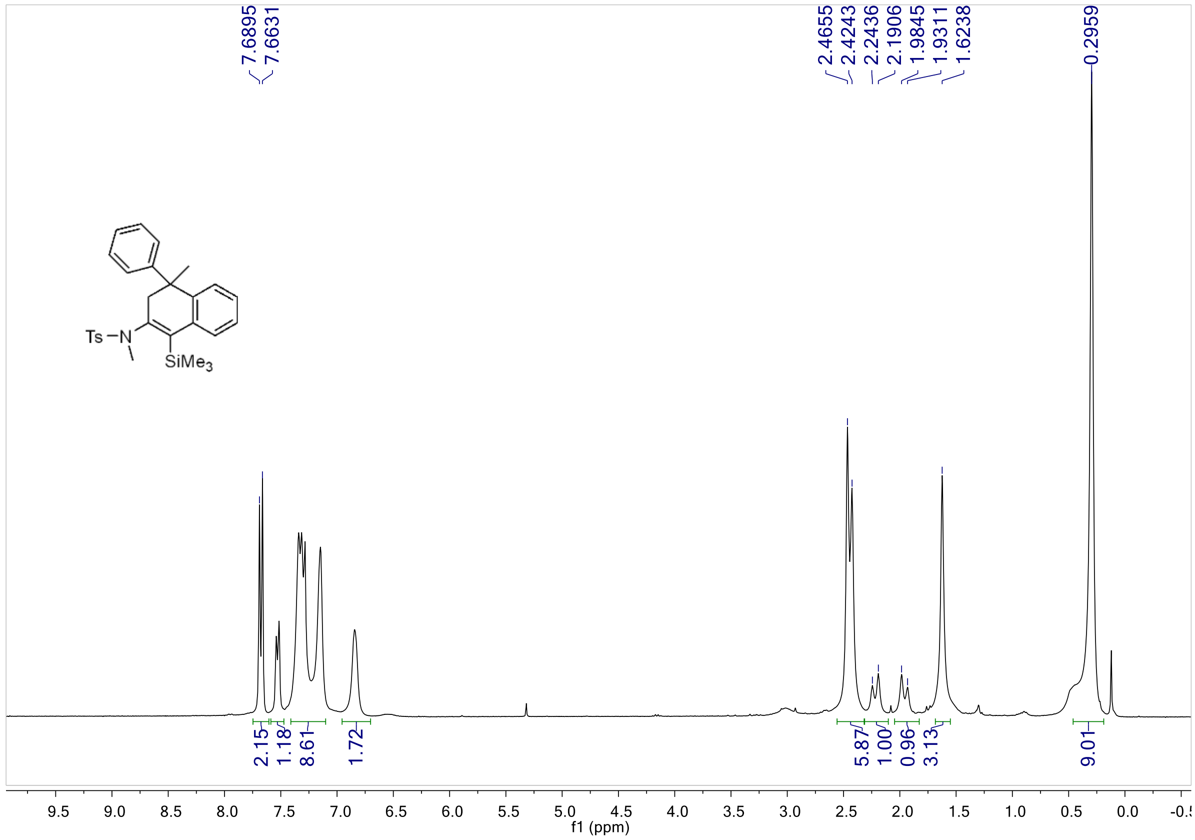

^{13}C NMR (75 MHz, CDCl3) spectrum of 6

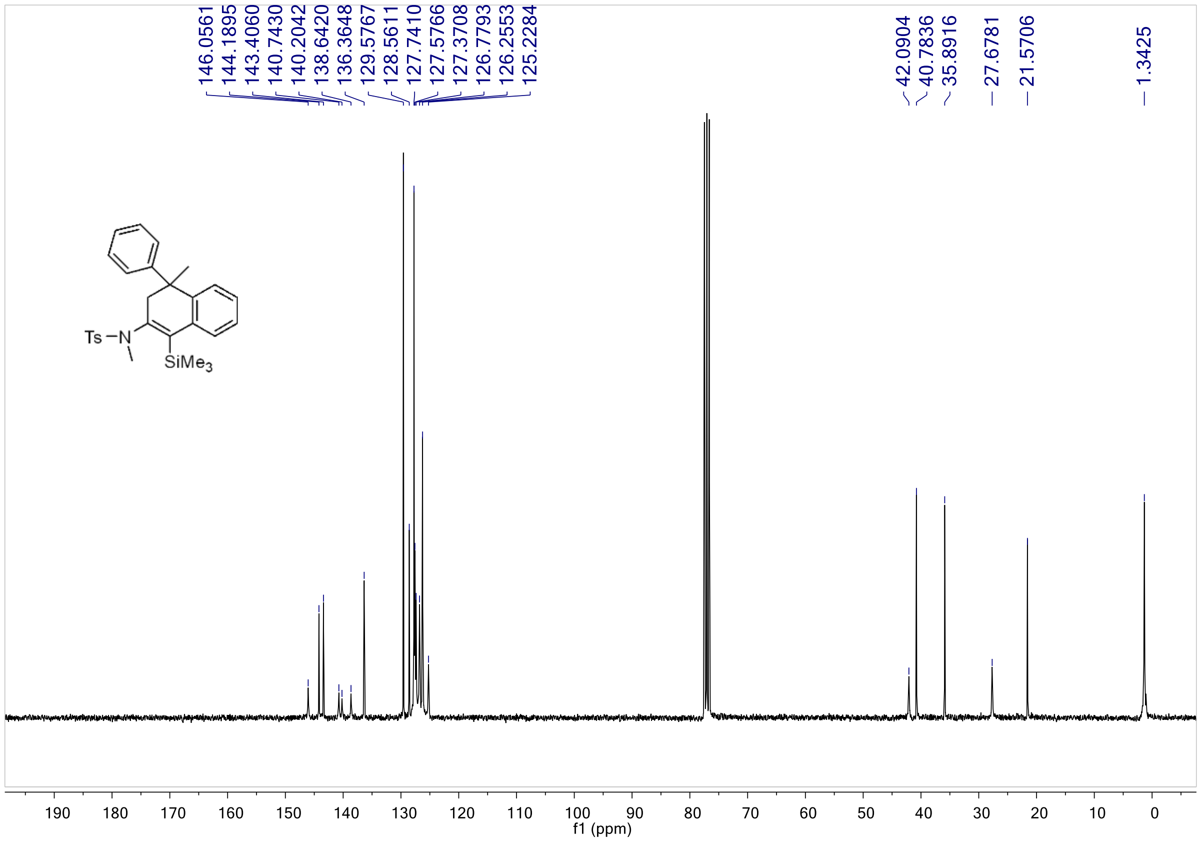

^{29}Si NMR (75 MHz, $CDCl_3$) spectrum of 6

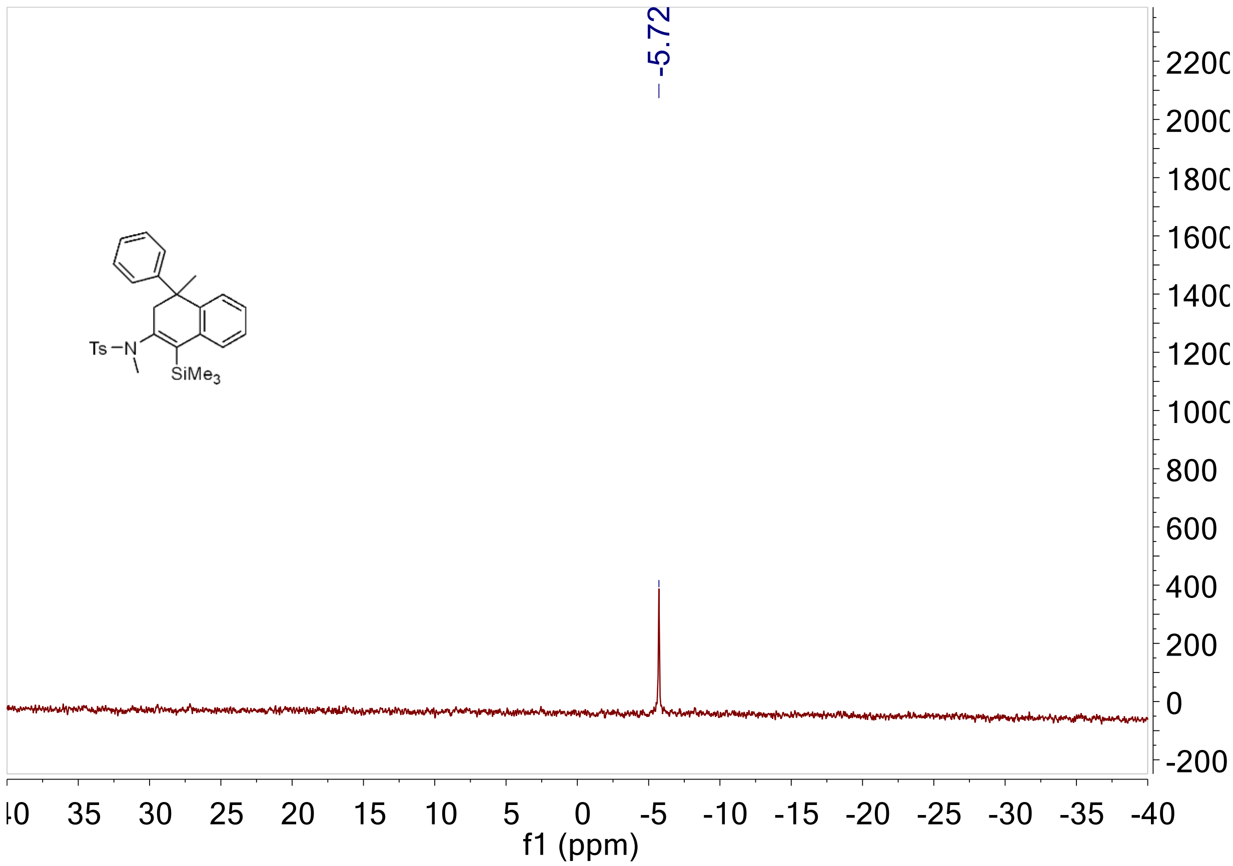

4.- X-Ray Crystal Structure Determination:

Crystals growth method

Crystals of **3j,q** suitable for X-ray analysis were obtained by slow evaporation of a concentrated solution of the corresponding compound in hexanes.

Crystallographic data

3j: empirical formula $C_{23} H_{30} F N O_2 S Si$, M_r = 431.63, T = 293 (2) K, λ = 1.5418 Å, crystal system, space group: orthorombic, *Pbca*, unit cell dimensions: a = 15.1534 (2), b = 10.7992 (2), c = 29.3469 (5) Å, $\alpha=\beta=\gamma$ =90.000 (0)º, V = 4802.46 (12) Å^3, Z = 8, ρ_{calcd} = 1.194 Mgm^{-3}, μ = 1.883 mm^{-1}, F(000) = 1840, crystal size: 0.475 x 0.226 x 0.118 mm, θ range data collection: 4.094–69.694º, index ranges: $-18\leq h\leq 18$, $-12\leq k\leq 12$, $-35\leq l\leq 31$, reflections collected/unique = 13362/4456 [R_{int} = 0.0223, completeness to 2θ = 68.133 (99.6%), absorption correction: gaussian, max. and min. transmission = 1.000 and 0.442, refinement method: full matrix least-squares on F^2, data/restraints/parameters = 4456/0/262, goodness-of-fit on F^2 = 1.037, final R indices [$I>2\sigma(I)$]: R_1 = 0.0448, wR_2 = 0.1311, R indices (all data): R_1 = 0.0514, wR_2 = 0.1400, largest difference peak and hole = 0.253 and –0.301 eÅ^{-3}.

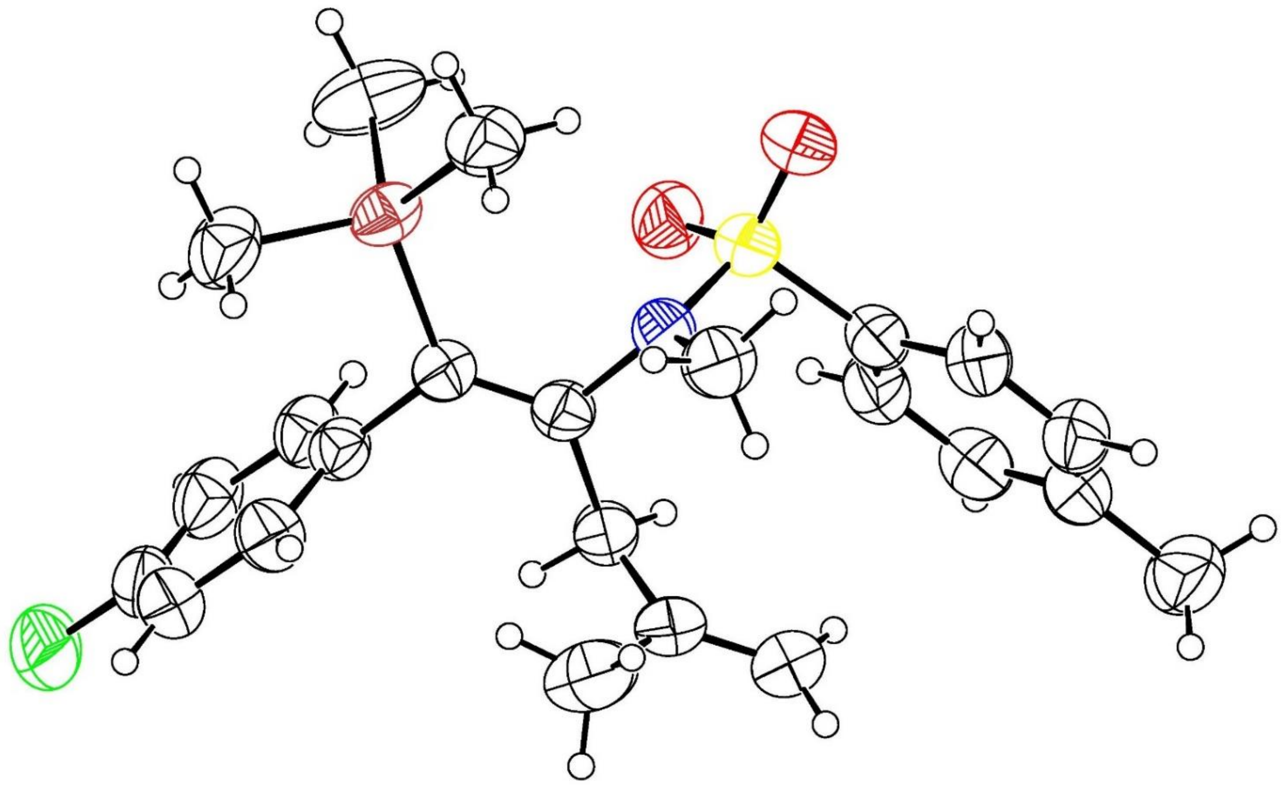

Ellipsoids at 50% of probability level

3q: empirical formula C_{28} H_{32} Br N O_2 S Si, M_r = 554.60, T = 293 (2) K, λ = 1.5418 Å, crystal system, space group: monoclinic, *P21/c*, unit cell dimensions: a = 9.8894 (2), b = 16.9643 (5), c = 17.0928 (5) Å, β = 94.308 (2)º, V = 2859.50 (13) Å^3, Z = 4, ρ_{calcd} = 1.288 Mgm^{-3}, μ = 3.207 mm^{-1}, F(000) = 1152, crystal size: 0.241 x 0.194 x 0.086 mm, θ range data collection: 3.676–69.694º, index ranges: $-11 \le h \le 10$, $-18 \le k \le 20$, $-20 \le l \le 15$, reflections collected/unique = 13932/5304 [R_{int} = 0.0410, completeness to 2θ = 68.133 (99.8%), absorption correction: gaussian, max. and min. transmission = 1.000 and 0.673, refinement method: full matrix least-squares on F^2, data/restraints/parameters = 5304/0/309, goodness-of-fit on F^2 = 1.045, final R indices [I>2σ(I)]: R_1 = 0.0484, wR_2 = 0.1372, R indices (all data): R_1 = 0.0636, wR_2 = 0.1548, largest difference peak and hole = 0.591 and –0.396 eÅ^{-3}.

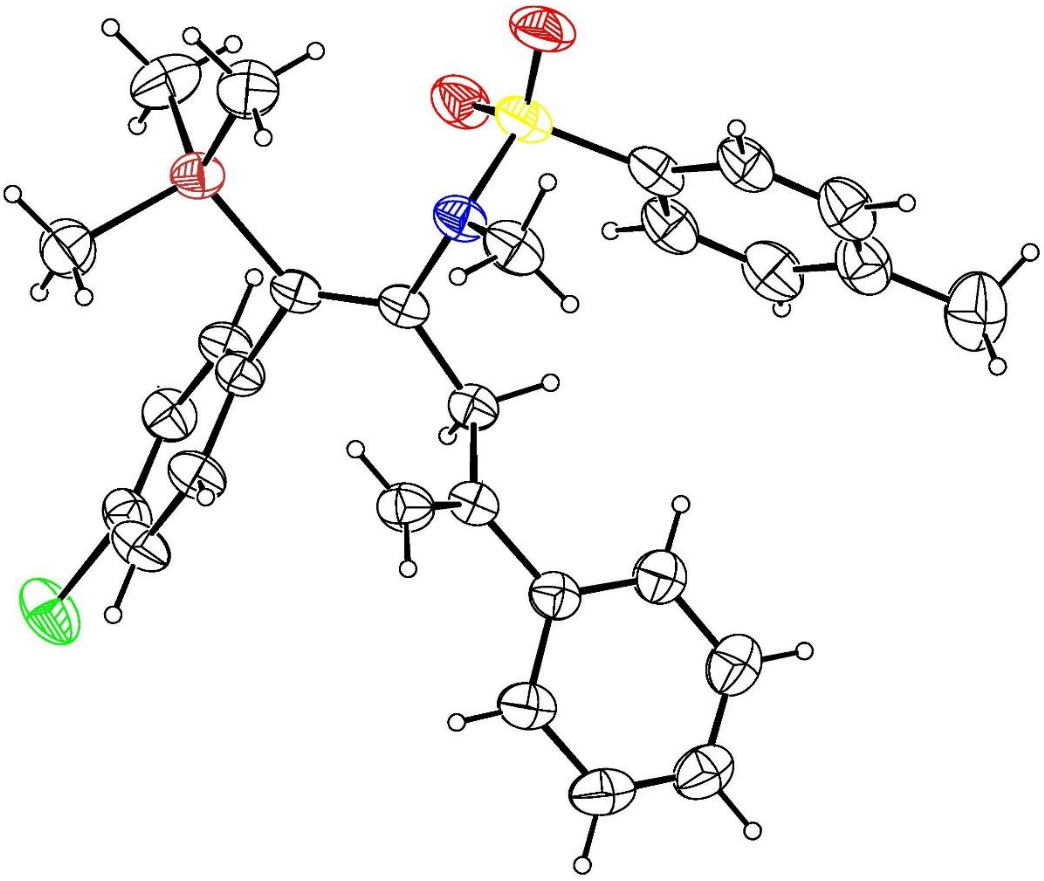

Ellipsoids at 50% of probability level

CCDC 2193409-2193410 contain the supplementary crystallographic data for this paper. These data can be obtained free of charge via www.ccdc.cam.ac.uk/data_request/cif, or by emailing data_request@ccdc.cam.ac.uk, or by contacting The Cambridge Crystallographic Data Center, 12 Union Road, Cambridge CB2 1EZ, UK; fax +44 1223 336033

5.-Theoretical calculations

Computational details

All calculations were carried out using the Gaussian 16 program package.[12] In view of the large molecules investigated experimentally, the molecular geometries were fully optimized without any molecular symmetry constraints for all model compounds using the M05-2X functional,[13] which includes dispersion energy effects, in combination with the 6-31G(d) basis set.[14] This level of theory proved useful in the elucidation of reaction mechanisms of silicon compounds species.[15] The optimized structures were characterized as minima or saddle points by frequency calculations. In order to fully prove the relevance of the transition structures we also computed the IRC (intrinsic reaction coordinate) connecting the critical points to confirm that the TS really relate the minima. Finally, to account for the solvation effects on the reaction profile, punctual calculations on the optimized structures were performed including the Polarizable Continuum Model (PCM),[16] using the integral equation formalism variant and the dielectric constant of dichloroethane (ε=10.1250).

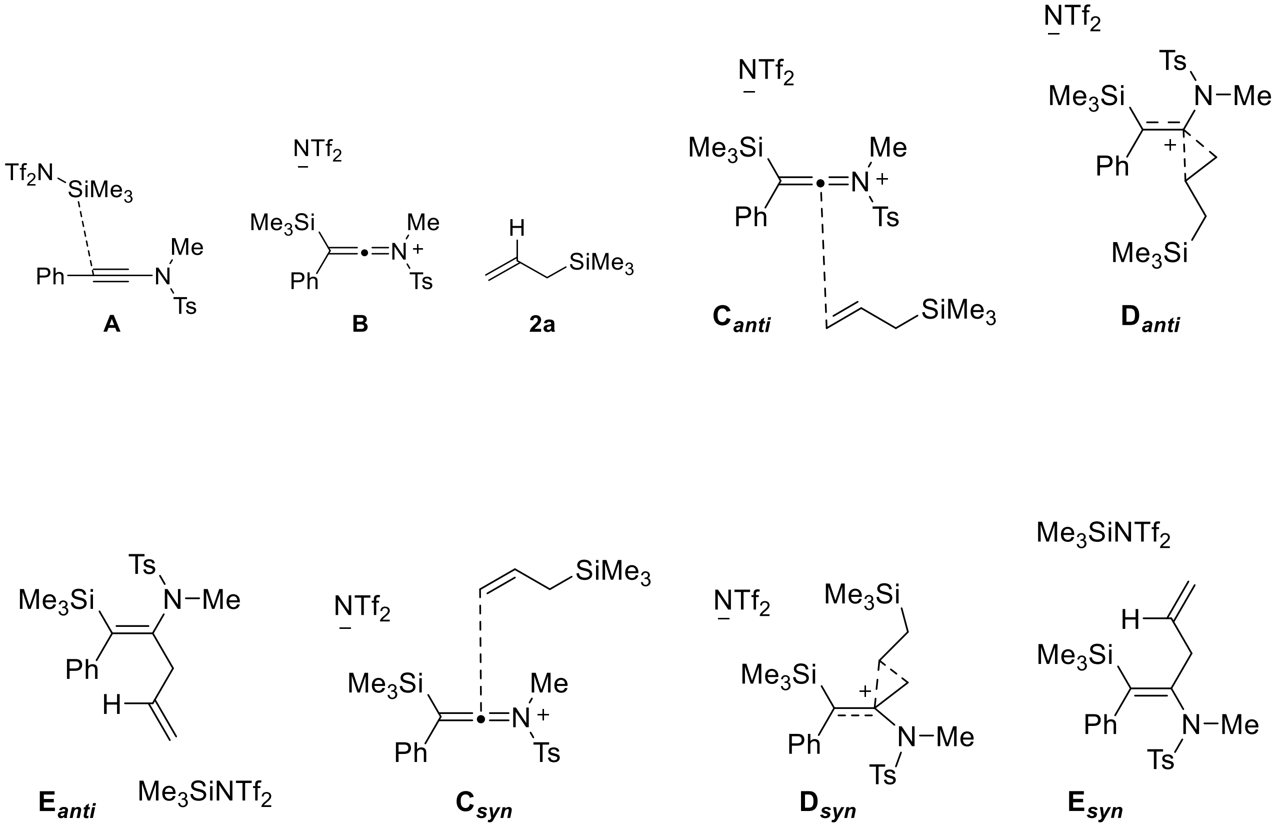

anti- *vs syn*-addition

The difference in the energy barriers to reach **TS2*anti*** or **TS2*syn*** is calculated to be 4.8 kcal·mol^{-1} (Figure S1). The optimized geometry for model compound **B** (Figure S2), shows bond angles of 107° (Si–C_{Ph}–C_N) and 131° (Ph–C_{Ph}–C_N). This difference of 24° could be explained by a stabilizing effect due to the hyperconjugative interaction between the Si–C_{Ph} σ-bond and the empty p-orbital of the carbocationic C_N atom (*β*-silicon effect).[17,18] As a result of the reduced angle, the *syn*-attack on that side becomes more difficult due to steric interactions, which raises its energy barrier and makes the *anti*-attack more favorable, in agreement with the experimental results.

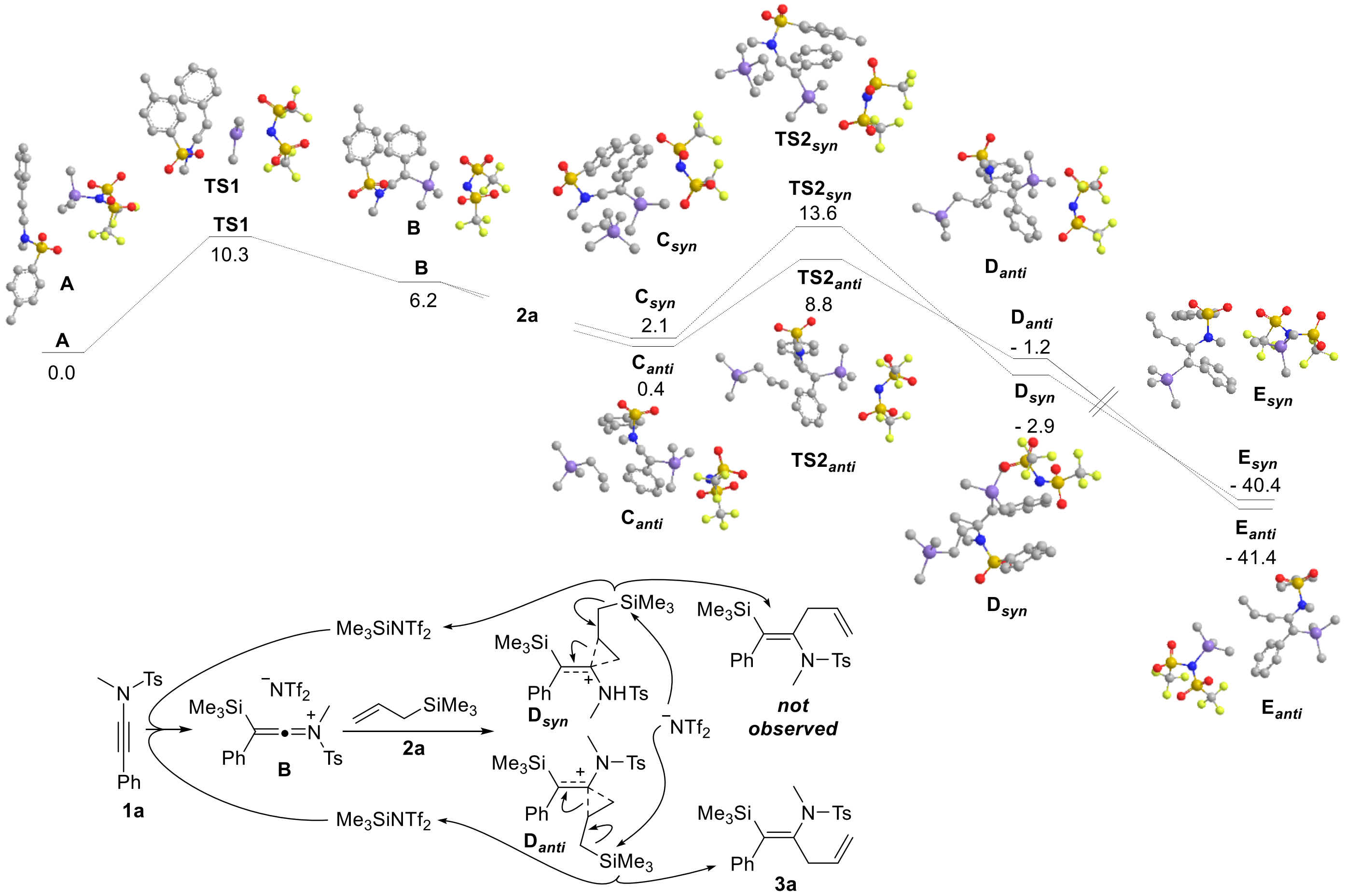

Figure S1. Calculated relative energy profile for the anti- and syn-addition, in kcal·mol^{-1} (for the sake of comparison, the values shown for **A**, **TS1**, and **B** also include the energy value of **2a**).

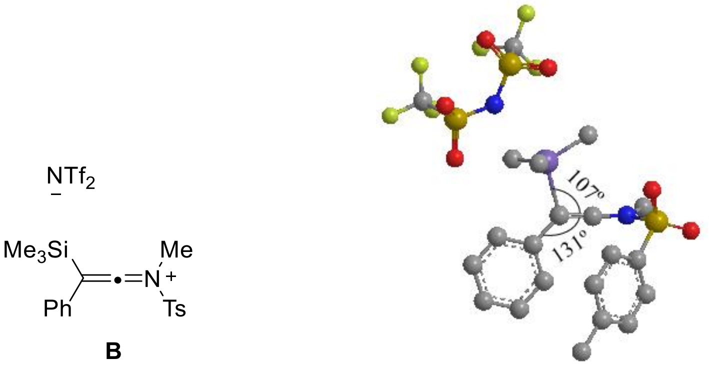

Figure S2. Optimized geometry for model compound **B**. H atoms have been omitted for clarity.

Symbolic Z-Matrix, Absolute Energies, in atomic units, and Number of Imaginary Frequencies [PCM-M05-2X/6-31G*//M05-2X/6-31G* level]

Model compound A

C	1.84538900	3.12387300	0.43607800
C	2.50830200	2.14470500	0.68844700
C	0.98726700	4.22309800	0.11366500
C	0.84063000	4.62815600	-1.22064500
C	0.25529500	4.87304200	1.11681600
C	-0.03095100	5.66152000	-1.54054900
H	1.40714600	4.12170600	-1.99117700
C	-0.60658900	5.91114300	0.78700600
H	0.36618500	4.55474700	2.14526400
C	-0.75526600	6.30616800	-0.54025800
H	-0.14440900	5.96425200	-2.57358000
H	-1.16970600	6.40722400	1.56701200
H	-1.43365200	7.11032000	-0.79400900
N	3.25269600	1.05248300	0.98743100
C	3.23292800	0.61109600	2.39261300
H	3.95590100	-0.19446700	2.51189900
H	3.53978700	1.45642200	3.00351600
H	2.24040200	0.26933300	2.68683400
S	3.13224500	-0.21655100	-0.16346100
C	4.72424700	-0.96404700	0.00472200
C	5.83970100	-0.26357100	-0.44893600
C	4.82841100	-2.23444000	0.55475700
C	7.08643600	-0.85721100	-0.33392700
H	5.72169400	0.72074300	-0.88325100
C	6.08990200	-2.81240800	0.65917900
H	3.93730400	-2.75182300	0.88449100
C	7.22785200	-2.13646800	0.21859700
H	7.96590000	-0.32729400	-0.67949700
H	6.18972300	-3.80296300	1.08555300
C	8.58927300	-2.77386700	0.30922200
H	9.33061800	-2.06172200	0.67423100
H	8.57854400	-3.63430300	0.97752700
H	8.91630800	-3.11569200	-0.67551400
O	3.02193900	0.43644000	-1.45586200
O	2.12505900	-1.17416700	0.28797500
Si	-1.58893500	1.03110700	-0.15377900
C	-0.39555600	0.83307500	-1.57613700
H	0.10600000	-0.13264000	-1.61534500
H	-0.87153600	1.02423600	-2.53643600
H	0.38678400	1.58030600	-1.41511300
C	-0.75442900	0.77575900	1.49957400
H	-0.07330800	-0.07506600	1.44788300
H	-0.16837800	1.67026700	1.72400600
H	-1.48025900	0.62535000	2.29683600
C	-2.54230800	2.62914500	-0.32256500
H	-1.84121200	3.44106700	-0.53315900
H	-3.24278300	2.56138800	-1.15819400
H	-3.09924200	2.86815800	0.58191400
N	-2.86428800	-0.35928300	-0.33337300
S	-2.85315800	-1.48544000	-1.57269300
S	-4.22366200	-0.30894600	0.66249100
C	-4.21428300	-1.96770900	1.52924000
C	-1.35875200	-2.53544500	-1.13218700
O	-5.45749100	-0.22202900	-0.08277400
O	-3.88241000	0.66645600	1.68398000
O	-4.00471100	-2.34963300	-1.40127100
O	-2.54212400	-0.84768200	-2.83289300
F	-1.77828000	-3.75771600	-0.84976900
F	-0.55210900	-2.56982900	-2.18070300
F	-0.70842500	-2.03946700	-0.08770200
F	-5.17877400	-2.73398800	1.06984600
F	-3.03414100	-2.56172400	1.36959800
F	-4.40887700	-1.72852300	2.81872000

Absolute Energy: -3458,129534 u.a.
Number of Imaginary Frequencies: 0

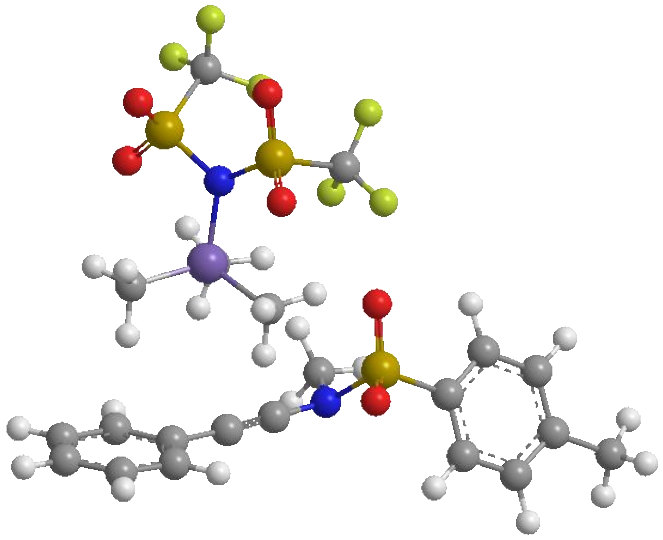

Model compound TS1

C	-1.62368600	0.18562400	-0.81269700
C	-2.21412900	-0.90552200	-1.03166200
C	-2.00313400	1.59694100	-0.92375100
C	-3.30380900	1.92059200	-1.34053700
C	-1.11595100	2.62214200	-0.58257100
C	-3.70605100	3.24487600	-1.42053500
H	-3.99560100	1.12447500	-1.59062700
C	-1.52666600	3.94922700	-0.66522700
H	-0.11130500	2.40217400	-0.24773700
C	-2.81483100	4.26375700	-1.08387000
H	-4.70994900	3.48307700	-1.74947200
H	-0.83137800	4.73411500	-0.39874900
H	-3.12580300	5.29868300	-1.14876800
N	-2.80435800	-2.05058500	-1.06333400
C	-2.72822700	-3.02004600	-2.17275300
H	-3.73149500	-3.34180000	-2.43698100
H	-2.25201200	-2.51332200	-3.00767700
H	-2.13197100	-3.87615800	-1.86395800
S	-3.68782100	-2.52883500	0.40119800
C	-4.68874400	-1.10312900	0.65143700
C	-4.23740300	-0.10749000	1.51440100
C	-5.87053300	-0.97897100	-0.07218700
C	-4.99117400	1.05024100	1.63425800
H	-3.31941100	-0.24222600	2.07021800
C	-6.60947300	0.18910400	0.06831400
H	-6.20438600	-1.78200000	-0.71650400
C	-6.17578000	1.21789400	0.90967100
H	-4.65027200	1.84148200	2.29045600
H	-7.53558500	0.30386800	-0.48097000
C	-6.95383800	2.50002500	1.03356800
H	-7.87635700	2.46322900	0.45552900
H	-7.20805100	2.69984300	2.07583000
H	-6.35384000	3.33920300	0.67419500
O	-2.70970700	-2.62953000	1.46599000
O	-4.47981900	-3.65725900	-0.05228200
Si	0.41059100	-0.38364400	-0.21767600
C	0.36081300	-2.25123100	-0.38989100
H	0.24265700	-2.54822400	-1.43501600
H	1.29032000	-2.67464600	-0.01420600
H	-0.44862000	-2.66912400	0.21163400
C	1.09645500	0.42078600	-1.78283800
H	1.99240700	-0.13640100	-2.05566800
H	0.36753100	0.34269900	-2.59166100
H	1.37661200	1.46127700	-1.64738300
C	0.06870400	0.11537400	1.56477300
H	-0.86239500	-0.37640400	1.85769600
H	0.87073400	-0.27185400	2.19269000
H	-0.01414300	1.18816200	1.72062700
N	2.83095200	-0.08559300	0.48481600
S	3.81025900	-1.31627200	0.92551800
S	3.14983800	1.44490000	0.93864500
C	4.46779600	2.02723800	-0.20883300
C	4.35623300	-1.87766800	-0.73854700
O	3.68271900	1.58853600	2.27974800
O	1.99046500	2.24246000	0.53327000
O	5.02687100	-0.91732100	1.60900700
O	2.99874500	-2.41468100	1.43409100
F	4.97250300	-0.88952200	-1.37999600
F	5.18721400	-2.90334800	-0.60427900
F	3.30312000	-2.26578600	-1.46696100
F	5.61529300	1.40801900	0.01701800
F	4.08805200	1.80670900	-1.46794300
F	4.62691200	3.33517300	-0.02827500

Absolute Energy: -3458,113196 u.a.
Number of Imaginary Frequencies: 1 (99i)

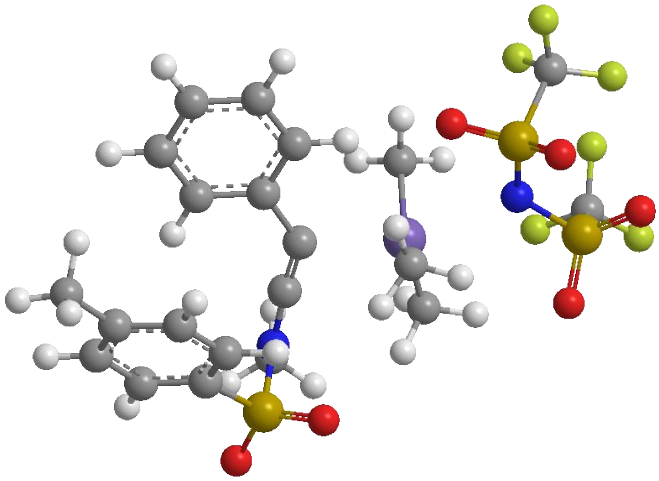

Model compound B

C	1.67585600	0.11854500	0.87191300
C	2.37235600	-0.91984100	1.11046400
C	2.00824600	1.54697900	0.96724500
C	3.31488500	1.93083600	1.30423700
C	1.05109700	2.52640000	0.68510700
C	3.65665700	3.27261000	1.36400500
H	4.06020400	1.17012800	1.50751200
C	1.40196300	3.87169800	0.74763600
H	0.03660500	2.26422900	0.41354200
C	2.69717000	4.24718700	1.08649100
H	4.66638200	3.55930700	1.63051300
H	0.65298900	4.62060500	0.52752000
H	2.96139300	5.29584000	1.13579200
N	3.04409800	-2.00144400	1.19095500
C	3.05420900	-2.91793200	2.35005700
H	4.08191600	-3.10685800	2.64602000
H	2.49953600	-2.43321600	3.14854600
H	2.57016100	-3.85007300	2.06641100
S	3.98213100	-2.51108500	-0.26123700
C	4.77222500	-0.99775900	-0.67200200
C	4.16169000	-0.15423600	-1.59775800
C	5.94989200	-0.65497700	-0.01414000
C	4.74475600	1.07867300	-1.84634400
H	3.25801400	-0.46466800	-2.10480600
C	6.51741400	0.58311500	-0.28697100
H	6.41087300	-1.34589600	0.67979400
C	5.91777200	1.46689200	-1.18979900
H	4.27921100	1.75477700	-2.55260600
H	7.43795700	0.86825500	0.20675300
C	6.50321700	2.82849900	-1.44664800
H	7.47404000	2.94188800	-0.96592200
H	6.62388500	3.00417600	-2.51656900
H	5.83351400	3.59978900	-1.05867600
O	3.00718000	-2.85567100	-1.27410700
O	4.91861100	-3.46773600	0.29611100
Si	-0.19828100	-0.51828900	0.30998900
C	-0.13842700	-2.39170500	0.36276400
H	-0.01774800	-2.77108300	1.38066000
H	-1.09457100	-2.75118300	-0.02283700
H	0.64480900	-2.79664600	-0.28084400
C	-1.10035400	0.15297100	1.81946800
H	-1.96459900	-0.48658000	1.99411700
H	-0.44180900	0.11273700	2.69083900
H	-1.46223600	1.16830900	1.68009900
C	-0.14191200	0.08291100	-1.46697100
H	0.86154500	-0.08744900	-1.86603700
H	-0.85253700	-0.51867900	-2.03422100
H	-0.40570100	1.13237400	-1.57619700
N	-2.94970500	-0.01812700	-0.47835800
S	-3.79338600	-1.24901400	-1.09712300
S	-3.28139400	1.52943900	-0.76907900
C	-4.62613200	1.93489700	0.41551600
C	-4.38740700	-2.01871500	0.46267800
O	-3.80670900	1.82614200	-2.09008300
O	-2.14521900	2.30830100	-0.26246400
O	-4.99543700	-0.89819400	-1.83411600
O	-2.85891600	-2.23524700	-1.63632300
F	-5.10713000	-1.15236600	1.16921000
F	-5.13289600	-3.08048600	0.17790200
F	-3.34660800	-2.41439700	1.20892900
F	-5.74335000	1.29816000	0.09143900
F	-4.25855200	1.57525400	1.64694400
F	-4.84517300	3.24722300	0.39754500

Absolute Energy: -3458,119685 u.a.
Number of Imaginary Frequencies: 0

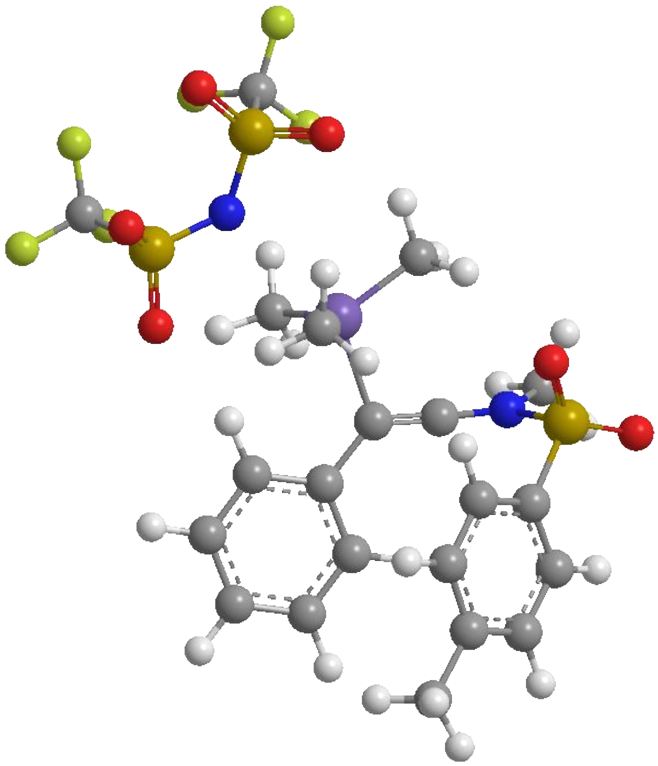

Model compound 2a

C	-2.93992100	-0.31235900	0.04216200
H	-2.91933400	-1.33411100	-0.32173800
H	-3.77155800	-0.02956300	0.67469100
C	-1.98456400	0.55687100	-0.28518900
H	-2.04682400	1.57081000	0.10227600
C	-0.77398100	0.24171700	-1.10750900
H	-0.93294100	-0.66929100	-1.69195700
H	-0.55770500	1.05258700	-1.80957000
Si	0.74181500	-0.02439200	0.01647600
C	0.91269400	1.44560300	1.18806300
H	0.04651900	1.51812400	1.84910500
H	1.80315600	1.34158500	1.81210300
H	0.99842300	2.38483400	0.63653400
C	0.47924800	-1.60721200	1.00520900
H	0.44829400	-2.47918800	0.34776400
H	1.28325600	-1.75913400	1.72862800
H	-0.46624200	-1.56044700	1.54930600
C	2.28940500	-0.17221900	-1.05548800
H	2.45748400	0.74181500	-1.62944200
H	3.17458500	-0.34988000	-0.44056400
H	2.20018300	-1.00106000	-1.76128900

Absolute Energy: -526,505785 u.a.
Number of Imaginary Frequencies: 0

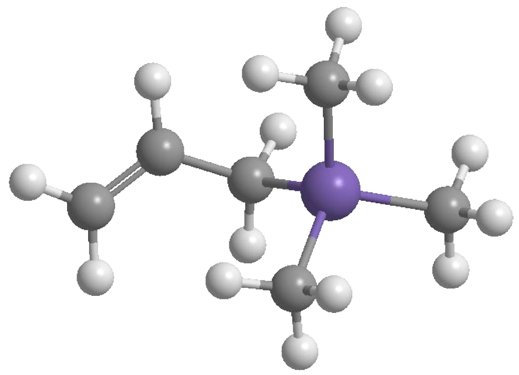

Model compound C_{anti}

C	0.76272000	0.05440100	-0.17528600
C	1.44173600	-0.96557700	0.15742100
C	1.12028600	1.44070900	-0.50088000
C	2.47252100	1.80472100	-0.60063300
C	0.13011400	2.39324400	-0.76235500
C	2.82514100	3.09982400	-0.95011400
H	3.24214000	1.06292000	-0.41629700
C	0.49215900	3.69121200	-1.10884800
H	-0.92144600	2.14472300	-0.70663400
C	1.83293700	4.04715600	-1.20300300
H	3.87124000	3.37043200	-1.02071300
H	-0.28482700	4.41720400	-1.30764000
H	2.10742100	5.05856100	-1.47427000
N	2.04291500	-2.08023500	0.33992400
C	2.22308500	-2.75041800	1.64474500
H	3.24782700	-3.10127100	1.72029800
H	2.00992100	-2.01015400	2.41191800
H	1.53334500	-3.58951900	1.70696700
S	2.60016300	-2.93952700	-1.13003700
C	3.54332400	-1.66716900	-1.89614600
C	2.93341900	-0.86718700	-2.85882400
C	4.85010200	-1.45066400	-1.46748600
C	3.65644500	0.19335900	-3.38636200
H	1.91906100	-1.07213100	-3.17459000
C	5.55260200	-0.38309300	-2.01210600
H	5.30239200	-2.10691600	-0.73453800
C	4.96404800	0.45532000	-2.96520800
H	3.19364600	0.83781200	-4.12331800
H	6.57162500	-0.19699300	-1.69501600
C	5.72266200	1.62057800	-3.54089100
H	6.60228700	1.85477100	-2.94180700
H	6.05482200	1.39605700	-4.55699700
H	5.08723900	2.50619100	-3.58863500
O	1.41762500	-3.21444600	-1.91909300
O	3.45703400	-3.97794600	-0.59119800
Si	-1.21756200	-0.55105600	-0.12175600
C	-1.20129700	-2.36673800	0.34392800
H	-0.86663000	-2.52419300	1.37202400
H	-2.23006600	-2.72332900	0.26200700
H	-0.59595700	-2.95981700	-0.34402400
C	-1.67536600	0.49733900	1.37501800
H	-2.47671600	-0.02199500	1.89929300
H	-0.80942800	0.58257200	2.03731400
H	-2.03763000	1.48748500	1.11168500
C	-1.57510600	-0.36403000	-1.95406700
H	-0.71107400	-0.73318900	-2.51373400
H	-2.43398000	-0.99636000	-2.18077000
H	-1.80129500	0.65611500	-2.25523600
N	-4.04955700	-0.04198300	-0.30024500
S	-5.05746500	-1.30302600	-0.38327400
S	-4.41006000	1.42265800	-0.86312200
C	-5.40740100	2.19294700	0.47430100
C	-5.24233300	-1.65428500	1.41144800
O	-5.24123600	1.45414700	-2.05350500
O	-3.16744300	2.20284100	-0.83981700
O	-6.40084400	-1.01974900	-0.85889900
O	-4.32398700	-2.46060700	-0.89082600
F	-5.73113500	-0.59476500	2.04904200
F	-6.06282000	-2.68526400	1.57909800
F	-4.05343100	-1.96136100	1.94835200
F	-6.58271000	1.59037000	0.59099400
F	-4.74966300	2.09791000	1.63159400
F	-5.59729300	3.47939300	0.19173700
C	2.56348200	0.30611500	3.88285000
H	2.89259400	-0.44384600	4.59515200
H	1.69966500	0.89961500	4.15389900
C	3.20965300	0.50104400	2.73176400
H	2.84731300	1.27447400	2.05862700
C	4.44241800	-0.23298600	2.30295200
H	4.61814000	-1.09793500	2.95066400
H	4.34101600	-0.60227300	1.27488000
Si	5.99778700	0.87134700	2.35484600
C	5.78588600	2.28883600	1.12564700
H	4.89662600	2.88138600	1.35138000
H	6.65029000	2.95614200	1.15256200
H	5.68936400	1.90444300	0.10575900
C	6.22033800	1.53247800	4.10133400
H	6.36131500	0.71551800	4.81273500
H	7.09017300	2.18940600	4.16681000
H	5.33975100	2.09777000	4.41210500
C	7.47547700	-0.18642600	1.84196100
H	7.32945400	-0.60817200	0.84385100
H	8.39335300	0.40517500	1.82248400
H	7.62809800	-1.01561700	2.53610600

Absolute Energy: -3984,634673 u.a.
Number of Imaginary Frequencies: 0

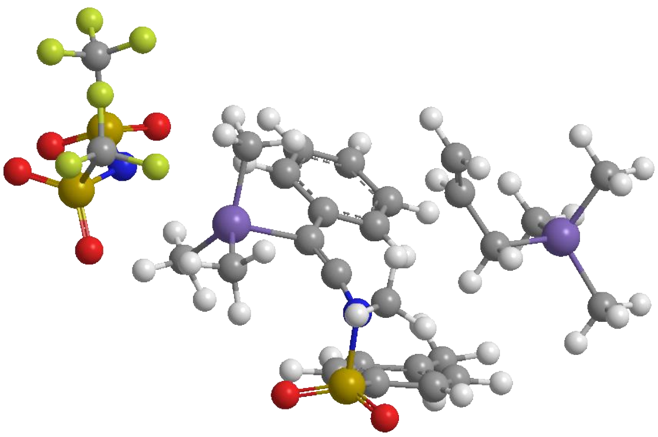

Model compound $TS2_{anti}$

C	-0.97410500	0.25432900	0.01883900
C	-1.91048200	-0.35664900	-0.68106600
C	-1.07435800	1.60039300	0.59977600
C	-2.16639700	1.90253400	1.42722500
C	-0.04937000	2.54536800	0.44999900
C	-2.25431800	3.14142800	2.05353600
H	-2.91755600	1.14427600	1.60840900
C	-0.14965500	3.78415100	1.06966000
H	0.83855700	2.31986900	-0.12402600
C	-1.25149700	4.08851700	1.86634400
H	-3.09780000	3.35897300	2.69754800
H	0.65361600	4.49800900	0.94469300
H	-1.31505300	5.05043400	2.35868800
N	-2.33713700	-1.56503400	-0.99332000
C	-2.35646900	-2.09959400	-2.36378600
H	-3.33218500	-1.95751300	-2.82852200
H	-1.57630300	-1.59840500	-2.93163700
H	-2.12693000	-3.16336100	-2.31405600
S	-3.07680300	-2.68298300	0.15496400
C	-3.12530000	-1.78662700	1.66562900
C	-1.97351100	-1.68993200	2.44490000
C	-4.33977900	-1.24868900	2.07644800
C	-2.03965800	-0.98533500	3.63638900
H	-1.05194500	-2.15731900	2.12557400
C	-4.38516700	-0.55758100	3.28270400
H	-5.22485600	-1.39605200	1.47338900
C	-3.23807100	-0.40044000	4.06429000
H	-1.14986100	-0.88770500	4.24615100
H	-5.32454600	-0.14095100	3.62474900
C	-3.27120900	0.39795100	5.33858500
H	-4.29381500	0.59094300	5.66078200
H	-2.74639200	-0.12348000	6.13975200
H	-2.77143500	1.35828700	5.18813900
O	-2.16392300	-3.80643100	0.25719600
O	-4.42741300	-2.87999500	-0.35577500
Si	0.83708600	-0.58408400	0.04343800
C	0.75116300	-2.44196500	-0.20023900
H	0.30538800	-2.71335300	-1.15939800
H	1.79568000	-2.76847500	-0.20923600
H	0.24080700	-3.00125500	0.58191600
C	1.50414900	0.14405300	-1.56627100
H	2.27465800	-0.53117400	-1.93690300
H	0.69822100	0.20474400	-2.30419300
H	1.97049400	1.11930200	-1.44535300
C	1.45012000	-0.12817600	1.75527800
H	0.63530000	-0.22892200	2.47760200
H	2.26422300	-0.80176900	2.02238400
H	1.82046200	0.89511100	1.78877500
N	3.97863000	0.01578000	0.08920500
S	4.87590200	-1.26451500	0.46485500
S	4.38115800	1.53724400	0.38826900
C	5.47732800	1.99510500	-1.01222300
C	5.12827000	-1.95887500	-1.21719800
O	5.16125300	1.76610500	1.59345900
O	3.18239200	2.35154700	0.15332900
O	6.21182500	-0.99713700	0.97392800
O	4.03233000	-2.26541500	1.11932200
F	5.71030300	-1.06669500	-2.01351900
F	5.89463400	-3.04239500	-1.14027200
F	3.95219900	-2.30791800	-1.75760600
F	6.62399000	1.32991500	-0.94669800
F	4.87304200	1.70869500	-2.16707900
F	5.72598700	3.30295000	-0.97112000
C	-2.90475600	1.00943900	-1.83915000
H	-2.96995600	0.35829600	-2.70388700
H	-2.14703100	1.78096900	-1.87023500
C	-3.99061900	1.15575700	-1.01833000
H	-3.98206600	1.98739300	-0.32320000
C	-5.19280000	0.31544300	-1.05313000
H	-4.99187400	-0.70596500	-1.38627200
H	-5.69272100	0.28239000	-0.08372300
Si	-6.45894700	1.06923100	-2.30855200
C	-6.74781400	2.87436200	-1.86256900
H	-5.83079200	3.45895100	-1.96389300
H	-7.49747500	3.31561700	-2.52290200
H	-7.10707800	2.97838400	-0.83628000
C	-5.73240500	0.91572100	-4.03756400
H	-5.49488200	-0.12338700	-4.27840800
H	-6.45128100	1.26998600	-4.77979100
H	-4.82451200	1.51225500	-4.14599600
C	-8.03909700	0.06212100	-2.14736900
H	-8.46041300	0.14694300	-1.14356000
H	-8.79189300	0.41245300	-2.85695600
H	-7.85352600	-0.99491500	-2.34840600

Absolute Energy: -3984,621320 u.a.
Number of Imaginary Frequencies: 1 (255i)

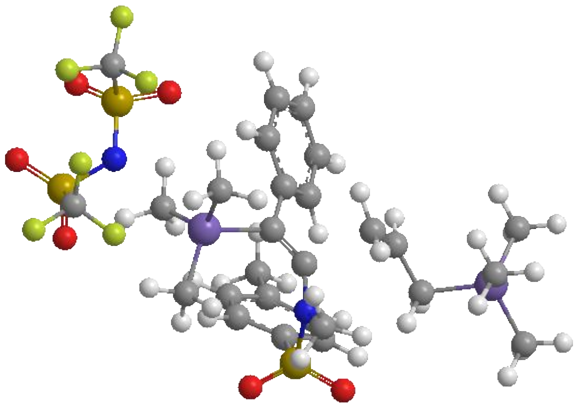

Model compound D$_{anti}$

C	-1.15827200	-0.05561700	-0.06295600
C	-2.40391100	-0.19454400	-0.76552100
C	-0.92484400	1.07416500	0.81341500
C	-1.89541000	1.39849900	1.78602300
C	0.31166700	1.74795000	0.83256500
C	-1.62315600	2.36006100	2.74515900
H	-2.82386800	0.84492500	1.82134600
C	0.55368100	2.74887600	1.75955900
H	1.07978200	1.52768200	0.10658100
C	-0.40786600	3.04667000	2.72292300
H	-2.35889700	2.57656200	3.50937600
H	1.50763000	3.25693400	1.73155900
H	-0.20634600	3.80677000	3.46687000
N	-2.74255800	-1.52038700	-1.19180900
C	-2.71981400	-1.93485600	-2.59746800
H	-3.56178900	-1.53802900	-3.17088200
H	-1.78020900	-1.61210800	-3.04226900
H	-2.74922800	-3.02253500	-2.62454000
S	-3.78601300	-2.47299500	-0.24968600
C	-3.49424800	-1.88635700	1.39453100
C	-2.29874500	-2.20180300	2.03844800
C	-4.51414800	-1.20578100	2.04865300
C	-2.11047100	-1.77444800	3.34479100
H	-1.54160100	-2.78619500	1.53193800
C	-4.31066500	-0.79738400	3.36431700
H	-5.45043300	-1.02571900	1.53780100
C	-3.10700800	-1.06047600	4.02135100
H	-1.18177300	-2.00549900	3.85271500
H	-5.10016900	-0.27265600	3.88853700
C	-2.86657700	-0.58095800	5.42746500
H	-3.78415900	-0.20895500	5.88202100
H	-2.47281700	-1.38449500	6.05119100
H	-2.13040100	0.22673700	5.42883900
O	-3.28299400	-3.83572000	-0.37877200
O	-5.18186300	-2.20509400	-0.58793500
Si	0.41685200	-1.22573600	-0.39198400
C	0.01741200	-2.97406200	-0.94690900
H	-0.26047100	-3.00257200	-2.00058500
H	0.95804300	-3.52089700	-0.84287800
H	-0.75800800	-3.49059500	-0.38334800
C	1.06090500	-0.29231900	-1.91192400
H	1.86387300	-0.90516800	-2.32232500
H	0.25390700	-0.22541500	-2.64846400
H	1.47848200	0.69599500	-1.73277000
C	1.23481700	-1.24366100	1.30159800
H	0.47229900	-1.18765700	2.08265600
H	1.78592200	-2.17812900	1.40433900
H	1.93674200	-0.42745900	1.47187600
N	3.81617200	0.25718500	-0.61694900
S	4.26647200	-1.25209300	-0.30657400
S	3.93783500	1.48617800	0.39741000
C	5.69665100	2.01036700	0.32587100
C	5.56457300	-1.47020200	-1.58476000
O	3.68883000	1.17003800	1.80236300
O	3.19576700	2.60358100	-0.19259900
O	4.91345600	-1.49300800	0.97541500
O	3.20371500	-2.16722200	-0.74559900
F	6.52915800	-0.57012600	-1.41557600
F	6.08109200	-2.69125200	-1.47629900
F	5.04002100	-1.32557700	-2.79870700
F	6.47617100	1.06956800	0.84466900
F	6.05493000	2.23080600	-0.93529700
F	5.84157300	3.13547700	1.02357100
C	-2.34416700	0.94275800	-1.87755400
H	-2.55084500	0.55434900	-2.86669300
H	-1.51192100	1.63190500	-1.81376600
C	-3.42507400	0.98462300	-0.89052100
H	-3.30494500	1.73982300	-0.12761000
C	-4.85234800	0.73019700	-1.30741700
H	-4.91768900	-0.09746700	-2.01642400
H	-5.44862300	0.42881100	-0.44391300
Si	-5.62699800	2.28579500	-2.09407300
C	-5.49516000	3.72141500	-0.87684300
H	-4.45484900	3.98556200	-0.67277800
H	-5.98519600	4.60977200	-1.28139800
H	-5.97772300	3.48049600	0.07323400
C	-4.69675800	2.70618800	-3.68133700
H	-4.68906300	1.86115400	-4.37418500
H	-5.17775300	3.54468000	-4.19011500
H	-3.66253700	2.99545200	-3.48124400
C	-7.43147800	1.90726300	-2.47377400
H	-7.97984600	1.65388700	-1.56404300
H	-7.92119800	2.76919000	-2.93209300
H	-7.51960400	1.06496800	-3.16318500

Absolute Energy: -3984,637277 u.a.
Number of Imaginary Frequencies: 0

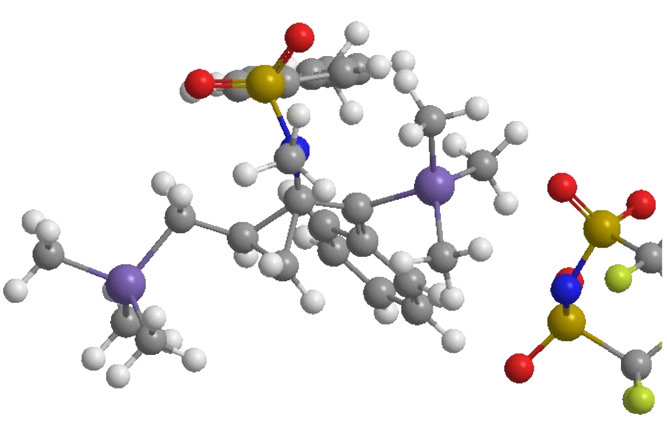

Model compound E_{anti}

C	-3.25028200	1.29540800	0.23879200
C	-3.30771500	0.41528300	1.24994200
C	-1.94157500	1.60511400	-0.40888700
C	-1.65490800	1.10924500	-1.68602300
C	-1.00497500	2.43400100	0.21608800
C	-0.45154900	1.41766400	-2.31299400
H	-2.37557300	0.46069300	-2.17105900
C	0.19787700	2.74401100	-0.41161300
H	-1.22862700	2.83928100	1.19626400
C	0.47875200	2.23624400	-1.67734000
H	-0.24004500	1.01826900	-3.29713100
H	0.91519100	3.38478400	0.08624100
H	1.41536100	2.47780200	-2.16358800
N	-4.59196700	0.19759700	1.87630500
C	-4.69978200	0.73012000	3.24531000
H	-4.07146600	0.19312600	3.96180100
H	-4.38833000	1.77273100	3.20300700
H	-5.73859600	0.67201600	3.55939600
S	-5.24930700	-1.35100700	1.73095200
C	-5.55718100	-1.50124000	-0.00740000
C	-6.84073200	-1.24618900	-0.47836800
C	-4.54607600	-1.94939100	-0.85149600
C	-7.10564100	-1.42382400	-1.83072000
H	-7.61450900	-0.94201100	0.21379600
C	-4.82978600	-2.11221500	-2.20229000
H	-3.56769200	-2.18138600	-0.45008500
C	-6.10594000	-1.85000700	-2.70904500
H	-8.10329800	-1.23682200	-2.20948800
H	-4.05365900	-2.46298400	-2.87229200
C	-6.39305000	-1.99619300	-4.18019600
H	-5.72680900	-2.72510800	-4.64169500
H	-7.42266100	-2.31159200	-4.35063600
H	-6.24884700	-1.04021600	-4.69044900
O	-6.53058900	-1.29210300	2.42834300
O	-4.26940500	-2.37333500	2.09957700
Si	-4.68731400	2.34932500	-0.44758300
C	-6.26196500	2.37945300	0.58428000
H	-6.06509800	2.71897300	1.60254900
H	-6.95988500	3.08601500	0.12565500
H	-6.74030200	1.40313500	0.64234900
C	-4.01078800	4.11281800	-0.51949100
H	-4.77890700	4.78850300	-0.90396100
H	-3.71899400	4.46792300	0.47191200
H	-3.13867800	4.18118100	-1.17199500
C	-5.07982400	1.78705200	-2.20547100
H	-5.33826400	0.72781900	-2.23739100
H	-5.93086000	2.35777700	-2.58628200
H	-4.23385700	1.96194500	-2.87273500
C	-2.10055000	-0.29762100	1.86076500
H	-2.42187200	-0.85469100	2.73944400
H	-1.38623500	0.46453600	2.18962000
C	-1.39174600	-1.24210000	0.92183000
H	-0.96272500	-0.79718800	0.03001600
C	-1.25399600	-2.54812100	1.14532000
H	-1.69774200	-3.02082000	2.01284100
H	-0.71954800	-3.18419700	0.44837300
Si	2.81866200	-1.15517600	0.85463700
C	2.09623700	-1.27376700	-0.86450200
H	1.08025400	-1.66770900	-0.78658700
H	2.03483900	-0.28448400	-1.32081900
H	2.67672500	-1.93574500	-1.50440000
C	1.90994000	0.10430300	1.89300500
H	2.32161100	0.17485700	2.89770300
H	1.85773800	1.09875500	1.45697000
H	0.89156100	-0.29152400	1.95757000
C	2.89180000	-2.75222400	1.82096000
H	1.89058200	-2.95281400	2.21257700
H	3.21495500	-3.59531100	1.21508000
H	3.56834800	-2.64996200	2.67254100
N	4.62032300	-0.56914600	0.58676200
S	5.66658600	-1.68767400	-0.13467800
S	5.24522400	0.84343100	1.24952100
O	4.80947200	-2.80093400	-0.50013000
O	6.88705600	-1.87325200	0.61002100
O	4.56701300	1.11605400	2.49750500
O	6.68373300	0.87221100	1.11998800
C	6.10026600	-0.90611900	-1.75380300
C	4.62657200	2.11320700	0.05519400
F	6.56954800	-1.86910900	-2.53251200
F	4.99613300	-0.39503600	-2.29168300
F	7.01012800	0.03738000	-1.60692200
F	5.33368900	2.06297600	-1.06274800
F	3.34141400	1.90566900	-0.23579300
F	4.76130300	3.30174400	0.62170400

Absolute Energy: -3984,701262 u.a.
Number of Imaginary Frequencies: 0

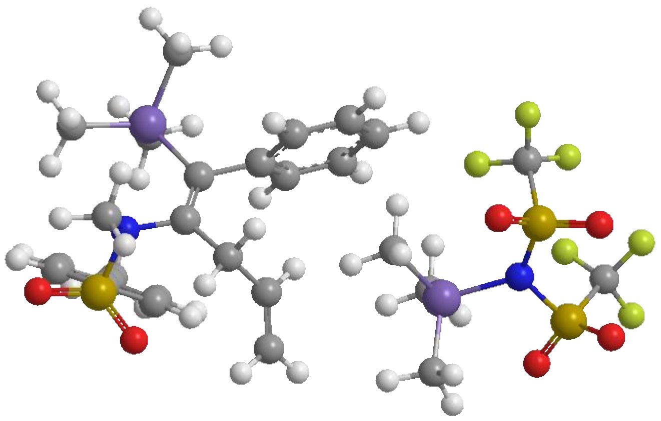

Model compound C_{syn}

C	-1.53806600	-0.41763300	-1.37723700
C	-2.92194600	-0.19275100	-1.11927600
C	-1.02575800	-1.75624700	-1.58545600
C	-1.77992500	-2.77491400	-2.21509400
C	0.29484900	-2.06183200	-1.18855500
C	-1.22662600	-4.02304900	-2.43725600
H	-2.78020900	-2.57721300	-2.56125300
C	0.83029100	-3.32543000	-1.37169500
H	0.89342200	-1.33288300	-0.66707300
C	0.07060500	-4.30620000	-2.00350000
H	-1.80982900	-4.78310600	-2.93935100
H	1.84101400	-3.51464800	-1.03804700
H	0.48800400	-5.29174600	-2.16616200
N	-3.83279400	-1.26501100	-0.90123300
C	-4.93181700	-1.52661500	-1.83324900
H	-5.81514600	-0.91732300	-1.62283500
H	-4.58780600	-1.33035200	-2.84868900
H	-5.20364500	-2.57878100	-1.75864800
S	-4.05619600	-2.10224800	0.57412300
C	-2.60789200	-1.80536000	1.53616100
C	-1.47623400	-2.59118200	1.32490600
C	-2.66464200	-0.88853700	2.58279800
C	-0.35116300	-2.37132500	2.10641400
H	-1.48584300	-3.35870100	0.56262900
C	-1.53285000	-0.69262000	3.36427400
H	-3.59143400	-0.37775400	2.80044600
C	-0.35677500	-1.40798200	3.12213200
H	0.55467300	-2.93419100	1.91870000
H	-1.56204500	0.02212700	4.17753200
C	0.88388000	-1.17181500	3.93776300
H	0.76310800	-0.31648400	4.60190000
H	1.11132000	-2.04979600	4.54675400
H	1.73621800	-0.99956600	3.27866300
O	-4.10207300	-3.50963900	0.18951000
O	-5.20781400	-1.52302800	1.26398100
Si	-0.13488000	1.00679700	-1.38808200
C	-0.71379800	2.69610400	-2.00776200
H	-1.51479100	3.19269800	-1.46170700
H	0.18998300	3.30423400	-1.90315200
H	-0.95989800	2.67795800	-3.07176700
C	0.22581400	1.06605500	0.46398000
H	0.72402000	2.01242400	0.67586500
H	-0.69535100	0.99241100	1.04728400
H	0.90055100	0.27717900	0.79320700
C	1.10105100	0.45085900	-2.69190900
H	0.57379100	-0.06242200	-3.50109300
H	1.57508500	1.34571500	-3.09574500
H	1.89538900	-0.20638700	-2.34009500
N	3.33556800	0.56370100	0.34022500
S	3.69417600	1.68629600	-0.75309900
S	3.60091900	-0.99829500	0.17657400
C	5.34427500	-1.24472200	0.69564900
C	4.82721900	2.73472600	0.23830000
O	3.53226100	-1.51936500	-1.18893800
O	2.80380600	-1.68881400	1.19588300
O	4.46324000	1.24434800	-1.90819300
O	2.52102200	2.54111600	-0.97193200
F	5.86901200	2.01522500	0.64591200
F	5.25778200	3.74024100	-0.51891500
F	4.19429900	3.22742000	1.29831100
F	6.16083600	-0.65167700	-0.16607000
F	5.53338100	-0.72954100	1.90667500
F	5.60962000	-2.54889200	0.72997800
C	-3.49965600	1.07606300	-1.86023800
H	-4.52337900	0.92712900	-2.17954700
H	-2.84302200	1.50854800	-2.59895900
C	-3.27545200	1.26733400	-0.44803200
H	-2.37351300	1.77895300	-0.14608200
C	-4.43318000	1.38316700	0.48861500
H	-5.26479100	0.74319900	0.18766200
H	-4.15152500	1.09305300	1.49939600
Si	-5.03104600	3.20325800	0.54832800
C	-3.62104600	4.28142400	1.18203500
H	-2.76729900	4.28394200	0.50004100
H	-3.95273500	5.31617600	1.29306700
H	-3.27033000	3.93923900	2.15835300
C	-5.53258000	3.74700600	-1.18683900
H	-6.29280800	3.08633600	-1.61054900
H	-5.95151300	4.75543300	-1.15796900
H	-4.67683000	3.76720600	-1.86586400
C	-6.50172800	3.27290300	1.71967800
H	-6.22179100	2.93958100	2.72107700
H	-6.88367900	4.29295800	1.80013900
H	-7.31518500	2.63470400	1.36876000

Absolute Energy: -3984,631985 u.a.
Number of Imaginary Frequencies: 0

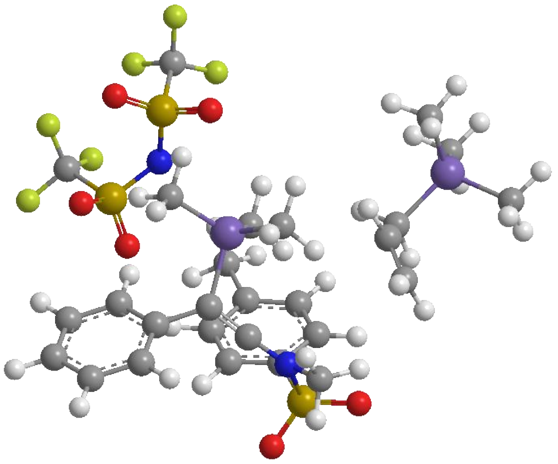

Model compound TS2$_{syn}$

C	-1.42078000	-0.82722500	-1.50589900
C	-2.68806200	-0.91196600	-1.16429800
C	-0.77707000	-2.18338200	-1.61228800
C	-1.39599900	-3.23164600	-2.29945700
C	0.46684600	-2.40098200	-1.01195000
C	-0.78432700	-4.47802500	-2.37386800
H	-2.34349800	-3.06534300	-2.79761000
C	1.07737000	-3.64638200	-1.08812200
H	0.95869800	-1.61422800	-0.45800600
C	0.45110300	-4.68834400	-1.76699600
H	-1.26957600	-5.28075100	-2.91384200
H	2.04785300	-3.77665200	-0.62817900
H	0.92895500	-5.65707700	-1.83367900
N	-3.67486400	-1.61824800	-0.71339500
C	-4.79512900	-2.07718400	-1.55330300
H	-5.71909700	-1.60692700	-1.21849900
H	-4.57952100	-1.81024200	-2.58407100
H	-4.87118000	-3.15895000	-1.45586700
S	-3.84112700	-2.23167700	0.97059700
C	-2.41491500	-1.66380600	1.79577600
C	-1.22502200	-2.37463400	1.64749600
C	-2.51863100	-0.55348100	2.63239400
C	-0.09431500	-1.91067000	2.30190600
H	-1.18814000	-3.26130600	1.02849300
C	-1.37570800	-0.11606900	3.28469500
H	-3.47595900	-0.07386000	2.77862000
C	-0.14767000	-0.76534500	3.10670900
H	0.85344700	-2.41347700	2.16382300
H	-1.43011100	0.74545300	3.93868400
C	1.10428400	-0.22669400	3.73734500
H	0.87343000	0.41829800	4.58510200
H	1.75846700	-1.03452200	4.06229600
H	1.66525800	0.34856900	2.99598600
O	-3.81059600	-3.67052200	0.78252400
O	-5.03762300	-1.57205200	1.47032100
Si	-0.18000200	0.69653200	-1.72366000
C	-0.84679200	2.14135200	-2.74126600
H	-1.65189200	2.73904400	-2.31878300
H	0.02925200	2.79112700	-2.83479600
H	-1.12259100	1.82845300	-3.75083900
C	0.09469200	1.16944200	0.07483600
H	0.55187000	2.15730300	0.12384400
H	-0.85176400	1.16127400	0.62078900
H	0.78391200	0.48118400	0.56442000
C	1.20404800	0.01256800	-2.79252000
H	0.78243400	-0.63777300	-3.56417000
H	1.69370900	0.85765100	-3.27737900
H	1.97277300	-0.55063700	-2.26572500
N	3.25732000	0.68630000	0.26762700
S	3.66632700	1.69793800	-0.91751200
S	3.66661800	-0.85551100	0.32461200
C	5.38566800	-0.88427600	0.97105200
C	4.62476900	2.92982500	0.04757800
O	3.73059900	-1.55015300	-0.95885000
O	2.86984800	-1.47280100	1.39058300
O	4.58362100	1.17540000	-1.92052800
O	2.48385400	2.44454400	-1.35760200
F	5.64969300	2.33770500	0.65530700
F	5.08743800	3.86396700	-0.77929300
F	3.84991000	3.50576900	0.96321500
F	6.21799200	-0.34751700	0.08851400
F	5.45312500	-0.19997400	2.11053400
F	5.74179600	-2.14669700	1.20133500
C	-3.77433700	0.98698500	-1.87422000
H	-4.73405600	0.50872900	-2.03591900
H	-3.15262800	1.15109900	-2.74134100
C	-3.50740000	1.57525100	-0.68265600
H	-2.58640200	2.13813200	-0.57969000
C	-4.41534000	1.58638600	0.47768400
H	-5.06865600	0.71015000	0.50949300
H	-3.85920300	1.65430800	1.41377200
Si	-5.54521800	3.14635200	0.37593900
C	-4.45781900	4.66046300	0.11337600
H	-3.93116800	4.60786500	-0.84233300
H	-5.06191900	5.57032200	0.10948200
H	-3.71275900	4.75947200	0.90600700
C	-6.72789200	2.92080200	-1.07115100
H	-7.34148500	2.02576300	-0.94524200
H	-7.40085600	3.77778300	-1.14727100
H	-6.18718200	2.83810500	-2.01613800
C	-6.48571300	3.25906900	2.00206700
H	-5.80220900	3.39456200	2.84289400
H	-7.17379200	4.10720700	1.99100700
H	-7.06996600	2.35437500	2.18238000

Absolute Energy: -3984,613714 u.a.
Number of Imaginary Frequencies: 1 (233i)

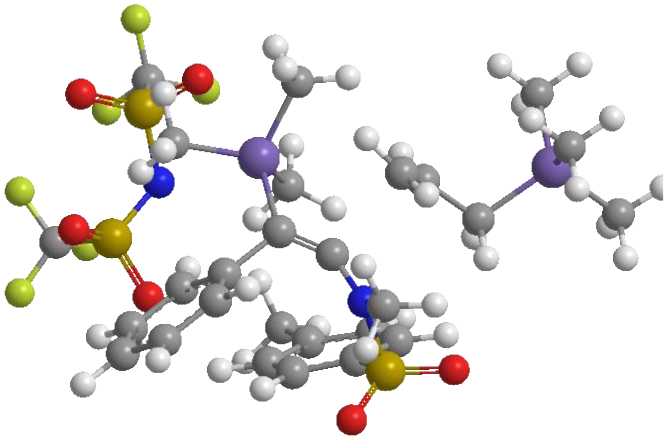

Model compound D_{syn}

C	-1.53806600	-0.41763300	-1.37723700
C	-2.92194600	-0.19275100	-1.11927600
C	-1.02575800	-1.75624700	-1.58545600
C	-1.77992500	-2.77491400	-2.21509400
C	0.29484900	-2.06183200	-1.18855500
C	-1.22662600	-4.02304900	-2.43725600
H	-2.78020900	-2.57721300	-2.56125300
C	0.83029100	-3.32543000	-1.37169500
H	0.89342200	-1.33288300	-0.66707300
C	0.07060500	-4.30620000	-2.00350000
H	-1.80982900	-4.78310600	-2.93935100
H	1.84101400	-3.51464800	-1.03804700
H	0.48800400	-5.29174600	-2.16616200
N	-3.83279400	-1.26501100	-0.90123300
C	-4.93181700	-1.52661500	-1.83324900
H	-5.81514600	-0.91732300	-1.62283500
H	-4.58780600	-1.33035200	-2.84868900
H	-5.20364500	-2.57878100	-1.75864800
S	-4.05619600	-2.10224800	0.57412300
C	-2.60789200	-1.80536000	1.53616100
C	-1.47623400	-2.59118200	1.32490600
C	-2.66464200	-0.88853700	2.58279800
C	-0.35116300	-2.37132500	2.10641400
H	-1.48584300	-3.35870100	0.56262900
C	-1.53285000	-0.69262000	3.36427400
H	-3.59143400	-0.37775400	2.80044600
C	-0.35677500	-1.40798200	3.12213200
H	0.55467300	-2.93419100	1.91870000
H	-1.56204500	0.02212700	4.17753200
C	0.88388000	-1.17181500	3.93776300
H	0.76310800	-0.31648400	4.60190000
H	1.11132000	-2.04979600	4.54675400
H	1.73621800	-0.99956600	3.27866300
O	-4.10207300	-3.50963900	0.18951000
O	-5.20781400	-1.52302800	1.26398100
Si	-0.13488000	1.00679700	-1.38808200
C	-0.71379800	2.69610400	-2.00776200
H	-1.51479100	3.19269800	-1.46170700
H	0.18998300	3.30423400	-1.90315200
H	-0.95989800	2.67795800	-3.07176700
C	0.22581400	1.06605500	0.46398000
H	0.72402000	2.01242400	0.67586500
H	-0.69535100	0.99241100	1.04728400
H	0.90055100	0.27717900	0.79320700
C	1.10105100	0.45085900	-2.69190900
H	0.57379100	-0.06242200	-3.50109300
H	1.57508500	1.34571500	-3.09574500
H	1.89538900	-0.20638700	-2.34009500
N	3.33556800	0.56370100	0.34022500
S	3.69417600	1.68629600	-0.75309900
S	3.60091900	-0.99829500	0.17657400
C	5.34427500	-1.24472200	0.69564900
C	4.82721900	2.73472600	0.23830000
O	3.53226100	-1.51936500	-1.18893800
O	2.80380600	-1.68881400	1.19588300
O	4.46324000	1.24434800	-1.90819300
O	2.52102200	2.54111600	-0.97193200
F	5.86901200	2.01522500	0.64591200
F	5.25778200	3.74024100	-0.51891500
F	4.19429900	3.22742000	1.29831100
F	6.16083600	-0.65167700	-0.16607000
F	5.53338100	-0.72954100	1.90667500
F	5.60962000	-2.54889200	0.72997800
C	-3.49965600	1.07606300	-1.86023800
H	-4.52337900	0.92712900	-2.17954700
H	-2.84302200	1.50854800	-2.59895900
C	-3.27545200	1.26733400	-0.44803200
H	-2.37351300	1.77895300	-0.14608200
C	-4.43318000	1.38316700	0.48861500
H	-5.26479100	0.74319900	0.18766200
H	-4.15152500	1.09305300	1.49939600
Si	-5.03104600	3.20325800	0.54832800
C	-3.62104600	4.28142400	1.18203500
H	-2.76729900	4.28394200	0.50004100
H	-3.95273500	5.31617600	1.29306700
H	-3.27033000	3.93923900	2.15835300
C	-5.53258000	3.74700600	-1.18683900
H	-6.29280800	3.08633600	-1.61054900
H	-5.95151300	4.75543300	-1.15796900
H	-4.67683000	3.76720600	-1.86586400
C	-6.50172800	3.27290300	1.71967800
H	-6.22179100	2.93958100	2.72107700
H	-6.88367900	4.29295800	1.80013900
H	-7.31518500	2.63470400	1.36876000

Absolute Energy: -3984,639884 u.a.

Number of Imaginary Frequencies: 0

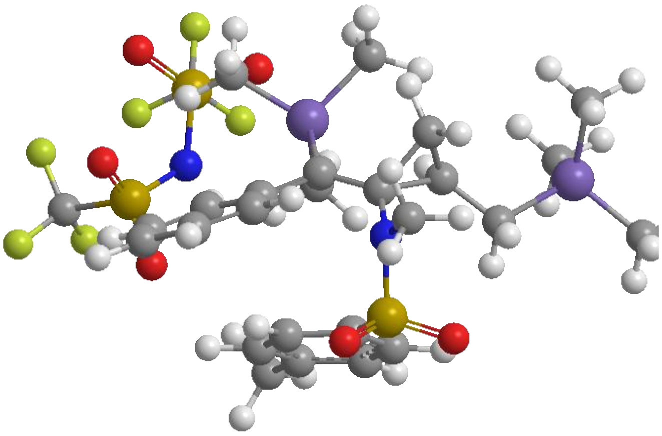

Model compound $\mathbf{E}_{syn}$

C	3.92242300	-1.22941100	0.35928900
C	4.06074900	-0.86607800	-0.92592600
C	2.57016800	-1.35046500	0.98460500
C	1.92210200	-2.59036700	1.00240700
C	1.98833100	-0.27860700	1.66671600
C	0.72471200	-2.75864400	1.69048200
H	2.37811000	-3.43174900	0.49270400
C	0.78673000	-0.44546200	2.35065700
H	2.47839700	0.68722900	1.65389700
C	0.15727700	-1.68730200	2.37483500
H	0.24121700	-3.72803700	1.70211100
H	0.33943600	0.40142700	2.85785200
H	-0.76901600	-1.81856000	2.91942300
N	2.85370100	-0.64006700	-1.69816300
C	2.52789700	-1.74816100	-2.60910600
H	3.32305200	-1.94425600	-3.33471300
H	2.37329500	-2.63297900	-1.99279600
H	1.60838500	-1.50726600	-3.13612400
S	2.64740900	0.89047000	-2.38681300
C	2.43263200	1.91960400	-0.96389400
C	1.14089600	2.21836300	-0.54757600
C	3.54924000	2.42337000	-0.30530700
C	0.96909300	3.01738100	0.57558900
H	0.28904800	1.84987300	-1.09948700
C	3.35553200	3.21180200	0.82370800
H	4.54188700	2.21154900	-0.68103500
C	2.06897700	3.51280800	1.28121300
H	-0.03478600	3.26174200	0.89943800
H	4.21467200	3.61184400	1.34942300
C	1.86971000	4.34894400	2.51891900
H	2.72071600	5.00822800	2.69055000
H	0.97024700	4.95976700	2.43767500
H	1.75961400	3.71121600	3.39994700
O	1.38479800	0.81852900	-3.11915900
O	3.85562200	1.32731000	-3.08608400
Si	5.30285100	-1.62000200	1.61935300
C	6.89637900	-2.33040100	0.89057800
H	7.46984200	-1.63669900	0.27716900
H	7.53066200	-2.63933200	1.72608000
H	6.68522600	-3.22302800	0.29702400
C	5.63090700	-0.03614500	2.59717800
H	6.40254900	-0.20740900	3.35161800
H	5.94825900	0.79265100	1.96227900
H	4.71785800	0.26974700	3.11331700
C	4.64821700	-2.91797600	2.82157500
H	4.40002400	-3.85200600	2.31291200
H	5.41755300	-3.13650600	3.56687400
H	3.75429800	-2.57144200	3.34200100
C	5.39260400	-0.79101300	-1.66699700
H	5.20494500	-0.68197900	-2.73406400
H	5.91700400	-1.73918200	-1.51838900
C	6.29330600	0.33429900	-1.22223100
H	6.55138600	0.36218100	-0.16820100
C	6.79584700	1.25638400	-2.03776200
H	6.53608400	1.27784900	-3.08857200
H	7.46289600	2.02743700	-1.67165900
Si	-2.56748800	-1.47511600	-1.22593600
C	-2.89109200	-2.89972200	-0.05651900
H	-2.13399600	-3.66793500	-0.23549300
H	-2.79615600	-2.57256200	0.97973100
H	-3.87609200	-3.33806300	-0.20534900
C	-0.82684500	-0.83538000	-1.03730000
H	-0.55652200	-0.16475200	-1.85261300
H	-0.59664800	-0.35822100	-0.08715000
H	-0.18531900	-1.72121400	-1.10265500
C	-2.87784900	-1.83343200	-3.03352200
H	-2.01378100	-2.37227700	-3.43152200
H	-3.77583800	-2.42409500	-3.19870600
H	-2.96217900	-0.89713500	-3.58982700
N	-3.80950800	-0.11060400	-0.72075800
S	-5.44698800	-0.44925500	-0.98167500
S	-3.34770000	1.41699300	-0.20747500
O	-5.45973900	-1.81853600	-1.46221300
O	-6.13720100	0.61289600	-1.66944600
O	-2.10305900	1.76906700	-0.85630400
O	-4.48310200	2.30879400	-0.16497400
C	-6.13781600	-0.55809900	0.73212700
C	-2.90768300	1.09625200	1.56736800
F	-7.24688600	-1.27681700	0.66330900
F	-5.25276500	-1.17401800	1.51355200
F	-6.40492500	0.64020400	1.21477600
F	-3.99009200	1.18477600	2.32234300
F	-2.37249900	-0.11380000	1.70879300
F	-2.02366900	2.01179300	1.94557800

Absolute Energy: -3984,699665 u.a.
Number of Imaginary Frequencies: 0

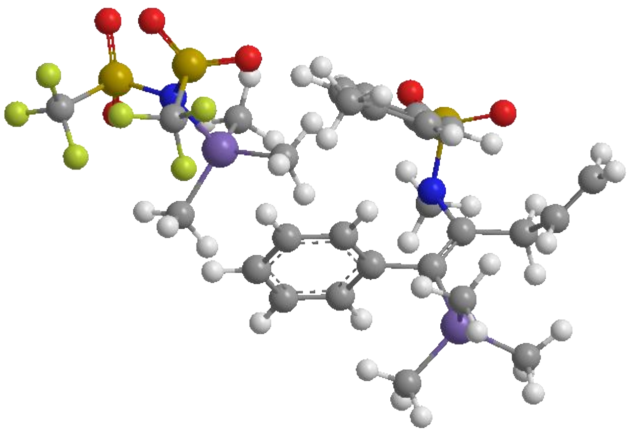

6.- References

(1) Zhang, Y.; Hsung, R. P.; Tracey, M. R.; Kurtz, K. C. M.; Vera E. L. *Org. Lett.* **2004**, *6*, 1151-1154.

(2) Hamada, T.; Ye, X.; Stahl, S. S. *J. Am. Chem. Soc.* **2008**, *130*, 823-835.

(3) Zeng, X.; Tu, Y.; Zhang, Z.; You, C.; Wu, J.; Ye, Z.; Zhao, J. *J. Org. Chem.* **2019**, *84*, 4458-4466.

(4) Wang, F.; Huang, Y.; Jing, J.; Wang, F.; Li, X. *Org. Lett.* **2022**, *24*, 2531-2535.

(5) Luo, W-F.; Ye, L.-W.; Li, L.; Qian, P.-C. *Chem. Commun.* **2021**, *57*, 5032-5035.

(6) Xu, W.; Chen, Y.; Wang, A.; Liu, Y. *Org. Lett.* **2019**, *21*, 7613-7619.

(7) Zhao, X.; Song, X.; Jin, H.; Zeng, Z.; Wang, Q.; Rudolph, M.; Rominger, F.; Hashmi, A. S. K. *Adv. Synth. Catal.* **2018**, *360*, 2720-2726.

(8) Gawade, S. A.; Huple, D. B.; Liu, R.-S. *J. Am. Chem. Soc.* **2014**, *136*, 2978-2981.

(9) Saito, N.; Saito, K.; Shiro, M.; Sato, Y. *Org. Lett.* **2011**, *13*, 2718-2721.

(10) Shimizu, R.; Egami, H.; Hamashima, Y.; Sodeoka, M. *Angew. Chem. Int. Ed.* **2012**, *51*, 4577-4580.

(11) Hou, Z.-L.; Yang, F.; Zhou, Z.; Ao, Y.-F.; Yao, B. *Tetrahedron Lett.* **2018**, *59*, 4557-4561

(12) Gaussian 16, Revision C.01, Gaussian, Inc., Wallingford CT, **2019**.

(13) Zhao, Y.; Truhlar, D. G. *Theor. Chem. Acc.* **2008**, *120*, 215.

(14) a) Ditchfield, R.; Hehre, W. J.; Pople, J. A. *J. Chem. Phys.* **1971**, *54*, 724; b) Rassolov, V. A.; Ratner, M. A.; Pople, J. A.; Redfern, P. C.; Curtiss, L. A. *J. Comp. Chem.* **2001**, *22*, 976.

(15) The performance of the M05-2X density functional with the small 6-31G(d) gauged against the MP2/cc-pvtz results justifies the application of this model chemistry to the experimentally investigated compounds, see: Großekappenberg, H.; Reißmann, M.; Schmidtmann, M.; Müller, T. *Organometallics* **2015**, *34*, 4952.

(16) Tomasi, J.; Mennucci, B.; Cammi, R. *Chem. Rev.* **2005**, *105*, 2999.

(17) Kim, Y.; Dateer, R. B.; Chang, S. *Org. Lett.* **2017**, *19*, 190-193.

(18) a) Klaer, A.; Saak, W.; Haase, D.; Müller, T. *J. Am. Chem. Soc.* **2008**, 14956–14957; b) Creary, X.; Kochly, E. D. *J. Org. Chem.* **2008**, *74*, 2134–2144; c) Ramachandran, P. V.; Nicponski, D. R.; Gagare, P. D. in: *Comprehensive Organic Synthesis II*, Vol. 2, (Eds.: Knochel, P.; Molander G.), Elsevier, Amsterdam **2014**, pp 71–147.
